# Associations Between Macronutrients From Different Dietary Sources and Serum Lipids in 24 639 UK Biobank Study Participants

**DOI:** 10.1161/ATVBAHA.120.315628

**Published:** 2021-05-27

**Authors:** Rebecca K. Kelly, Cody Z. Watling, Tammy Y.N. Tong, Carmen Piernas, Jennifer L. Carter, Keren Papier, Timothy J. Key, Aurora Perez-Cornago

**Affiliations:** 1 Cancer Epidemiology Unit (R.K.K., C.Z.W., T.Y.N.T., K.P., T.J.K., A.P.-C.), University of Oxford, United Kingdom.; 2 Clinical Trial Service Unit and Epidemiological Studies Unit (J.L.C.), University of Oxford, United Kingdom.; 3 Nuffield Department of Population Health and Nuffield Department of Primary Care Health Sciences (C.P.), University of Oxford, United Kingdom.

**Keywords:** biomarkers, cardiovascular diseases, diet, risk factors, triglycerides

## Abstract

**Objective:**

Macronutrients may relate differently with serum lipids depending on their source, and understanding this relationship is important for cardiovascular disease prevention. We aimed to investigate the associations between macronutrients and macronutrients from different sources with serum lipids in UK Biobank.

**Approach and Results:**

Serum lipids were obtained from serum collected at baseline in 24 639 participants with diet assessed using ≥2 twenty-four–hour dietary assessments completed at baseline and during follow-up. Multivariable linear regressions were conducted to calculate geometric mean concentrations of serum lipids by quintiles of macronutrients. We modeled the association between isoenergetic substitution of 5% energy intake from saturated fatty acids (SFA) with other macronutrients and serum lipids. Free sugar intake was positively associated with triglycerides (0.15 mmol/L geometric mean difference between highest and lowest quintile of intake [95% CI, 0.12–0.17 mmol/L]), whereas nonfree sugar intake was inversely associated with triglycerides (−0.08 [−0.10 to −0.05]). SFA intake was positively associated with LDL-C (low-density lipoprotein cholesterol; 0.17 [0.13–0.20]), omega-3 fatty acid intake was inversely associated with triglycerides (−0.15 [−0.17 to −0.12]). Modeled substitution of SFA with polyunsaturated fatty acids was associated with lower total cholesterol, LDL-C, and triglycerides.

**Conclusions:**

The relationship between carbohydrates and triglycerides may depend on their quality, and reducing free sugar intake may be important in cardiovascular disease prevention. Consistent with previous studies, SFA intake is associated with LDL-C and substitution of SFA intake with polyunsaturated fatty acids intake may be associated with a more favorable serum lipid profile.

## Abstract 2

Supplemental Digital Content is available in the text.

**Highlights**

- Intake of free sugars is positively associated with serum triglycerides and inversely associated with HDL-C (high-density lipoprotein cholesterol), whereas intake of nonfree sugars is inversely associated with triglycerides in this large, prospective cohort study.
- This suggests that types of carbohydrates relate differently with serum lipids, and that reducing intake of free sugars may be important in cardiovascular disease prevention.
- Consistent with previous studies, intake of saturated fatty acids is associated with LDL-C, and modeled substitution of intake of saturated fatty acids with intake of polyunsaturated fatty acids is associated with a more favorable serum lipid profile.

Dyslipidemia is a known risk factor for cardiovascular disease (CVD), which is the leading cause of death worldwide.^1^ Elevated LDL-C (low-density lipoprotein cholesterol) has been linked to higher CVD risk in randomized controlled trials, Mendelian randomization, and observational studies.^2^ Whereas HDL-C (high-density lipoprotein cholesterol) is associated with a lower CVD risk in observational studies only.^3,4^ Some studies suggest that LDL-C- and HDL-C-associated proteins, ApoB (apolipoprotein B) and ApoA1 (apolipoprotein A1), respectively,^5,6^ and lipid ratios^7,8^ may be even stronger predictors of CVD risk. Previous prospective studies suggest that diet may influence CVD risk through specific lipids^9–11^; however, further research is needed to clarify which dietary factors are most strongly associated with serum lipids.

Based on evidence from dietary substitution trials, most dietary guidelines for CVD prevention recommend reducing saturated fatty acids (SFA) by replacing them with polyunsaturated fatty acids (PUFA) or monounsaturated fatty acids.^9,12–14^ Substitution trials also suggest that replacing SFA with total carbohydrates has a beneficial effect on total cholesterol and LDL-C but may result in a concomitant increase in triglycerides and reduction in HDL-C.^9,12,15^ However, research on the replacement of SFA with different types of carbohydrates is sparse. There is also limited evidence for the association of total dietary protein with serum lipids,^16^ although some previous controlled trials have suggested substituting animal protein with plant protein may decrease LDL-C and increase HDL-C.^17^ Overall, the relationship between many dietary macronutrients and serum lipids, particularly apolipoproteins and lipid ratios, is not fully understood.^18,19^ Moreover, some prospective studies have suggested that SFA-rich foods may relate differently with CVD risk; for example, while several prospective studies have suggested a positive association between red meat and CVD risk, the association with dairy products is unclear. Therefore, understanding how macronutrients from different sources relate to serum lipids is important for CVD prevention.

Therefore, the aim of the present study was to investigate the associations between dietary macronutrients and macronutrients from different sources and serum lipids, using data from a large British cohort.

## Materials and Methods

The data that support the findings of this study are available from the UK Biobank study at http://ukbiobank.ac.uk/register-apply/.

### Subjects and Study Design

The UK Biobank is a population-based cohort study of middle-aged UK adults established between 2006 and 2010 to study risk factors for disease.^20,21^ Approximately 9.2 million individuals living within 25 miles of one of the 22 assessment centers in England, Wales, and Scotland were invited to participate. A total of 503 317 women and men aged 37 to 73 years at baseline were recruited (response rate =5.5%). Further details, including study protocol and data access permissions, are available online (http://www.ukbiobank.ac.uk/wp-content/uploads/2011/11/UK-Biobank-Protocol.pdf), and recruitment methods are described in detail elsewhere.^21^ All individuals provided informed consent to participate, and the study was approved by the National Information Governance Board for Health and Social care and the National Health Service North West Multicentre Research Ethics Committee (reference number 06/MRE08/65).

### Assessment of Macronutrient Intake

Participants who were recruited to the study between April 2009 and September 2010 completed a validated web-based 24-hour dietary assessment,^22,23^ the Oxford WebQ Questionnaire, at the assessment center at baseline (n=70 747). Moreover, those who provided a valid email address were invited via email to complete this dietary questionnaire on 4 further occasions during the follow-up period (follow-up 1: February 2011 to April 2011; follow-up 2: June 2011 to September 2011; follow-up 3: October 2011 to December 2011; and follow-up 4: April 2012 to June 2012; Figure I in the Data Supplement).

Intakes of 206 food items and 32 beverages were calculated from responses to the 24-hour dietary assessment, and macronutrient intakes were calculated from the UK Nutrient Databank Food Composition Tables (2013).^24^ Macronutrient intakes were expressed as a percentage of total energy intake. Carbohydrate sources included starch from whole grains (brown, seeded and whole meal bread, whole meal pasta and rice, bran cereal, biscuit cereal, oat cereal, and muesli), starch from refined grains (white and other bread, white pasta and rice, other cereals), free sugars (from added sugars or naturally occurring in honey, syrups, and fruit juices^15^), and nonfree sugars (total sugars minus free sugars). Intakes of fat and protein from plant sources and animal sources, including dairy and nondairy animal sources, were also measured (Data Supplement). Dietary intake of macronutrients was averaged from ≥2 twenty-four–hour dietary questionnaire responses (including baseline assessment) to provide an estimate of usual intake.^22,23^ The baseline 24-hour dietary assessment, that was completed at the assessment center, was obligatory for inclusion in this study to ensure that at least one dietary assessment was performed at the same time serum was collected.

### Cardiovascular Disease-Related Biomarkers

Nonfasting venous blood samples were collected at assessment centers on the same day the baseline 24-hour dietary assessment was completed (April 2009 to September 2010).^25^ Serum lipids measured included total cholesterol, LDL-C, HDL-C, triglycerides, ApoB and ApoA1. Serum lipids were used to calculate lipid ratios, including total cholesterol to HDL-C ratio, triglyceride to HDL-C ratio, and ApoB to ApoA1 ratio.^26^ Serum lipid profile refers to the overall pattern of total cholesterol, LDL-C, HDL-C, and triglycerides. Blood collection procedures are described in detail elsewhere^27^ and information on assay performance can be found on the UK Biobank website.^28^ A total of 20,239 participants (4.0%) had repeat blood samples taken between August 2012 and June 2013.

### Inclusion Criteria

Participants were excluded if they (1) withdrew consent, (2) reported they were taking lipid-lowering medication(s) at baseline, (3) were missing a baseline lipid measurement so that all included participants completed one dietary assessment at the same time as serum lipids were measured, or (4) were missing a valid mandatory baseline 24-hour dietary assessment plus at least one valid follow-up assessment (Figure II in the Data Supplement). Participants were also excluded if they did not meet the minimum requirements for a valid baseline and follow-up 24-hour dietary assessment, after 24-hour dietary assessments were removed if participants had extreme values for total energy intake (outside the range of 3347 kJ to 17 573 or 800 to 4200 kcal for men, outside the range of 2092 kJ to14 644 or 500 to 3500 kcal for women),^29^ or if participants reported they were ill or fasting on the respective day. After exclusions, a total of 24 639 participants contributed data to this study. Eligible participants completed between two and five 24-hour dietary assessments (including a mandatory baseline assessment) as follows: 2 (n=8113), 3 (n=6921), 4 (n=5953) and 5 (n=3652).

### Statistical Analysis

Macronutrient intakes as a percentage of energy intake were converted into quintiles, except fiber which was expressed in quintiles of grams per day. Correlations between serum lipids were calculated using Spearman correlation coefficients.

Several serum lipid measurements did not follow a normal distribution, and for consistency, all serum lipid variables were log-transformed to obtain geometric mean concentrations and relative differences of serum lipids with 95% CI. The geometric mean estimates were calculated based on predicted values from linear regressions of serum lipids against each macronutrient and fiber intake, with adjustment for age at recruitment (<45, 45–49, 50–54, 55–59, 60–64, and ≥65 years) and sex in the minimally adjusted model. Multivariable models were further adjusted for ethnicity (White, mixed race, Asian or Asian British, Black or Black British, other, and unknown), recruitment region (London, North-West England, North-Eastern England, Yorkshire and the Humber, West Midlands, East Midlands, South-West England, and Wales), Townsend deprivation index^30^ (quintiles from least to most affluent, unknown), smoking status (never, previous, <15 cigarettes/d, 15–29 cigarettes/d, ≥30 cigarettes/d, unknown), physical activity (low, medium, or high according to metabolic equivalent tasks in hours per week, unknown), alcohol (<1, 1–9, 10–19, 20–29, ≥30 g/d, unknown), body mass index (<20, 20–22.4, 22.5–24.9, 25–27.4, 27.5–29.9, 30–32.4, 32.5–34.9, 35–37.4, 37.5–39.9, or ≥40 in kg/m^2^, unknown), height (sex-specific groups in 5 cm increments, unknown), self-reported diabetes at baseline (yes, no, and unknown), and mean total daily energy intake (quintiles). Relative geometric means were then derived from the geometric means with the lowest category of macronutrient intake as the reference group. Tests for linear trend were performed using the percentage of total energy intake from macronutrients as continuous variables in the regression model. Macronutrient intakes were also modeled as continuous variables in increments of 5% higher energy to estimate the absolute difference in serum lipid per 5% higher energy from the macronutrient of interest. We modeled isoenergetic substitution of 5% of energy from SFA for other macronutrients using a multivariable nutrient density model, which included energy from all macronutrients except for SFA, as well as total energy.^31^ The regression coefficients from the model were interpreted as the effect of isoenergetic replacement of SFA for another macronutrient, while energy intake from all other macronutrients remained constant.

Sensitivity analyses were performed by restricting the sample to participants: (1) who completed ≥4 twenty-four–hour dietary assessments including mandatory baseline assessment, (2) with no self-reported change in diet in the prior 5 years, or (3) with biomarker concentrations within the range of (lower quartile − 3×interquartile range, upper quartile + 3×interquartile range) for the biomarker of interest. We also conducted a sensitivity analysis in participants (n=6215) who had all serum lipids measured at the reassessment visit (≈4 years after recruitment) and had ≥2 twenty-four–hour dietary assessments before the blood sample was taken (Figure III in the Data Supplement) and a sensitivity analyses using absolute macronutrient intakes expressed in grams per day as exposures.

Statistical tests were 2-sided, and the threshold for statistical significance was set at *P*<0.002 with Bonferroni correction for multiple comparisons (0.05/25 exposures).^32^ Most results were statistically significant due to the large sample size. Therefore, only the largest percent differences in serum lipids between the highest and lowest quintiles of macronutrient intake have been discussed in the text. STATA version 15.1 (StataCorp LP, College Station, TX) was used for data analyses, and R 3.5.2 (R Core Team, Vienna, Austria) was used to create the figures.

## Results

Table 1 displays participant characteristics according to lowest and highest quintile of carbohydrate, fat, and protein intake, expressed as percentages of total energy intake. Among participants who reported the highest intakes of carbohydrates, protein, or fat, there were a higher proportion of women and lower mean alcohol intakes. There was a higher proportion of never smokers and a lower total energy intake among participants who reported the highest carbohydrate intake. Participants who reported the highest fat intake had a higher total energy intake, whereas those who reported the highest protein intake had a lower total energy intake. There was a higher proportion of participants with a diabetes diagnosis and higher body mass index among participants with the lowest intakes of carbohydrate and highest intakes of protein. Mean grams and mean percentage of total energy intake within each quintile of macronutrient exposure are shown in Table I in the Data Supplement.

**Table 1.**
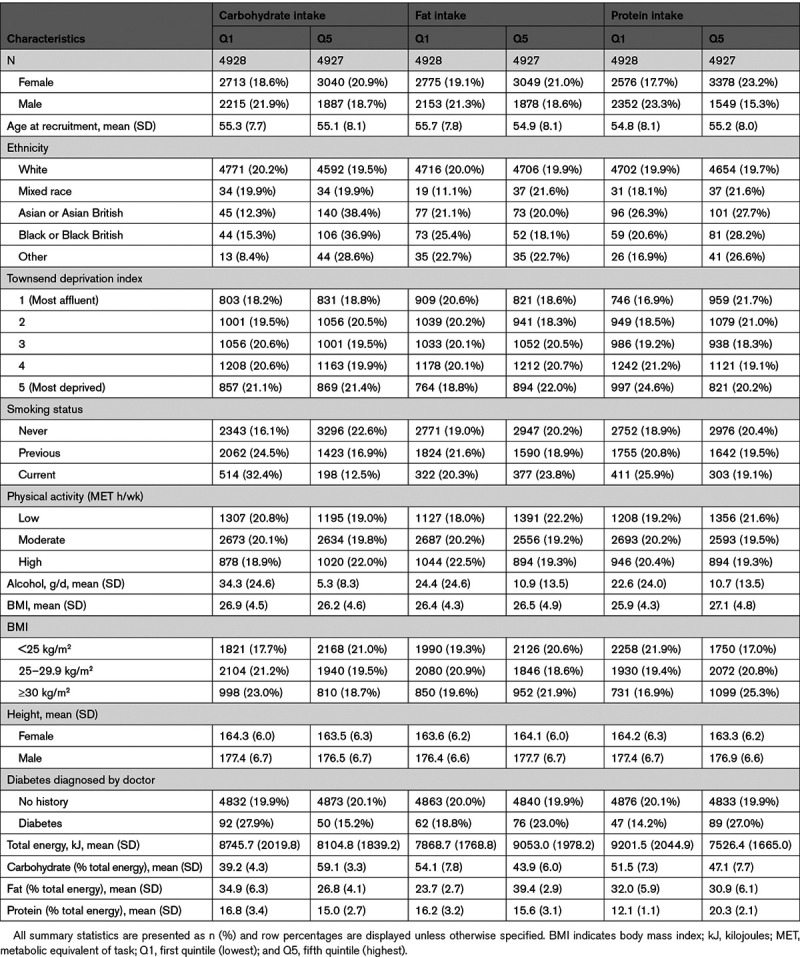
Baseline Characteristics Across Lowest and Highest Percentage Intakes of Carbohydrate, Fat, and Protein in 24 639 UK Biobank Participants

LDL-C was positively correlated with total cholesterol (*r*=0.94) and ApoB (*r*=0.96), whereas HDL-C was positively correlated with ApoA1 (*r*=0.93) and inversely correlated with triglycerides (*r*=−0.49; Figure IV in the Data Supplement).

### Macronutrient and Serum Lipid Associations

**Figure 1.**
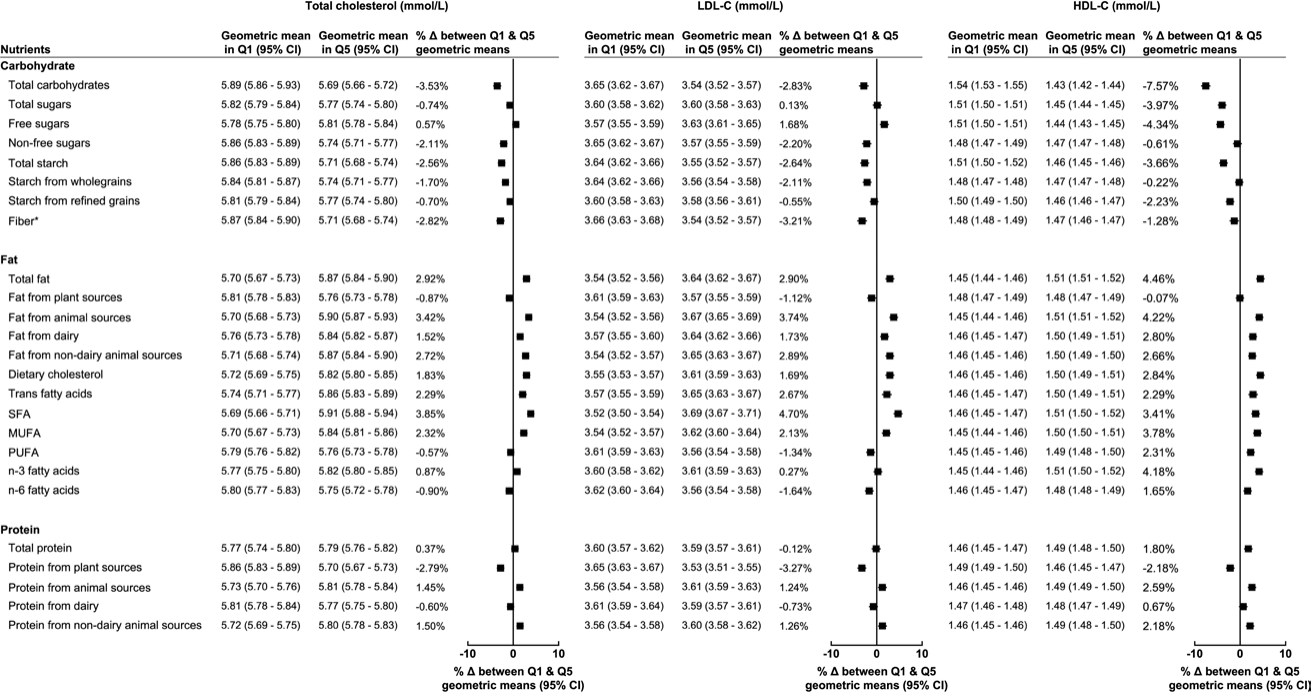
**Geometric mean of total cholesterol (mmol/L), LDL-C (low-density lipoprotein cholesterol; mmol/L), and HDL-C (high-density lipoprotein cholesterol; mmol/L) by lowest (Q1) and highest (Q5) percentage intake of macronutrients.** Models adjusted for age at recruitment, sex, ethnicity, region, Townsend deprivation index, smoking status, physical activity, alcohol, body mass index, height, diabetes diagnosed by a doctor, and mean daily energy intake in quintiles. Δ indicates difference; MUFA, monounsaturated fatty acids; n-3, omega-3; n-6, omega-6; PUFA, polyunsaturated fatty acids; and Q, quintile. *For fiber, Q1 and Q5 represent quintiles of total fiber intake (g/d).

**Figure 2.**
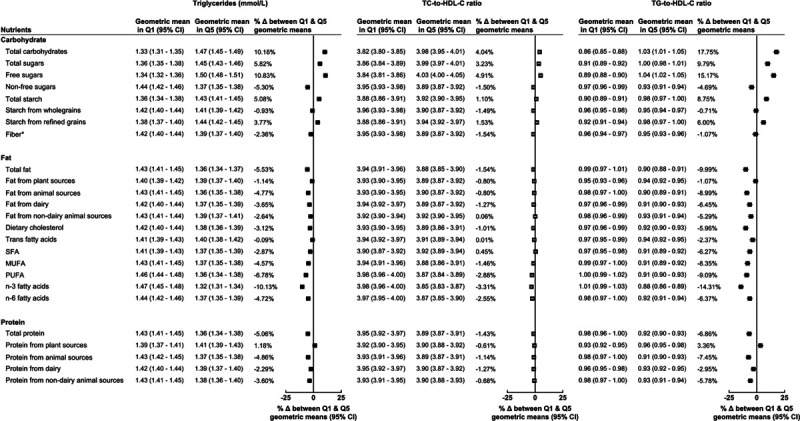
**Geometric mean of triglycerides (TG; mmol/L), total cholesterol (TC) to HDL-C (high-density lipoprotein cholesterol) ratio, and TG-to-HDL-C ratio by lowest (Q1) and highest (Q5) percentage intake of macronutrients.** Models adjusted for age at recruitment, sex, ethnicity, region, Townsend deprivation index, smoking status, physical activity, alcohol, body mass index, height, diabetes diagnosed by a doctor, and mean daily energy intake in quintiles. Δ indicates difference; PUFA, polyunsaturated fatty acids; Q, quintile; SFA, saturated fatty acids; TC, total cholesterol; and TG, triglycerides. *For fiber, Q1 and Q5 represent quintiles of total fiber intake (g/d).

For carbohydrates, the strongest positive associations were observed for both total carbohydrates and free sugars with triglycerides (Figure 2). Whereas the strongest inverse associations were observed for nonfree sugars with triglycerides and for both total carbohydrates and free sugars with HDL-C (Figure 1). Total carbohydrates and free sugars were also positively associated with total cholesterol to HDL-C ratio, while triglyceride to HDL ratio showed associations to similar those for triglycerides. Results minimally adjusted for age and sex are displayed in Figures V through VII in the Data Supplement, and results of tests for trend and participant numbers in each quintile are presented in Tables II through X in the Data Supplement.

When looking at the associations of dietary fat with serum lipids, the strongest associations were for SFA with LDL-C, and for omega-3 fatty acids with triglycerides, for which there were a positive and an inverse association, respectively (Figures 1 and 2). Monounsaturated fatty acid and omega-6 fatty acids were also inversely associated with triglycerides (Figure 2), while fat from animal sources was positively associated with HDL-C and inversely associated with triglycerides (Figure 1). Triglyceride to HDL-C-ratio had associations with dietary fats similar to those for triglycerides, and overall total cholesterol to HDL-C ratio had no associations with dietary fats.

Total protein and protein from animal sources were inversely associated with higher triglycerides and triglyceride to HDL-C ratio (Figure 2), while there were no strong associations between protein intake and other serum lipids (Figure 1).

**Figure 3.**
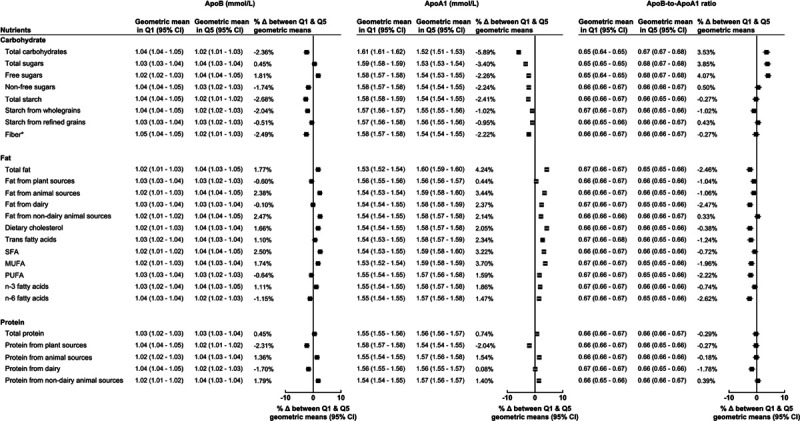
**Geometric mean of ApoB (apolipoprotein B; mmol/L), ApoA1 (apolipoprotein A1; mmol/L), and ApoB to ApoA1 ratio by lowest (Q1) and highest (Q5) percentage intake of macronutrients.** Models adjusted for age at recruitment, sex, ethnicity, region, Townsend deprivation index, smoking status, physical activity, alcohol, body mass index, height, diabetes diagnosed by a doctor, and mean daily energy intake in quintiles. Δ indicates difference; MUFA, monounsaturated fatty acids; n-3, omega-3; n-6, omega-6; PUFA, polyunsaturated fatty acids; Q, quintile; and SFA, saturated fatty acids. *For fiber, Q1 and Q5 represent quintiles of total fiber intake (g/d).

ApoB and ApoA1 had similar but weaker directions of association with macronutrients compared to LDL-C and HDL-C, respectively (Figure 3). Furthermore, we detected no sex-based differences in associations.

### Modeled Substitution Analyses

Modeled isoenergetic substitution of 5% energy from intake of SFA with intake from free sugars was associated with lower HDL-C and higher triglycerides (Table 2). Modeled substitution of intake of SFA with intake of starch from refined grains or whole grains was associated with similar but weaker associations as substitution with free sugars. While modeled substitution of intake of SFA with intake of monounsaturated fatty acids was not associated with a more favorable serum lipid profile, substitution of intake of SFA with intake of PUFA was associated with lower total cholesterol, LDL-C, triglycerides, and ApoB. Modeled substitution of intake of SFA with intake of protein did not show any strong associations with serum lipids. Absolute changes in serum lipids in mmol/L for isoenergetic substitutions of 5% energy from intake of SFA with other macronutrients are presented in Table XI in the Data Supplement.

**Table 2.**
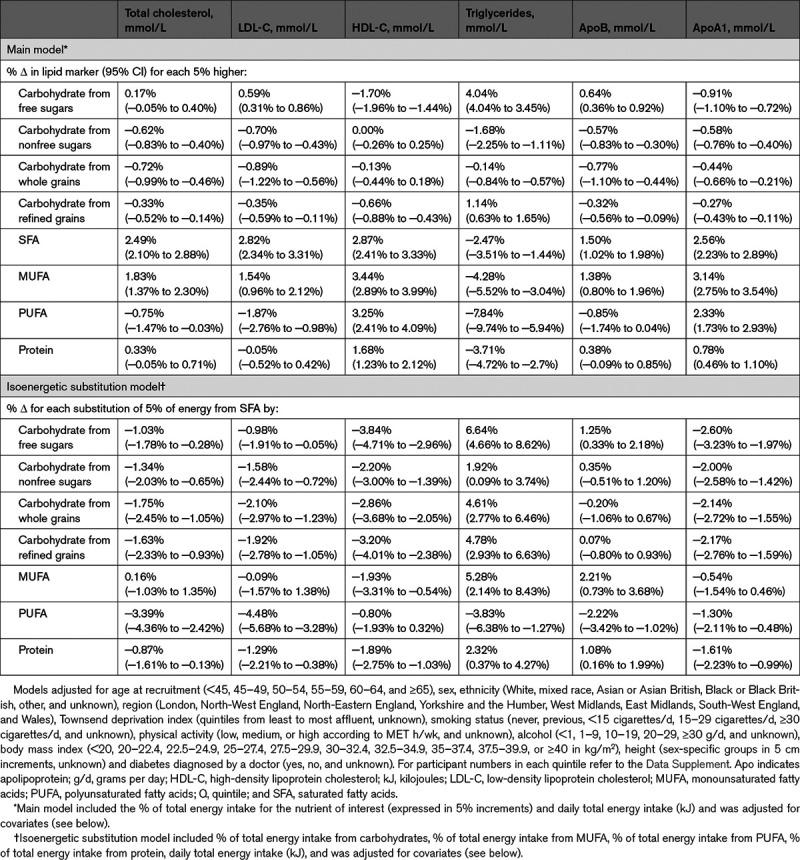
Percentage Change in Serum Lipids for Every 5% Higher Energy Intake From Macronutrients (Main Model) and Percentage Change in Serum Lipids for an Isoenergetic Substitution of 5% Energy From Saturated Fatty Acids With Other Macronutrients (Isoenergetic Substitution Model)

### Sensitivity Analyses

**Figure 4.**
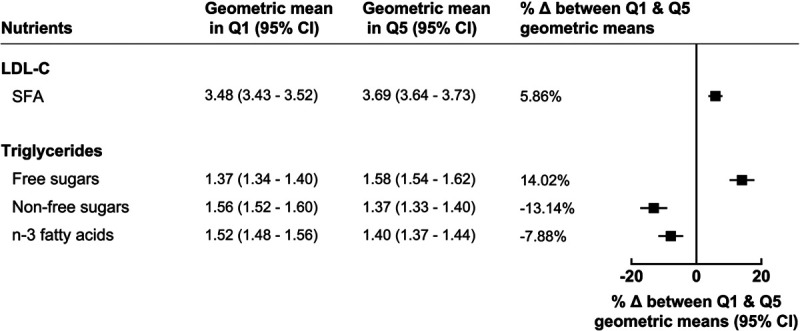
**Percentage difference in geometric mean concentrations of serum lipids between highest (Q5) and lowest (Q1) percentage intake of macronutrients restricting to participants with follow-up serum lipid measurements (n=6125).** Models adjusted for age at recruitment, sex, ethnicity, region, Townsend deprivation index, smoking status, physical activity, alcohol, body mass index, height, diabetes diagnosed by a doctor, and mean daily energy intake in quintiles. Δ indicates difference; LDL-C, low-density lipoprotein cholesterol; n-3, omega-3; Q, quintile; and SFA, saturated fatty acids.

Our findings remained similar after restricting the sample to participants (1) who completed ≥4 twenty-four–hour dietary assessments, (2) did not report change in diet in the prior 5 years, and (3) excluding outliers in biomarker concentrations, as well as in sensitivity analyses using absolute nutrient intakes as the exposure (Figures VIII through XIX in the Data Supplement). Findings were similar, or in some cases stronger, for most associations between macronutrient intake and follow-up serum lipid measurements (Figures SXX through SXII in the Data Supplement) when compared with our main analyses. For example, the percentage difference in geometric mean concentration (mmol/L) of LDL-C and triglycerides between highest and lowest SFA intake and free sugar intake, respectively, were 0.20 mmol/L (5.9%) and 0.19 mmol/L (14.0%) in this sensitivity analysis compared with 0.17 mmol/L (4.7%) and 0.15 mmol/L (10.8%) in our main analyses (Figure 4).

## Discussion

In this large British cohort, we observed that intakes of free sugars and nonfree sugars relate differently to serum lipids, with free sugars being related to higher triglycerides and lower HDL-C and nonfree sugars were associated with lower triglycerides. We also found that SFA is positively associated with LDL-C and that modeled substitution of intakes of SFA with intakes of PUFA is associated with a more favorable serum lipid profile. Our findings showed similar directions of associations for ApoB and ApoA1 to LDL-C and HDL-C, respectively.

The associations we report of free sugars with higher triglycerides and lower HDL-C are relatively novel; while previous cross-sectional studies have found that higher intake of total carbohydrates is associated with a less favorable overall serum lipid profile,^33,34^ these studies did not look separately at different types of carbohydrates. Previous randomized controlled trials have found that diets high in whole grains are associated with lower LDL-C and total cholesterol, compared with diets low in whole grains.^35^ Randomized controlled trials and modeled substitution studies have also observed that substitution of SFA with total carbohydrates is associated with decreased LDL-C but also increased triglycerides and decreased HDL-C, which aligns with our modeled substitution analysis replacing intake of SFA by intake of starch, particularly starch from refined grains.^33^ These findings suggest that it is important to consider carbohydrate subtype when assessing associations with serum lipids.

Our finding of a positive association between intake of SFA and LDL-C is in accordance with randomized controlled trials and some previous observational studies.^9–11,36^ This supports the US dietary recommendation to consume <10% of total energy from SFA,^37^ as the mean intake of SFA in the lowest consumers was 7.7% of total energy intake compared with 15.7% in the highest consumers. While dairy is an important source of dietary saturated fat in the UK biobank cohort,^38^ fat from dairy was only modestly positively associated with LDL-C, whereas fat from nondairy animal sources was more positively associated with LDL-C.

Our study found an inverse association between intake of PUFA and serum triglycerides, which has been found in previous observational studies.^39^ In our modeled isoenergetic substitution analyses, we found that substitution of intake of SFA with intake of PUFA was associated with lower LDL-C and triglycerides; previous clinical substitution trials have consistently demonstrated similar effects on LDL-C, but not on triglycerides.^9,14^ We observed that higher intakes of omega-3 fatty acids were associated with lower total cholesterol, LDL-C, triglycerides, and lipid ratios as compared to equivalent differences in intakes of omega-6 fatty acids, which is consistent with previous studies.^3,40,41^ Overall, our findings support dietary guidelines that recommend reducing intakes of SFA and replacing with intakes of PUFA rather than monounsaturated fatty acids or carbohydrates.^42–44^ However, the absolute reductions in lipids, particularly LDL-C, observed in this study are small when compared with those observed with clinical trials of dietary substitution and of statin therapy.^44,45^

We found no meaningful associations between protein intake and serum lipids, excepting the inverse associations between total protein and protein from animal sources with triglycerides and triglyceride to HDL-C ratio. Our findings are similar to those found in the OmniHeart trial,^46^ but not to the PURE study (Prospective Urban Rural Epidemiology).^33^

This study has several strengths. Our study had a large sample size and detailed dietary information, which allowed us to look at macronutrients from different sources. The validation study for this questionnaire also showed that correlations of dietary estimates with recovery biomarkers for total sugars and protein improved significantly with 2 dietary assessments when compared with one dietary assessment, which is unlikely to capture usual intake.^23^ Moreover, biomarker measurements collected 4 years after recruitment were available for some of the participants, and this made it possible for us to conduct sensitivity analyses examining associations with follow-up serum lipid measurements, which supported the primary results from our main analyses.

There are some limitations to the current study. Although we minimized random error by calculating macronutrient intakes using at least two 24-hour dietary measurements, and sensitivity analyses restricted to ≥4 twenty-four–hour dietary assessments showed similar results, the 24-hour dietary assessment is a self-reported measure and, therefore, our findings may still be prone to measurement error, particularly for total energy intakes,^23^ and regression dilution bias.^47–49^ Reverse causality is possible, although sensitivity analysis looking at associations with follow-up biomarkers supported our main findings. As with every observational study, residual confounding may operate, and we were also unable to adjust for some potential covariates, such as family history of dyslipidemia, which may be a source of confounding. The UK Biobank has predominantly healthy participants of White ethnicity, and our sample who completed ≥2 twenty-four–hour dietary assessments may differ slightly from those who did not within this cohort, and therefore, we cannot exclude selection bias. However, previous studies have shown that large nonrepresentative samples can be used to obtain generalizable risk factor-disease associations.^20,25,50,51^

The present study found novel diverging associations between intakes of free sugars and nonfree sugars and serum lipids, with free sugars being related to a less favorable lipid profile, which suggests that carbohydrate quality could play an important role in CVD risk reduction. Our findings also support known associations between SFA and LDL-C and between modeled substitution of intakes of SFA with intakes of PUFA and overall serum lipid profile. Further research is necessary to determine if the observed differences in serum lipids with sources of macronutrient intake, particularly carbohydrates, correspond to differences in cardiovascular risk.

## Acknowledgments

This research has been conducted using the UK Biobank Resource under application number 24494. We thank all participants, researchers, and support staff who make the study possible. Bona fide researchers can apply to use the UK Biobank data set by registering and applying at http://ukbiobank.ac.uk/register-apply/.

## Sources of Funding

Dr Kelly is supported by a Clarendon Fund Medical Sciences Scholarship (Oxford, United Kingdom) and a MIGA doctors in training grant (Adelaide, Australia). C.Z. Watling is supported by the Nuffield Department of Population Health Doctor of Philosophy student scholarship (Oxford, United Kingdom). T.Y.N. Tong is supported by the UK Medical Research Council (MR/M012190/1; Swindon, United Kingdom). C. Piernas is supported by the National Institute for Health Research (NIHR) Applied Research Collaboration (ARC; Oxford, United Kingdom). J.L. Carter is supported by core grants to Clinical Trial Service Unit (CTSU) from the Medical Research Council (Clinical Trial Service Unit A310; Swindon, United Kingdom) and the British Heart Foundation (CH/1996001/9454; London, United Kingdom), and by the NIHR Oxford Biomedical Research Centre (Oxford, United Kingdom). K. Papier is supported by the Wellcome Trust, Our Planet Our Health (Livestock, Environment, and People [LEAP]) grant number (205212/Z/16/Z; London, United Kingdom). A. Perez-Cornago is supported by a Cancer Research UK Population Research Fellowship (C60192/A28516; London, United Kingdom) and by the World Cancer Research Fund (WCRF UK; London, United Kingdom), as part of the Word Cancer Research Fund International grant programme (2019/1953).

## Disclosures

None.

## Supplementary Material


