# Supplementary material for: Associations Between Macronutrients From Different Dietary Sources and Serum Lipids in 24 639 UK Biobank Study Participants
Source: Arterioscler Thromb Vasc Biol. 2021 May 27;41(7):2190–200. doi: 10.1161/ATVBAHA.120.315628 (PMC8216602; doi:10.1161/ATVBAHA.120.315628)
Supplement: Supplementary file 1 [file atv-41-2190-s001.pdf]

SUPPLEMENTAL MATERIALS

Associations between macronutrients from different dietary sources and serum lipids in 24,639 UK Biobank study participants

Rebecca K Kelly[1], MBBS, MPH, Cody Z Watling[1], MSc, Tammy Y N Tong[1], MPhil, PhD, Carmen Piernas[2], MSc, PhD, Jennifer L Carter[3], MRes, PhD, Keren Papier[1], PhD, Timothy J Key[1] MSc, DPhil, Aurora Perez-Cornago[1] MPH, MSc, PhD

[1] Cancer Epidemiology Unit, Nuffield Department of Population Health, University of Oxford

[2] Nuffield Department of Primary Care Health Sciences, University of Oxford, Oxford

[3] Clinical Trial Service Unit and Epidemiological Studies Unit, Nuffield Department of Population Health, University of Oxford, Oxford

SUPPLEMENTAL METHODS...5
Measurement of macronutrient intakes and total energy using the Oxford WebQ Questionnaire...5
Measurement of serum lipids...6
Measurement of demographic variables using the Touchscreen questionnaire......7
Expanded statistical methods...9
SUPPLEMENTAL FIGURES AND FIGURE LEGENDS..10
Supplemental Figure I. Dietary assessments in sub-sample of UK biobank participants included in our main analyses (n=24,639). 10
Supplemental Figure II. Flow chart of UK biobank participants included in the current study after exclusion criteria were applied. 11
Supplemental Figure III. Dietary assessment in sub-sample of UK biobank participants included in a sensitivity analysis restricting to participants with follow-up serum lipid measurement(s) (n=6,215). 12
Supplemental Figure IV. Spearman's correlation between lipid markers collected at baseline in 24,639 participants in the UK Biobank study. 13
Supplemental Figure V. Minimally adjusted geometric mean of total cholesterol (mmol/L), LDL-C (mmol/L) and HDL-C (mmol/L) by lowest (Q1) and highest (Q5) percentage intake of macronutrients. 14
Supplemental Figure VI. **Minimally adjusted** geometric mean of triglycerides (mmol/L), TC-to-HDL-C ratio and TG-to-HDL-C ratio by lowest (Q1) and highest (Q5) percentage intake of macronutrients. 16
Supplemental Figure VII. **Minimally adjusted** geometric mean ApoB (mmol/L), ApoA1 (mmol/L) and ApoB-to-ApoA1 ratio by lowest (Q1) and highest (Q5) percentage intake of macronutrients. 18
Supplemental Figure VIII. Geometric mean of total cholesterol (mmol/L), LDL-C (mmol/L) and HDL-C (mmol/L) by lowest (Q1) and highest (Q5) percentage intake of macronutrients **restricting to participants with ≥ 4 24-hour dietary assessments (including mandatory baseline assessment)** (n=9,605).........20
Supplemental Figure IX. Geometric mean of triglycerides (mmol/L), TC-to-HDL-C ratio and TG-to-HDL-C ratio by lowest (Q1) and highest (Q5) percentage intake of macronutrients restricting to participants with **≥ 4 24-hour dietary assessments** (including mandatory baseline assessment) (n=9,605). 22
Supplemental Figure X. Geometric mean of ApoB (mmol/L), ApoA1 (mmol/L) and ApoB-to-ApoA1 ratio by lowest (Q1) and highest (Q5) percentage intake of macronutrients **restricting to participants with ≥ 4 24-hour dietary assessments (including mandatory baseline assessment)** (n=9,605).........24
Supplemental Figure XI. Geometric mean of total cholesterol (mmol/L), LDL-C (mmol/L) and HDL-C (mmol/L) by lowest (Q1) and highest (Q5) percentage intake of macronutrients **excluding participants with a self-reported change in diet in the prior five years** (n=16,072). 26
Supplemental Figure XII. Geometric mean of triglycerides (mmol/L), TC-to-HDL-C ratio and TG-to-HDL-C ratio by lowest (Q1) and highest (Q5) percentage

intake of macronutrients **excluding participants with a self-reported change in diet in the prior five years** (n=16,072). 28

Supplemental Figure XIII. Geometric mean of ApoB (mmol/L), ApoA1 (mmol/L) and ApoB-to-ApoA1 ratio by lowest (Q1) and highest (Q5) percentage intake of macronutrients **excluding participants with a self-reported change in diet in the prior five years** (n=16,072). 30

Supplemental Figure XIV. Geometric mean of total cholesterol (mmol/L), LDL-C (mmol/L) and HDL-C (mmol/L) by lowest (Q1) and highest (Q5) percentage intake of macronutrients **excluding participants with extreme levels of lipid markers** (n=24,408). 32

Supplemental Figure XV. Geometric mean of triglycerides (mmol/L), TC-to-HDL-C ratio and TG-to-HDL-C ratio by lowest (Q1) and highest (Q5) percentage intake of macronutrients **excluding participants with extreme levels of lipid markers** (n=24,408). 34

Supplemental Figure XVI. Geometric mean of ApoB (mmol/L), ApoA1 (mmol/L) and ApoB-to-ApoA1 ratio by lowest (Q1) and highest (Q5) percentage intake of macronutrients **excluding participants with extreme levels of lipid markers** (n=24,408). 36

Supplemental Figure XVII. Geometric mean of total cholesterol (mmol/L), LDL-C (mmol/L) and HDL-C (mmol/L) by lowest (Q1) and highest (Q5) intake of **macronutrients in grams per day** (n=24,639). 38

Supplemental Figure XVIII. Geometric mean of triglycerides (mmol/L), TC-to-HDL-C ratio and TG-to-HDL-C ratio by lowest (Q1) and highest (Q5) intake of **macronutrients in grams per day** (n=24,639). 40

Supplemental Figure XIX. Geometric mean of ApoB (mmol/L), ApoA1 (mmol/L) and ApoB-to-ApoA1 ratio by lowest (Q1) and highest (Q5) intake of **macronutrients in grams per day** (n=24,639). 42

Supplemental Figure XX. Geometric mean of total cholesterol (mmol/L), LDL-C (mmol/L) and HDL-C (mmol/L) by lowest (Q1) and highest (Q5) percentage intake of macronutrients **restricting to participants with follow-up serum lipid measurements** (n=6,215). 44

Supplemental Figure XXI. Geometric mean of triglycerides (mmol/L), TC-to-HDL-C ratio and TG-to-HDL-C ratio by lowest (Q1) and highest (Q5) percentage intake of macronutrients in a **restricting to participants with follow-up serum lipid measurements** (n=6,215). 46

Supplemental Figure XXII. Geometric mean of ApoB (mmol/L), ApoA1 (mmol/L) and ApoB-to-ApoA1 ratio by lowest (Q1) and highest (Q5) percentage intake of macronutrients **restricting to participants with follow-up serum lipid measurements** (n=6,215). 48

SUPPLEMENTAL TABLES AND SUPPORTING INFORMATION 50

Supplemental Table I. Daily macronutrient intake in quintiles by daily macronutrient intake in grams and percentage of total energy intake. 50

Supplemental Table II. Geometric mean concentrations of total cholesterol (mmol/L) by percentage intake of macronutrients. 52

Supplemental Table III. Geometric mean concentrations of LDL-C (mmol/L) by percentage intake of macronutrients. 54

Supplemental Table IV. Geometric mean concentrations of HDL-C (mmol/L) by percentage intake of macronutrients. 56

Supplemental Table V. Geometric mean concentrations of triglycerides (mmol/L) by percentage intake of macronutrients. 58

Supplemental Table VI. Geometric mean concentrations of TC-to-HDL-C ratio by percentage intake of macronutrients. 60

Supplemental Table VII. Geometric mean concentrations of TG-to-HDL-C ratio by percentage intake of macronutrients. 62

Supplemental Table VIII. Geometric mean concentrations of ApoB (mmol/L) by percentage intake of macronutrients. 64

Supplemental Table IX. Geometric mean concentrations of ApoA1 (mmol/L) by percentage intake of macronutrients. 66

Supplemental Table X. Geometric mean concentrations of ApoB-to-ApoA1 ratio by percentage intake of macronutrients. 68

Supplemental Table XI. Percentage change in serum lipids for every 5% of energy intake increase in macronutrients (main model) and percentage change in serum lipids for a 5% of energy intake isoenergetic replacement of saturated fatty acids with other macronutrients (isoenergetic substitution model). 70

Supplemental Table XII. Sample aliquot number for lipid marker measurements at baseline in 24,639 UK Biobank participants. 72

Supplemental Table XIII. List of lipid-modifying drug names and codes obtained from baseline touchscreen questionnaire used to exclude participants taking lipid modifying medication (n=84,008). 73

MAJOR RESOURCES TABLE 74

REFERENCES 75

SUPPLEMENTAL METHODS

Measurement of macronutrient intakes and total energy using the Oxford WebQ Questionnaire

The Oxford WebQ Questionnaire (WebQ) is an internet based 24-hour dietary assessment designed to measure diet on repeated occasions in large cohort studies. Similar to a 24-hour dietary recall, this questionnaire aims to measure the type and quantity of food and beverages consumed by a participant in the last 24 hours. However, unlike a 24-hour dietary recall, responses are limited to the 206 foods and 32 beverages presented. Further, this dietary assessment is on average quicker to complete than a traditional 24-hour dietary recall and is completed on the internet.1

The WebQ has been validated for macronutrients and total energy intake using recovery biomarkers[2] and compared with interviewer-administered 24-hour dietary recall.[1] The reproducibility of macronutrient intakes measured using two WebQ was similar to intake measured using a single food frequency questionnaire.[3] Further, assessment of WebQ response rates has demonstrated that the WebQ is acceptable to the public and therefore feasible for repeated administrations in large cohort studies.[4]

Participant responses to the questions “Would you say that what you ate and drank yesterday was fairly typical for you?” and “Reason for not eating or drinking normally”. If participants answered that they were “ill” or “fasting” then the WebQ assessment completed on this day was excluded from these analyses.

Sources of carbohydrates

Starch from whole grains was calculated as the sum of mixed (50/50) brown & seeded bread, wholemeal bread, wholemeal pasta & rice, bran cereal, biscuit cereal, oat cereal (with sugar), oat cereal (non-sugar) and muesli. Starch from refined grains was calculated as the sum of starch from other bread, white bread, white pasta & rice, and other cereal (with sugar).

Sources of fat

Fat from animal sources was calculated as the sum of fat contained in animal derived food items, including meats, poultry, eggs, dairy products, and fish. Fat from dairy sources was calculated as the sum of fat from whole milk (>3.6 g fat per 100g), semi-skimmed milk (>1g fat per 100g), skimmed milk (including cholesterol lowering), full fat yoghurt, low fat yoghurt, high fat cheese (>17.5g fat per 100g), medium and low fat cheese (<17.5g fat per 100g), cream, animal fat spread lower fat and animal fat spread normal. Animal fat from non-dairy sources was calculated by subtracting animal fat from dairy from total animal fat. Fat from plant sources was calculated as the sum of fat contained in plant derived food items, such as legumes, pulses, vegetables, fruits and, nuts and seeds.

Sources of protein

Protein from plant and animal sources were calculated using the same methods used for fat from plant and animal sources.

Alcohol

Alcohol in grams was calculated using WebQ intake and classed as <1g/day, 1-9 g/day, 10-19 g/day, 20-29 g/day or $\geq$30 g/day.

Total energy

Total daily energy intake was estimated as the sum of kilojoules (kJ) consumed from each food item and beverage over a 24-hour period.

Measurement of serum lipids

Serum lipids were measured in samples collected at baseline assessment from all participants, and at follow-up assessment for a sub-sample, according to the UK Biobank sample handling and storage protocol, which is detailed elsewhere.[5,6] Non-fasting blood samples (45ml) were collected by a trained phlebotomist at assessment centres using vacutainers specific to the analysis to be performed. For cholesterol this was collected in a serum separator tube (STT) to later obtain serum fractions. These samples were stored in a refrigerator between 2°C to 8°C and then transported to a central processing laboratory and stored at -80°C within an average time of 24 hours until they were analyzed. Serum samples were centrifuged for 10 minutes at 2000 RCF prior to analyses using automated analyzers. Internal Quality Control (IQC) samples at low, medium and high levels were run with each batch of participant samples and then used to validate participant results if they were within the opening and closing results of IQC for that batch. External validation was performed through an external quality assurance (EQA) scheme.[7] The UK Biobank adjusted biomarker data prior to release.

Total cholesterol, LDL-C, HDL-C and ApoB were measured directly using homogenous assays.[7] Direct homogenous assays do not require ultracentrifugation or precipitation.[8] Total cholesterol was measured by CHO-POD analysis. LDL-C was measured by enzymatic protective selection analysis. HDL was measured by enzyme immunoinhibition analysis. Triglycerides were measured by GPO-POD analysis. ApoB and ApoA1 were measured by immunoturbidimetric analysis. All analyses were performed on a Beckman Coulter AU5800.

Lipid ratios were calculated using the following formulae:

- $TC - to - HDL - C\ ratio = \frac{TC\ (mmol/L)}{HDL{-}C\ (mmol/L)}$,
- $TG - to - HDL - C\ ratio = \frac{TG\ (mmol/L)}{HDL{-}C\ (mmol/L)}$, *and*
- $ApoA1 - to - ApoB\ ratio = \frac{ApoA1\ (mmol/L)}{ApoB\ (mmol/L)}$.

Measurement of demographic variables using the Touchscreen questionnaire

Age at recruitment

Calculated using month and year of birth, obtained from central registry and verified or updated by participants. All participants were nominally assigned 15 as the day of birth to calculate age at recruitment as <45 years, 45-49 years, 50-54 years, 55-59 years, 60-64 years or $\geq$65 years.

Region of recruitment

Region of recruitment was coded based on the assessment center for each participant and included; London, North-West England, North-Eastern England, Yorkshire & the Humber, West Midlands, East Midlands, South-West England and Wales. Specifically, assessments centers were assigned the following region: London (assessment centers: St Bartholomew's Hospital, Hounslow, Croydon) North-West England (assessment centers: Stockport, Manchester, Liverpool, Bury), North-East England (assessment centers: Newcastle, Middlesbrough), Yorkshire (assessment centers: Leeds, Sheffield), West Midlands (assessment centers: Stoke, Birmingham) East Midlands (assessment center: Nottingham), South-East England (assessment centers: Oxford, Reading), South-West England (assessment center: Bristol) and Wales (assessment centers: Swansea, Wrexham, Cardiff).

Ethnicity

Participant responses to the questions "What is your ethnicity?" and "What is your ethnic background?" were used to classify participants as white (British, Irish, any other white background), mixed (white and black Caribbean, white and black African, white and Asian, any other mixed background), Asian or Asian British (Indian, Pakistani, Bangladeshi, any other Asian background), African or African British (Caribbean, African, any other Black background), Other (Other ethnic group) or missing/unknown/prefer not to say (PNTS).

Townsend deprivation index

Townsend deprivation index was used as an indicator of socioeconomic status and was calculated by assigning each participant a score based on the output area corresponding to their post-code and linking this to national census output area. This

was converted to quintiles for use in our analysis, with most-affluent (quintile 1) to most-deprived (quintile 5). An additional category for missing/unknown/ PNTS was included.

Smoking

Self-reported smoking status was based on UK biobank variable *n_20116_0_0.* Participants were classified as never smokers, ex-smokers, light smokers (<15 cigarettes per day), medium smokers (15-29 cigarettes per day), heaving smokers (≥30 cigarettes per day) or missing/unknown/PNTS. Quantity of cigarettes smoked per day was based on the question "About how many cigarettes do you smoke on average each day?".

Physical activity

Participant's answers to questions about daily walking, moderate physical activity and vigorous physical activity were used to estimate excess metabolic equivalents (METs) per week.[9] Excess METs refer to energy expenditure greater than that of an inactive person. Participant's excess METs per week was used to categorize physical activity as low (<10 excess METs/week), medium (10-49.99 excess METs/week), high (≥50 excess METs/week) or missing/unknown/PNTS.

Anthropometric measures

All anthropometric measurements were made by trained research staff. Participant standing height was measured using the SECA 240 height measure, without shoes and to the closest centimeter (cm). Height was categorized in 5 cm increments for men as follows: <160cm, 160-164.9, 165-169.9, 170-174.9, 175-179.9, 180-184.9, 185-189.9, ≥190 cm. Height was categorized in 5 cm increments for women as follows: <150cm, 150-154.9, 155-159.9, 160-164.9, 165-169.9, 170-174.9, 175-179.9, ≥180 cm. Weight was measured using standard scales without shoes and outdoor clothing. However, if participants underwent bioimpedance analysis using the Tanita BC 418 body composition analyzer this value was used for weight instead. Body Mass Index (BMI) was calculated using weight (kg) and height (cm) measurements, as described above, using the formula: weight (kg)/height(m^2). BMI was categorized in 2.5-unit increments as follows; <20, 20-22.4, 22.5-24.9, 25-27.4, 27.5-29.9, 30-32.4, 32.5-34.9, 35-37.4, 37.5-39.9, ≥40 kg/m^2.

Diabetes

Participant response to the question "Has a doctor ever told you that you have diabetes?" was used to classify participants with diabetes diagnosis, no history of diagnosis or missing/unknown/PNTS.

Change in diet

Participant response to the question “Have you made any major changes to your diet in the last 5 years?” was used to create the dichotomous variable for change in diet over the last five years.

Lipid-modifying drug use

Lipid-lowering medication use was coded using self-reported medication use at baseline. A list of lipid-modifying drug(s) prescribed in the UK was obtained from the National Institute for Clinical Excellence.[10] The UK biobank ‘Treatment/medication code’ variable (n_20003_0) and was hand-searched for individual lipid-modifying drug names and drug Brand names found using the National Health Services “Medicines A to Z” handbook (Supplemental Table XIII).[11]

Expanded statistical methods

In sensitivity analyses, we created a derived variable for each lipid marker to identify participants with extreme measurements, using the following formula: [Lower quartile – 3*Interquartile range] and [Upper quartile + 3*Interquartile range]. Those beyond this range were identified as having extreme values and excluded in a sensitivity analysis. This method was chosen to account for the approximately non-normal distribution of some biomarkers.

SUPPLEMENTAL FIGURES AND FIGURE LEGENDS

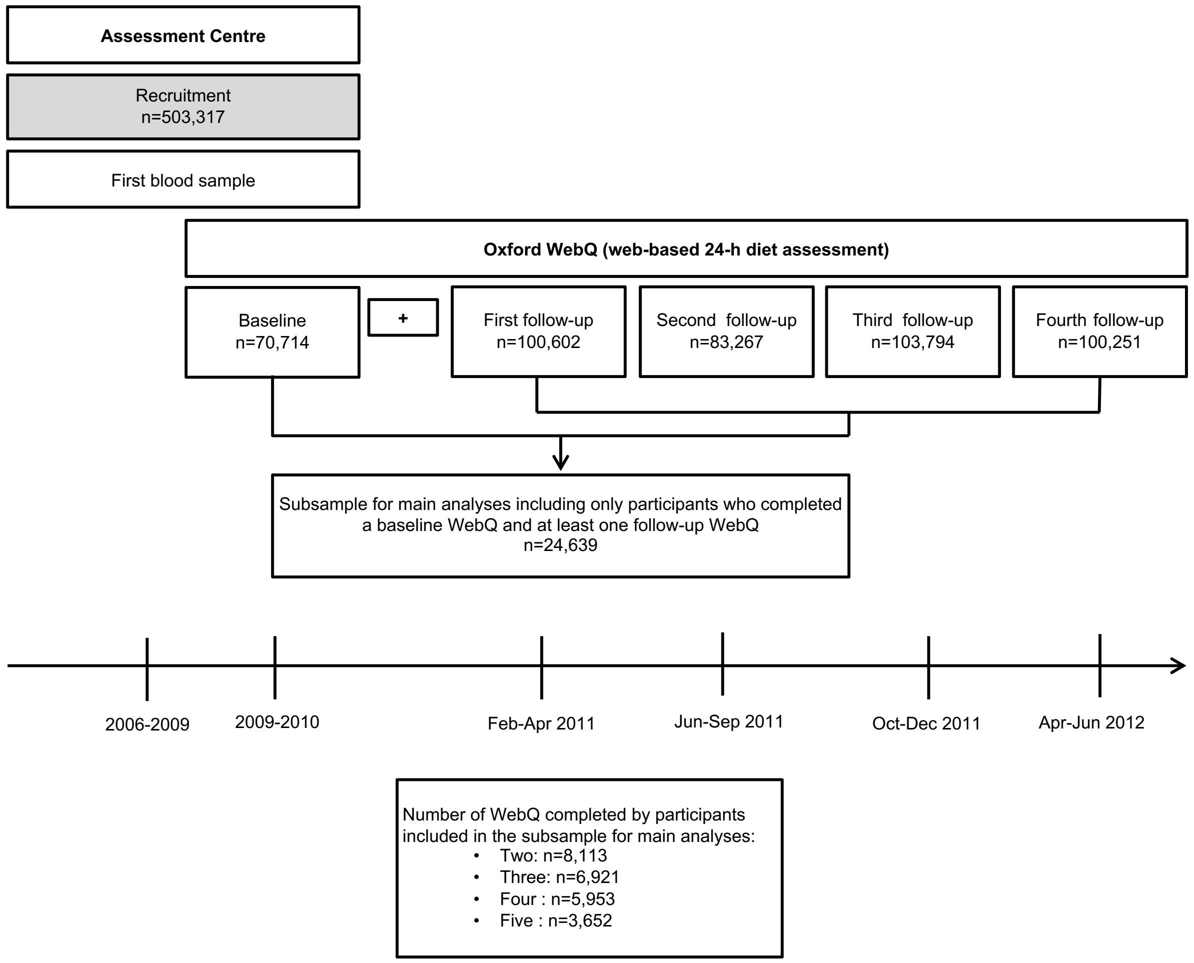

Supplemental Figure I. Dietary assessments in sub-sample of UK biobank participants included in our main analyses (n=24,639).

Final numbers for 24-hour dietary assessment (WebQ) completion excluded participants who withdrew consent, reported they were taking lipid-lowering medication(s) at baseline, were missing all baseline lipid measurements, or were missing a mandatory baseline 24-hour dietary assessment plus at least one follow-up assessment. Participants were also excluded if they did not meet the minimum requirements for a valid baseline 24-hour dietary assessment and valid follow-up 24-hour dietary assessment after assessments were excluded if participants reported extreme values for total energy intake or if participants reported they were ill or fasting on the respective day.

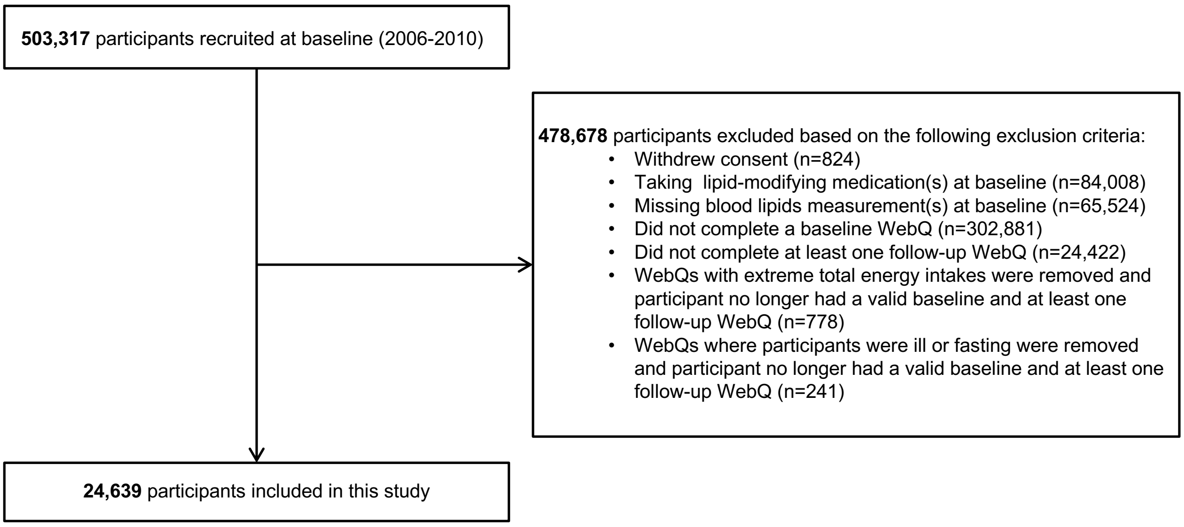

Supplemental Figure II. Flow chart of UK biobank participants included in the current study after exclusion criteria were applied.

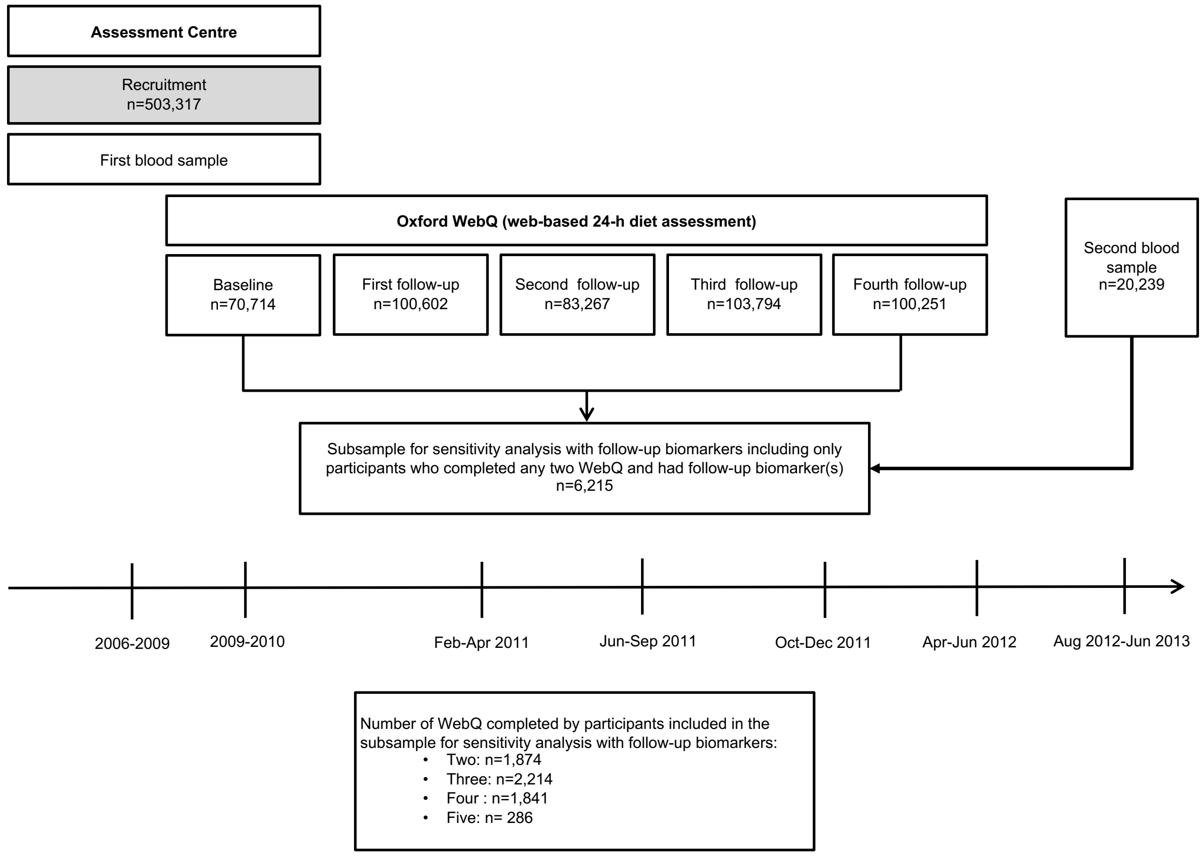

Supplemental Figure III. Dietary assessment in sub-sample of UK biobank participants included in a sensitivity analysis restricting to participants with follow-up serum lipid measurement(s) (n=6,215).

Final numbers for 24-hour dietary assessment (WebQ) completion excluded participants who withdrew consent, reported they were taking lipid-lowering medication(s) at baseline, were missing all follow-up lipid measurements, or who did not have at least two 24-hour dietary assessments. Participants were also excluded if they did not meet the minimum requirements for at least two 24-hour dietary assessments after assessments were excluded if participants reported extreme values for total energy intake or if participants reported they were ill or fasting on the respective day.

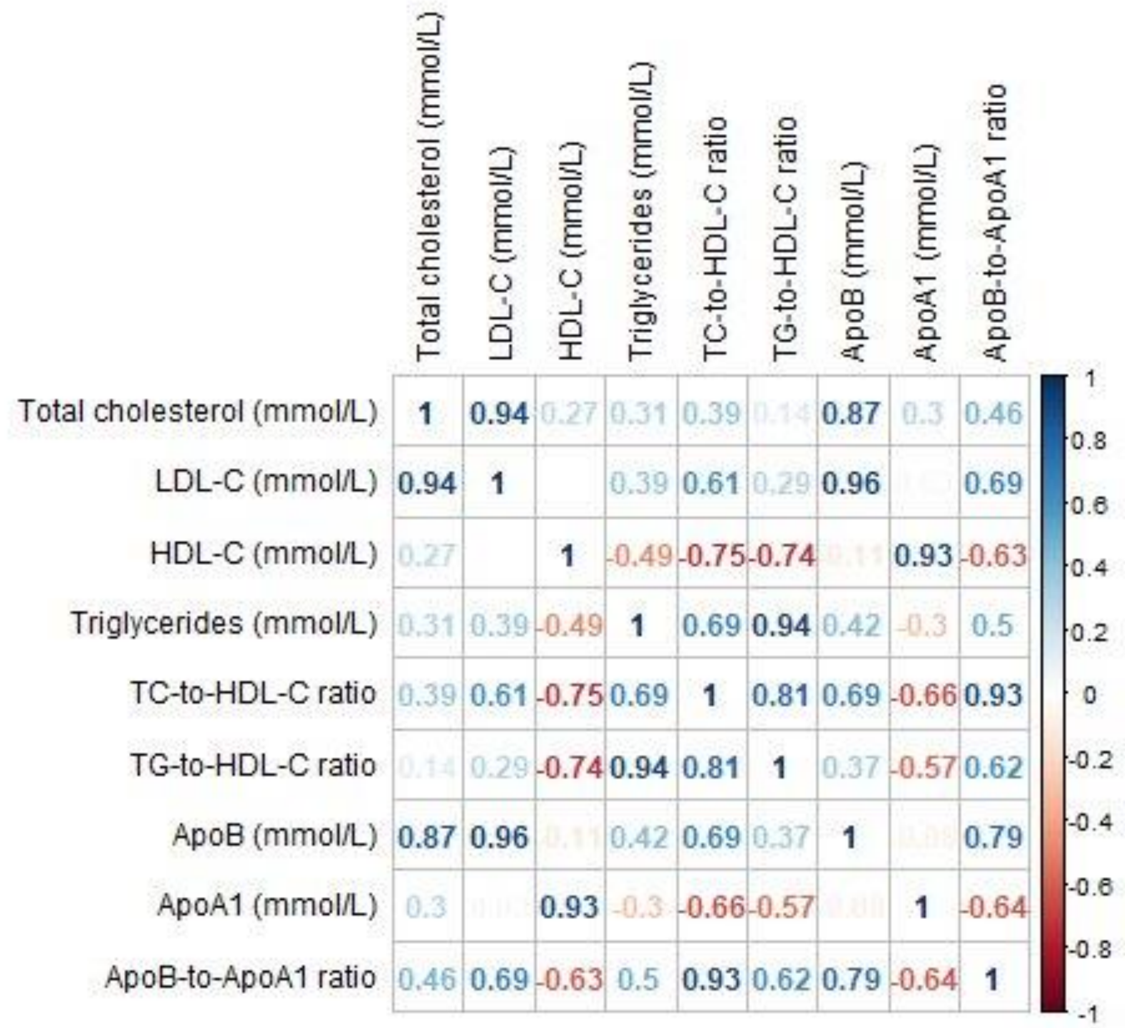

Supplemental Figure IV. Spearman's correlation between lipid markers collected at baseline in 24,639 participants in the UK Biobank study.

Abbreviations: Apo = apolipoprotein; HDL-C = high-density lipoprotein cholesterol; LDL-C= low-density lipoprotein cholesterol; TC = total cholesterol; TG = triglycerides

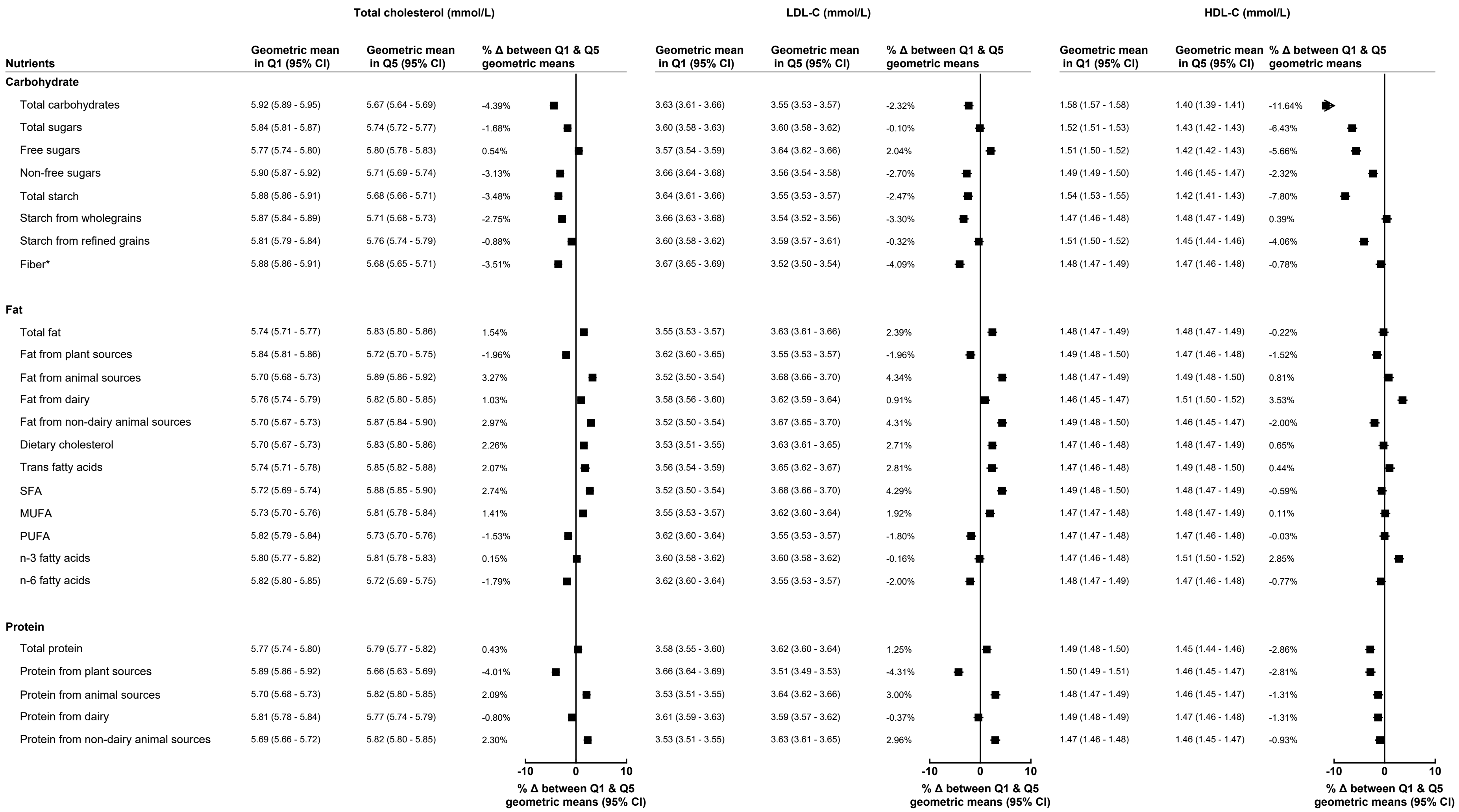

Nutrients	Total cholesterol (mmol/L) Geometric mean in Q1 (95% CI)	Geometric mean in Q5 (95% CI)	% Δ between Q1 & Q5 geometric means	LDL-C (mmol/L) Geometric mean in Q1 (95% CI)	Geometric mean in Q5 (95% CI)	% Δ between Q1 & Q5 geometric means	HDL-C (mmol/L) Geometric mean in Q1 (95% CI)	Geometric mean in Q5 (95% CI)	% Δ between Q1 & Q5 geometric means
**Carbohydrate**									
Total carbohydrates	5.92 (5.89 - 5.95)	5.67 (5.64 - 5.69)	-4.39%	3.63 (3.61 - 3.66)	3.55 (3.53 - 3.57)	-2.32%	1.58 (1.57 - 1.58)	1.40 (1.39 - 1.41)	-11.64%
Total sugars	5.84 (5.81 - 5.87)	5.74 (5.72 - 5.77)	-1.68%	3.60 (3.58 - 3.63)	3.60 (3.58 - 3.62)	-0.10%	1.52 (1.51 - 1.53)	1.43 (1.42 - 1.43)	-6.43%
Free sugars	5.77 (5.74 - 5.80)	5.80 (5.78 - 5.83)	0.54%	3.57 (3.54 - 3.59)	3.64 (3.62 - 3.66)	2.04%	1.51 (1.50 - 1.52)	1.42 (1.42 - 1.43)	-5.66%
Non-free sugars	5.90 (5.87 - 5.92)	5.71 (5.69 - 5.74)	-3.13%	3.66 (3.64 - 3.68)	3.56 (3.54 - 3.58)	-2.70%	1.49 (1.49 - 1.50)	1.46 (1.45 - 1.47)	-2.32%
Total starch	5.88 (5.86 - 5.91)	5.68 (5.66 - 5.71)	-3.48%	3.64 (3.61 - 3.66)	3.55 (3.53 - 3.57)	-2.47%	1.54 (1.53 - 1.55)	1.42 (1.41 - 1.43)	-7.80%
Starch from wholegrains	5.87 (5.84 - 5.89)	5.71 (5.68 - 5.73)	-2.75%	3.66 (3.63 - 3.68)	3.54 (3.52 - 3.56)	-3.30%	1.47 (1.46 - 1.48)	1.48 (1.47 - 1.49)	0.39%
Starch from refined grains	5.81 (5.79 - 5.84)	5.76 (5.74 - 5.79)	-0.88%	3.60 (3.58 - 3.62)	3.59 (3.57 - 3.61)	-0.32%	1.51 (1.50 - 1.52)	1.45 (1.44 - 1.46)	-4.06%
Fiber*	5.88 (5.86 - 5.91)	5.68 (5.65 - 5.71)	-3.51%	3.67 (3.65 - 3.69)	3.52 (3.50 - 3.54)	-4.09%	1.48 (1.47 - 1.49)	1.47 (1.46 - 1.48)	-0.78%
**Fat**									
Total fat	5.74 (5.71 - 5.77)	5.83 (5.80 - 5.86)	1.54%	3.55 (3.53 - 3.57)	3.63 (3.61 - 3.66)	2.39%	1.48 (1.47 - 1.49)	1.48 (1.47 - 1.49)	-0.22%
Fat from plant sources	5.84 (5.81 - 5.86)	5.72 (5.70 - 5.75)	-1.96%	3.62 (3.60 - 3.65)	3.55 (3.53 - 3.57)	-1.96%	1.49 (1.48 - 1.50)	1.47 (1.46 - 1.48)	-1.52%
Fat from animal sources	5.70 (5.68 - 5.73)	5.89 (5.86 - 5.92)	3.27%	3.52 (3.50 - 3.54)	3.68 (3.66 - 3.70)	4.34%	1.48 (1.47 - 1.49)	1.49 (1.48 - 1.50)	0.81%
Fat from dairy	5.76 (5.74 - 5.79)	5.82 (5.80 - 5.85)	1.03%	3.58 (3.56 - 3.60)	3.62 (3.59 - 3.64)	0.91%	1.46 (1.45 - 1.47)	1.51 (1.50 - 1.52)	3.53%
Fat from non-dairy animal sources	5.70 (5.67 - 5.73)	5.87 (5.84 - 5.90)	2.97%	3.52 (3.50 - 3.54)	3.67 (3.65 - 3.70)	4.31%	1.49 (1.48 - 1.50)	1.46 (1.45 - 1.47)	-2.00%
Dietary cholesterol	5.70 (5.67 - 5.73)	5.83 (5.80 - 5.86)	2.26%	3.53 (3.51 - 3.55)	3.63 (3.61 - 3.65)	2.71%	1.47 (1.46 - 1.48)	1.48 (1.47 - 1.49)	0.65%
Trans fatty acids	5.74 (5.71 - 5.78)	5.85 (5.82 - 5.88)	2.07%	3.56 (3.54 - 3.59)	3.65 (3.62 - 3.67)	2.81%	1.47 (1.46 - 1.48)	1.49 (1.48 - 1.50)	0.44%
SFA	5.72 (5.69 - 5.74)	5.88 (5.85 - 5.90)	2.74%	3.52 (3.50 - 3.54)	3.68 (3.66 - 3.70)	4.29%	1.49 (1.48 - 1.50)	1.48 (1.47 - 1.49)	-0.59%
MUFA	5.73 (5.70 - 5.76)	5.81 (5.78 - 5.84)	1.41%	3.55 (3.53 - 3.57)	3.62 (3.60 - 3.64)	1.92%	1.47 (1.47 - 1.48)	1.48 (1.47 - 1.49)	0.11%
PUFA	5.82 (5.79 - 5.84)	5.73 (5.70 - 5.76)	-1.53%	3.62 (3.60 - 3.64)	3.55 (3.53 - 3.57)	-1.80%	1.47 (1.47 - 1.48)	1.47 (1.46 - 1.48)	-0.03%
n-3 fatty acids	5.80 (5.77 - 5.82)	5.81 (5.78 - 5.83)	0.15%	3.60 (3.58 - 3.62)	3.60 (3.58 - 3.62)	-0.16%	1.47 (1.46 - 1.48)	1.51 (1.50 - 1.52)	2.85%
n-6 fatty acids	5.82 (5.80 - 5.85)	5.72 (5.69 - 5.75)	-1.79%	3.62 (3.60 - 3.64)	3.55 (3.53 - 3.57)	-2.00%	1.48 (1.47 - 1.49)	1.47 (1.46 - 1.48)	-0.77%
**Protein**									
Total protein	5.77 (5.74 - 5.80)	5.79 (5.77 - 5.82)	0.43%	3.58 (3.55 - 3.60)	3.62 (3.60 - 3.64)	1.25%	1.49 (1.48 - 1.50)	1.45 (1.44 - 1.46)	-2.86%
Protein from plant sources	5.89 (5.86 - 5.92)	5.66 (5.63 - 5.69)	-4.01%	3.66 (3.64 - 3.69)	3.51 (3.49 - 3.53)	-4.31%	1.50 (1.49 - 1.51)	1.46 (1.45 - 1.47)	-2.81%
Protein from animal sources	5.70 (5.68 - 5.73)	5.82 (5.80 - 5.85)	2.09%	3.53 (3.51 - 3.55)	3.64 (3.62 - 3.66)	3.00%	1.48 (1.47 - 1.49)	1.46 (1.45 - 1.47)	-1.31%
Protein from dairy	5.81 (5.78 - 5.84)	5.77 (5.74 - 5.79)	-0.80%	3.61 (3.59 - 3.63)	3.59 (3.57 - 3.62)	-0.37%	1.49 (1.48 - 1.49)	1.47 (1.46 - 1.48)	-1.31%
Protein from non-dairy animal sources	5.69 (5.66 - 5.72)	5.82 (5.80 - 5.85)	2.30%	3.53 (3.51 - 3.55)	3.63 (3.61 - 3.65)	2.96%	1.47 (1.46 - 1.48)	1.46 (1.45 - 1.47)	-0.93%

Supplemental Figure V. Minimally adjusted geometric mean of total cholesterol (mmol/L), LDL-C (mmol/L) and HDL-C (mmol/L) by lowest (Q1) and highest (Q5) percentage intake of macronutrients.

Models adjusted for age at recruitment (<45, 45-49, 50-54, 55-59, 60-64, ≥65) and sex.

*For fiber, Q1 and Q5 represent quintiles of total fiber intake (g/day).

Abbreviations: CI = confidence interval; g/day = grams per day; HDL-C = high-density lipoprotein cholesterol; LDL-C= low-density lipoprotein cholesterol; MUFA = monounsaturated fatty acids; n-3 = omega-3; n-6 = omega-6; PUFA = polyunsaturated fatty acids; Q = quintile; SFA = saturated fatty acids.

Nutrients	Triglycerides (mmol/L)			TC-to-HDL-C ratio			TG-to-HDL-C ratio		
	Geometric mean in Q1 (95% CI)	Geometric mean in Q5 (95% CI)	% Δ between Q1 & Q5 geometric means	Geometric mean in Q1 (95% CI)	Geometric mean in Q5 (95% CI)	% Δ between Q1 & Q5 geometric means	Geometric mean in Q1 (95% CI)	Geometric mean in Q5 (95% CI)	% Δ between Q1 & Q5 geometric means
**Carbohydrate**									
Total carbohydrates	1.34 (1.32 - 1.36)	1.46 (1.44 - 1.48)	8.19%	3.76 (3.73 - 3.78)	4.04 (4.01 - 4.07)	7.25%	0.85 (0.84 - 0.87)	1.04 (1.02 - 1.06)	19.84%
Total sugars	1.38 (1.36 - 1.40)	1.44 (1.42 - 1.46)	4.27%	3.84 (3.82 - 3.87)	4.03 (4.00 - 4.06)	4.75%	0.91 (0.89 - 0.92)	1.01 (0.99 - 1.03)	10.70%
Free sugars	1.34 (1.32 - 1.36)	1.51 (1.49 - 1.53)	12.10%	3.83 (3.80 - 3.86)	4.07 (4.05 - 4.10)	6.20%	0.89 (0.87 - 0.90)	1.06 (1.04 - 1.08)	17.76%
Non-free sugars	1.46 (1.44 - 1.48)	1.35 (1.33 - 1.37)	-8.13%	3.94 (3.92 - 3.97)	3.91 (3.89 - 3.94)	-0.81%	0.98 (0.96 - 1.00)	0.92 (0.91 - 0.94)	-5.81%
Total starch	1.35 (1.33 - 1.37)	1.44 (1.42 - 1.46)	6.57%	3.82 (3.80 - 3.85)	3.99 (3.97 - 4.02)	4.32%	0.88 (0.86 - 0.89)	1.01 (1.00 - 1.03)	14.37%
Starch from wholegrains	1.45 (1.43 - 1.47)	1.36 (1.34 - 1.38)	-6.35%	3.98 (3.96 - 4.01)	3.86 (3.83 - 3.89)	-3.14%	0.98 (0.97 - 1.00)	0.92 (0.90 - 0.94)	-6.73%
Starch from refined grains	1.37 (1.35 - 1.39)	1.45 (1.43 - 1.47)	6.06%	3.86 (3.83 - 3.89)	3.98 (3.96 - 4.01)	3.18%	0.91 (0.89 - 0.92)	1.00 (0.99 - 1.02)	10.11%
Fiber*	1.43 (1.41 - 1.45)	1.37 (1.35 - 1.39)	-4.28%	3.97 (3.95 - 4.00)	3.87 (3.84 - 3.89)	-2.73%	0.97 (0.95 - 0.98)	0.93 (0.92 - 0.95)	-3.50%
**Fat**									
Total fat	1.40 (1.38 - 1.42)	1.39 (1.37 - 1.41)	-0.81%	3.87 (3.85 - 3.90)	3.94 (3.91 - 3.97)	1.76%	0.94 (0.93 - 0.96)	0.94 (0.92 - 0.95)	-0.60%
Fat from plant sources	1.41 (1.39 - 1.43)	1.38 (1.36 - 1.40)	-2.13%	3.91 (3.89 - 3.94)	3.89 (3.87 - 3.92)	-0.44%	0.94 (0.93 - 0.96)	0.94 (0.92 - 0.95)	-0.61%
Fat from animal sources	1.38 (1.37 - 1.40)	1.40 (1.38 - 1.42)	0.99%	3.86 (3.84 - 3.89)	3.96 (3.93 - 3.98)	2.46%	0.94 (0.92 - 0.95)	0.94 (0.92 - 0.95)	0.18%
Fat from dairy	1.42 (1.41 - 1.44)	1.35 (1.33 - 1.37)	-5.30%	3.96 (3.93 - 3.98)	3.86 (3.83 - 3.89)	-2.50%	0.98 (0.96 - 1.00)	0.90 (0.88 - 0.91)	-8.83%
Fat from non-dairy animal sources	1.37 (1.35 - 1.39)	1.45 (1.43 - 1.46)	5.59%	3.83 (3.80 - 3.86)	4.02 (4.00 - 4.05)	4.97%	0.92 (0.90 - 0.93)	0.99 (0.97 - 1.01)	7.59%
Dietary cholesterol	1.39 (1.37 - 1.41)	1.41 (1.39 - 1.43)	1.59%	3.88 (3.86 - 3.91)	3.95 (3.92 - 3.97)	1.62%	0.95 (0.93 - 0.96)	0.95 (0.94 - 0.97)	0.94%
Trans fatty acids	1.39 (1.37 - 1.41)	1.42 (1.39 - 1.44)	2.02%	3.90 (3.87 - 3.93)	3.93 (3.90 - 3.96)	0.83%	0.94 (0.92 - 0.96)	0.95 (0.93 - 0.97)	1.06%
SFA	1.37 (1.36 - 1.39)	1.40 (1.38 - 1.41)	1.57%	3.84 (3.81 - 3.86)	3.97 (3.94 - 3.99)	3.33%	0.92 (0.91 - 0.94)	0.94 (0.93 - 0.96)	2.16%
MUFA	1.40 (1.38 - 1.42)	1.39 (1.38 - 1.41)	-0.32%	3.89 (3.86 - 3.91)	3.94 (3.91 - 3.96)	1.30%	0.95 (0.93 - 0.96)	0.94 (0.93 - 0.96)	-0.43%
PUFA	1.45 (1.43 - 1.47)	1.36 (1.34 - 1.38)	-6.16%	3.95 (3.92 - 3.97)	3.89 (3.86 - 3.91)	-1.51%	0.98 (0.96 - 1.00)	0.92 (0.91 - 0.94)	-6.14%
n-3 fatty acids	1.46 (1.44 - 1.48)	1.31 (1.29 - 1.33)	-10.63%	3.95 (3.92 - 3.98)	3.84 (3.82 - 3.87)	-2.70%	0.99 (0.98 - 1.01)	0.87 (0.85 - 0.88)	-13.48%
n-6 fatty acids	1.42 (1.41 - 1.44)	1.37 (1.35 - 1.39)	-3.77%	3.93 (3.91 - 3.96)	3.89 (3.87 - 3.92)	-1.02%	0.96 (0.95 - 0.98)	0.93 (0.92 - 0.95)	-3.00%
**Protein**									
Total protein	1.40 (1.38 - 1.42)	1.41 (1.39 - 1.43)	0.46%	3.87 (3.84 - 3.90)	4.00 (3.97 - 4.03)	3.29%	0.94 (0.92 - 0.96)	0.97 (0.96 - 0.99)	3.32%
Protein from plant sources	1.42 (1.40 - 1.44)	1.37 (1.35 - 1.39)	-3.42%	3.93 (3.90 - 3.96)	3.88 (3.86 - 3.91)	-1.20%	0.95 (0.93 - 0.96)	0.94 (0.93 - 0.96)	-0.61%
Protein from animal sources	1.38 (1.37 - 1.40)	1.41 (1.39 - 1.43)	2.03%	3.85 (3.83 - 3.88)	3.99 (3.96 - 4.01)	3.40%	0.94 (0.92 - 0.95)	0.97 (0.95 - 0.98)	3.34%
Protein from dairy	1.40 (1.39 - 1.42)	1.39 (1.37 - 1.41)	-1.04%	3.91 (3.89 - 3.94)	3.93 (3.91 - 3.96)	0.51%	0.95 (0.93 - 0.96)	0.95 (0.93 - 0.96)	0.28%
Protein from non-dairy animal sources	1.39 (1.37 - 1.40)	1.43 (1.41 - 1.45)	2.96%	3.86 (3.84 - 3.89)	3.99 (3.96 - 4.02)	3.23%	0.94 (0.92 - 0.96)	0.98 (0.96 - 0.99)	3.89%

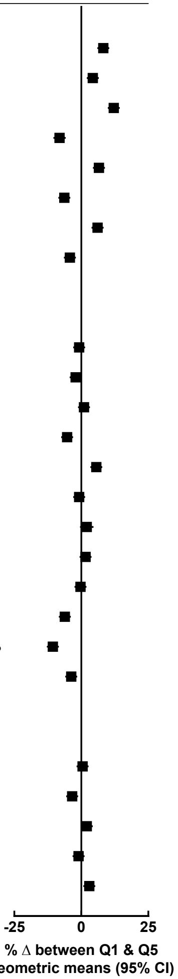

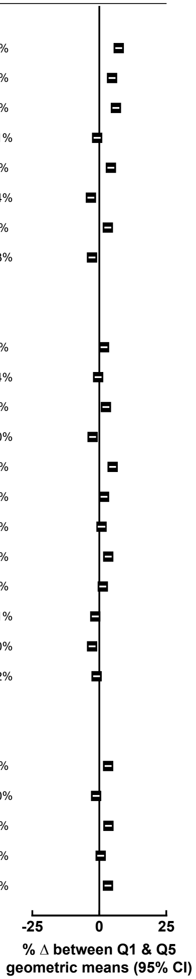

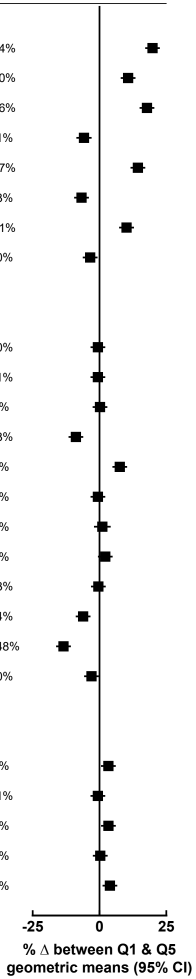

Supplemental Figure VI. **Minimally adjusted** geometric mean of triglycerides (mmol/L), TC-to-HDL-C ratio and TG-to-HDL-C ratio by lowest (Q1) and highest (Q5) percentage intake of macronutrients.

Models adjusted for age at recruitment (<45, 45-49, 50-54, 55-59, 60-64, >65) and sex.

*For fiber Q1 and Q5 represent quintiles of total fiber intake (g/day).

Abbreviations: CI = confidence interval; g/day = grams per day; HDL-C = high-density lipoprotein cholesterol; MUFA = monounsaturated fatty acids; n-3 = omega-3; n-6 = omega-6; PUFA = polyunsaturated fatty acids; Q = quintile; SFA = saturated fatty acids; TC = total cholesterol; TG = triglycerides.

ApoB (mmol/L)

Nutrients	Geometric mean in Q1 (95% CI)	Geometric mean in Q5 (95% CI)	% Δ between Q1 & Q5 geometric means
**Carbohydrate**			
Total carbohydrates	1.04 (1.04 - 1.05)	1.02 (1.01 - 1.03)	-2.26%
Total sugars	1.04 (1.03 - 1.04)	1.03 (1.03 - 1.04)	-0.11%
Free sugars	1.02 (1.02 - 1.03)	1.04 (1.04 - 1.05)	2.07%
Non-free sugars	1.05 (1.04 - 1.06)	1.02 (1.02 - 1.03)	-2.63%
Total starch	1.04 (1.04 - 1.05)	1.02 (1.01 - 1.02)	-2.62%
Starch from wholegrains	1.05 (1.04 - 1.06)	1.01 (1.01 - 1.02)	-3.63%
Starch from refined grains	1.03 (1.02 - 1.04)	1.03 (1.02 - 1.04)	-0.15%
Fiber*	1.06 (1.05 - 1.06)	1.01 (1.01 - 1.02)	-4.25%
**Fat**			
Total fat	1.02 (1.02 - 1.03)	1.04 (1.03 - 1.04)	1.26%
Fat from plant sources	1.04 (1.03 - 1.05)	1.02 (1.02 - 1.03)	-1.75%
Fat from animal sources	1.02 (1.01 - 1.02)	1.05 (1.04 - 1.05)	3.12%
Fat from dairy	1.04 (1.03 - 1.04)	1.03 (1.02 - 1.03)	-1.11%
Fat from non-dairy animal sources	1.01 (1.00 - 1.02)	1.05 (1.05 - 1.06)	4.17%
Dietary cholesterol	1.01 (1.01 - 1.02)	1.04 (1.04 - 1.05)	2.95%
Trans fatty acids	1.03 (1.02 - 1.04)	1.04 (1.03 - 1.04)	0.71%
SFA	1.02 (1.01 - 1.03)	1.04 (1.04 - 1.05)	2.18%
MUFA	1.02 (1.02 - 1.03)	1.04 (1.03 - 1.04)	1.53%
PUFA	1.04 (1.03 - 1.04)	1.02 (1.02 - 1.03)	-1.32%
n-3 fatty acids	1.03 (1.03 - 1.04)	1.04 (1.03 - 1.04)	0.54%
n-6 fatty acids	1.04 (1.03 - 1.04)	1.02 (1.01 - 1.03)	-1.72%
**Protein**			
Total protein	1.02 (1.02 - 1.03)	1.04 (1.04 - 1.05)	2.09%
Protein from plant sources	1.05 (1.04 - 1.05)	1.01 (1.00 - 1.02)	-3.75%
Protein from animal sources	1.01 (1.01 - 1.02)	1.05 (1.04 - 1.05)	3.52%
Protein from dairy	1.04 (1.03 - 1.05)	1.03 (1.02 - 1.03)	-1.41%
Protein from non-dairy animal sources	1.01 (1.00 - 1.01)	1.05 (1.04 - 1.05)	3.95%

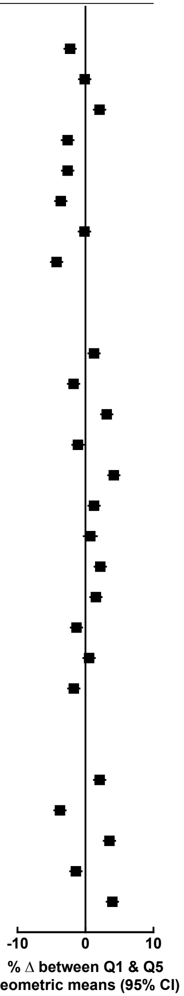

ApoA1 (mmol/L)

Nutrients	Geometric mean in Q1 (95% CI)	Geometric mean in Q5 (95% CI)	% Δ between Q1 & Q5 geometric means
**Carbohydrate**			
Total carbohydrates	1.64 (1.63 - 1.65)	1.50 (1.49 - 1.50)	-9.29%
Total sugars	1.60 (1.59 - 1.61)	1.51 (1.51 - 1.52)	-5.58%
Free sugars	1.58 (1.57 - 1.58)	1.53 (1.52 - 1.53)	-3.20%
Non-free sugars	1.59 (1.58 - 1.60)	1.53 (1.52 - 1.54)	-3.92%
Total starch	1.61 (1.60 - 1.61)	1.52 (1.51 - 1.52)	-5.67%
Starch from wholegrains	1.57 (1.56 - 1.58)	1.55 (1.54 - 1.56)	-1.20%
Starch from refined grains	1.58 (1.57 - 1.58)	1.54 (1.54 - 1.55)	-2.16%
Fiber*	1.57 (1.57 - 1.58)	1.55 (1.54 - 1.55)	-1.65%
**Fat**			
Total fat	1.56 (1.55 - 1.56)	1.57 (1.56 - 1.58)	0.94%
Fat from plant sources	1.57 (1.56 - 1.58)	1.55 (1.55 - 1.56)	-0.89%
Fat from animal sources	1.55 (1.55 - 1.56)	1.57 (1.57 - 1.58)	1.41%
Fat from dairy	1.54 (1.54 - 1.55)	1.59 (1.58 - 1.59)	2.82%
Fat from non-dairy animal sources	1.56 (1.56 - 1.57)	1.55 (1.55 - 1.56)	-0.63%
Dietary cholesterol	1.55 (1.54 - 1.56)	1.56 (1.56 - 1.57)	0.86%
Trans fatty acids	1.55 (1.54 - 1.56)	1.57 (1.57 - 1.58)	1.64%
SFA	1.56 (1.55 - 1.57)	1.57 (1.56 - 1.58)	0.53%
MUFA	1.55 (1.54 - 1.56)	1.57 (1.56 - 1.58)	1.23%
PUFA	1.56 (1.55 - 1.57)	1.56 (1.55 - 1.56)	-0.25%
n-3 fatty acids	1.56 (1.55 - 1.57)	1.57 (1.57 - 1.58)	0.74%
n-6 fatty acids	1.56 (1.56 - 1.57)	1.56 (1.55 - 1.56)	-0.41%
**Protein**			
Total protein	1.57 (1.57 - 1.58)	1.53 (1.53 - 1.54)	-2.44%
Protein from plant sources	1.58 (1.58 - 1.59)	1.54 (1.53 - 1.54)	-3.12%
Protein from animal sources	1.56 (1.55 - 1.57)	1.55 (1.54 - 1.55)	-0.84%
Protein from dairy	1.57 (1.56 - 1.58)	1.55 (1.54 - 1.55)	-1.45%
Protein from non-dairy animal sources	1.55 (1.55 - 1.56)	1.55 (1.54 - 1.56)	-0.35%

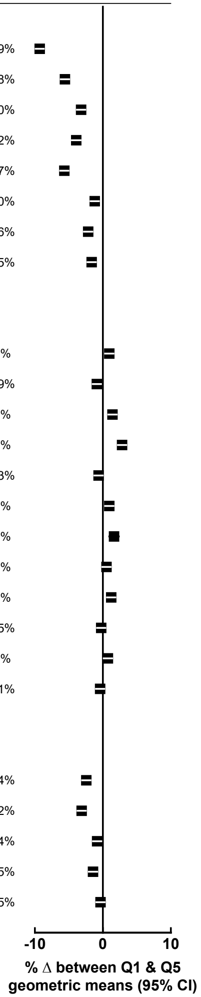

ApoB-to-ApoA1 ratio

Nutrients	Geometric mean in Q1 (95% CI)	Geometric mean in Q5 (95% CI)	% Δ between Q1 & Q5 geometric means
**Carbohydrate**			
Total carbohydrates	0.64 (0.63 - 0.64)	0.68 (0.68 - 0.69)	7.03%
Total sugars	0.65 (0.64 - 0.65)	0.68 (0.68 - 0.69)	5.47%
Free sugars	0.65 (0.64 - 0.65)	0.68 (0.68 - 0.69)	5.27%
Non-free sugars	0.66 (0.65 - 0.67)	0.67 (0.66 - 0.67)	1.29%
Total starch	0.65 (0.65 - 0.66)	0.67 (0.67 - 0.68)	3.05%
Starch from wholegrains	0.67 (0.66 - 0.67)	0.65 (0.65 - 0.66)	-2.43%
Starch from refined grains	0.65 (0.65 - 0.66)	0.67 (0.66 - 0.67)	2.01%
Fiber*	0.67 (0.67 - 0.68)	0.65 (0.65 - 0.66)	-2.60%
**Fat**			
Total fat	0.66 (0.65 - 0.66)	0.66 (0.66 - 0.67)	0.32%
Fat from plant sources	0.66 (0.66 - 0.67)	0.66 (0.65 - 0.66)	-0.86%
Fat from animal sources	0.65 (0.65 - 0.66)	0.67 (0.66 - 0.67)	1.72%
Fat from dairy	0.67 (0.67 - 0.68)	0.65 (0.64 - 0.65)	-3.93%
Fat from non-dairy animal sources	0.65 (0.64 - 0.65)	0.68 (0.67 - 0.68)	4.80%
Dietary cholesterol	0.65 (0.65 - 0.66)	0.67 (0.66 - 0.67)	2.08%
Trans fatty acids	0.66 (0.66 - 0.67)	0.66 (0.65 - 0.66)	-0.93%
SFA	0.65 (0.65 - 0.66)	0.66 (0.66 - 0.67)	1.65%
MUFA	0.66 (0.65 - 0.66)	0.66 (0.66 - 0.67)	0.31%
PUFA	0.66 (0.66 - 0.67)	0.66 (0.65 - 0.66)	-1.07%
n-3 fatty acids	0.66 (0.66 - 0.67)	0.66 (0.65 - 0.66)	-0.20%
n-6 fatty acids	0.66 (0.66 - 0.67)	0.66 (0.65 - 0.66)	-1.31%
**Protein**			
Total protein	0.65 (0.65 - 0.66)	0.68 (0.67 - 0.69)	4.52%
Protein from plant sources	0.66 (0.66 - 0.67)	0.66 (0.65 - 0.66)	-0.63%
Protein from animal sources	0.65 (0.64 - 0.65)	0.68 (0.67 - 0.68)	4.37%
Protein from dairy	0.66 (0.66 - 0.67)	0.66 (0.66 - 0.67)	0.04%
Protein from non-dairy animal sources	0.65 (0.64 - 0.65)	0.68 (0.67 - 0.68)	4.30%

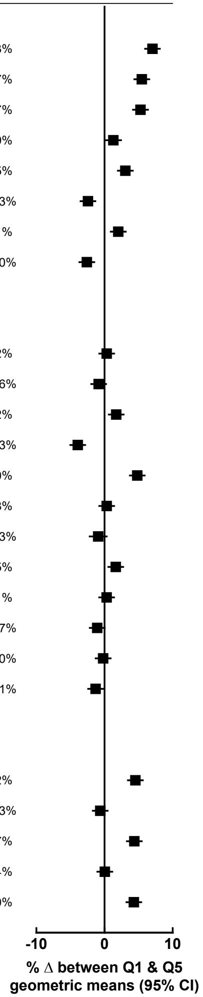

Supplemental Figure VII. **Minimally adjusted** geometric mean ApoB (mmol/L), ApoA1 (mmol/L) and ApoB-to-ApoA1 ratio by lowest (Q1) and highest (Q5) percentage intake of macronutrients.

Models adjusted for age at recruitment (<45, 45-49, 50-54, 55-59, 60-64, >65) and sex.

*For fiber Q1 and Q5 represent quintiles of total fiber intake (g/day).

Abbreviations: Apo = apolipoprotein; CI = confidence interval; g/day = grams per day; MUFA = monounsaturated fatty acids; n-3 = omega-3; n-6 = omega-6; PUFA = polyunsaturated fatty acids; Q = quintile; SFA = saturated fatty acids.

Nutrients	Total cholesterol (mmol/L) Geometric mean in Q1 (95% CI)	Total cholesterol (mmol/L) Geometric mean in Q5 (95% CI)	Total cholesterol (mmol/L) % Δ between Q1 & Q5 geometric means	LDL-C (mmol/L) Geometric mean in Q1 (95% CI)	LDL-C (mmol/L) Geometric mean in Q5 (95% CI)	LDL-C (mmol/L) % Δ between Q1 & Q5 geometric means	HDL-C (mmol/L) Geometric mean in Q1 (95% CI)	HDL-C (mmol/L) Geometric mean in Q5 (95% CI)	HDL-C (mmol/L) % Δ between Q1 & Q5 geometric means
**Carbohydrate**									
Total carbohydrates	5.89 (5.84 - 5.94)	5.72 (5.68 - 5.77)	-2.67%	3.62 (3.59 - 3.66)	3.56 (3.53 - 3.59)	-1.57%	1.57 (1.55 - 1.58)	1.45 (1.43 - 1.46)	-7.69%
Total sugars	5.81 (5.76 - 5.85)	5.84 (5.79 - 5.88)	0.67%	3.57 (3.54 - 3.61)	3.64 (3.61 - 3.67)	1.85%	1.53 (1.52 - 1.54)	1.47 (1.46 - 1.48)	-3.54%
Free sugars	5.80 (5.76 - 5.84)	5.88 (5.84 - 5.93)	1.18%	3.58 (3.54 - 3.61)	3.67 (3.64 - 3.70)	2.28%	1.53 (1.52 - 1.54)	1.46 (1.44 - 1.47)	-4.50%
Non-free sugars	5.91 (5.86 - 5.95)	5.78 (5.74 - 5.83)	-1.87%	3.67 (3.63 - 3.70)	3.59 (3.55 - 3.62)	-2.01%	1.50 (1.49 - 1.52)	1.50 (1.49 - 1.52)	-0.01%
Total starch	5.89 (5.84 - 5.93)	5.73 (5.69 - 5.77)	-2.61%	3.65 (3.61 - 3.68)	3.54 (3.51 - 3.57)	-2.75%	1.53 (1.52 - 1.55)	1.49 (1.47 - 1.50)	-3.19%
Starch from wholegrains	5.88 (5.83 - 5.92)	5.73 (5.69 - 5.78)	-2.14%	3.65 (3.62 - 3.69)	3.54 (3.51 - 3.58)	-2.74%	1.49 (1.48 - 1.50)	1.50 (1.48 - 1.51)	0.88%
Starch from refined grains	5.81 (5.76 - 5.85)	5.81 (5.77 - 5.86)	0.10%	3.59 (3.56 - 3.62)	3.60 (3.57 - 3.63)	0.33%	1.51 (1.50 - 1.53)	1.48 (1.47 - 1.50)	-2.28%
Fiber*	5.92 (5.88 - 5.97)	5.71 (5.67 - 5.76)	-3.20%	3.67 (3.64 - 3.71)	3.54 (3.50 - 3.57)	-3.49%	1.50 (1.49 - 1.52)	1.48 (1.47 - 1.50)	-0.86%
**Fat**									
Total fat	5.75 (5.71 - 5.79)	5.88 (5.83 - 5.92)	2.13%	3.57 (3.54 - 3.60)	3.63 (3.60 - 3.67)	1.71%	1.47 (1.45 - 1.48)	1.53 (1.52 - 1.55)	4.48%
Fat from plant sources	5.87 (5.83 - 5.91)	5.77 (5.72 - 5.81)	-1.70%	3.65 (3.62 - 3.68)	3.56 (3.53 - 3.60)	-2.17%	1.50 (1.49 - 1.52)	1.50 (1.48 - 1.51)	-0.47%
Fat from animal sources	5.74 (5.70 - 5.78)	5.94 (5.89 - 5.98)	3.29%	3.56 (3.53 - 3.59)	3.68 (3.65 - 3.71)	3.30%	1.47 (1.46 - 1.49)	1.54 (1.53 - 1.56)	4.75%
Fat from dairy	5.77 (5.73 - 5.81)	5.86 (5.82 - 5.90)	1.55%	3.57 (3.54 - 3.60)	3.63 (3.60 - 3.66)	1.61%	1.48 (1.46 - 1.49)	1.53 (1.52 - 1.54)	3.41%
Fat from non-dairy animal sources	5.76 (5.72 - 5.80)	5.92 (5.87 - 5.96)	2.60%	3.56 (3.53 - 3.60)	3.67 (3.64 - 3.71)	2.77%	1.49 (1.47 - 1.50)	1.52 (1.50 - 1.53)	2.17%
Dietary cholesterol	5.76 (5.72 - 5.80)	5.86 (5.82 - 5.91)	1.27%	3.57 (3.54 - 3.60)	3.63 (3.60 - 3.66)	1.08%	1.48 (1.47 - 1.49)	1.52 (1.50 - 1.53)	2.30%
Trans fatty acids	5.75 (5.70 - 5.80)	5.88 (5.84 - 5.93)	2.04%	3.57 (3.53 - 3.60)	3.64 (3.61 - 3.68)	1.83%	1.47 (1.45 - 1.48)	1.53 (1.52 - 1.55)	4.01%
SFA	5.74 (5.70 - 5.78)	5.92 (5.88 - 5.96)	3.07%	3.55 (3.51 - 3.58)	3.67 (3.64 - 3.71)	3.46%	1.48 (1.47 - 1.49)	1.54 (1.52 - 1.55)	3.70%
MUFA	5.77 (5.73 - 5.81)	5.83 (5.79 - 5.88)	1.02%	3.59 (3.55 - 3.62)	3.60 (3.57 - 3.64)	0.40%	1.47 (1.46 - 1.49)	1.52 (1.51 - 1.54)	3.28%
PUFA	5.85 (5.81 - 5.89)	5.78 (5.74 - 5.82)	-1.18%	3.65 (3.61 - 3.68)	3.57 (3.54 - 3.60)	-2.02%	1.48 (1.47 - 1.49)	1.50 (1.49 - 1.52)	1.36%
n-3 fatty acids	5.81 (5.77 - 5.86)	5.86 (5.82 - 5.91)	0.83%	3.61 (3.58 - 3.65)	3.63 (3.60 - 3.66)	0.43%	1.48 (1.46 - 1.49)	1.52 (1.51 - 1.54)	3.01%
n-6 fatty acids	5.86 (5.82 - 5.90)	5.78 (5.74 - 5.82)	-1.41%	3.65 (3.62 - 3.69)	3.57 (3.54 - 3.60)	-2.24%	1.48 (1.47 - 1.50)	1.50 (1.49 - 1.52)	1.06%
**Protein**									
Total protein	5.82 (5.77 - 5.86)	5.82 (5.78 - 5.87)	-0.43%	3.61 (3.58 - 3.65)	3.60 (3.57 - 3.63)	-1.08%	1.48 (1.47 - 1.50)	1.51 (1.50 - 1.52)	1.76%
Protein from plant sources	5.90 (5.85 - 5.94)	5.72 (5.68 - 5.76)	-2.44%	3.66 (3.62 - 3.69)	3.54 (3.50 - 3.57)	-2.73%	1.52 (1.50 - 1.53)	1.48 (1.47 - 1.50)	-1.90%
Protein from animal sources	5.78 (5.73 - 5.82)	5.84 (5.80 - 5.88)	0.72%	3.59 (3.56 - 3.62)	3.62 (3.58 - 3.65)	0.35%	1.47 (1.46 - 1.49)	1.51 (1.50 - 1.53)	2.41%
Protein from dairy	5.86 (5.81 - 5.90)	5.78 (5.74 - 5.82)	-1.39%	3.63 (3.60 - 3.67)	3.58 (3.55 - 3.61)	-1.75%	1.48 (1.47 - 1.50)	1.50 (1.49 - 1.51)	0.95%
Protein from non-dairy animal sources	5.75 (5.71 - 5.80)	5.83 (5.79 - 5.88)	1.07%	3.57 (3.54 - 3.60)	3.61 (3.58 - 3.65)	0.87%	1.48 (1.47 - 1.49)	1.50 (1.49 - 1.51)	1.28%

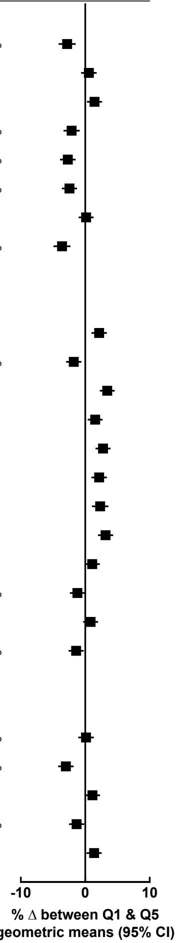

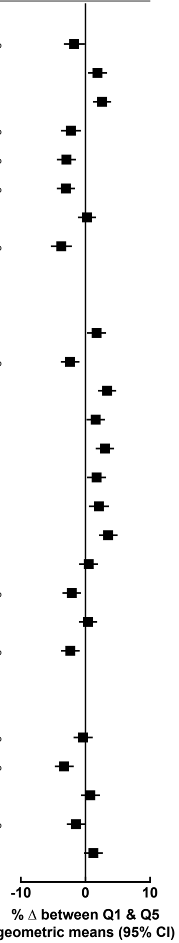

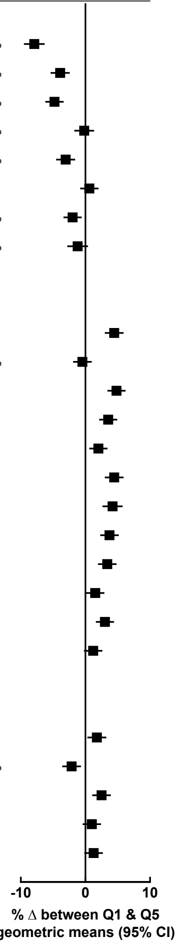

Supplemental Figure VIII. Geometric mean of total cholesterol (mmol/L), LDL-C (mmol/L) and HDL-C (mmol/L) by lowest (Q1) and highest (Q5) percentage intake of macronutrients **restricting to participants with ≥ 4 24-hour dietary assessments (including mandatory baseline assessment)** (n=9,605).

Models adjusted for age at recruitment (<45, 45-49, 50-54, 55-59, 60-64, ≥65), sex, ethnicity (white, mixed race, Asian or Asian British, black or black British, other, unknown), region (London, North-West England, North-Eastern England, Yorkshire & the Humber, West Midlands, East Midlands, South-West England, Wales), Townsend deprivation index (quintiles from least to most affluent, unknown), smoking status (never, previous, <15 cigarettes/day, 15-29 cigarettes/day, ≥30 cigarettes/day, unknown), physical activity (low, medium or high according to MET hours per week, unknown), alcohol (<1, 1-9, 10-19, 20-29, ≥30 g/day, unknown), body mass index (<20, 20-22.4, 22.5-24.9, 25-27.4, 27.5-29.9, 30-32.4, 32.5-34.9, 35-37.4, 37.5-39.9 or ≥40 in kg/m2), height (sex-specific groups in 5cm increments, unknown), diabetes diagnosed by a doctor (yes, no, unknown) and mean daily energy intake (quintiles).

*For fiber Q1 and Q5 represent quintiles of total fiber intake (g/day).

Abbreviations: CI = confidence interval; g/day = grams per day; HDL-C = high-density lipoprotein cholesterol; LDL-C= low-density lipoprotein cholesterol; MUFA = monounsaturated fatty acids; n-3 = omega-3; n-6 = omega-6; PUFA = polyunsaturated fatty acids; Q = quintile; SFA = saturated fatty acids.

	Triglycerides (mmol/L)			TC-to-HDL-C ratio			TG-to-HDL-C ratio		
Nutrients	Geometric mean in Q1 (95% CI)	Geometric mean in Q5 (95% CI)	% Δ between Q1 & Q5 geometric means	Geometric mean in Q1 (95% CI)	Geometric mean in Q5 (95% CI)	% Δ between Q1 & Q5 geometric means	Geometric mean in Q1 (95% CI)	Geometric mean in Q5 (95% CI)	% Δ between Q1 & Q5 geometric means
**Carbohydrate**									
Total carbohydrates	1.31 (1.29 - 1.34)	1.47 (1.44 - 1.50)	11.36%	3.76 (3.72 - 3.80)	3.95 (3.91 - 3.99)	5.02%	0.83 (0.81 - 0.86)	1.01 (0.99 - 1.04)	19.05%
Total sugars	1.36 (1.34 - 1.39)	1.44 (1.41 - 1.47)	5.82%	3.79 (3.75 - 3.83)	3.97 (3.93 - 4.01)	4.20%	0.89 (0.86 - 0.91)	0.98 (0.95 - 1.00)	9.35%
Free sugars	1.33 (1.30 - 1.36)	1.51 (1.48 - 1.54)	13.07%	3.79 (3.76 - 3.83)	4.03 (3.99 - 4.07)	5.68%	0.87 (0.85 - 0.89)	1.04 (1.01 - 1.07)	17.57%
Non-free sugars	1.45 (1.42 - 1.48)	1.35 (1.32 - 1.38)	-6.89%	3.93 (3.89 - 3.97)	3.85 (3.82 - 3.89)	-1.85%	0.96 (0.93 - 0.99)	0.90 (0.87 - 0.92)	-6.87%
Total starch	1.34 (1.32 - 1.37)	1.41 (1.39 - 1.44)	5.45%	3.84 (3.80 - 3.88)	3.85 (3.82 - 3.89)	0.58%	0.87 (0.85 - 0.90)	0.95 (0.93 - 0.97)	8.63%
Starch from wholegrains	1.42 (1.39 - 1.45)	1.38 (1.36 - 1.41)	-2.72%	3.94 (3.91 - 3.98)	3.83 (3.79 - 3.87)	-3.02%	0.95 (0.93 - 0.98)	0.92 (0.90 - 0.95)	-3.60%
Starch from refined grains	1.37 (1.34 - 1.39)	1.44 (1.41 - 1.47)	5.35%	3.84 (3.80 - 3.88)	3.92 (3.88 - 3.96)	2.38%	0.90 (0.88 - 0.92)	0.97 (0.95 - 0.99)	7.63%
Fiber*	1.43 (1.40 - 1.46)	1.38 (1.35 - 1.41)	-4.17%	3.94 (3.90 - 3.98)	3.85 (3.81 - 3.90)	-2.34%	0.95 (0.93 - 0.98)	0.93 (0.90 - 0.95)	-3.30%
**Fat**									
Total fat	1.43 (1.40 - 1.45)	1.36 (1.33 - 1.38)	-5.64%	3.92 (3.88 - 3.96)	3.83 (3.79 - 3.87)	-2.35%	0.97 (0.95 - 1.00)	0.88 (0.86 - 0.91)	-10.12%
Fat from plant sources	1.38 (1.35 - 1.40)	1.39 (1.37 - 1.42)	-0.03%	3.91 (3.87 - 3.94)	3.85 (3.81 - 3.89)	-1.23%	0.92 (0.90 - 0.94)	0.92 (0.90 - 0.95)	0.44%
Fat from animal sources	1.41 (1.39 - 1.44)	1.33 (1.31 - 1.36)	-6.44%	3.90 (3.86 - 3.94)	3.84 (3.80 - 3.88)	-1.45%	0.96 (0.94 - 0.99)	0.86 (0.84 - 0.88)	-11.19%
Fat from dairy	1.42 (1.39 - 1.45)	1.35 (1.32 - 1.37)	-5.61%	3.90 (3.87 - 3.94)	3.84 (3.80 - 3.87)	-1.86%	0.96 (0.94 - 0.99)	0.88 (0.86 - 0.90)	-9.02%
Fat from non-dairy animal sources	1.41 (1.39 - 1.44)	1.38 (1.35 - 1.40)	-3.04%	3.87 (3.84 - 3.91)	3.90 (3.86 - 3.94)	0.43%	0.95 (0.92 - 0.97)	0.90 (0.88 - 0.93)	-5.22%
Dietary cholesterol	1.42 (1.40 - 1.45)	1.37 (1.34 - 1.39)	-4.18%	3.90 (3.86 - 3.94)	3.86 (3.83 - 3.90)	-1.03%	0.96 (0.94 - 0.99)	0.90 (0.88 - 0.92)	-6.48%
Trans fatty acids	1.40 (1.37 - 1.43)	1.38 (1.35 - 1.40)	-3.20%	3.92 (3.87 - 3.96)	3.84 (3.80 - 3.89)	-1.97%	0.96 (0.93 - 0.99)	0.90 (0.87 - 0.92)	-7.21%
SFA	1.41 (1.38 - 1.44)	1.35 (1.32 - 1.37)	-4.78%	3.87 (3.84 - 3.91)	3.86 (3.82 - 3.89)	-0.64%	0.95 (0.92 - 0.97)	0.87 (0.85 - 0.90)	-8.48%
MUFA	1.42 (1.39 - 1.45)	1.36 (1.34 - 1.39)	-4.87%	3.92 (3.88 - 3.96)	3.83 (3.79 - 3.86)	-2.27%	0.96 (0.94 - 0.99)	0.89 (0.87 - 0.91)	-8.15%
PUFA	1.43 (1.40 - 1.46)	1.39 (1.36 - 1.41)	-3.86%	3.96 (3.92 - 3.99)	3.85 (3.81 - 3.88)	-2.53%	0.97 (0.94 - 0.99)	0.92 (0.90 - 0.94)	-5.22%
n-3 fatty acids	1.45 (1.42 - 1.48)	1.33 (1.30 - 1.35)	-9.00%	3.94 (3.90 - 3.98)	3.85 (3.81 - 3.89)	-2.17%	0.98 (0.95 - 1.00)	0.87 (0.85 - 0.89)	-12.01%
n-6 fatty acids	1.42 (1.39 - 1.45)	1.39 (1.37 - 1.42)	-2.72%	3.95 (3.91 - 3.99)	3.84 (3.81 - 3.88)	-2.47%	0.96 (0.93 - 0.98)	0.92 (0.90 - 0.94)	-3.78%
**Protein**									
Total protein	1.44 (1.42 - 1.47)	1.36 (1.33 - 1.38)	-6.58%	3.92 (3.89 - 3.96)	3.85 (3.81 - 3.88)	-2.20%	0.97 (0.95 - 0.99)	0.89 (0.87 - 0.92)	-8.34%
Protein from plant sources	1.39 (1.36 - 1.41)	1.41 (1.38 - 1.44)	1.45%	3.89 (3.85 - 3.93)	3.86 (3.83 - 3.90)	-0.54%	0.91 (0.89 - 0.94)	0.95 (0.93 - 0.97)	3.35%
Protein from animal sources	1.44 (1.41 - 1.47)	1.35 (1.32 - 1.37)	-6.57%	3.92 (3.88 - 3.96)	3.86 (3.82 - 3.90)	-1.69%	0.97 (0.95 - 1.00)	0.89 (0.87 - 0.91)	-8.98%
Protein from dairy	1.44 (1.41 - 1.47)	1.38 (1.36 - 1.41)	-3.89%	3.94 (3.91 - 3.98)	3.85 (3.82 - 3.89)	-2.34%	0.97 (0.95 - 0.99)	0.92 (0.90 - 0.94)	-4.84%
Protein from non-dairy animal sources	1.44 (1.41 - 1.46)	1.38 (1.35 - 1.40)	-4.16%	3.89 (3.85 - 3.93)	3.88 (3.84 - 3.92)	-0.21%	0.97 (0.94 - 0.99)	0.92 (0.89 - 0.94)	-5.44%

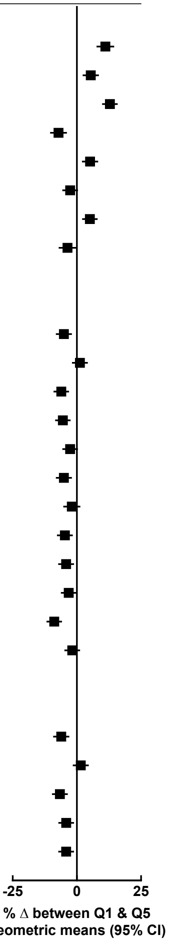

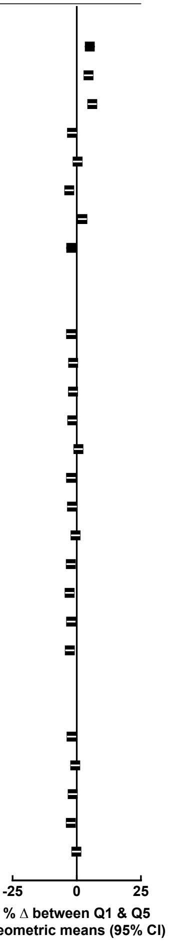

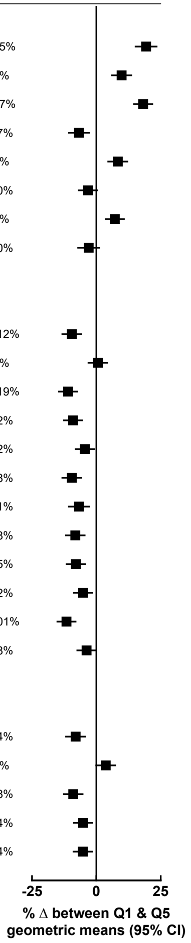

Supplemental Figure IX. Geometric mean of triglycerides (mmol/L), TC-to-HDL-C ratio and TG-to-HDL-C ratio by lowest (Q1) and highest (Q5) percentage intake of macronutrients restricting to participants with **≥ 4 24-hour dietary assessments** (including mandatory baseline assessment) (n=9,605).

Models adjusted for age at recruitment (<45, 45-49, 50-54, 55-59, 60-64, ≥65), sex, ethnicity (white, mixed race, Asian or Asian British, black or black British, other, unknown), region (London, North-West England, North-Eastern England, Yorkshire & the Humber, West Midlands, East Midlands, South-West England, Wales), Townsend deprivation index (quintiles from least to most affluent, unknown), smoking status (never, previous, <15 cigarettes/day, 15-29 cigarettes/day, ≥30 cigarettes/day, unknown), physical activity (low, medium or high according to MET hours per week, unknown), alcohol (<1, 1-9, 10-19, 20-29, ≥30 g/day, unknown), body mass index (<20, 20-22.4, 22.5-24.9, 25-27.4, 27.5-29.9, 30-32.4, 32.5-34.9, 35-37.4, 37.5-39.9 or ≥40 in kg/m2), height (sex-specific groups in 5cm increments, unknown), diabetes diagnosed by a doctor (yes, no, unknown) and mean daily energy intake (quintiles).

*For fiber Q1 and Q5 represent quintiles of total fiber intake (g/day).

Abbreviations: CI = confidence interval; g/day = grams per day; HDL-C = high-density lipoprotein cholesterol; MUFA = monounsaturated fatty acids; n-3 = omega-3; n-6 = omega-6; PUFA = polyunsaturated fatty acids; Q = quintile; SFA = saturated fatty acids; TC = total cholesterol; TG = triglycerides.

Nutrients	ApoB (mmol/L) Geometric mean in Q1 (95% CI)	ApoB (mmol/L) Geometric mean in Q5 (95% CI)	ApoB (mmol/L) % Δ between Q1 & Q5 geometric means	ApoA1 (mmol/L) Geometric mean in Q1 (95% CI)	ApoA1 (mmol/L) Geometric mean in Q5 (95% CI)	ApoA1 (mmol/L) % Δ between Q1 & Q5 geometric means	ApoB-to-ApoA1 ratio Geometric mean in Q1 (95% CI)	ApoB-to-ApoA1 ratio Geometric mean in Q5 (95% CI)	ApoB-to-ApoA1 ratio % Δ between Q1 & Q5 geometric means
**Carbohydrate**									
Total carbohydrates	1.04 (1.03 - 1.05)	1.03 (1.02 - 1.04)	-0.93%	1.62 (1.61 - 1.64)	1.53 (1.52 - 1.54)	-6.23%	0.64 (0.63 - 0.65)	0.67 (0.66 - 0.68)	5.31%
Total sugars	1.02 (1.01 - 1.03)	1.04 (1.04 - 1.05)	2.28%	1.60 (1.59 - 1.61)	1.54 (1.53 - 1.55)	-3.26%	0.64 (0.63 - 0.65)	0.68 (0.67 - 0.68)	5.54%
Free sugars	1.03 (1.02 - 1.04)	1.05 (1.04 - 1.06)	2.37%	1.58 (1.57 - 1.59)	1.55 (1.54 - 1.56)	-1.95%	0.65 (0.64 - 0.66)	0.68 (0.67 - 0.68)	4.32%
Non-free sugars	1.05 (1.04 - 1.06)	1.03 (1.02 - 1.04)	-1.69%	1.59 (1.58 - 1.60)	1.55 (1.54 - 1.56)	-2.24%	0.66 (0.65 - 0.67)	0.66 (0.65 - 0.67)	0.56%
Total starch	1.05 (1.04 - 1.05)	1.02 (1.01 - 1.03)	-2.66%	1.59 (1.58 - 1.60)	1.56 (1.55 - 1.57)	-2.03%	0.66 (0.65 - 0.66)	0.65 (0.64 - 0.66)	-0.64%
Starch from wholegrains	1.05 (1.04 - 1.06)	1.02 (1.01 - 1.03)	-2.52%	1.57 (1.56 - 1.58)	1.57 (1.56 - 1.58)	-0.50%	0.66 (0.66 - 0.67)	0.65 (0.64 - 0.66)	-2.02%
Starch from refined grains	1.03 (1.02 - 1.04)	1.03 (1.02 - 1.04)	0.47%	1.57 (1.56 - 1.58)	1.57 (1.56 - 1.58)	-0.64%	0.65 (0.65 - 0.66)	0.66 (0.65 - 0.67)	1.11%
Fiber*	1.05 (1.04 - 1.06)	1.02 (1.01 - 1.03)	-2.74%	1.59 (1.58 - 1.60)	1.55 (1.54 - 1.56)	-2.46%	0.66 (0.65 - 0.67)	0.66 (0.65 - 0.67)	-0.28%
**Fat**									
Total fat	1.03 (1.02 - 1.04)	1.03 (1.03 - 1.04)	0.52%	1.54 (1.53 - 1.55)	1.61 (1.60 - 1.62)	4.45%	0.67 (0.66 - 0.68)	0.64 (0.64 - 0.65)	-3.93%
Fat from plant sources	1.04 (1.03 - 1.05)	1.03 (1.02 - 1.04)	-1.66%	1.57 (1.56 - 1.58)	1.57 (1.56 - 1.58)	0.39%	0.66 (0.66 - 0.67)	0.65 (0.64 - 0.66)	-2.05%
Fat from animal sources	1.03 (1.02 - 1.04)	1.05 (1.04 - 1.05)	1.83%	1.54 (1.53 - 1.55)	1.60 (1.59 - 1.61)	3.99%	0.67 (0.66 - 0.67)	0.65 (0.64 - 0.66)	-2.16%
Fat from dairy	1.04 (1.03 - 1.04)	1.03 (1.02 - 1.04)	-0.28%	1.55 (1.54 - 1.56)	1.59 (1.58 - 1.60)	2.98%	0.67 (0.66 - 0.68)	0.65 (0.64 - 0.65)	-3.26%
Fat from non-dairy animal sources	1.02 (1.02 - 1.03)	1.05 (1.04 - 1.06)	2.41%	1.56 (1.55 - 1.57)	1.58 (1.57 - 1.59)	1.71%	0.66 (0.65 - 0.66)	0.66 (0.65 - 0.67)	0.70%
Dietary cholesterol	1.03 (1.02 - 1.04)	1.04 (1.03 - 1.05)	1.05%	1.56 (1.55 - 1.57)	1.58 (1.57 - 1.59)	1.60%	0.66 (0.65 - 0.67)	0.66 (0.65 - 0.66)	-0.55%
Trans fatty acids	1.03 (1.02 - 1.04)	1.03 (1.02 - 1.04)	0.16%	1.54 (1.53 - 1.55)	1.60 (1.59 - 1.61)	3.64%	0.67 (0.66 - 0.68)	0.65 (0.64 - 0.65)	-3.48%
SFA	1.03 (1.02 - 1.04)	1.04 (1.03 - 1.05)	1.39%	1.55 (1.54 - 1.56)	1.60 (1.59 - 1.61)	3.48%	0.66 (0.66 - 0.67)	0.65 (0.64 - 0.66)	-2.10%
MUFA	1.03 (1.02 - 1.04)	1.03 (1.02 - 1.04)	0.19%	1.54 (1.53 - 1.55)	1.60 (1.59 - 1.61)	3.66%	0.67 (0.66 - 0.67)	0.64 (0.64 - 0.65)	-3.47%
PUFA	1.04 (1.03 - 1.05)	1.03 (1.02 - 1.04)	-1.33%	1.55 (1.54 - 1.56)	1.58 (1.57 - 1.59)	1.34%	0.67 (0.66 - 0.68)	0.65 (0.64 - 0.66)	-2.67%
n-3 fatty acids	1.03 (1.02 - 1.04)	1.05 (1.04 - 1.06)	1.39%	1.56 (1.55 - 1.57)	1.58 (1.57 - 1.59)	1.04%	0.66 (0.65 - 0.67)	0.66 (0.66 - 0.67)	0.34%
n-6 fatty acids	1.04 (1.03 - 1.05)	1.03 (1.02 - 1.03)	-1.74%	1.55 (1.54 - 1.56)	1.58 (1.57 - 1.59)	1.51%	0.67 (0.66 - 0.68)	0.65 (0.64 - 0.66)	-3.25%
**Protein**									
Total protein	1.03 (1.02 - 1.04)	1.03 (1.02 - 1.04)	-0.48%	1.56 (1.55 - 1.57)	1.57 (1.56 - 1.58)	0.31%	0.66 (0.65 - 0.67)	0.66 (0.65 - 0.66)	-0.79%
Protein from plant sources	1.04 (1.03 - 1.05)	1.02 (1.01 - 1.03)	-1.84%	1.58 (1.57 - 1.59)	1.56 (1.55 - 1.57)	-1.82%	0.66 (0.65 - 0.66)	0.66 (0.65 - 0.66)	-0.02%
Protein from animal sources	1.03 (1.02 - 1.04)	1.04 (1.03 - 1.05)	0.37%	1.55 (1.54 - 1.56)	1.57 (1.56 - 1.58)	1.06%	0.66 (0.66 - 0.67)	0.66 (0.65 - 0.67)	-0.69%
Protein from dairy	1.05 (1.04 - 1.06)	1.02 (1.01 - 1.03)	-2.80%	1.56 (1.55 - 1.57)	1.57 (1.56 - 1.58)	0.29%	0.67 (0.66 - 0.68)	0.65 (0.64 - 0.66)	-3.09%
Protein from non-dairy animal sources	1.02 (1.01 - 1.03)	1.04 (1.03 - 1.05)	1.45%	1.56 (1.55 - 1.57)	1.57 (1.56 - 1.58)	0.39%	0.65 (0.65 - 0.66)	0.66 (0.65 - 0.67)	1.06%

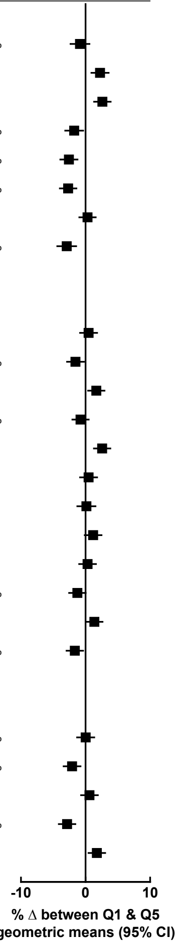

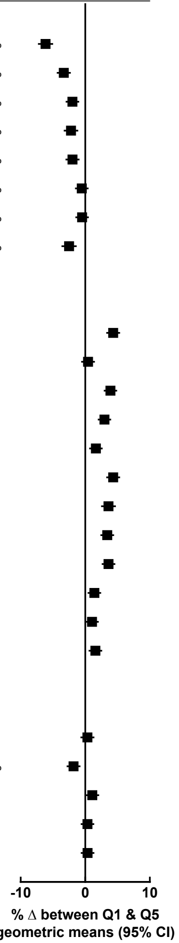

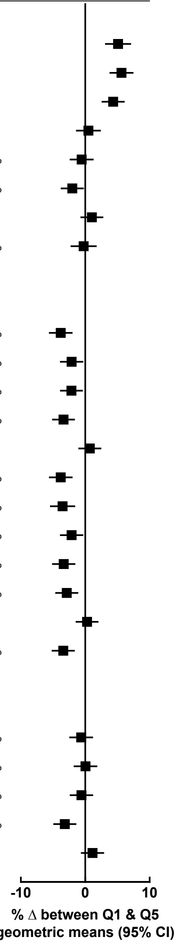

Supplemental Figure X. Geometric mean of ApoB (mmol/L), ApoA1 (mmol/L) and ApoB-to-ApoA1 ratio by lowest (Q1) and highest (Q5) percentage intake of macronutrients **restricting to participants with ≥ 4 24-hour dietary assessments (including mandatory baseline assessment)** (n=9,605).

Models adjusted for age at recruitment (<45, 45-49, 50-54, 55-59, 60-64, ≥65), sex, ethnicity (white, mixed race, Asian or Asian British, black or black British, other, unknown), region (London, North-West England, North-Eastern England, Yorkshire & the Humber, West Midlands, East Midlands, South-West England, Wales), Townsend deprivation index (quintiles from least to most affluent, unknown), smoking status (never, previous, <15 cigarettes/day, 15-29 cigarettes/day, ≥30 cigarettes/day, unknown), physical activity (low, medium or high according to MET hours per week, unknown), alcohol (<1, 1-9, 10-19, 20-29, ≥30 g/day, unknown), body mass index (<20, 20-22.4, 22.5-24.9, 25-27.4, 27.5-29.9, 30-32.4, 32.5-34.9, 35-37.4, 37.5-39.9 or ≥40 in kg/m2), height (sex-specific groups in 5cm increments, unknown), diabetes diagnosed by a doctor (yes, no, unknown) and mean daily energy intake (quintiles).

*For fiber Q1 and Q5 represent quintiles of total fiber intake (g/day).

Abbreviations: Apo = apolipoprotein; CI = confidence interval; g/day = grams per day; MUFA = monounsaturated fatty acids; n-3 = omega-3; n-6 = omega-6; PUFA = polyunsaturated fatty acids; Q = quintile; SFA = saturated fatty acids.

Nutrients	Total cholesterol (mmol/L) Geometric mean in Q1 (95% CI)	Total cholesterol Geometric mean in Q5 (95% CI)	Total cholesterol % Δ between Q1 & Q5 geometric means	LDL-C (mmol/L) Geometric mean in Q1 (95% CI)	LDL-C Geometric mean in Q5 (95% CI)	LDL-C % Δ between Q1 & Q5 geometric means	HDL-C (mmol/L) Geometric mean in Q1 (95% CI)	HDL-C Geometric mean in Q5 (95% CI)	HDL-C % Δ between Q1 & Q5 geometric means
**Carbohydrate**									
Total carbohydrates	5.91 (5.88 - 5.95)	5.70 (5.66 - 5.73)	-3.72%	3.65 (3.63 - 3.68)	3.54 (3.51 - 3.56)	-3.31%	1.56 (1.55 - 1.57)	1.45 (1.44 - 1.46)	-7.34%
Total sugars	5.82 (5.79 - 5.86)	5.78 (5.75 - 5.82)	-0.74%	3.60 (3.57 - 3.62)	3.60 (3.57 - 3.63)	0.10%	1.53 (1.51 - 1.54)	1.46 (1.45 - 1.47)	-4.46%
Free sugars	5.80 (5.77 - 5.84)	5.80 (5.77 - 5.84)	0.03%	3.58 (3.56 - 3.61)	3.62 (3.59 - 3.65)	1.06%	1.52 (1.51 - 1.54)	1.45 (1.44 - 1.46)	-4.80%
Non-free sugars	5.87 (5.83 - 5.90)	5.76 (5.73 - 5.80)	-1.83%	3.65 (3.62 - 3.67)	3.58 (3.55 - 3.60)	-1.98%	1.50 (1.49 - 1.51)	1.49 (1.48 - 1.50)	-0.63%
Total starch	5.87 (5.83 - 5.90)	5.72 (5.69 - 5.76)	-2.51%	3.64 (3.62 - 3.67)	3.54 (3.52 - 3.57)	-2.76%	1.52 (1.51 - 1.53)	1.47 (1.46 - 1.48)	-2.89%
Starch from wholegrains	5.85 (5.82 - 5.88)	5.76 (5.73 - 5.79)	-1.54%	3.64 (3.61 - 3.67)	3.56 (3.54 - 3.59)	-2.09%	1.49 (1.48 - 1.50)	1.49 (1.48 - 1.51)	0.33%
Starch from refined grains	5.83 (5.79 - 5.86)	5.80 (5.76 - 5.83)	-0.47%	3.61 (3.58 - 3.63)	3.59 (3.57 - 3.62)	-0.39%	1.51 (1.50 - 1.52)	1.48 (1.47 - 1.49)	-1.65%
Fiber*	5.88 (5.84 - 5.92)	5.73 (5.69 - 5.76)	-2.64%	3.66 (3.63 - 3.69)	3.55 (3.52 - 3.58)	-3.13%	1.50 (1.49 - 1.51)	1.48 (1.47 - 1.49)	-1.00%
**Fat**									
Total fat	5.73 (5.69 - 5.76)	5.88 (5.84 - 5.91)	2.63%	3.54 (3.52 - 3.57)	3.65 (3.62 - 3.67)	2.81%	1.47 (1.46 - 1.48)	1.53 (1.52 - 1.54)	4.11%
Fat from plant sources	5.84 (5.80 - 5.87)	5.76 (5.73 - 5.80)	-1.29%	3.63 (3.60 - 3.65)	3.57 (3.54 - 3.59)	-1.59%	1.50 (1.49 - 1.51)	1.49 (1.48 - 1.50)	-0.47%
Fat from animal sources	5.72 (5.69 - 5.76)	5.91 (5.88 - 5.95)	3.30%	3.54 (3.51 - 3.56)	3.68 (3.65 - 3.70)	3.84%	1.47 (1.46 - 1.48)	1.53 (1.52 - 1.54)	3.80%
Fat from dairy	5.79 (5.75 - 5.83)	5.85 (5.82 - 5.88)	0.99%	3.59 (3.56 - 3.62)	3.63 (3.61 - 3.66)	1.27%	1.48 (1.47 - 1.49)	1.51 (1.50 - 1.52)	1.94%
Fat from non-dairy animal sources	5.73 (5.69 - 5.76)	5.88 (5.85 - 5.91)	2.64%	3.54 (3.52 - 3.57)	3.65 (3.63 - 3.68)	3.02%	1.47 (1.46 - 1.48)	1.51 (1.50 - 1.52)	2.36%
Dietary cholesterol	5.73 (5.70 - 5.76)	5.84 (5.80 - 5.87)	1.88%	3.55 (3.52 - 3.57)	3.62 (3.59 - 3.65)	1.94%	1.47 (1.46 - 1.48)	1.51 (1.50 - 1.52)	2.58%
Trans fatty acids	5.76 (5.73 - 5.80)	5.88 (5.85 - 5.91)	2.05%	3.57 (3.54 - 3.59)	3.66 (3.63 - 3.68)	2.56%	1.49 (1.47 - 1.50)	1.51 (1.50 - 1.52)	1.91%
SFA	5.72 (5.68 - 5.75)	5.92 (5.88 - 5.95)	3.41%	3.53 (3.50 - 3.56)	3.68 (3.66 - 3.71)	4.31%	1.48 (1.47 - 1.49)	1.53 (1.52 - 1.54)	2.95%
MUFA	5.72 (5.68 - 5.75)	5.85 (5.82 - 5.89)	2.32%	3.54 (3.52 - 3.57)	3.63 (3.60 - 3.65)	2.32%	1.47 (1.46 - 1.48)	1.52 (1.51 - 1.53)	3.60%
PUFA	5.80 (5.77 - 5.84)	5.77 (5.73 - 5.80)	-0.62%	3.61 (3.59 - 3.64)	3.56 (3.54 - 3.59)	-1.35%	1.47 (1.46 - 1.48)	1.51 (1.49 - 1.52)	2.38%
n-3 fatty acids	5.79 (5.76 - 5.82)	5.85 (5.81 - 5.88)	0.95%	3.60 (3.58 - 3.63)	3.62 (3.59 - 3.64)	0.41%	1.46 (1.45 - 1.47)	1.53 (1.52 - 1.54)	4.45%
n-6 fatty acids	5.82 (5.79 - 5.86)	5.76 (5.73 - 5.79)	-1.08%	3.62 (3.60 - 3.65)	3.56 (3.53 - 3.59)	-1.73%	1.48 (1.47 - 1.49)	1.50 (1.49 - 1.51)	1.53%
**Protein**									
Total protein	5.77 (5.74 - 5.80)	5.82 (5.78 - 5.85)	0.82%	3.59 (3.56 - 3.61)	3.60 (3.57 - 3.63)	0.46%	1.48 (1.47 - 1.49)	1.50 (1.49 - 1.52)	1.89%
Protein from plant sources	5.87 (5.83 - 5.90)	5.71 (5.67 - 5.74)	-2.76%	3.65 (3.62 - 3.67)	3.53 (3.50 - 3.55)	-3.32%	1.51 (1.50 - 1.52)	1.48 (1.47 - 1.49)	-1.98%
Protein from animal sources	5.72 (5.69 - 5.76)	5.85 (5.81 - 5.88)	2.08%	3.55 (3.53 - 3.58)	3.63 (3.60 - 3.65)	2.07%	1.47 (1.46 - 1.48)	1.51 (1.50 - 1.52)	2.79%
Protein from dairy	5.82 (5.78 - 5.85)	5.81 (5.78 - 5.85)	-0.09%	3.61 (3.58 - 3.64)	3.61 (3.58 - 3.64)	-0.09%	1.49 (1.48 - 1.50)	1.50 (1.49 - 1.51)	0.56%
Protein from non-dairy animal sources	5.72 (5.69 - 5.75)	5.83 (5.79 - 5.86)	1.82%	3.55 (3.53 - 3.58)	3.61 (3.58 - 3.64)	1.61%	1.47 (1.46 - 1.48)	1.50 (1.49 - 1.52)	2.47%

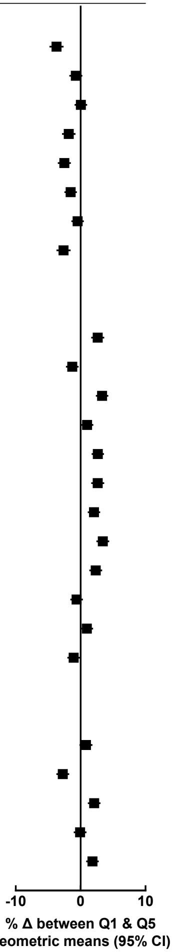

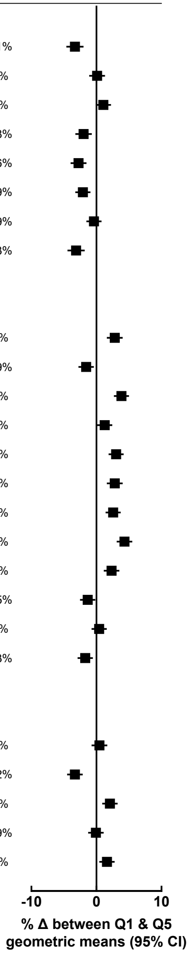

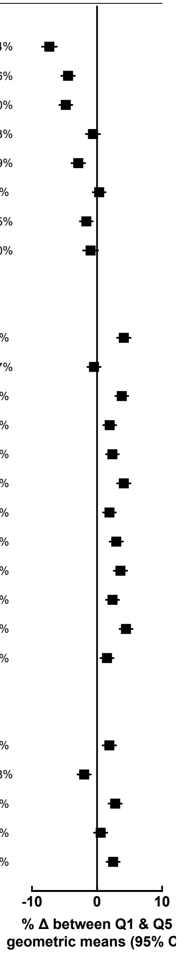

Supplemental Figure XI. Geometric mean of total cholesterol (mmol/L), LDL-C (mmol/L) and HDL-C (mmol/L) by lowest (Q1) and highest (Q5) percentage intake of macronutrients **excluding participants with a self-reported change in diet in the prior five years** (n=16,072).

Models adjusted for age at recruitment (<45, 45-49, 50-54, 55-59, 60-64, ≥65), sex, ethnicity (white, mixed race, Asian or Asian British, black or black British, other, unknown), region (London, North-West England, North-Eastern England, Yorkshire & the Humber, West Midlands, East Midlands, South-West England, Wales), Townsend deprivation index (quintiles from least to most affluent, unknown), smoking status (never, previous, <15 cigarettes/day, 15-29 cigarettes/day, ≥30 cigarettes/day, unknown), physical activity (low, medium or high according to MET hours per week, unknown), alcohol (<1, 1-9, 10-19, 20-29, ≥30 g/day, unknown), body mass index (<20, 20-22.4, 22.5-24.9, 25-27.4, 27.5-29.9, 30-32.4, 32.5-34.9, 35-37.4, 37.5-39.9 or ≥40 in kg/m2), height (sex-specific groups in 5cm increments, unknown), diabetes diagnosed by a doctor (yes, no, unknown) and mean daily energy intake (quintiles).

*For fiber Q1 and Q5 represent quintiles of total fiber intake (g/day).

Abbreviations: CI = confidence interval; g/day = grams per day; HDL-C = high-density lipoprotein cholesterol; LDL-C= low-density lipoprotein cholesterol; MUFA = monounsaturated fatty acids; n-3 = omega-3; n-6 = omega-6; PUFA = polyunsaturated fatty acids; Q = quintile; SFA = saturated fatty acids.

Nutrients	Triglycerides (mmol/L): Geometric mean in Q1 (95% CI)	Triglycerides (mmol/L): Geometric mean in Q5 (95% CI)	Triglycerides (mmol/L): % Δ between Q1 & Q5 geometric means	TC-to-HDL-C ratio: Geometric mean in Q1 (95% CI)	TC-to-HDL-C ratio: Geometric mean in Q5 (95% CI)	TC-to-HDL-C ratio: % Δ between Q1 & Q5 geometric means	TG-to-HDL-C ratio: Geometric mean in Q1 (95% CI)	TG-to-HDL-C ratio: Geometric mean in Q5 (95% CI)	TG-to-HDL-C ratio: % Δ between Q1 & Q5 geometric means
**Carbohydrate**									
Total carbohydrates	1.31 (1.29 - 1.33)	1.46 (1.43 - 1.48)	10.86%	3.79 (3.76 - 3.82)	3.93 (3.90 - 3.97)	3.62%	0.84 (0.82 - 0.86)	1.01 (0.98 - 1.03)	18.20%
Total sugars	1.33 (1.31 - 1.35)	1.44 (1.42 - 1.46)	7.66%	3.82 (3.79 - 3.85)	3.96 (3.93 - 4.00)	3.71%	0.87 (0.86 - 0.89)	0.99 (0.97 - 1.01)	12.12%
Free sugars	1.33 (1.31 - 1.35)	1.48 (1.46 - 1.51)	11.24%	3.81 (3.78 - 3.84)	3.99 (3.96 - 4.02)	4.84%	0.87 (0.85 - 0.89)	1.02 (1.00 - 1.04)	16.04%
Non-free sugars	1.42 (1.39 - 1.44)	1.36 (1.34 - 1.39)	-3.72%	3.92 (3.89 - 3.95)	3.87 (3.84 - 3.90)	-1.20%	0.95 (0.93 - 0.96)	0.92 (0.90 - 0.94)	-3.09%
Total starch	1.35 (1.33 - 1.37)	1.41 (1.39 - 1.43)	4.73%	3.87 (3.84 - 3.90)	3.88 (3.85 - 3.91)	0.37%	0.89 (0.87 - 0.91)	0.96 (0.94 - 0.98)	7.62%
Starch from wholegrains	1.40 (1.37 - 1.42)	1.39 (1.37 - 1.42)	-0.12%	3.93 (3.90 - 3.96)	3.85 (3.83 - 3.88)	-1.87%	0.94 (0.92 - 0.96)	0.93 (0.92 - 0.95)	-0.45%
Starch from refined grains	1.37 (1.35 - 1.39)	1.42 (1.40 - 1.44)	3.21%	3.86 (3.83 - 3.89)	3.90 (3.88 - 3.93)	1.18%	0.91 (0.89 - 0.93)	0.96 (0.94 - 0.97)	4.85%
Fiber*	1.40 (1.38 - 1.42)	1.38 (1.36 - 1.40)	-1.41%	3.93 (3.90 - 3.96)	3.86 (3.83 - 3.90)	-1.64%	0.93 (0.92 - 0.95)	0.93 (0.91 - 0.95)	-0.41%
**Fat**									
Total fat	1.43 (1.40 - 1.45)	1.33 (1.31 - 1.35)	-7.40%	3.90 (3.87 - 3.93)	3.84 (3.81 - 3.87)	-1.48%	0.97 (0.95 - 0.99)	0.87 (0.85 - 0.88)	-11.51%
Fat from plant sources	1.38 (1.36 - 1.41)	1.37 (1.35 - 1.39)	-1.17%	3.89 (3.86 - 3.92)	3.86 (3.83 - 3.89)	-0.83%	0.92 (0.91 - 0.94)	0.92 (0.90 - 0.93)	-0.70%
Fat from animal sources	1.42 (1.40 - 1.45)	1.34 (1.32 - 1.36)	-5.70%	3.89 (3.86 - 3.92)	3.87 (3.84 - 3.90)	-0.50%	0.97 (0.95 - 0.99)	0.88 (0.86 - 0.90)	-9.50%
Fat from dairy	1.40 (1.38 - 1.43)	1.35 (1.33 - 1.37)	-3.88%	3.90 (3.87 - 3.93)	3.86 (3.84 - 3.89)	-0.95%	0.94 (0.93 - 0.96)	0.89 (0.88 - 0.91)	-5.83%
Fat from non-dairy animal sources	1.42 (1.39 - 1.44)	1.37 (1.35 - 1.39)	-3.47%	3.88 (3.86 - 3.91)	3.90 (3.87 - 3.93)	0.29%	0.96 (0.94 - 0.98)	0.91 (0.89 - 0.92)	-5.83%
Dietary cholesterol	1.40 (1.38 - 1.42)	1.37 (1.34 - 1.39)	-2.49%	3.89 (3.86 - 3.92)	3.86 (3.83 - 3.89)	-0.70%	0.95 (0.93 - 0.97)	0.90 (0.89 - 0.92)	-5.07%
Trans fatty acids	1.39 (1.37 - 1.41)	1.38 (1.36 - 1.40)	-0.74%	3.88 (3.85 - 3.91)	3.88 (3.86 - 3.91)	0.14%	0.93 (0.92 - 0.95)	0.91 (0.89 - 0.93)	-2.66%
SFA	1.39 (1.37 - 1.42)	1.35 (1.33 - 1.37)	-3.53%	3.86 (3.83 - 3.89)	3.88 (3.85 - 3.90)	0.46%	0.94 (0.92 - 0.96)	0.88 (0.87 - 0.90)	-6.48%
MUFA	1.42 (1.40 - 1.45)	1.34 (1.32 - 1.37)	-5.74%	3.90 (3.87 - 3.93)	3.85 (3.82 - 3.88)	-1.27%	0.97 (0.95 - 0.99)	0.88 (0.87 - 0.90)	-9.34%
PUFA	1.45 (1.43 - 1.47)	1.34 (1.32 - 1.36)	-7.76%	3.95 (3.92 - 3.98)	3.83 (3.80 - 3.86)	-3.00%	0.99 (0.97 - 1.01)	0.89 (0.87 - 0.91)	-10.13%
n-3 fatty acids	1.46 (1.44 - 1.48)	1.30 (1.28 - 1.33)	-11.39%	3.96 (3.93 - 3.99)	3.82 (3.79 - 3.85)	-3.50%	1.00 (0.98 - 1.02)	0.85 (0.84 - 0.87)	-15.84%
n-6 fatty acids	1.43 (1.41 - 1.45)	1.35 (1.33 - 1.38)	-5.31%	3.94 (3.91 - 3.97)	3.84 (3.81 - 3.87)	-2.61%	0.97 (0.95 - 0.99)	0.90 (0.89 - 0.92)	-6.84%
**Protein**									
Total protein	1.41 (1.39 - 1.44)	1.36 (1.33 - 1.38)	-4.22%	3.91 (3.88 - 3.94)	3.87 (3.83 - 3.90)	-1.07%	0.96 (0.94 - 0.98)	0.90 (0.88 - 0.92)	-6.11%
Protein from plant sources	1.37 (1.35 - 1.39)	1.39 (1.37 - 1.42)	1.79%	3.89 (3.87 - 3.92)	3.86 (3.83 - 3.90)	-0.77%	0.91 (0.89 - 0.93)	0.94 (0.93 - 0.96)	3.77%
Protein from animal sources	1.41 (1.39 - 1.43)	1.35 (1.33 - 1.38)	-4.31%	3.90 (3.87 - 3.93)	3.87 (3.84 - 3.90)	-0.71%	0.96 (0.94 - 0.98)	0.90 (0.88 - 0.91)	-7.09%
Protein from dairy	1.40 (1.38 - 1.42)	1.38 (1.36 - 1.41)	-1.26%	3.91 (3.88 - 3.94)	3.88 (3.86 - 3.91)	-0.64%	0.94 (0.92 - 0.96)	0.93 (0.91 - 0.94)	-1.82%
Protein from non-dairy animal sources	1.41 (1.39 - 1.43)	1.37 (1.35 - 1.39)	-3.03%	3.90 (3.87 - 3.93)	3.87 (3.84 - 3.90)	-0.65%	0.96 (0.94 - 0.98)	0.91 (0.89 - 0.93)	-5.50%

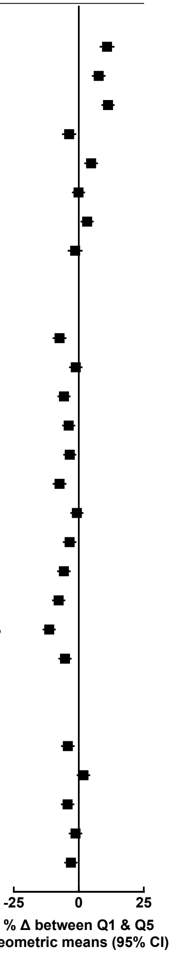

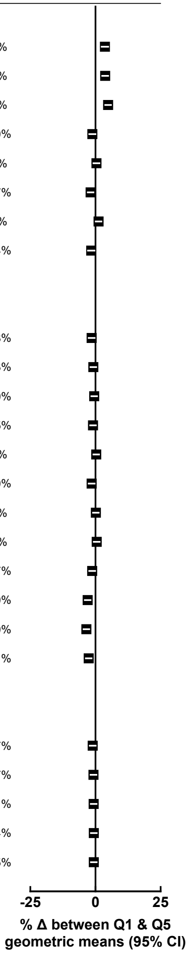

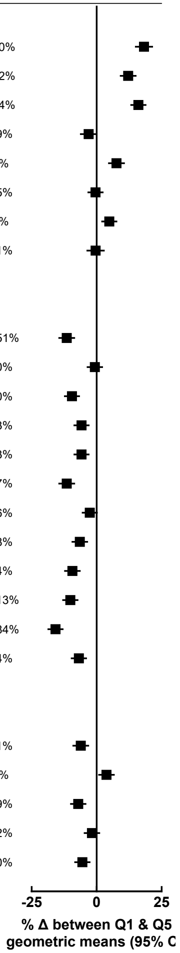

Supplemental Figure XII. Geometric mean of triglycerides (mmol/L), TC-to-HDL-C ratio and TG-to-HDL-C ratio by lowest (Q1) and highest (Q5) percentage intake of macronutrients **excluding participants with a self-reported change in diet in the prior five years** (n=16,072).

Models adjusted for age at recruitment (<45, 45-49, 50-54, 55-59, 60-64, ≥65), sex, ethnicity (white, mixed race, Asian or Asian British, black or black British, other, unknown), region (London, North-West England, North-Eastern England, Yorkshire & the Humber, West Midlands, East Midlands, South-West England, Wales), Townsend deprivation index (quintiles from least to most affluent, unknown), smoking status (never, previous, <15 cigarettes/day, 15-29 cigarettes/day, ≥30 cigarettes/day, unknown), physical activity (low, medium or high according to MET hours per week, unknown), alcohol (<1, 1-9, 10-19, 20-29, ≥30 g/day, unknown), body mass index (<20, 20-22.4, 22.5-24.9, 25-27.4, 27.5-29.9, 30-32.4, 32.5-34.9, 35-37.4, 37.5-39.9 or ≥40 in kg/m2), height (sex-specific groups in 5cm increments, unknown), diabetes diagnosed by a doctor (yes, no, unknown) and mean daily energy intake (quintiles).

*For fiber Q1 and Q5 represent quintiles of total fiber intake (g/day).

Abbreviations: CI = confidence interval; g/day = grams per day; HDL-C = high-density lipoprotein cholesterol; MUFA = monounsaturated fatty acids; n-3 = omega-3; n-6 = omega-6; PUFA = polyunsaturated fatty acids; Q = quintile; SFA = saturated fatty acids; TC = total cholesterol; TG = triglycerides.

Nutrients	ApoB (mmol/L) Geometric mean in Q1 (95% CI)	ApoB (mmol/L) Geometric mean in Q5 (95% CI)	ApoB (mmol/L) % Δ between Q1 & Q5 geometric means	ApoA1 (mmol/L) Geometric mean in Q1 (95% CI)	ApoA1 (mmol/L) Geometric mean in Q5 (95% CI)	ApoA1 (mmol/L) % Δ between Q1 & Q5 geometric means	ApoB-to-ApoA1 ratio Geometric mean in Q1 (95% CI)	ApoB-to-ApoA1 ratio Geometric mean in Q5 (95% CI)	ApoB-to-ApoA1 ratio % Δ between Q1 & Q5 geometric means
**Carbohydrate**									
Total carbohydrates	1.04 (1.04 - 1.05)	1.01 (1.01 - 1.02)	-2.94%	1.63 (1.62 - 1.63)	1.53 (1.53 - 1.54)	-5.81%	0.64 (0.64 - 0.65)	0.66 (0.65 - 0.67)	2.88%
Total sugars	1.03 (1.02 - 1.04)	1.03 (1.02 - 1.04)	0.47%	1.60 (1.59 - 1.61)	1.54 (1.53 - 1.55)	-3.61%	0.64 (0.64 - 0.65)	0.67 (0.66 - 0.68)	4.07%
Free sugars	1.02 (1.02 - 1.03)	1.04 (1.03 - 1.04)	1.27%	1.59 (1.58 - 1.60)	1.55 (1.54 - 1.56)	-2.56%	0.64 (0.64 - 0.65)	0.67 (0.66 - 0.68)	3.83%
Non-free sugars	1.04 (1.03 - 1.05)	1.03 (1.02 - 1.03)	-1.50%	1.59 (1.58 - 1.59)	1.55 (1.55 - 1.56)	-2.06%	0.66 (0.65 - 0.66)	0.66 (0.65 - 0.67)	0.55%
Total starch	1.04 (1.04 - 1.05)	1.01 (1.01 - 1.02)	-2.95%	1.59 (1.58 - 1.60)	1.56 (1.55 - 1.57)	-1.98%	0.66 (0.65 - 0.66)	0.65 (0.64 - 0.66)	-0.96%
Starch from wholegrains	1.04 (1.03 - 1.05)	1.02 (1.01 - 1.03)	-2.08%	1.58 (1.57 - 1.59)	1.57 (1.56 - 1.58)	-0.64%	0.66 (0.65 - 0.67)	0.65 (0.64 - 0.66)	-1.44%
Starch from refined grains	1.03 (1.02 - 1.04)	1.03 (1.02 - 1.03)	-0.39%	1.58 (1.57 - 1.59)	1.57 (1.56 - 1.58)	-0.63%	0.65 (0.65 - 0.66)	0.65 (0.65 - 0.66)	0.24%
Fiber*	1.04 (1.04 - 1.05)	1.02 (1.01 - 1.03)	-2.38%	1.59 (1.58 - 1.59)	1.56 (1.55 - 1.56)	-1.96%	0.66 (0.65 - 0.67)	0.66 (0.65 - 0.66)	-0.42%
**Fat**									
Total fat	1.02 (1.01 - 1.03)	1.04 (1.03 - 1.04)	1.68%	1.55 (1.54 - 1.55)	1.61 (1.60 - 1.61)	3.86%	0.66 (0.65 - 0.67)	0.65 (0.64 - 0.65)	-2.18%
Fat from plant sources	1.03 (1.03 - 1.04)	1.02 (1.02 - 1.03)	-1.05%	1.57 (1.57 - 1.58)	1.57 (1.57 - 1.58)	0.00%	0.66 (0.65 - 0.66)	0.65 (0.64 - 0.66)	-1.04%
Fat from animal sources	1.02 (1.01 - 1.03)	1.04 (1.04 - 1.05)	2.49%	1.55 (1.54 - 1.56)	1.60 (1.59 - 1.61)	3.15%	0.66 (0.65 - 0.66)	0.65 (0.65 - 0.66)	-0.66%
Fat from dairy	1.03 (1.03 - 1.04)	1.03 (1.02 - 1.04)	-0.48%	1.56 (1.56 - 1.57)	1.59 (1.58 - 1.59)	1.54%	0.66 (0.66 - 0.67)	0.65 (0.64 - 0.65)	-2.02%
Fat from non-dairy animal sources	1.02 (1.01 - 1.02)	1.04 (1.04 - 1.05)	2.72%	1.56 (1.55 - 1.56)	1.59 (1.58 - 1.60)	2.08%	0.65 (0.65 - 0.66)	0.66 (0.65 - 0.66)	0.65%
Dietary cholesterol	1.02 (1.01 - 1.02)	1.04 (1.03 - 1.04)	1.99%	1.55 (1.55 - 1.56)	1.59 (1.58 - 1.59)	1.99%	0.65 (0.65 - 0.66)	0.65 (0.65 - 0.66)	0.00%
Trans fatty acids	1.03 (1.02 - 1.03)	1.04 (1.03 - 1.04)	1.16%	1.56 (1.55 - 1.57)	1.59 (1.58 - 1.60)	1.95%	0.66 (0.65 - 0.66)	0.65 (0.65 - 0.66)	-0.80%
SFA	1.02 (1.01 - 1.03)	1.04 (1.03 - 1.05)	2.10%	1.56 (1.55 - 1.56)	1.60 (1.59 - 1.61)	2.83%	0.65 (0.65 - 0.66)	0.65 (0.64 - 0.66)	-0.72%
MUFA	1.02 (1.01 - 1.02)	1.04 (1.03 - 1.04)	1.84%	1.55 (1.54 - 1.55)	1.60 (1.59 - 1.61)	3.48%	0.66 (0.65 - 0.66)	0.65 (0.64 - 0.65)	-1.65%
PUFA	1.03 (1.02 - 1.04)	1.02 (1.02 - 1.03)	-0.64%	1.56 (1.55 - 1.57)	1.58 (1.57 - 1.59)	1.56%	0.66 (0.66 - 0.67)	0.65 (0.64 - 0.65)	-2.20%
n-3 fatty acids	1.03 (1.02 - 1.04)	1.04 (1.03 - 1.05)	1.15%	1.56 (1.55 - 1.56)	1.59 (1.58 - 1.60)	2.07%	0.66 (0.65 - 0.67)	0.65 (0.65 - 0.66)	-0.92%
n-6 fatty acids	1.03 (1.03 - 1.04)	1.02 (1.01 - 1.03)	-1.17%	1.56 (1.55 - 1.57)	1.58 (1.57 - 1.59)	1.28%	0.66 (0.66 - 0.67)	0.65 (0.64 - 0.65)	-2.45%
**Protein**									
Total protein	1.02 (1.02 - 1.03)	1.03 (1.03 - 1.04)	0.99%	1.56 (1.55 - 1.57)	1.58 (1.57 - 1.59)	0.95%	0.66 (0.65 - 0.66)	0.66 (0.65 - 0.66)	0.04%
Protein from plant sources	1.04 (1.03 - 1.05)	1.01 (1.01 - 1.02)	-2.37%	1.59 (1.58 - 1.59)	1.55 (1.55 - 1.56)	-1.93%	0.66 (0.65 - 0.66)	0.65 (0.65 - 0.66)	-0.44%
Protein from animal sources	1.02 (1.01 - 1.02)	1.04 (1.03 - 1.05)	2.17%	1.56 (1.55 - 1.56)	1.58 (1.58 - 1.59)	1.86%	0.65 (0.65 - 0.66)	0.66 (0.65 - 0.66)	0.32%
Protein from dairy	1.04 (1.03 - 1.04)	1.03 (1.02 - 1.04)	-0.78%	1.57 (1.56 - 1.58)	1.57 (1.56 - 1.58)	-0.17%	0.66 (0.65 - 0.67)	0.66 (0.65 - 0.66)	-0.61%
Protein from non-dairy animal sources	1.02 (1.01 - 1.02)	1.04 (1.03 - 1.05)	2.11%	1.55 (1.55 - 1.56)	1.58 (1.57 - 1.59)	1.85%	0.65 (0.65 - 0.66)	0.66 (0.65 - 0.66)	0.26%

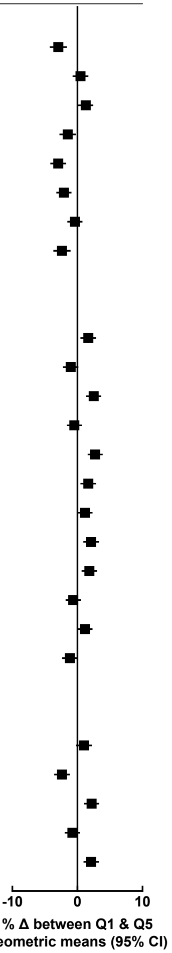

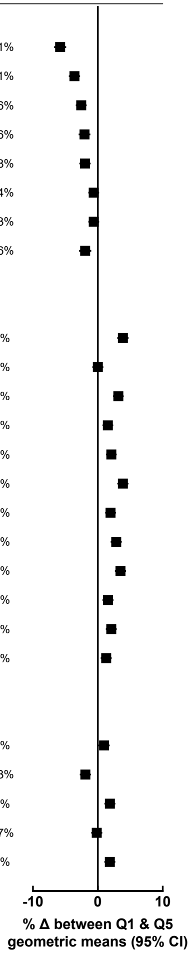

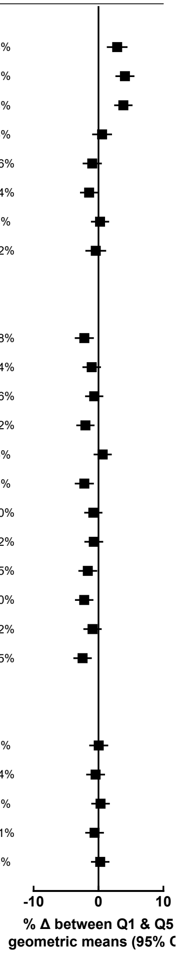

Supplemental Figure XIII. Geometric mean of ApoB (mmol/L), ApoA1 (mmol/L) and ApoB-to-ApoA1 ratio by lowest (Q1) and highest (Q5) percentage intake of macronutrients **excluding participants with a self-reported change in diet in the prior five years** (n=16,072).

Models adjusted for age at recruitment (<45, 45-49, 50-54, 55-59, 60-64, ≥65), sex, ethnicity (white, mixed race, Asian or Asian British, black or black British, other, unknown), region (London, North-West England, North-Eastern England, Yorkshire & the Humber, West Midlands, East Midlands, South-West England, Wales), Townsend deprivation index (quintiles from least to most affluent, unknown), smoking status (never, previous, <15 cigarettes/day, 15-29 cigarettes/day, ≥30 cigarettes/day, unknown), physical activity (low, medium or high according to MET hours per week, unknown), alcohol (<1, 1-9, 10-19, 20-29, ≥30 g/day, unknown), body mass index (<20, 20-22.4, 22.5-24.9, 25-27.4, 27.5-29.9, 30-32.4, 32.5-34.9, 35-37.4, 37.5-39.9 or ≥40 in kg/m2), height (sex-specific groups in 5cm increments, unknown), diabetes diagnosed by a doctor (yes, no, unknown) and mean daily energy intake (quintiles).

*For fiber Q1 and Q5 represent quintiles of total fiber intake (g/day).

Abbreviations: Apo = apolipoprotein; CI = confidence interval; g/day = grams per day; MUFA = monounsaturated fatty acids; n-3 = omega-3; n-6 = omega-6; PUFA = polyunsaturated fatty acids; Q = quintile; SFA = saturated fatty acids.

Nutrients	Total cholesterol (mmol/L) Geometric mean in Q1 (95% CI)	Geometric mean in Q5 (95% CI)	% Δ between Q1 & Q5 geometric means	LDL-C (mmol/L) Geometric mean in Q1 (95% CI)	Geometric mean in Q5 (95% CI)	% Δ between Q1 & Q5 geometric means	HDL-C (mmol/L) Geometric mean in Q1 (95% CI)	Geometric mean in Q5 (95% CI)	% Δ between Q1 & Q5 geometric means
**Carbohydrate**									
Total carbohydrates	5.90 (5.87 - 5.93)	5.69 (5.66 - 5.72)	-3.53%	3.64 (3.62 - 3.66)	3.54 (3.51 - 3.56)	-2.81%	1.55 (1.54 - 1.56)	1.43 (1.43 - 1.44)	-7.45%
Total sugars	5.83 (5.80 - 5.85)	5.78 (5.75 - 5.80)	-0.71%	3.60 (3.58 - 3.62)	3.60 (3.58 - 3.62)	0.17%	1.51 (1.51 - 1.52)	1.45 (1.44 - 1.46)	-3.86%
Free sugars	5.78 (5.75 - 5.80)	5.82 (5.79 - 5.84)	0.58%	3.56 (3.54 - 3.58)	3.62 (3.60 - 3.65)	1.71%	1.51 (1.50 - 1.52)	1.45 (1.44 - 1.45)	-4.27%
Non-free sugars	5.88 (5.85 - 5.90)	5.74 (5.71 - 5.77)	-2.06%	3.65 (3.63 - 3.67)	3.56 (3.54 - 3.58)	-2.13%	1.49 (1.48 - 1.50)	1.48 (1.47 - 1.49)	-0.58%
Total starch	5.86 (5.84 - 5.89)	5.70 (5.68 - 5.73)	-2.53%	3.64 (3.62 - 3.66)	3.53 (3.51 - 3.55)	-2.64%	1.51 (1.51 - 1.52)	1.46 (1.45 - 1.47)	-3.66%
Starch from wholegrains	5.85 (5.82 - 5.88)	5.73 (5.71 - 5.76)	-1.76%	3.64 (3.62 - 3.66)	3.55 (3.53 - 3.57)	-2.19%	1.48 (1.47 - 1.49)	1.48 (1.47 - 1.49)	-0.26%
Starch from refined grains	5.82 (5.79 - 5.84)	5.77 (5.75 - 5.80)	-0.73%	3.60 (3.58 - 3.62)	3.58 (3.56 - 3.60)	-0.59%	1.50 (1.49 - 1.51)	1.47 (1.46 - 1.48)	-2.19%
Fiber*	5.88 (5.85 - 5.91)	5.70 (5.67 - 5.73)	-3.19%	3.65 (3.63 - 3.68)	3.53 (3.51 - 3.55)	-3.47%	1.49 (1.48 - 1.50)	1.47 (1.46 - 1.48)	-0.94%
**Fat**									
Total fat	5.70 (5.68 - 5.73)	5.88 (5.85 - 5.90)	2.95%	3.53 (3.51 - 3.55)	3.64 (3.62 - 3.66)	2.93%	1.45 (1.44 - 1.46)	1.52 (1.51 - 1.53)	4.36%
Fat from plant sources	5.82 (5.79 - 5.84)	5.75 (5.73 - 5.78)	-0.91%	3.61 (3.59 - 3.63)	3.56 (3.54 - 3.58)	-1.18%	1.48 (1.48 - 1.49)	1.48 (1.47 - 1.49)	-0.18%
Fat from animal sources	5.70 (5.67 - 5.73)	5.91 (5.89 - 5.94)	3.45%	3.53 (3.51 - 3.55)	3.67 (3.65 - 3.69)	3.77%	1.46 (1.45 - 1.47)	1.52 (1.51 - 1.53)	4.18%
Fat from dairy	5.75 (5.73 - 5.78)	5.85 (5.82 - 5.88)	1.54%	3.56 (3.54 - 3.58)	3.63 (3.61 - 3.65)	1.74%	1.47 (1.46 - 1.47)	1.51 (1.50 - 1.52)	2.71%
Fat from non-dairy animal sources	5.71 (5.69 - 5.74)	5.88 (5.85 - 5.90)	2.82%	3.54 (3.52 - 3.56)	3.65 (3.63 - 3.67)	2.99%	1.46 (1.45 - 1.47)	1.50 (1.49 - 1.51)	2.63%
Dietary cholesterol	5.71 (5.68 - 5.74)	5.83 (5.81 - 5.86)	1.82%	3.54 (3.52 - 3.56)	3.61 (3.59 - 3.63)	1.70%	1.46 (1.45 - 1.47)	1.50 (1.50 - 1.51)	2.86%
Trans fatty acids	5.73 (5.70 - 5.76)	5.87 (5.84 - 5.90)	2.02%	3.56 (3.53 - 3.58)	3.64 (3.62 - 3.67)	2.20%	1.46 (1.45 - 1.47)	1.50 (1.49 - 1.51)	2.81%
SFA	5.68 (5.66 - 5.71)	5.92 (5.89 - 5.94)	3.83%	3.51 (3.49 - 3.53)	3.68 (3.66 - 3.70)	4.65%	1.46 (1.46 - 1.47)	1.52 (1.51 - 1.52)	3.25%
MUFA	5.71 (5.68 - 5.74)	5.84 (5.82 - 5.87)	2.33%	3.54 (3.52 - 3.56)	3.62 (3.60 - 3.64)	2.11%	1.45 (1.44 - 1.46)	1.51 (1.50 - 1.52)	3.72%
PUFA	5.80 (5.77 - 5.82)	5.75 (5.73 - 5.78)	-0.54%	3.61 (3.59 - 3.63)	3.55 (3.53 - 3.57)	-1.33%	1.46 (1.45 - 1.47)	1.49 (1.49 - 1.50)	2.23%
n-3 fatty acids	5.78 (5.75 - 5.80)	5.83 (5.80 - 5.85)	0.93%	3.59 (3.57 - 3.61)	3.60 (3.58 - 3.62)	0.34%	1.46 (1.45 - 1.46)	1.52 (1.51 - 1.53)	4.14%
n-6 fatty acids	5.81 (5.78 - 5.83)	5.75 (5.72 - 5.77)	-0.89%	3.61 (3.59 - 3.63)	3.55 (3.53 - 3.57)	-1.66%	1.47 (1.46 - 1.47)	1.49 (1.48 - 1.50)	1.58%
**Protein**									
Total protein	5.77 (5.74 - 5.79)	5.80 (5.77 - 5.82)	0.38%	3.58 (3.56 - 3.60)	3.59 (3.57 - 3.61)	-0.11%	1.47 (1.46 - 1.48)	1.49 (1.48 - 1.50)	1.80%
Protein from plant sources	5.87 (5.84 - 5.89)	5.69 (5.67 - 5.72)	-2.87%	3.65 (3.62 - 3.67)	3.52 (3.50 - 3.54)	-3.35%	1.50 (1.49 - 1.51)	1.47 (1.46 - 1.48)	-2.18%
Protein from animal sources	5.73 (5.70 - 5.75)	5.82 (5.80 - 5.85)	1.47%	3.55 (3.53 - 3.57)	3.61 (3.59 - 3.63)	1.26%	1.46 (1.45 - 1.47)	1.50 (1.49 - 1.51)	2.52%
Protein from dairy	5.81 (5.78 - 5.84)	5.78 (5.76 - 5.81)	-0.59%	3.60 (3.58 - 3.62)	3.59 (3.57 - 3.61)	-0.76%	1.48 (1.47 - 1.49)	1.49 (1.48 - 1.50)	0.56%
Protein from non-dairy animal sources	5.72 (5.69 - 5.74)	5.82 (5.79 - 5.84)	1.50%	3.55 (3.53 - 3.57)	3.60 (3.58 - 3.62)	1.28%	1.46 (1.45 - 1.47)	1.49 (1.48 - 1.50)	2.20%

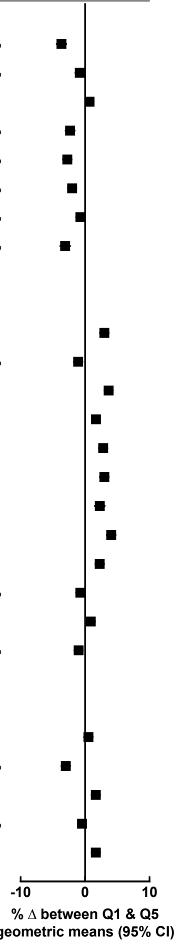

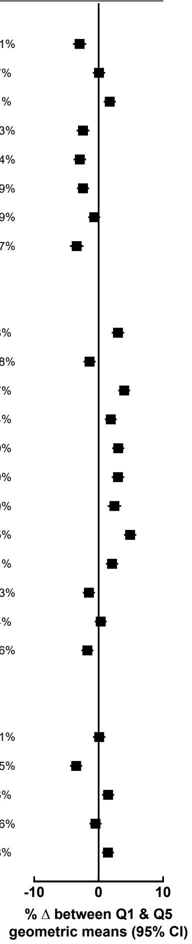

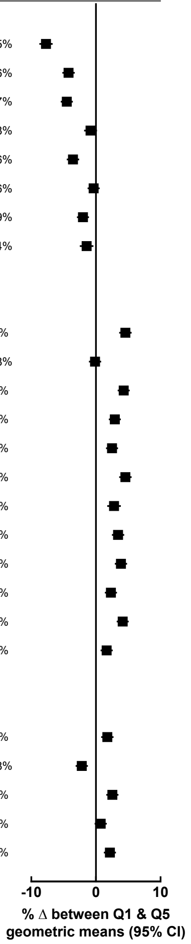

Supplemental Figure XIV. Geometric mean of total cholesterol (mmol/L), LDL-C (mmol/L) and HDL-C (mmol/L) by lowest (Q1) and highest (Q5) percentage intake of macronutrients **excluding participants with extreme levels of lipid markers** (n=24,408).

Models adjusted for age at recruitment (<45, 45-49, 50-54, 55-59, 60-64, ≥65), sex, ethnicity (white, mixed race, Asian or Asian British, black or black British, other, unknown), region (London, North-West England, North-Eastern England, Yorkshire & the Humber, West Midlands, East Midlands, South-West England, Wales), Townsend deprivation index (quintiles from least to most affluent, unknown), smoking status (never, previous, <15 cigarettes/day, 15-29 cigarettes/day, ≥30 cigarettes/day, unknown), physical activity (low, medium or high according to MET hours per week, unknown), alcohol (<1, 1-9, 10-19, 20-29, ≥30 g/day, unknown), body mass index (<20, 20-22.4, 22.5-24.9, 25-27.4, 27.5-29.9, 30-32.4, 32.5-34.9, 35-37.4, 37.5-39.9 or ≥40 in kg/m2), height (sex-specific groups in 5cm increments, unknown), diabetes diagnosed by a doctor (yes, no, unknown) and mean daily energy intake (quintiles).

*For fiber Q1 and Q5 represent quintiles of total fiber intake (g/day).

Abbreviations: CI = confidence interval; g/day = grams per day; HDL-C = high-density lipoprotein cholesterol; LDL-C= low-density lipoprotein cholesterol; MUFA = monounsaturated fatty acids; n-3 = omega-3; n-6 = omega-6; PUFA = polyunsaturated fatty acids; Q = quintile; SFA = saturated fatty acids.

Nutrients	Triglycerides (mmol/L) Geometric mean in Q1 (95% CI)	Geometric mean in Q5 (95% CI)	% Δ between Q1 & Q5 geometric means	TC-to-HDL-C ratio Geometric mean in Q1 (95% CI)	Geometric mean in Q5 (95% CI)	% Δ between Q1 & Q5 geometric means	TG-to-HDL-C ratio Geometric mean in Q1 (95% CI)	Geometric mean in Q5 (95% CI)	% Δ between Q1 & Q5 geometric means
**Carbohydrate**									
Total carbohydrates	1.31 (1.30 - 1.33)	1.44 (1.43 - 1.46)	9.80%	3.81 (3.78 - 3.83)	3.97 (3.94 - 3.99)	3.92%	0.85 (0.83 - 0.86)	1.00 (0.99 - 1.02)	17.25%
Total sugars	1.35 (1.34 - 1.37)	1.42 (1.41 - 1.44)	5.49%	3.85 (3.82 - 3.87)	3.98 (3.96 - 4.01)	3.14%	0.89 (0.88 - 0.90)	0.98 (0.96 - 0.99)	9.35%
Free sugars	1.32 (1.31 - 1.34)	1.47 (1.45 - 1.48)	10.49%	3.82 (3.80 - 3.84)	4.02 (3.99 - 4.04)	4.85%	0.87 (0.86 - 0.88)	1.01 (0.99 - 1.03)	14.75%
Non-free sugars	1.42 (1.40 - 1.44)	1.35 (1.33 - 1.36)	-5.33%	3.94 (3.91 - 3.97)	3.89 (3.86 - 3.91)	-1.48%	0.95 (0.94 - 0.97)	0.91 (0.89 - 0.92)	-4.75%
Total starch	1.34 (1.32 - 1.35)	1.41 (1.39 - 1.43)	5.35%	3.87 (3.85 - 3.90)	3.91 (3.88 - 3.93)	1.13%	0.88 (0.87 - 0.89)	0.96 (0.95 - 0.98)	9.01%
Starch from wholegrains	1.40 (1.38 - 1.41)	1.39 (1.37 - 1.40)	-0.94%	3.95 (3.92 - 3.97)	3.89 (3.86 - 3.91)	-1.50%	0.94 (0.93 - 0.96)	0.94 (0.92 - 0.95)	-0.69%
Starch from refined grains	1.36 (1.35 - 1.38)	1.41 (1.40 - 1.43)	3.66%	3.87 (3.85 - 3.90)	3.93 (3.90 - 3.95)	1.46%	0.91 (0.89 - 0.92)	0.96 (0.95 - 0.97)	5.85%
Fiber*	1.39 (1.38 - 1.41)	1.37 (1.35 - 1.38)	-4.09%	3.94 (3.91 - 3.97)	3.88 (3.86 - 3.91)	-2.25%	0.93 (0.92 - 0.95)	0.93 (0.91 - 0.94)	-3.16%
**Fat**									
Total fat	1.41 (1.39 - 1.43)	1.34 (1.33 - 1.36)	-5.13%	3.92 (3.90 - 3.95)	3.87 (3.84 - 3.89)	-1.41%	0.97 (0.95 - 0.98)	0.88 (0.87 - 0.89)	-9.49%
Fat from plant sources	1.38 (1.36 - 1.40)	1.37 (1.36 - 1.39)	-0.88%	3.91 (3.89 - 3.94)	3.88 (3.86 - 3.91)	-0.73%	0.93 (0.91 - 0.94)	0.92 (0.91 - 0.94)	-0.70%
Fat from animal sources	1.40 (1.39 - 1.42)	1.34 (1.33 - 1.36)	-4.63%	3.92 (3.89 - 3.94)	3.89 (3.86 - 3.91)	-0.73%	0.96 (0.95 - 0.98)	0.88 (0.87 - 0.89)	-8.81%
Fat from dairy	1.39 (1.38 - 1.41)	1.35 (1.33 - 1.37)	-3.35%	3.93 (3.90 - 3.95)	3.88 (3.85 - 3.90)	-1.16%	0.95 (0.93 - 0.96)	0.89 (0.88 - 0.91)	-6.06%
Fat from non-dairy animal sources	1.40 (1.39 - 1.42)	1.37 (1.35 - 1.38)	-2.42%	3.91 (3.88 - 3.93)	3.92 (3.89 - 3.94)	0.18%	0.95 (0.94 - 0.97)	0.91 (0.90 - 0.92)	-5.05%
Dietary cholesterol	1.40 (1.38 - 1.41)	1.36 (1.34 - 1.37)	-3.27%	3.91 (3.89 - 3.94)	3.88 (3.85 - 3.90)	-1.04%	0.96 (0.94 - 0.97)	0.90 (0.88 - 0.91)	-6.13%
Trans fatty acids	1.39 (1.37 - 1.41)	1.38 (1.36 - 1.40)	-0.96%	3.92 (3.90 - 3.95)	3.90 (3.88 - 3.93)	-0.80%	0.95 (0.93 - 0.96)	0.92 (0.90 - 0.93)	-3.77%
SFA	1.38 (1.37 - 1.40)	1.35 (1.34 - 1.37)	-2.55%	3.88 (3.86 - 3.91)	3.90 (3.88 - 3.93)	0.58%	0.94 (0.93 - 0.95)	0.89 (0.88 - 0.90)	-5.81%
MUFA	1.41 (1.39 - 1.43)	1.35 (1.33 - 1.36)	-4.31%	3.93 (3.90 - 3.95)	3.87 (3.85 - 3.89)	-1.39%	0.96 (0.95 - 0.98)	0.89 (0.88 - 0.91)	-8.03%
PUFA	1.42 (1.41 - 1.44)	1.35 (1.33 - 1.36)	-6.29%	3.97 (3.94 - 3.99)	3.85 (3.83 - 3.87)	-2.77%	0.97 (0.96 - 0.99)	0.90 (0.88 - 0.91)	-8.51%
n-3 fatty acids	1.44 (1.43 - 1.46)	1.31 (1.30 - 1.33)	-9.90%	3.97 (3.94 - 3.99)	3.84 (3.82 - 3.86)	-3.20%	0.99 (0.97 - 1.00)	0.86 (0.85 - 0.87)	-14.04%
n-6 fatty acids	1.41 (1.39 - 1.42)	1.35 (1.34 - 1.37)	-4.29%	3.96 (3.93 - 3.98)	3.86 (3.83 - 3.88)	-2.47%	0.96 (0.94 - 0.97)	0.91 (0.89 - 0.92)	-5.87%
**Protein**									
Total protein	1.41 (1.40 - 1.43)	1.34 (1.32 - 1.36)	-5.07%	3.93 (3.91 - 3.96)	3.88 (3.86 - 3.90)	-1.42%	0.96 (0.95 - 0.97)	0.90 (0.88 - 0.91)	-6.87%
Protein from plant sources	1.37 (1.35 - 1.38)	1.39 (1.37 - 1.40)	1.02%	3.91 (3.89 - 3.94)	3.89 (3.86 - 3.91)	-0.69%	0.91 (0.90 - 0.92)	0.94 (0.93 - 0.96)	3.20%
Protein from animal sources	1.41 (1.39 - 1.43)	1.35 (1.33 - 1.36)	-4.62%	3.92 (3.90 - 3.94)	3.88 (3.86 - 3.90)	-1.05%	0.96 (0.95 - 0.98)	0.90 (0.88 - 0.91)	-7.14%
Protein from dairy	1.39 (1.38 - 1.41)	1.37 (1.35 - 1.38)	-1.85%	3.94 (3.91 - 3.96)	3.88 (3.86 - 3.91)	-1.16%	0.94 (0.93 - 0.96)	0.92 (0.90 - 0.93)	-2.42%
Protein from non-dairy animal sources	1.41 (1.40 - 1.43)	1.36 (1.34 - 1.37)	-3.75%	3.91 (3.89 - 3.94)	3.89 (3.87 - 3.92)	-0.70%	0.96 (0.95 - 0.98)	0.91 (0.89 - 0.92)	-5.95%

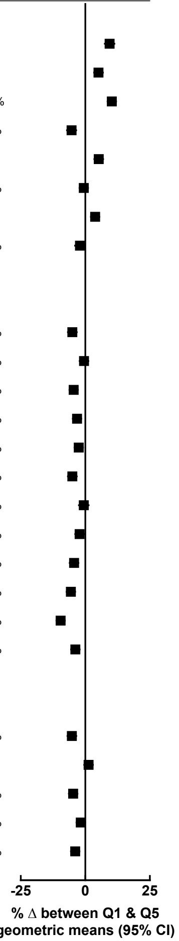

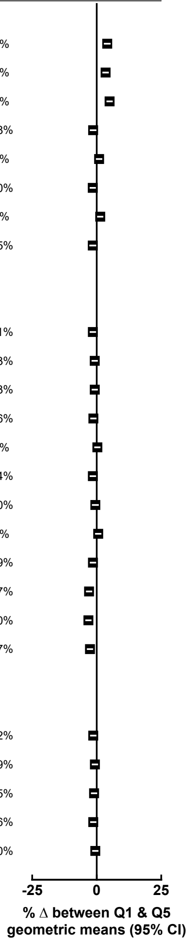

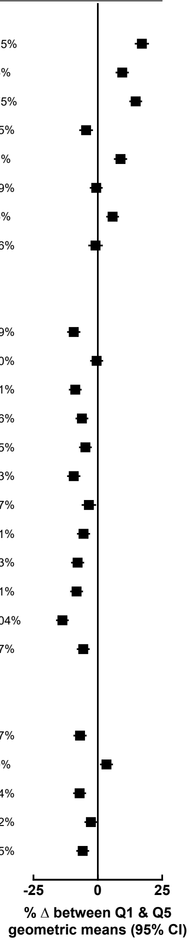

Supplemental Figure XV. Geometric mean of triglycerides (mmol/L), TC-to-HDL-C ratio and TG-to-HDL-C ratio by lowest (Q1) and highest (Q5) percentage intake of macronutrients **excluding participants with extreme levels of lipid markers** (n=24,408).

Models adjusted for age at recruitment (<45, 45-49, 50-54, 55-59, 60-64, ≥65), sex, ethnicity (white, mixed race, Asian or Asian British, black or black British, other, unknown), region (London, North-West England, North-Eastern England, Yorkshire & the Humber, West Midlands, East Midlands, South-West England, Wales), Townsend deprivation index (quintiles from least to most affluent, unknown), smoking status (never, previous, <15 cigarettes/day, 15-29 cigarettes/day, ≥30 cigarettes/day, unknown), physical activity (low, medium or high according to MET hours per week, unknown), alcohol (<1, 1-9, 10-19, 20-29, ≥30 g/day, unknown), body mass index (<20, 20-22.4, 22.5-24.9, 25-27.4, 27.5-29.9, 30-32.4, 32.5-34.9, 35-37.4, 37.5-39.9 or ≥40 in kg/m2), height (sex-specific groups in 5cm increments, unknown), diabetes diagnosed by a doctor (yes, no, unknown) and mean daily energy intake (quintiles).

*For fiber Q1 and Q5 represent quintiles of total fiber intake (g/day).

Abbreviations: CI = confidence interval; g/day = grams per day; HDL-C = high-density lipoprotein cholesterol; MUFA = monounsaturated fatty acids; n-3 = omega-3; n-6 = omega-6; PUFA = polyunsaturated fatty acids; Q = quintile; SFA = saturated fatty acids; TC = total cholesterol; TG = triglycerides.

Nutrients	ApoB (mmol/L) Geometric mean in Q1 (95% CI)	ApoB Geometric mean in Q5 (95% CI)	ApoB % Δ between Q1 & Q5 geometric means	ApoA1 (mmol/L) Geometric mean in Q1 (95% CI)	ApoA1 Geometric mean in Q5 (95% CI)	ApoA1 % Δ between Q1 & Q5 geometric means	ApoB-to-ApoA1 ratio Geometric mean in Q1 (95% CI)	ApoB-to-ApoA1 ratio Geometric mean in Q5 (95% CI)	ApoB-to-ApoA1 ratio % Δ between Q1 & Q5 geometric means
**Carbohydrate**									
Total carbohydrates	1.04 (1.04 - 1.05)	1.02 (1.01 - 1.03)	-2.35%	1.61 (1.60 - 1.62)	1.52 (1.51 - 1.53)	-5.83%	0.65 (0.64 - 0.65)	0.67 (0.67 - 0.68)	3.48%
Total sugars	1.03 (1.03 - 1.04)	1.04 (1.03 - 1.04)	0.50%	1.59 (1.58 - 1.59)	1.53 (1.53 - 1.54)	-3.34%	0.65 (0.65 - 0.65)	0.68 (0.67 - 0.68)	3.84%
Free sugars	1.02 (1.02 - 1.03)	1.04 (1.04 - 1.05)	1.82%	1.58 (1.57 - 1.58)	1.54 (1.53 - 1.55)	-2.21%	0.65 (0.65 - 0.65)	0.68 (0.67 - 0.68)	4.03%
Non-free sugars	1.04 (1.04 - 1.05)	1.03 (1.02 - 1.03)	-1.66%	1.58 (1.57 - 1.58)	1.54 (1.54 - 1.55)	-2.23%	0.66 (0.66 - 0.67)	0.66 (0.66 - 0.67)	0.58%
Total starch	1.04 (1.04 - 1.05)	1.02 (1.01 - 1.02)	-2.70%	1.58 (1.57 - 1.59)	1.54 (1.54 - 1.55)	-2.40%	0.66 (0.66 - 0.67)	0.66 (0.65 - 0.66)	-0.30%
Starch from wholegrains	1.04 (1.04 - 1.05)	1.02 (1.02 - 1.03)	-2.12%	1.57 (1.56 - 1.57)	1.55 (1.54 - 1.56)	-1.04%	0.67 (0.66 - 0.67)	0.66 (0.65 - 0.66)	-1.07%
Starch from refined grains	1.03 (1.03 - 1.04)	1.03 (1.02 - 1.03)	-0.58%	1.57 (1.56 - 1.58)	1.56 (1.55 - 1.56)	-0.91%	0.66 (0.65 - 0.66)	0.66 (0.66 - 0.67)	0.33%
Fiber*	1.05 (1.04 - 1.05)	1.02 (1.01 - 1.03)	-2.67%	1.58 (1.57 - 1.58)	1.54 (1.53 - 1.55)	-2.59%	0.66 (0.66 - 0.67)	0.66 (0.66 - 0.67)	-0.07%
**Fat**									
Total fat	1.02 (1.02 - 1.03)	1.04 (1.03 - 1.05)	1.82%	1.53 (1.52 - 1.53)	1.59 (1.59 - 1.60)	4.21%	0.67 (0.66 - 0.67)	0.65 (0.65 - 0.66)	-2.39%
Fat from plant sources	1.04 (1.03 - 1.04)	1.03 (1.02 - 1.03)	-0.64%	1.56 (1.55 - 1.56)	1.56 (1.56 - 1.57)	0.37%	0.66 (0.66 - 0.67)	0.66 (0.65 - 0.66)	-1.02%
Fat from animal sources	1.02 (1.01 - 1.03)	1.05 (1.04 - 1.05)	2.41%	1.54 (1.53 - 1.54)	1.59 (1.58 - 1.60)	3.45%	0.66 (0.66 - 0.67)	0.66 (0.65 - 0.66)	-1.04%
Fat from dairy	1.03 (1.03 - 1.04)	1.03 (1.03 - 1.04)	-0.08%	1.54 (1.54 - 1.55)	1.58 (1.57 - 1.59)	2.34%	0.67 (0.66 - 0.67)	0.65 (0.65 - 0.66)	-2.43%
Fat from non-dairy animal sources	1.02 (1.01 - 1.02)	1.05 (1.04 - 1.05)	2.59%	1.54 (1.54 - 1.55)	1.58 (1.57 - 1.58)	2.14%	0.66 (0.66 - 0.66)	0.66 (0.66 - 0.67)	0.45%
Dietary cholesterol	1.02 (1.01 - 1.02)	1.04 (1.03 - 1.04)	1.66%	1.54 (1.54 - 1.55)	1.57 (1.57 - 1.58)	2.05%	0.66 (0.66 - 0.67)	0.66 (0.65 - 0.66)	-0.39%
Trans fatty acids	1.03 (1.02 - 1.04)	1.04 (1.03 - 1.04)	0.59%	1.54 (1.53 - 1.54)	1.58 (1.57 - 1.59)	2.75%	0.67 (0.66 - 0.68)	0.66 (0.65 - 0.66)	-2.16%
SFA	1.02 (1.01 - 1.02)	1.04 (1.04 - 1.05)	2.49%	1.54 (1.53 - 1.54)	1.59 (1.58 - 1.59)	3.18%	0.66 (0.66 - 0.67)	0.66 (0.65 - 0.66)	-0.69%
MUFA	1.02 (1.02 - 1.03)	1.04 (1.03 - 1.04)	1.72%	1.53 (1.52 - 1.54)	1.59 (1.58 - 1.59)	3.68%	0.67 (0.66 - 0.67)	0.65 (0.65 - 0.66)	-1.96%
PUFA	1.03 (1.03 - 1.04)	1.03 (1.02 - 1.03)	-0.62%	1.55 (1.54 - 1.55)	1.57 (1.56 - 1.58)	1.57%	0.67 (0.66 - 0.67)	0.65 (0.65 - 0.66)	-2.19%
n-3 fatty acids	1.03 (1.02 - 1.03)	1.04 (1.04 - 1.05)	1.20%	1.55 (1.54 - 1.55)	1.58 (1.57 - 1.58)	1.82%	0.67 (0.66 - 0.67)	0.66 (0.66 - 0.67)	-0.62%
n-6 fatty acids	1.04 (1.03 - 1.04)	1.02 (1.02 - 1.03)	-1.17%	1.55 (1.54 - 1.55)	1.57 (1.56 - 1.57)	1.46%	0.67 (0.66 - 0.67)	0.65 (0.65 - 0.66)	-2.63%
**Protein**									
Total protein	1.03 (1.02 - 1.03)	1.03 (1.03 - 1.04)	0.44%	1.55 (1.55 - 1.56)	1.56 (1.56 - 1.57)	0.74%	0.66 (0.66 - 0.67)	0.66 (0.66 - 0.67)	-0.30%
Protein from plant sources	1.04 (1.04 - 1.05)	1.02 (1.01 - 1.02)	-2.40%	1.57 (1.57 - 1.58)	1.54 (1.54 - 1.55)	-2.07%	0.66 (0.66 - 0.67)	0.66 (0.65 - 0.66)	-0.34%
Protein from animal sources	1.02 (1.02 - 1.03)	1.04 (1.03 - 1.04)	1.39%	1.55 (1.54 - 1.55)	1.57 (1.56 - 1.58)	1.51%	0.66 (0.66 - 0.67)	0.66 (0.66 - 0.67)	-0.12%
Protein from dairy	1.04 (1.04 - 1.05)	1.03 (1.02 - 1.03)	-1.69%	1.56 (1.55 - 1.56)	1.56 (1.55 - 1.57)	0.05%	0.67 (0.66 - 0.67)	0.66 (0.65 - 0.66)	-1.73%
Protein from non-dairy animal sources	1.02 (1.01 - 1.02)	1.04 (1.03 - 1.04)	1.80%	1.54 (1.54 - 1.55)	1.57 (1.56 - 1.57)	1.41%	0.66 (0.65 - 0.66)	0.66 (0.66 - 0.67)	0.39%

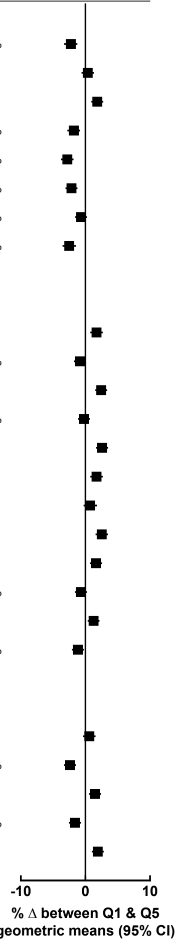

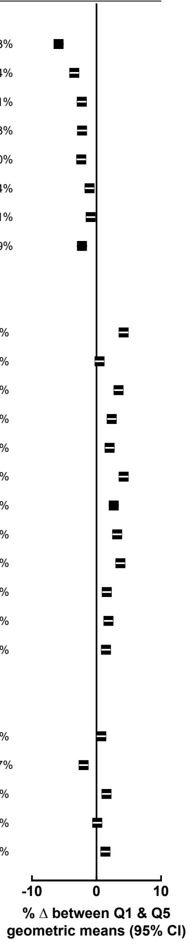

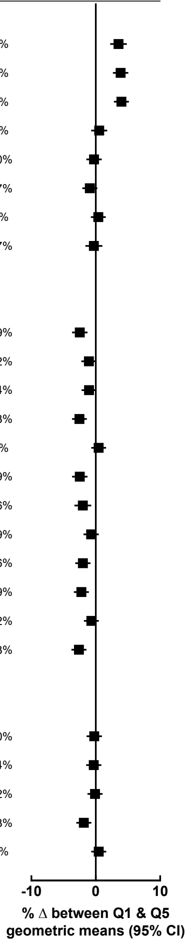

Supplemental Figure XVI. Geometric mean of ApoB (mmol/L), ApoA1 (mmol/L) and ApoB-to-ApoA1 ratio by lowest (Q1) and highest (Q5) percentage intake of macronutrients **excluding participants with extreme levels of lipid markers** (n=24,408).

Models adjusted for age at recruitment (<45, 45-49, 50-54, 55-59, 60-64, ≥65), sex, ethnicity (white, mixed race, Asian or Asian British, black or black British, other, unknown), region (London, North-West England, North-Eastern England, Yorkshire & the Humber, West Midlands, East Midlands, South-West England, Wales), Townsend deprivation index (quintiles from least to most affluent, unknown), smoking status (never, previous, <15 cigarettes/day, 15-29 cigarettes/day, ≥30 cigarettes/day, unknown), physical activity (low, medium or high according to MET hours per week, unknown), alcohol (<1, 1-9, 10-19, 20-29, ≥30 g/day, unknown), body mass index (<20, 20-22.4, 22.5-24.9, 25-27.4, 27.5-29.9, 30-32.4, 32.5-34.9, 35-37.4, 37.5-39.9 or ≥40 in kg/m2), height (sex-specific groups in 5cm increments, unknown), diabetes diagnosed by a doctor (yes, no, unknown) and mean daily energy intake (quintiles).

*For fiber Q1 and Q5 represent quintiles of total fiber intake (g/day).

Abbreviations: Apo = apolipoprotein; CI = confidence interval; g/day = grams per day; MUFA = monounsaturated fatty acids; n-3 = omega-3; n-6 = omega-6; PUFA = polyunsaturated fatty acids; Q = quintile; SFA = saturated fatty acids.

Nutrients	Total cholesterol (mmol/L): Geometric mean in Q1 (95% CI)	Total cholesterol (mmol/L): Geometric mean in Q5 (95% CI)	Total cholesterol (mmol/L): % Δ between Q1 & Q5 geometric means	LDL-C (mmol/L): Geometric mean in Q1 (95% CI)	LDL-C (mmol/L): Geometric mean in Q5 (95% CI)	LDL-C (mmol/L): % Δ between Q1 & Q5 geometric means	HDL-C (mmol/L): Geometric mean in Q1 (95% CI)	HDL-C (mmol/L): Geometric mean in Q5 (95% CI)	HDL-C (mmol/L): % Δ between Q1 & Q5 geometric means
**Carbohydrate**									
Total carbohydrates	5.91 (5.88 - 5.95)	5.68 (5.65 - 5.72)	-3.99%	3.65 (3.62 - 3.68)	3.55 (3.52 - 3.58)	-2.77%	1.56 (1.55 - 1.57)	1.41 (1.40 - 1.42)	-10.05%
Total sugars	5.83 (5.80 - 5.86)	5.76 (5.73 - 5.79)	-1.25%	3.61 (3.59 - 3.63)	3.60 (3.58 - 3.62)	-0.28%	1.51 (1.50 - 1.52)	1.44 (1.43 - 1.45)	-4.54%
Free sugars	5.78 (5.75 - 5.81)	5.80 (5.77 - 5.83)	0.34%	3.57 (3.55 - 3.60)	3.62 (3.60 - 3.65)	1.42%	1.51 (1.50 - 1.52)	1.44 (1.43 - 1.45)	-4.83%
Non-free sugars	5.87 (5.84 - 5.90)	5.75 (5.72 - 5.78)	-2.06%	3.65 (3.63 - 3.68)	3.57 (3.55 - 3.60)	-2.19%	1.48 (1.47 - 1.49)	1.47 (1.46 - 1.48)	-0.63%
Total starch	5.89 (5.86 - 5.92)	5.71 (5.68 - 5.74)	-3.16%	3.66 (3.63 - 3.68)	3.54 (3.52 - 3.57)	-3.17%	1.52 (1.51 - 1.53)	1.45 (1.44 - 1.46)	-4.65%
Starch from wholegrains	5.84 (5.81 - 5.87)	5.75 (5.72 - 5.78)	-1.59%	3.64 (3.62 - 3.66)	3.57 (3.55 - 3.59)	-2.00%	1.48 (1.47 - 1.48)	1.48 (1.47 - 1.49)	0.02%
Starch from refined grains	5.81 (5.79 - 5.84)	5.76 (5.74 - 5.79)	-0.85%	3.60 (3.58 - 3.63)	3.58 (3.56 - 3.60)	-0.72%	1.50 (1.49 - 1.51)	1.46 (1.45 - 1.47)	-2.45%
Fiber	5.87 (5.84 - 5.90)	5.71 (5.68 - 5.74)	-2.82%	3.66 (3.63 - 3.68)	3.54 (3.52 - 3.57)	-3.21%	1.48 (1.48 - 1.49)	1.47 (1.46 - 1.47)	-1.28%
**Fat**									
Total fat	5.68 (5.64 - 5.72)	5.90 (5.86 - 5.94)	3.75%	3.53 (3.50 - 3.55)	3.67 (3.64 - 3.70)	3.91%	1.44 (1.43 - 1.45)	1.52 (1.51 - 1.53)	5.86%
Fat from plant sources	5.82 (5.79 - 5.85)	5.76 (5.73 - 5.79)	-1.03%	3.62 (3.60 - 3.64)	3.57 (3.54 - 3.59)	-1.45%	1.48 (1.47 - 1.49)	1.48 (1.47 - 1.49)	0.20%
Fat from animal sources	5.67 (5.64 - 5.70)	5.92 (5.89 - 5.95)	4.33%	3.52 (3.49 - 3.54)	3.69 (3.66 - 3.71)	4.78%	1.44 (1.43 - 1.45)	1.51 (1.51 - 1.52)	4.95%
Fat from dairy	5.74 (5.72 - 5.77)	5.86 (5.83 - 5.88)	1.94%	3.57 (3.54 - 3.59)	3.65 (3.63 - 3.67)	2.30%	1.46 (1.45 - 1.47)	1.50 (1.49 - 1.51)	2.63%
Fat from non-dairy animal sources	5.71 (5.68 - 5.74)	5.88 (5.85 - 5.91)	2.98%	3.54 (3.52 - 3.57)	3.66 (3.63 - 3.68)	3.12%	1.46 (1.45 - 1.46)	1.50 (1.50 - 1.51)	3.32%
Dietary cholesterol	5.73 (5.70 - 5.76)	5.83 (5.80 - 5.86)	1.66%	3.56 (3.54 - 3.59)	3.62 (3.59 - 3.64)	1.42%	1.45 (1.45 - 1.46)	1.50 (1.49 - 1.51)	3.01%
Trans fatty acids	5.71 (5.68 - 5.74)	5.86 (5.83 - 5.89)	2.49%	3.54 (3.52 - 3.56)	3.65 (3.63 - 3.67)	2.99%	1.46 (1.45 - 1.47)	1.50 (1.49 - 1.51)	2.58%
SFA	5.66 (5.63 - 5.69)	5.92 (5.89 - 5.96)	4.47%	3.50 (3.48 - 3.53)	3.70 (3.67 - 3.73)	5.45%	1.45 (1.44 - 1.46)	1.51 (1.50 - 1.52)	4.25%
MUFA	5.72 (5.68 - 5.75)	5.87 (5.84 - 5.91)	2.70%	3.55 (3.53 - 3.58)	3.64 (3.62 - 3.67)	2.51%	1.45 (1.44 - 1.46)	1.51 (1.50 - 1.52)	4.46%
PUFA	5.81 (5.78 - 5.84)	5.76 (5.73 - 5.79)	-0.88%	3.63 (3.60 - 3.65)	3.56 (3.54 - 3.59)	-1.77%	1.46 (1.45 - 1.47)	1.50 (1.49 - 1.51)	2.60%
n-3 fatty acids	5.77 (5.74 - 5.80)	5.81 (5.78 - 5.83)	0.66%	3.60 (3.57 - 3.62)	3.60 (3.57 - 3.62)	0.01%	1.45 (1.44 - 1.46)	1.51 (1.50 - 1.52)	4.05%
n-6 fatty acids	5.81 (5.78 - 5.85)	5.76 (5.73 - 5.79)	-0.98%	3.63 (3.60 - 3.65)	3.56 (3.54 - 3.59)	-1.82%	1.46 (1.45 - 1.47)	1.49 (1.48 - 1.50)	1.90%
**Protein**									
Total protein	5.78 (5.75 - 5.81)	5.83 (5.80 - 5.86)	0.96%	3.60 (3.57 - 3.62)	3.62 (3.60 - 3.65)	0.73%	1.46 (1.45 - 1.47)	1.49 (1.48 - 1.50)	1.88%
Protein from plant sources	5.88 (5.85 - 5.91)	5.69 (5.66 - 5.72)	-3.27%	3.66 (3.64 - 3.69)	3.53 (3.50 - 3.55)	-3.77%	1.49 (1.48 - 1.50)	1.46 (1.45 - 1.46)	-2.62%
Protein from animal sources	5.72 (5.70 - 5.75)	5.84 (5.81 - 5.87)	1.95%	3.56 (3.53 - 3.58)	3.63 (3.60 - 3.65)	1.97%	1.46 (1.45 - 1.46)	1.49 (1.48 - 1.50)	2.26%
Protein from dairy	5.81 (5.78 - 5.83)	5.79 (5.77 - 5.82)	-0.21%	3.61 (3.59 - 3.63)	3.60 (3.58 - 3.62)	-0.17%	1.47 (1.47 - 1.48)	1.48 (1.47 - 1.49)	0.56%
Protein from non-dairy animal sources	5.72 (5.69 - 5.75)	5.83 (5.80 - 5.86)	1.90%	3.56 (3.53 - 3.58)	3.62 (3.60 - 3.64)	1.82%	1.46 (1.45 - 1.47)	1.49 (1.48 - 1.50)	2.00%

-10 0 10 % Δ between Q1 & Q5 geometric means (95% CI)

Supplemental Figure XVII. Geometric mean of total cholesterol (mmol/L), LDL-C (mmol/L) and HDL-C (mmol/L) by lowest (Q1) and highest (Q5) intake of **macronutrients in grams per day** (n=24,639).

Models adjusted for age at recruitment (<45, 45-49, 50-54, 55-59, 60-64, ≥65), sex, ethnicity (white, mixed race, Asian or Asian British, black or black British, other, unknown), region (London, North-West England, North-Eastern England, Yorkshire & the

Humber, West Midlands, East Midlands, South-West England, Wales), Townsend deprivation index (quintiles from least to most affluent, unknown), smoking status (never, previous, <15 cigarettes/day, 15-29 cigarettes/day, ≥30 cigarettes/day, unknown), physical activity (low, medium or high according to MET hours per week, unknown), alcohol (<1, 1-9, 10-19, 20-29, ≥30 g/day, unknown), body mass index (<20, 20-22.4, 22.5-24.9, 25-27.4, 27.5-29.9, 30-32.4, 32.5-34.9, 35-37.4, 37.5-39.9 or ≥40 in kg/m2), height (sex-specific groups in 5cm increments, unknown), diabetes diagnosed by a doctor (yes, no, unknown) and mean daily energy intake (quintiles).

Abbreviations: CI = confidence interval; g/day = grams per day; HDL-C = high-density lipoprotein cholesterol; LDL-C= low-density lipoprotein cholesterol; MUFA = monounsaturated fatty acids; n-3 = omega-3; n-6 = omega-6; PUFA = polyunsaturated fatty acids; Q = quintile; SFA = saturated fatty acids.

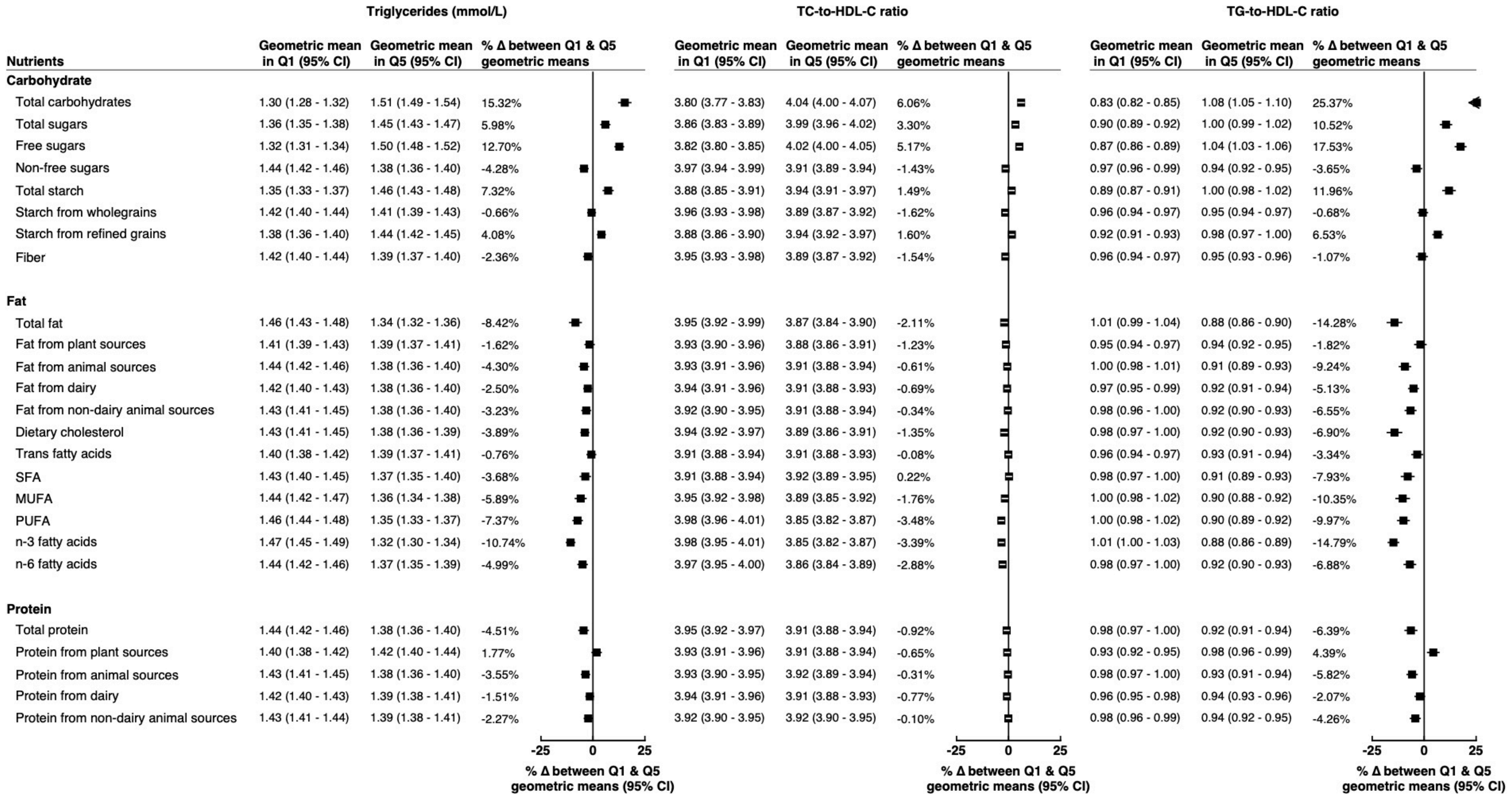

Triglycerides (mmol/L)

Nutrients	Geometric mean in Q1 (95% CI)	Geometric mean in Q5 (95% CI)	% Δ between Q1 & Q5 geometric means
**Carbohydrate**			
Total carbohydrates	1.30 (1.28 - 1.32)	1.51 (1.49 - 1.54)	15.32%
Total sugars	1.36 (1.35 - 1.38)	1.45 (1.43 - 1.47)	5.98%
Free sugars	1.32 (1.31 - 1.34)	1.50 (1.48 - 1.52)	12.70%
Non-free sugars	1.44 (1.42 - 1.46)	1.38 (1.36 - 1.40)	-4.28%
Total starch	1.35 (1.33 - 1.37)	1.46 (1.43 - 1.48)	7.32%
Starch from wholegrains	1.42 (1.40 - 1.44)	1.41 (1.39 - 1.43)	-0.66%
Starch from refined grains	1.38 (1.36 - 1.40)	1.44 (1.42 - 1.45)	4.08%
Fiber	1.42 (1.40 - 1.44)	1.39 (1.37 - 1.40)	-2.36%
**Fat**			
Total fat	1.46 (1.43 - 1.48)	1.34 (1.32 - 1.36)	-8.42%
Fat from plant sources	1.41 (1.39 - 1.43)	1.39 (1.37 - 1.41)	-1.62%
Fat from animal sources	1.44 (1.42 - 1.46)	1.38 (1.36 - 1.40)	-4.30%
Fat from dairy	1.42 (1.40 - 1.43)	1.38 (1.36 - 1.40)	-2.50%
Fat from non-dairy animal sources	1.43 (1.41 - 1.45)	1.38 (1.36 - 1.40)	-3.23%
Dietary cholesterol	1.43 (1.41 - 1.45)	1.38 (1.36 - 1.39)	-3.89%
Trans fatty acids	1.40 (1.38 - 1.42)	1.39 (1.37 - 1.41)	-0.76%
SFA	1.43 (1.40 - 1.45)	1.37 (1.35 - 1.40)	-3.68%
MUFA	1.44 (1.42 - 1.47)	1.36 (1.34 - 1.38)	-5.89%
PUFA	1.46 (1.44 - 1.48)	1.35 (1.33 - 1.37)	-7.37%
n-3 fatty acids	1.47 (1.45 - 1.49)	1.32 (1.30 - 1.34)	-10.74%
n-6 fatty acids	1.44 (1.42 - 1.46)	1.37 (1.35 - 1.39)	-4.99%
**Protein**			
Total protein	1.44 (1.42 - 1.46)	1.38 (1.36 - 1.40)	-4.51%
Protein from plant sources	1.40 (1.38 - 1.42)	1.42 (1.40 - 1.44)	1.77%
Protein from animal sources	1.43 (1.41 - 1.45)	1.38 (1.36 - 1.40)	-3.55%
Protein from dairy	1.42 (1.40 - 1.43)	1.39 (1.38 - 1.41)	-1.51%
Protein from non-dairy animal sources	1.43 (1.41 - 1.44)	1.39 (1.38 - 1.41)	-2.27%

TC-to-HDL-C ratio

Nutrients	Geometric mean in Q1 (95% CI)	Geometric mean in Q5 (95% CI)	% Δ between Q1 & Q5 geometric means
**Carbohydrate**			
Total carbohydrates	3.80 (3.77 - 3.83)	4.04 (4.00 - 4.07)	6.06%
Total sugars	3.86 (3.83 - 3.89)	3.99 (3.96 - 4.02)	3.30%
Free sugars	3.82 (3.80 - 3.85)	4.02 (4.00 - 4.05)	5.17%
Non-free sugars	3.97 (3.94 - 3.99)	3.91 (3.89 - 3.94)	-1.43%
Total starch	3.88 (3.85 - 3.91)	3.94 (3.91 - 3.97)	1.49%
Starch from wholegrains	3.96 (3.93 - 3.98)	3.89 (3.87 - 3.92)	-1.62%
Starch from refined grains	3.88 (3.86 - 3.90)	3.94 (3.92 - 3.97)	1.60%
Fiber	3.95 (3.93 - 3.98)	3.89 (3.87 - 3.92)	-1.54%
**Fat**			
Total fat	3.95 (3.92 - 3.99)	3.87 (3.84 - 3.90)	-2.11%
Fat from plant sources	3.93 (3.90 - 3.96)	3.88 (3.86 - 3.91)	-1.23%
Fat from animal sources	3.93 (3.91 - 3.96)	3.91 (3.88 - 3.94)	-0.61%
Fat from dairy	3.94 (3.91 - 3.96)	3.91 (3.88 - 3.93)	-0.69%
Fat from non-dairy animal sources	3.92 (3.90 - 3.95)	3.91 (3.88 - 3.94)	-0.34%
Dietary cholesterol	3.94 (3.92 - 3.97)	3.89 (3.86 - 3.91)	-1.35%
Trans fatty acids	3.91 (3.88 - 3.94)	3.91 (3.88 - 3.93)	-0.08%
SFA	3.91 (3.88 - 3.94)	3.92 (3.89 - 3.95)	0.22%
MUFA	3.95 (3.92 - 3.98)	3.89 (3.85 - 3.92)	-1.76%
PUFA	3.98 (3.96 - 4.01)	3.85 (3.82 - 3.87)	-3.48%
n-3 fatty acids	3.98 (3.95 - 4.01)	3.85 (3.82 - 3.87)	-3.39%
n-6 fatty acids	3.97 (3.95 - 4.00)	3.86 (3.84 - 3.89)	-2.88%
**Protein**			
Total protein	3.95 (3.92 - 3.97)	3.91 (3.88 - 3.94)	-0.92%
Protein from plant sources	3.93 (3.91 - 3.96)	3.91 (3.88 - 3.94)	-0.65%
Protein from animal sources	3.93 (3.90 - 3.95)	3.92 (3.89 - 3.94)	-0.31%
Protein from dairy	3.94 (3.91 - 3.96)	3.91 (3.88 - 3.93)	-0.77%
Protein from non-dairy animal sources	3.92 (3.90 - 3.95)	3.92 (3.90 - 3.95)	-0.10%

TG-to-HDL-C ratio

Nutrients	Geometric mean in Q1 (95% CI)	Geometric mean in Q5 (95% CI)	% Δ between Q1 & Q5 geometric means
**Carbohydrate**			
Total carbohydrates	0.83 (0.82 - 0.85)	1.08 (1.05 - 1.10)	25.37%
Total sugars	0.90 (0.89 - 0.92)	1.00 (0.99 - 1.02)	10.52%
Free sugars	0.87 (0.86 - 0.89)	1.04 (1.03 - 1.06)	17.53%
Non-free sugars	0.97 (0.96 - 0.99)	0.94 (0.92 - 0.95)	-3.65%
Total starch	0.89 (0.87 - 0.91)	1.00 (0.98 - 1.02)	11.96%
Starch from wholegrains	0.96 (0.94 - 0.97)	0.95 (0.94 - 0.97)	-0.68%
Starch from refined grains	0.92 (0.91 - 0.93)	0.98 (0.97 - 1.00)	6.53%
Fiber	0.96 (0.94 - 0.97)	0.95 (0.93 - 0.96)	-1.07%
**Fat**			
Total fat	1.01 (0.99 - 1.04)	0.88 (0.86 - 0.90)	-14.28%
Fat from plant sources	0.95 (0.94 - 0.97)	0.94 (0.92 - 0.95)	-1.82%
Fat from animal sources	1.00 (0.98 - 1.01)	0.91 (0.89 - 0.93)	-9.24%
Fat from dairy	0.97 (0.95 - 0.99)	0.92 (0.91 - 0.94)	-5.13%
Fat from non-dairy animal sources	0.98 (0.96 - 1.00)	0.92 (0.90 - 0.93)	-6.55%
Dietary cholesterol	0.98 (0.97 - 1.00)	0.92 (0.90 - 0.93)	-6.90%
Trans fatty acids	0.96 (0.94 - 0.97)	0.93 (0.91 - 0.94)	-3.34%
SFA	0.98 (0.97 - 1.00)	0.91 (0.89 - 0.93)	-7.93%
MUFA	1.00 (0.98 - 1.02)	0.90 (0.88 - 0.92)	-10.35%
PUFA	1.00 (0.98 - 1.02)	0.90 (0.89 - 0.92)	-9.97%
n-3 fatty acids	1.01 (1.00 - 1.03)	0.88 (0.86 - 0.89)	-14.79%
n-6 fatty acids	0.98 (0.97 - 1.00)	0.92 (0.90 - 0.93)	-6.88%
**Protein**			
Total protein	0.98 (0.97 - 1.00)	0.92 (0.91 - 0.94)	-6.39%
Protein from plant sources	0.93 (0.92 - 0.95)	0.98 (0.96 - 0.99)	4.39%
Protein from animal sources	0.98 (0.97 - 1.00)	0.93 (0.91 - 0.94)	-5.82%
Protein from dairy	0.96 (0.95 - 0.98)	0.94 (0.93 - 0.96)	-2.07%
Protein from non-dairy animal sources	0.98 (0.96 - 0.99)	0.94 (0.92 - 0.95)	-4.26%

Supplemental Figure XVIII. Geometric mean of triglycerides (mmol/L), TC-to-HDL-C ratio and TG-to-HDL-C ratio by lowest (Q1) and highest (Q5) intake of **macronutrients in grams per day** (n=24,639).

Models adjusted for age at recruitment (<45, 45-49, 50-54, 55-59, 60-64, ≥65), sex, ethnicity (white, mixed race, Asian or Asian British, black or black British, other, unknown), region (London, North-West England, North-Eastern England, Yorkshire & the Humber, West Midlands, East Midlands, South-West England, Wales), Townsend deprivation index (quintiles from least to most affluent, unknown), smoking status (never, previous, <15 cigarettes/day, 15-29 cigarettes/day, ≥30 cigarettes/day, unknown), physical activity (low, medium or high according to MET hours per week, unknown), alcohol (<1, 1-9, 10-19, 20-29, ≥30 g/day, unknown), body mass index (<20, 20-22.4, 22.5-24.9, 25-27.4, 27.5-29.9, 30-32.4, 32.5-34.9, 35-37.4, 37.5-39.9 or ≥40 in kg/m2), height (sex-specific groups in 5cm increments, unknown), diabetes diagnosed by a doctor (yes, no, unknown) and mean daily energy intake (quintiles).

Abbreviations: CI = confidence interval; g/day = grams per day; HDL-C = high-density lipoprotein cholesterol; MUFA = monounsaturated fatty acids; n-3 = omega-3; n-6 = omega-6; PUFA = polyunsaturated fatty acids; Q = quintile; SFA = saturated fatty acids; TC = total cholesterol; TG = triglycerides.

Nutrients	ApoB (mmol/L) Geometric mean in Q1 (95% CI)	ApoB (mmol/L) Geometric mean in Q5 (95% CI)	ApoB (mmol/L) % Δ between Q1 & Q5 geometric means	ApoA1 (mmol/L) Geometric mean in Q1 (95% CI)	ApoA1 (mmol/L) Geometric mean in Q5 (95% CI)	ApoA1 (mmol/L) % Δ between Q1 & Q5 geometric means	ApoB-to-ApoA1 ratio Geometric mean in Q1 (95% CI)	ApoB-to-ApoA1 ratio Geometric mean in Q5 (95% CI)	ApoB-to-ApoA1 ratio % Δ between Q1 & Q5 geometric means
**Carbohydrate**									
Total carbohydrates	1.04 (1.04 - 1.05)	1.02 (1.01 - 1.03)	-2.16%	1.62 (1.61 - 1.63)	1.50 (1.50 - 1.51)	-7.58%	0.64 (0.64 - 0.65)	0.68 (0.67 - 0.69)	5.42%
Total sugars	1.03 (1.03 - 1.04)	1.03 (1.03 - 1.04)	0.08%	1.59 (1.58 - 1.60)	1.53 (1.52 - 1.54)	-3.86%	0.65 (0.64 - 0.66)	0.68 (0.67 - 0.68)	3.94%
Free sugars	1.02 (1.02 - 1.03)	1.04 (1.03 - 1.05)	1.60%	1.58 (1.57 - 1.59)	1.54 (1.53 - 1.55)	-2.46%	0.65 (0.64 - 0.65)	0.68 (0.67 - 0.68)	4.06%
Non-free sugars	1.05 (1.04 - 1.05)	1.03 (1.02 - 1.03)	-1.93%	1.57 (1.57 - 1.58)	1.54 (1.54 - 1.55)	-1.96%	0.67 (0.66 - 0.67)	0.67 (0.66 - 0.67)	0.02%
Total starch	1.05 (1.04 - 1.06)	1.02 (1.01 - 1.02)	-3.13%	1.59 (1.58 - 1.60)	1.54 (1.53 - 1.55)	-2.93%	0.66 (0.65 - 0.67)	0.66 (0.65 - 0.66)	-0.20%
Starch from wholegrains	1.04 (1.04 - 1.05)	1.02 (1.02 - 1.03)	-1.88%	1.57 (1.56 - 1.57)	1.56 (1.55 - 1.56)	-0.77%	0.67 (0.66 - 0.67)	0.66 (0.65 - 0.66)	-1.11%
Starch from refined grains	1.03 (1.03 - 1.04)	1.03 (1.02 - 1.03)	-0.59%	1.57 (1.56 - 1.58)	1.55 (1.55 - 1.56)	-1.17%	0.66 (0.65 - 0.66)	0.66 (0.66 - 0.67)	0.58%
Fiber	1.05 (1.04 - 1.05)	1.02 (1.01 - 1.03)	-2.49%	1.58 (1.57 - 1.58)	1.54 (1.54 - 1.55)	-2.22%	0.66 (0.66 - 0.67)	0.66 (0.66 - 0.67)	-0.27%
**Fat**									
Total fat	1.02 (1.01 - 1.03)	1.04 (1.03 - 1.05)	2.23%	1.52 (1.51 - 1.53)	1.60 (1.59 - 1.61)	5.25%	0.67 (0.66 - 0.68)	0.65 (0.64 - 0.66)	-3.02%
Fat from plant sources	1.04 (1.03 - 1.04)	1.03 (1.02 - 1.03)	-0.97%	1.56 (1.55 - 1.56)	1.57 (1.56 - 1.58)	0.77%	0.67 (0.66 - 0.67)	0.65 (0.65 - 0.66)	-1.74%
Fat from animal sources	1.02 (1.01 - 1.02)	1.05 (1.04 - 1.05)	2.93%	1.53 (1.52 - 1.54)	1.59 (1.59 - 1.60)	4.08%	0.67 (0.66 - 0.67)	0.66 (0.65 - 0.66)	-1.15%
Fat from dairy	1.03 (1.02 - 1.04)	1.04 (1.03 - 1.04)	0.48%	1.54 (1.54 - 1.55)	1.58 (1.57 - 1.59)	2.34%	0.67 (0.66 - 0.67)	0.66 (0.65 - 0.66)	-1.86%
Fat from non-dairy animal sources	1.02 (1.01 - 1.02)	1.04 (1.04 - 1.05)	2.48%	1.54 (1.54 - 1.55)	1.58 (1.57 - 1.59)	2.49%	0.66 (0.65 - 0.67)	0.66 (0.65 - 0.67)	-0.01%
Dietary cholesterol	1.02 (1.02 - 1.03)	1.04 (1.03 - 1.04)	1.32%	1.54 (1.54 - 1.55)	1.58 (1.57 - 1.58)	2.24%	0.66 (0.66 - 0.67)	0.66 (0.65 - 0.66)	-0.92%
Trans fatty acids	1.02 (1.02 - 1.03)	1.04 (1.03 - 1.04)	1.24%	1.54 (1.53 - 1.55)	1.58 (1.57 - 1.59)	2.63%	0.66 (0.66 - 0.67)	0.66 (0.65 - 0.66)	-1.39%
SFA	1.02 (1.01 - 1.02)	1.05 (1.04 - 1.05)	2.79%	1.53 (1.52 - 1.54)	1.59 (1.58 - 1.60)	4.01%	0.66 (0.66 - 0.67)	0.66 (0.65 - 0.66)	-1.21%
MUFA	1.02 (1.01 - 1.03)	1.04 (1.03 - 1.05)	1.98%	1.53 (1.52 - 1.53)	1.59 (1.59 - 1.60)	4.32%	0.67 (0.66 - 0.68)	0.65 (0.65 - 0.66)	-2.34%
PUFA	1.04 (1.03 - 1.04)	1.03 (1.02 - 1.03)	-0.97%	1.55 (1.54 - 1.55)	1.58 (1.57 - 1.58)	1.88%	0.67 (0.66 - 0.68)	0.65 (0.65 - 0.66)	-2.85%
n-3 fatty acids	1.03 (1.02 - 1.03)	1.04 (1.03 - 1.04)	0.95%	1.55 (1.54 - 1.55)	1.57 (1.57 - 1.58)	1.80%	0.66 (0.66 - 0.67)	0.66 (0.65 - 0.66)	-0.85%
n-6 fatty acids	1.04 (1.03 - 1.04)	1.03 (1.02 - 1.03)	-1.23%	1.55 (1.54 - 1.55)	1.57 (1.57 - 1.58)	1.64%	0.67 (0.67 - 0.68)	0.65 (0.65 - 0.66)	-2.87%
**Protein**									
Total protein	1.03 (1.02 - 1.03)	1.04 (1.03 - 1.05)	1.31%	1.56 (1.55 - 1.56)	1.57 (1.56 - 1.57)	0.63%	0.66 (0.65 - 0.67)	0.66 (0.66 - 0.67)	0.68%
Protein from plant sources	1.05 (1.04 - 1.05)	1.02 (1.01 - 1.02)	-2.79%	1.58 (1.57 - 1.59)	1.54 (1.53 - 1.55)	-2.49%	0.66 (0.66 - 0.67)	0.66 (0.66 - 0.67)	-0.30%
Protein from animal sources	1.02 (1.01 - 1.03)	1.04 (1.03 - 1.05)	2.07%	1.55 (1.54 - 1.55)	1.57 (1.56 - 1.57)	1.42%	0.66 (0.65 - 0.66)	0.66 (0.66 - 0.67)	0.65%
Protein from dairy	1.04 (1.03 - 1.05)	1.03 (1.02 - 1.03)	-1.29%	1.56 (1.56 - 1.57)	1.56 (1.56 - 1.57)	0.02%	0.67 (0.66 - 0.67)	0.66 (0.65 - 0.66)	-1.31%
Protein from non-dairy animal sources	1.02 (1.01 - 1.02)	1.04 (1.04 - 1.05)	2.29%	1.55 (1.54 - 1.55)	1.57 (1.56 - 1.57)	1.30%	0.66 (0.65 - 0.66)	0.66 (0.66 - 0.67)	0.99%

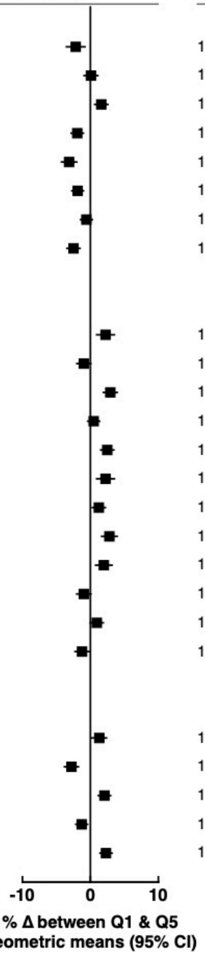

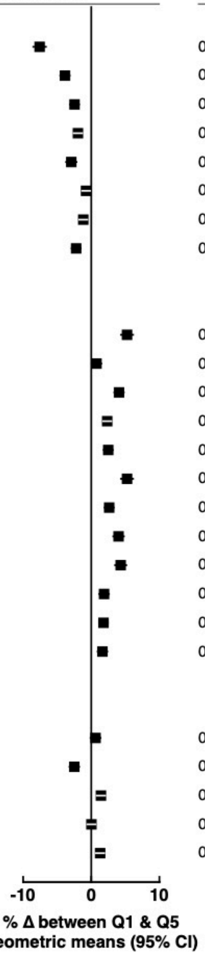

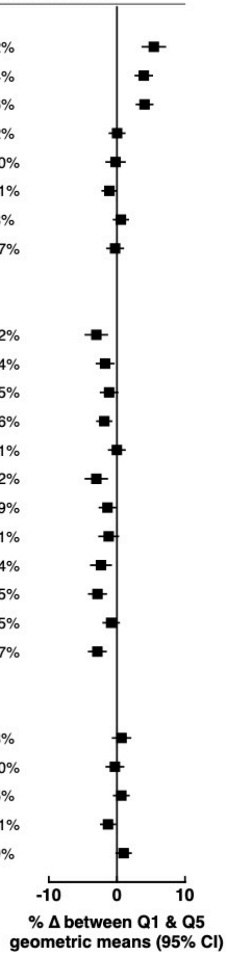

Supplemental Figure XIX. Geometric mean of ApoB (mmol/L), ApoA1 (mmol/L) and ApoB-to-ApoA1 ratio by lowest (Q1) and highest (Q5) intake of **macronutrients in grams per day** (n=24,639).

Models adjusted for age at recruitment (<45, 45-49, 50-54, 55-59, 60-64, ≥65), sex, ethnicity (white, mixed race, Asian or Asian British, black or black British, other, unknown), region (London, North-West England, North-Eastern England, Yorkshire & the Humber, West Midlands, East Midlands, South-West England, Wales), Townsend deprivation index (quintiles from least to most affluent, unknown), smoking status (never, previous, <15 cigarettes/day, 15-29 cigarettes/day, ≥30 cigarettes/day, unknown), physical activity (low, medium or high according to MET hours per week, unknown), alcohol (<1, 1-9, 10-19, 20-29, ≥30 g/day, unknown), body mass index (<20, 20-22.4, 22.5-24.9, 25-27.4, 27.5-29.9, 30-32.4, 32.5-34.9, 35-37.4, 37.5-39.9 or ≥40 in kg/m2), height (sex-specific groups in 5cm increments, unknown), diabetes diagnosed by a doctor (yes, no, unknown) and mean daily energy intake (quintiles).

Abbreviations: Apo = apolipoprotein; CI = confidence interval; g/day = grams per day; MUFA = monounsaturated fatty acids; n-3 = omega-3; n-6 = omega-6; PUFA = polyunsaturated fatty acids; Q = quintile; SFA = saturated fatty acids.

Nutrients	Total cholesterol (mmol/L) Geometric mean in Q1 (95% CI)	Geometric mean in Q5 (95% CI)	% Δ between Q1 & Q5 geometric means	LDL-C (mmol/L) Geometric mean in Q1 (95% CI)	Geometric mean in Q5 (95% CI)	% Δ between Q1 & Q5 geometric means	HDL-C (mmol/L) Geometric mean in Q1 (95% CI)	Geometric mean in Q5 (95% CI)	% Δ between Q1 & Q5 geometric means
**Carbohydrate**									
Total carbohydrates	5.93 (5.86 - 5.99)	5.63 (5.57 - 5.69)	-5.07%	3.66 (3.61 - 3.72)	3.50 (3.46 - 3.55)	-4.50%	1.57 (1.55 - 1.59)	1.46 (1.44 - 1.47)	-7.27%
Total sugars	5.82 (5.76 - 5.88)	5.74 (5.68 - 5.80)	-1.36%	3.60 (3.55 - 3.65)	3.58 (3.54 - 3.63)	-0.53%	1.54 (1.52 - 1.56)	1.48 (1.47 - 1.50)	-3.56%
Free sugars	5.74 (5.68 - 5.80)	5.80 (5.74 - 5.86)	1.06%	3.53 (3.49 - 3.58)	3.63 (3.58 - 3.68)	2.70%	1.55 (1.53 - 1.57)	1.46 (1.45 - 1.48)	-5.66%
Non-free sugars	5.87 (5.81 - 5.93)	5.70 (5.64 - 5.76)	-2.93%	3.65 (3.60 - 3.70)	3.52 (3.47 - 3.57)	-3.60%	1.51 (1.49 - 1.52)	1.52 (1.51 - 1.54)	1.20%
Total starch	5.86 (5.80 - 5.92)	5.67 (5.61 - 5.73)	-3.22%	3.63 (3.59 - 3.68)	3.51 (3.47 - 3.56)	-3.37%	1.53 (1.51 - 1.55)	1.48 (1.47 - 1.50)	-3.19%
Starch from wholegrains	5.80 (5.74 - 5.86)	5.68 (5.62 - 5.74)	-2.00%	3.60 (3.56 - 3.65)	3.51 (3.46 - 3.55)	-2.67%	1.51 (1.49 - 1.53)	1.51 (1.50 - 1.53)	0.33%
Starch from refined grains	5.81 (5.75 - 5.87)	5.73 (5.67 - 5.78)	-1.38%	3.60 (3.55 - 3.64)	3.56 (3.51 - 3.61)	-1.06%	1.53 (1.51 - 1.54)	1.49 (1.47 - 1.51)	-2.56%
Fiber*	5.86 (5.80 - 5.92)	5.65 (5.58 - 5.71)	-3.72%	3.66 (3.61 - 3.71)	3.47 (3.42 - 3.52)	-5.30%	1.49 (1.47 - 1.51)	1.53 (1.51 - 1.55)	2.72%
**Fat**									
Total fat	5.68 (5.62 - 5.74)	5.94 (5.87 - 6.00)	4.36%	3.52 (3.47 - 3.57)	3.69 (3.64 - 3.74)	4.79%	1.49 (1.48 - 1.51)	1.55 (1.53 - 1.56)	3.42%
Fat from plant sources	5.86 (5.80 - 5.92)	5.73 (5.68 - 5.79)	-2.20%	3.65 (3.61 - 3.70)	3.54 (3.49 - 3.59)	-3.20%	1.51 (1.49 - 1.53)	1.52 (1.50 - 1.54)	0.79%
Fat from animal sources	5.59 (5.54 - 5.65)	5.94 (5.88 - 6.00)	5.96%	3.45 (3.41 - 3.50)	3.70 (3.66 - 3.75)	7.02%	1.48 (1.46 - 1.50)	1.53 (1.52 - 1.55)	3.53%
Fat from dairy	5.71 (5.65 - 5.77)	5.89 (5.83 - 5.95)	3.10%	3.53 (3.49 - 3.58)	3.67 (3.62 - 3.71)	3.74%	1.49 (1.48 - 1.51)	1.53 (1.51 - 1.55)	2.62%
Fat from non-dairy animal sources	5.65 (5.59 - 5.71)	5.85 (5.79 - 5.91)	3.60%	3.49 (3.44 - 3.53)	3.64 (3.60 - 3.69)	4.32%	1.50 (1.48 - 1.52)	1.52 (1.50 - 1.54)	1.34%
Dietary cholesterol	5.67 (5.62 - 5.73)	5.86 (5.80 - 5.92)	3.22%	3.51 (3.47 - 3.56)	3.64 (3.60 - 3.69)	3.64%	1.49 (1.48 - 1.51)	1.52 (1.50 - 1.54)	1.80%
Trans fatty acids	5.65 (5.59 - 5.71)	5.85 (5.80 - 5.91)	3.51%	3.49 (3.44 - 3.53)	3.64 (3.60 - 3.69)	4.40%	1.50 (1.48 - 1.52)	1.52 (1.51 - 1.54)	1.76%
SFA	5.63 (5.58 - 5.69)	5.90 (5.84 - 5.96)	4.66%	3.48 (3.43 - 3.52)	3.69 (3.64 - 3.73)	5.86%	1.50 (1.48 - 1.52)	1.52 (1.51 - 1.54)	1.71%
MUFA	5.71 (5.65 - 5.77)	5.88 (5.81 - 5.94)	2.79%	3.55 (3.51 - 3.60)	3.65 (3.60 - 3.70)	2.70%	1.49 (1.47 - 1.50)	1.53 (1.51 - 1.55)	2.94%
PUFA	5.77 (5.71 - 5.83)	5.76 (5.70 - 5.82)	-0.15%	3.59 (3.54 - 3.64)	3.55 (3.51 - 3.60)	-1.09%	1.49 (1.47 - 1.50)	1.54 (1.52 - 1.56)	3.35%
n-3 fatty acids	5.74 (5.69 - 5.80)	5.82 (5.77 - 5.88)	1.38%	3.57 (3.53 - 3.62)	3.60 (3.55 - 3.65)	0.68%	1.48 (1.46 - 1.50)	1.55 (1.53 - 1.57)	4.75%
n-6 fatty acids	5.79 (5.73 - 5.85)	5.74 (5.68 - 5.80)	-0.77%	3.60 (3.55 - 3.65)	3.54 (3.49 - 3.59)	-1.64%	1.49 (1.48 - 1.51)	1.53 (1.52 - 1.55)	2.65%
**Protein**									
Total protein	5.75 (5.69 - 5.81)	5.84 (5.78 - 5.90)	1.62%	3.57 (3.52 - 3.61)	3.62 (3.57 - 3.67)	1.57%	1.50 (1.48 - 1.51)	1.53 (1.51 - 1.55)	2.38%
Protein from plant sources	5.86 (5.79 - 5.92)	5.61 (5.55 - 5.67)	-4.28%	3.65 (3.60 - 3.70)	3.46 (3.41 - 3.50)	-5.46%	1.51 (1.49 - 1.53)	1.50 (1.48 - 1.52)	-0.97%
Protein from animal sources	5.69 (5.63 - 5.75)	5.84 (5.78 - 5.90)	2.66%	3.52 (3.48 - 3.57)	3.63 (3.58 - 3.67)	2.87%	1.50 (1.48 - 1.51)	1.53 (1.51 - 1.55)	2.32%
Protein from dairy	5.80 (5.74 - 5.86)	5.77 (5.71 - 5.83)	-0.49%	3.61 (3.56 - 3.65)	3.57 (3.52 - 3.62)	-0.96%	1.50 (1.48 - 1.52)	1.53 (1.51 - 1.55)	2.17%
Protein from non-dairy animal sources	5.66 (5.60 - 5.72)	5.87 (5.81 - 5.93)	3.64%	3.51 (3.46 - 3.55)	3.65 (3.60 - 3.70)	4.10%	1.49 (1.47 - 1.51)	1.53 (1.51 - 1.55)	2.61%

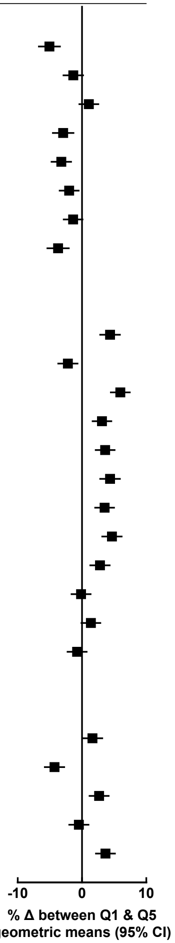

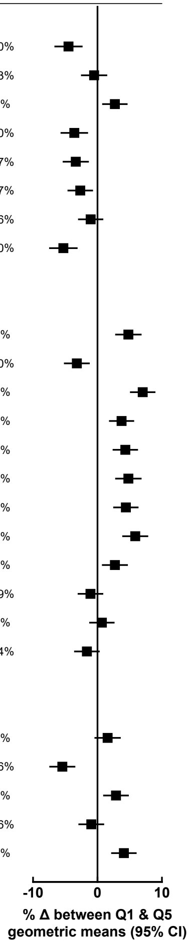

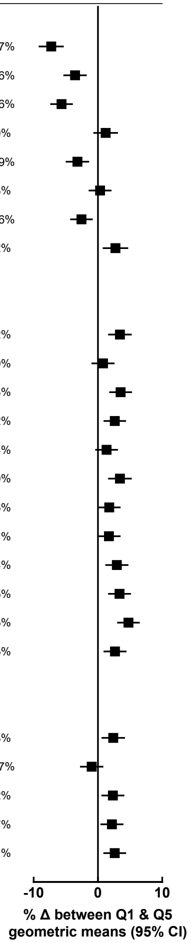

Supplemental Figure XX. Geometric mean of total cholesterol (mmol/L), LDL-C (mmol/L) and HDL-C (mmol/L) by lowest (Q1) and highest (Q5) percentage intake of macronutrients **restricting to participants with follow-up serum lipid measurements** (n=6,215)**.**

Models adjusted for age at recruitment (<45, 45-49, 50-54, 55-59, 60-64, ≥65), sex, ethnicity (white, mixed race, Asian or Asian British, black or black British, other, unknown), region (London, North-West England, North-Eastern England, Yorkshire & the Humber, West Midlands, East Midlands, South-West England, Wales), Townsend deprivation index (quintiles from least to most affluent, unknown), smoking status (never, previous, <15 cigarettes/day, 15-29 cigarettes/day, ≥30 cigarettes/day, unknown), physical activity (low, medium or high according to MET hours per week, unknown), alcohol (<1, 1-9, 10-19, 20-29, ≥30 g/day, unknown), body mass index (<20, 20-22.4, 22.5-24.9, 25-27.4, 27.5-29.9, 30-32.4, 32.5-34.9, 35-37.4, 37.5-39.9 or ≥40 in kg/m2), height (sex-specific groups in 5cm increments, unknown), diabetes diagnosed by a doctor (yes, no, unknown) and mean daily energy intake (quintiles).

*For fiber Q1 and Q5 represent quintiles of total fiber intake (g/day).

Abbreviations: CI = confidence interval; g/day = grams per day; HDL-C = high-density lipoprotein cholesterol; LDL-C= low-density lipoprotein cholesterol; MUFA = monounsaturated fatty acids; n-3 = omega-3; n-6 = omega-6; PUFA = polyunsaturated fatty acids; Q = quintile; SFA = saturated fatty acids.

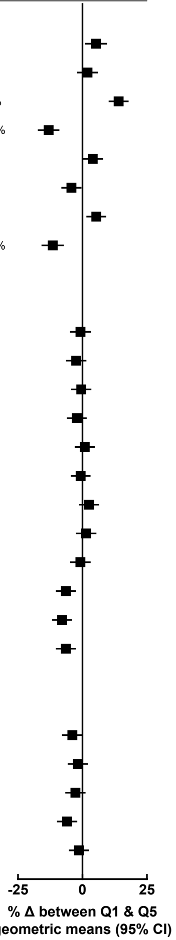

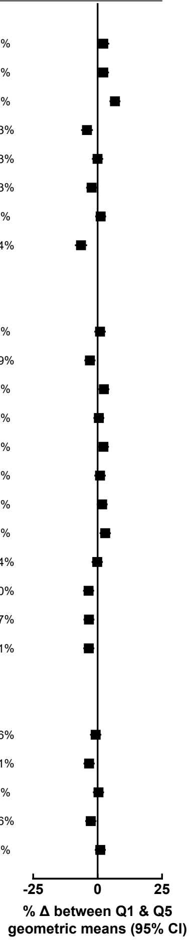

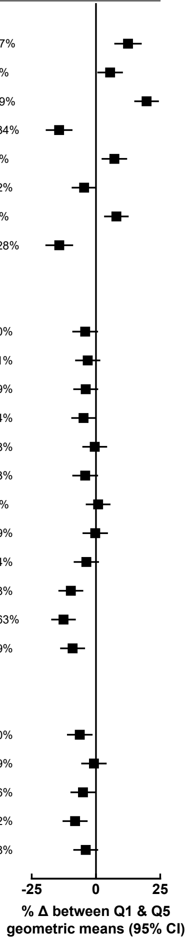

	Triglycerides (mmol/L)			TC-to-HDL-C ratio			TG-to-HDL-C ratio		
**Nutrients**	**Geometric mean in Q1 (95% CI)**	**Geometric mean in Q5 (95% CI)**	**% Δ between Q1 & Q5 geometric means**	**Geometric mean in Q1 (95% CI)**	**Geometric mean in Q5 (95% CI)**	**% Δ between Q1 & Q5 geometric means**	**Geometric mean in Q1 (95% CI)**	**Geometric mean in Q5 (95% CI)**	**% Δ between Q1 & Q5 geometric means**
**Carbohydrate**									
Total carbohydrates	1.43 (1.40 - 1.47)	1.51 (1.47 - 1.55)	5.20%	3.78 (3.73 - 3.83)	3.87 (3.81 - 3.92)	2.20%	0.92 (0.88 - 0.95)	1.04 (1.00 - 1.07)	12.47%
Total sugars	1.46 (1.42 - 1.49)	1.49 (1.45 - 1.52)	1.94%	3.79 (3.74 - 3.84)	3.87 (3.82 - 3.92)	2.20%	0.95 (0.92 - 0.98)	1.00 (0.97 - 1.03)	5.50%
Free sugars	1.37 (1.34 - 1.40)	1.58 (1.54 - 1.62)	14.02%	3.71 (3.66 - 3.75)	3.96 (3.91 - 4.01)	6.73%	0.88 (0.86 - 0.91)	1.08 (1.04 - 1.11)	19.69%
Non-free sugars	1.56 (1.52 - 1.60)	1.37 (1.33 - 1.40)	-13.14%	3.90 (3.85 - 3.95)	3.74 (3.69 - 3.79)	-4.13%	1.04 (1.00 - 1.07)	0.90 (0.87 - 0.93)	-14.34%
Total starch	1.43 (1.40 - 1.47)	1.49 (1.45 - 1.53)	3.96%	3.82 (3.77 - 3.87)	3.82 (3.77 - 3.87)	-0.03%	0.94 (0.91 - 0.97)	1.00 (0.97 - 1.04)	7.16%
Starch from wholegrains	1.48 (1.45 - 1.52)	1.42 (1.39 - 1.46)	-4.29%	3.85 (3.80 - 3.89)	3.76 (3.71 - 3.80)	-2.33%	0.98 (0.95 - 1.02)	0.94 (0.91 - 0.97)	-4.62%
Starch from refined grains	1.41 (1.37 - 1.44)	1.48 (1.45 - 1.52)	5.37%	3.80 (3.76 - 3.85)	3.85 (3.80 - 3.90)	1.18%	0.92 (0.89 - 0.95)	1.00 (0.97 - 1.03)	7.94%
Fiber*	1.53 (1.49 - 1.57)	1.37 (1.33 - 1.40)	-11.56%	3.93 (3.88 - 3.98)	3.68 (3.63 - 3.73)	-6.44%	1.03 (0.99 - 1.06)	0.89 (0.86 - 0.92)	-14.28%
**Fat**									
Total fat	1.47 (1.43 - 1.51)	1.46 (1.42 - 1.50)	-0.78%	3.81 (3.76 - 3.86)	3.84 (3.79 - 3.89)	0.94%	0.98 (0.95 - 1.02)	0.94 (0.91 - 0.97)	-4.20%
Fat from plant sources	1.49 (1.45 - 1.53)	1.45 (1.42 - 1.49)	-2.43%	3.88 (3.83 - 3.93)	3.77 (3.72 - 3.82)	-2.99%	0.99 (0.95 - 1.02)	0.95 (0.92 - 0.98)	-3.21%
Fat from animal sources	1.45 (1.42 - 1.49)	1.45 (1.41 - 1.48)	-0.46%	3.78 (3.73 - 3.82)	3.87 (3.82 - 3.92)	2.43%	0.98 (0.95 - 1.01)	0.94 (0.91 - 0.97)	-3.99%
Fat from dairy	1.49 (1.45 - 1.52)	1.45 (1.42 - 1.49)	-2.22%	3.83 (3.78 - 3.87)	3.84 (3.80 - 3.89)	0.48%	1.00 (0.97 - 1.03)	0.95 (0.92 - 0.98)	-4.84%
Fat from non-dairy animal sources	1.44 (1.41 - 1.48)	1.46 (1.42 - 1.49)	0.86%	3.77 (3.72 - 3.81)	3.85 (3.80 - 3.90)	2.26%	0.96 (0.93 - 0.99)	0.96 (0.93 - 0.99)	-0.48%
Dietary cholesterol	1.46 (1.43 - 1.50)	1.45 (1.42 - 1.49)	-0.72%	3.80 (3.75 - 3.85)	3.86 (3.81 - 3.90)	1.42%	0.98 (0.95 - 1.01)	0.96 (0.93 - 0.99)	-2.53%
Trans fatty acids	1.43 (1.39 - 1.46)	1.47 (1.43 - 1.50)	2.59%	3.77 (3.73 - 3.82)	3.84 (3.79 - 3.89)	1.76%	0.95 (0.92 - 0.98)	0.96 (0.93 - 0.99)	0.84%
SFA	1.44 (1.40 - 1.48)	1.46 (1.42 - 1.50)	1.42%	3.76 (3.71 - 3.81)	3.87 (3.83 - 3.92)	2.95%	0.96 (0.93 - 0.99)	0.96 (0.93 - 0.99)	-0.29%
MUFA	1.47 (1.43 - 1.51)	1.46 (1.42 - 1.50)	-0.80%	3.84 (3.79 - 3.89)	3.84 (3.79 - 3.89)	-0.14%	0.99 (0.96 - 1.02)	0.95 (0.92 - 0.98)	-3.74%
PUFA	1.51 (1.47 - 1.55)	1.42 (1.38 - 1.45)	-6.42%	3.88 (3.83 - 3.93)	3.75 (3.70 - 3.80)	-3.50%	1.02 (0.98 - 1.05)	0.92 (0.89 - 0.95)	-9.78%
n-3 fatty acids	1.52 (1.48 - 1.56)	1.40 (1.37 - 1.44)	-7.88%	3.88 (3.83 - 3.93)	3.75 (3.71 - 3.80)	-3.37%	1.03 (0.99 - 1.06)	0.90 (0.88 - 0.93)	-12.63%
n-6 fatty acids	1.51 (1.47 - 1.55)	1.41 (1.38 - 1.45)	-6.44%	3.88 (3.83 - 3.92)	3.75 (3.70 - 3.79)	-3.41%	1.01 (0.98 - 1.04)	0.92 (0.89 - 0.95)	-9.09%
**Protein**									
Total protein	1.48 (1.44 - 1.52)	1.42 (1.39 - 1.46)	-3.92%	3.84 (3.79 - 3.89)	3.81 (3.76 - 3.86)	-0.76%	0.99 (0.96 - 1.02)	0.93 (0.90 - 0.96)	-6.30%
Protein from plant sources	1.46 (1.42 - 1.50)	1.43 (1.40 - 1.47)	-1.76%	3.87 (3.82 - 3.92)	3.75 (3.70 - 3.79)	-3.31%	0.96 (0.93 - 1.00)	0.96 (0.93 - 0.99)	-0.79%
Protein from animal sources	1.47 (1.43 - 1.50)	1.43 (1.39 - 1.46)	-2.74%	3.81 (3.76 - 3.85)	3.82 (3.77 - 3.87)	0.34%	0.98 (0.95 - 1.01)	0.93 (0.90 - 0.96)	-5.06%
Protein from dairy	1.50 (1.46 - 1.54)	1.41 (1.38 - 1.45)	-5.95%	3.87 (3.82 - 3.92)	3.77 (3.72 - 3.82)	-2.66%	1.00 (0.97 - 1.03)	0.92 (0.89 - 0.95)	-8.12%
Protein from non-dairy animal sources	1.47 (1.43 - 1.51)	1.45 (1.41 - 1.49)	-1.37%	3.80 (3.76 - 3.85)	3.84 (3.80 - 3.89)	1.03%	0.99 (0.96 - 1.02)	0.95 (0.92 - 0.98)	-3.98%

Supplemental Figure XXI. Geometric mean of triglycerides (mmol/L), TC-to-HDL-C ratio and TG-to-HDL-C ratio by lowest (Q1) and highest (Q5) percentage intake of macronutrients in a **restricting to participants with follow-up serum lipid measurements** (n=6,215).

Models adjusted for age at recruitment (<45, 45-49, 50-54, 55-59, 60-64, ≥65), sex, ethnicity (white, mixed race, Asian or Asian British, black or black British, other, unknown), region (London, North-West England, North-Eastern England, Yorkshire & the Humber, West Midlands, East Midlands, South-West England, Wales), Townsend deprivation index (quintiles from least to most affluent, unknown), smoking status (never, previous, <15 cigarettes/day, 15-29 cigarettes/day, ≥30 cigarettes/day, unknown), physical activity (low, medium or high according to MET hours per week, unknown), alcohol (<1, 1-9, 10-19, 20-29, ≥30 g/day, unknown), body mass index (<20, 20-22.4, 22.5-24.9, 25-27.4, 27.5-29.9, 30-32.4, 32.5-34.9, 35-37.4, 37.5-39.9 or ≥40 in kg/m2), height (sex-specific groups in 5cm increments, unknown), diabetes diagnosed by a doctor (yes, no, unknown) and mean daily energy intake (quintiles).

*For fiber Q1 and Q5 represent quintiles of total fiber intake (g/day).

Abbreviations: CI = confidence interval; g/day = grams per day; HDL-C = high-density lipoprotein cholesterol; MUFA = monounsaturated fatty acids; n-3 = omega-3; n-6 = omega-6; PUFA = polyunsaturated fatty acids; Q = quintile; SFA = saturated fatty acids; TC = total cholesterol; TG = triglycerides

Nutrients	ApoB (mmol/L) Geometric mean in Q1 (95% CI)	ApoB (mmol/L) Geometric mean in Q5 (95% CI)	ApoB (mmol/L) % Δ between Q1 & Q5 geometric means	ApoA1 (mmol/L) Geometric mean in Q1 (95% CI)	ApoA1 (mmol/L) Geometric mean in Q5 (95% CI)	ApoA1 (mmol/L) % Δ between Q1 & Q5 geometric means	ApoB-to-ApoA1 ratio Geometric mean in Q1 (95% CI)	ApoB-to-ApoA1 ratio Geometric mean in Q5 (95% CI)	ApoB-to-ApoA1 ratio % Δ between Q1 & Q5 geometric means
**Carbohydrate**									
Total carbohydrates	1.05 (1.03 - 1.06)	1.01 (1.00 - 1.02)	-3.58%	1.63 (1.61 - 1.64)	1.54 (1.53 - 1.56)	-5.33%	0.64 (0.63 - 0.65)	0.65 (0.64 - 0.66)	1.75%
Total sugars	1.03 (1.01 - 1.04)	1.03 (1.01 - 1.04)	0.08%	1.61 (1.60 - 1.62)	1.56 (1.55 - 1.57)	-3.16%	0.64 (0.63 - 0.65)	0.66 (0.65 - 0.67)	3.24%
Free sugars	1.01 (1.00 - 1.02)	1.04 (1.03 - 1.05)	2.97%	1.60 (1.59 - 1.62)	1.56 (1.55 - 1.57)	-2.78%	0.63 (0.62 - 0.64)	0.67 (0.66 - 0.68)	5.75%
Non-free sugars	1.04 (1.03 - 1.05)	1.01 (0.99 - 1.02)	-3.29%	1.60 (1.58 - 1.61)	1.58 (1.56 - 1.59)	-1.29%	0.65 (0.64 - 0.66)	0.64 (0.63 - 0.65)	-2.00%
Total starch	1.04 (1.03 - 1.05)	1.01 (1.00 - 1.02)	-3.07%	1.60 (1.58 - 1.61)	1.57 (1.56 - 1.58)	-1.77%	0.65 (0.64 - 0.66)	0.64 (0.63 - 0.65)	-1.30%
Starch from wholegrains	1.03 (1.02 - 1.04)	1.01 (0.99 - 1.02)	-2.44%	1.59 (1.58 - 1.60)	1.58 (1.57 - 1.59)	-0.56%	0.65 (0.64 - 0.66)	0.64 (0.63 - 0.65)	-1.88%
Starch from refined grains	1.03 (1.02 - 1.04)	1.02 (1.01 - 1.03)	-0.93%	1.59 (1.57 - 1.60)	1.57 (1.56 - 1.58)	-0.99%	0.65 (0.64 - 0.66)	0.65 (0.64 - 0.66)	0.06%
Fiber*	1.05 (1.03 - 1.06)	1.00 (0.98 - 1.01)	-5.00%	1.58 (1.57 - 1.59)	1.59 (1.57 - 1.60)	0.36%	0.66 (0.65 - 0.67)	0.63 (0.62 - 0.64)	-5.35%
**Fat**									
Total fat	1.01 (1.00 - 1.03)	1.05 (1.04 - 1.06)	3.63%	1.57 (1.55 - 1.58)	1.62 (1.60 - 1.63)	3.15%	0.65 (0.64 - 0.66)	0.65 (0.64 - 0.66)	0.49%
Fat from plant sources	1.04 (1.03 - 1.06)	1.01 (1.00 - 1.03)	-2.88%	1.58 (1.57 - 1.59)	1.59 (1.58 - 1.61)	0.98%	0.66 (0.65 - 0.67)	0.63 (0.63 - 0.64)	-3.85%
Fat from animal sources	1.00 (0.98 - 1.01)	1.05 (1.04 - 1.06)	5.51%	1.56 (1.55 - 1.58)	1.60 (1.59 - 1.62)	2.55%	0.64 (0.63 - 0.65)	0.66 (0.65 - 0.67)	2.95%
Fat from dairy	1.02 (1.00 - 1.03)	1.04 (1.03 - 1.06)	2.52%	1.57 (1.56 - 1.58)	1.60 (1.59 - 1.62)	2.12%	0.65 (0.64 - 0.66)	0.65 (0.64 - 0.66)	0.39%
Fat from non-dairy animal sources	1.00 (0.99 - 1.01)	1.04 (1.03 - 1.05)	3.76%	1.58 (1.56 - 1.59)	1.59 (1.58 - 1.60)	0.89%	0.63 (0.63 - 0.64)	0.65 (0.64 - 0.66)	2.87%
Dietary cholesterol	1.01 (1.00 - 1.02)	1.04 (1.03 - 1.06)	3.49%	1.57 (1.56 - 1.58)	1.59 (1.58 - 1.60)	1.23%	0.64 (0.63 - 0.65)	0.66 (0.65 - 0.67)	2.26%
Trans fatty acids	1.01 (0.99 - 1.02)	1.04 (1.02 - 1.05)	3.13%	1.57 (1.56 - 1.58)	1.60 (1.59 - 1.61)	1.69%	0.64 (0.63 - 0.65)	0.65 (0.64 - 0.66)	1.44%
SFA	1.00 (0.99 - 1.02)	1.05 (1.03 - 1.06)	4.30%	1.57 (1.56 - 1.59)	1.60 (1.59 - 1.61)	1.79%	0.64 (0.63 - 0.65)	0.65 (0.64 - 0.66)	2.51%
MUFA	1.02 (1.01 - 1.03)	1.04 (1.03 - 1.05)	2.19%	1.56 (1.55 - 1.57)	1.61 (1.59 - 1.62)	2.92%	0.65 (0.64 - 0.66)	0.65 (0.64 - 0.66)	-0.73%
PUFA	1.03 (1.01 - 1.04)	1.02 (1.00 - 1.03)	-0.93%	1.57 (1.56 - 1.58)	1.60 (1.59 - 1.62)	2.22%	0.65 (0.64 - 0.66)	0.63 (0.62 - 0.64)	-3.15%
n-3 fatty acids	1.02 (1.01 - 1.04)	1.03 (1.02 - 1.04)	0.63%	1.57 (1.56 - 1.58)	1.60 (1.59 - 1.62)	2.00%	0.65 (0.64 - 0.66)	0.64 (0.63 - 0.65)	-1.37%
n-6 fatty acids	1.03 (1.02 - 1.04)	1.01 (1.00 - 1.03)	-1.43%	1.57 (1.56 - 1.59)	1.60 (1.59 - 1.62)	1.89%	0.65 (0.64 - 0.66)	0.63 (0.62 - 0.64)	-3.32%
**Protein**									
Total protein	1.02 (1.01 - 1.03)	1.04 (1.02 - 1.05)	1.48%	1.58 (1.57 - 1.59)	1.59 (1.58 - 1.60)	0.63%	0.65 (0.64 - 0.66)	0.65 (0.64 - 0.66)	0.85%
Protein from plant sources	1.04 (1.03 - 1.06)	1.00 (0.98 - 1.01)	-4.66%	1.59 (1.57 - 1.60)	1.57 (1.55 - 1.58)	-1.15%	0.66 (0.65 - 0.67)	0.63 (0.63 - 0.64)	-3.50%
Protein from animal sources	1.01 (1.00 - 1.02)	1.04 (1.02 - 1.05)	2.58%	1.58 (1.56 - 1.59)	1.59 (1.58 - 1.61)	1.12%	0.64 (0.63 - 0.65)	0.65 (0.64 - 0.66)	1.46%
Protein from dairy	1.03 (1.02 - 1.05)	1.02 (1.00 - 1.03)	-1.72%	1.58 (1.57 - 1.59)	1.59 (1.58 - 1.60)	0.59%	0.65 (0.64 - 0.66)	0.64 (0.63 - 0.65)	-2.31%
Protein from non-dairy animal sources	1.00 (0.99 - 1.02)	1.05 (1.03 - 1.06)	4.02%	1.57 (1.56 - 1.58)	1.59 (1.58 - 1.61)	1.43%	0.64 (0.63 - 0.65)	0.66 (0.65 - 0.67)	2.58%

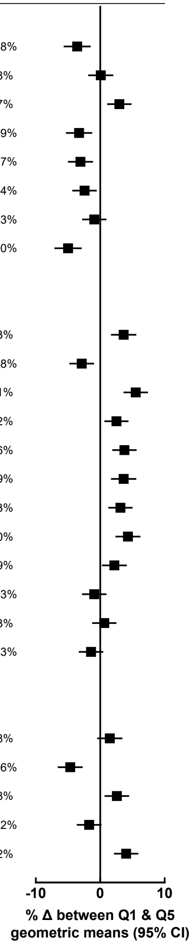

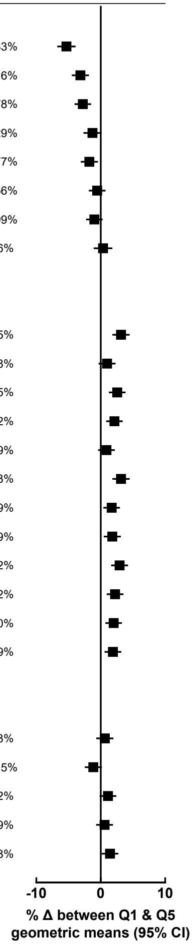

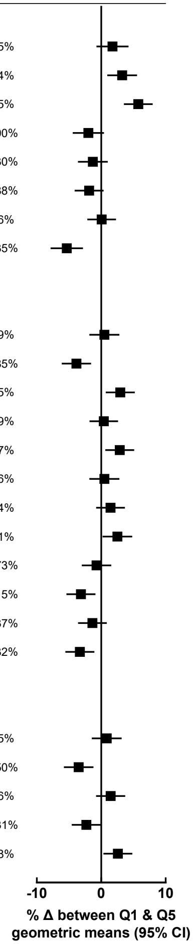

Supplemental Figure XXII. Geometric mean of ApoB (mmol/L), ApoA1 (mmol/L) and ApoB-to-ApoA1 ratio by lowest (Q1) and highest (Q5) percentage intake of macronutrients **restricting to participants with follow-up serum lipid measurements** (n=6,215**)**.

Models adjusted for age at recruitment (<45, 45-49, 50-54, 55-59, 60-64, ≥65), sex, ethnicity (white, mixed race, Asian or Asian British, black or black British, other, unknown), region (London, North-West England, North-Eastern England, Yorkshire & the Humber, West Midlands, East Midlands, South-West England, Wales), Townsend deprivation index (quintiles from least to most affluent, unknown), smoking status (never, previous, <15 cigarettes/day, 15-29 cigarettes/day, ≥30 cigarettes/day, unknown), physical activity (low, medium or high according to MET hours per week, unknown), alcohol (<1, 1-9, 10-19, 20-29, ≥30 g/day, unknown), body mass index (<20, 20-22.4, 22.5-24.9, 25-27.4, 27.5-29.9, 30-32.4, 32.5-34.9, 35-37.4, 37.5-39.9 or ≥40 in kg/m2), height (sex-specific groups in 5cm increments, unknown), diabetes diagnosed by a doctor (yes, no, unknown) and mean daily energy intake (quintiles).

*For fiber Q1 and Q5 represent quintiles of total fiber intake (g/day).

Abbreviations: Apo = apolipoprotein; CI = confidence interval; g/day = grams per day; MUFA = monounsaturated fatty acids; n-3 = omega-3; n-6 = omega-6; PUFA = polyunsaturated fatty acids; Q = quintile; SFA = saturated fatty acids.

SUPPLEMENTAL TABLES AND SUPPORTING INFORMATION

Supplemental Table I. Daily macronutrient intake in quintiles by daily macronutrient intake in grams and percentage of total energy intake.

		Intake*		% of total energy intake				Intake*		% of total energy intake	
**Nutrient†**	**N**	**mean**	**SD**	**mean**	**SD**	**Nutrient†**	**N**	**mean**	**SD**	**mean**	**SD**
Total carbohydrates						Dietary cholesterol					
Q1 (Lowest)	4928	205.64	53.44	39.16%	4.31%	Q1 (Lowest)	4928	118.71	42.16	0.51%	0.13%
Q2	4928	241.84	54.00	46.04%	1.21%	Q2	4928	179.18	40.85	0.77%	0.05%
Q3	4928	256.42	54.77	49.72%	0.98%	Q3	4928	219.81	51.26	0.96%	0.06%
Q4	4928	271.06	58.53	53.28%	1.14%	Q4	4928	276.24	67.27	1.21%	0.10%
Q5 (Highest)	4927	284.79	64.36	59.11%	3.34%	Q5 (Highest)	4927	408.83	132.63	1.90%	0.53%
Total sugars						Trans fatty acids					
Q1 (Lowest)	4928	83.57	23.44	16.09%	2.48%	Q1 (Lowest)	4928	0.60	0.21	0.27%	0.06%
Q2	4928	109.47	24.09	20.96%	0.98%	Q2	4928	0.92	0.22	0.40%	0.03%
Q3	4928	124.36	27.33	24.13%	0.90%	Q3	4928	1.15	0.26	0.50%	0.03%
Q4	4928	138.79	31.23	27.51%	1.11%	Q4	4928	1.40	0.32	0.60%	0.03%
Q5 (Highest)	4927	163.60	40.58	33.83%	3.96%	Q5 (Highest)	4927	1.88	0.52	0.81%	0.14%
Free sugars						SFA					
Q1 (Lowest)	4928	26.96	10.30	5.48%	1.51%	Q1 (Lowest)	4928	16.54	4.79	7.73%	1.13%
Q2	4928	44.15	10.45	8.64%	0.69%	Q2	4928	22.44	5.10	9.99%	0.46%
Q3	4928	56.57	12.80	10.90%	0.66%	Q3	4928	26.51	5.74	11.48%	0.42%
Q4	4928	70.91	16.21	13.43%	0.84%	Q4	4928	30.88	6.84	13.02%	0.50%
Q5 (Highest)	4927	98.66	29.01	18.60%	3.53%	Q5 (Highest)	4927	37.96	9.14	15.74%	1.59%
Non-free sugars						MUFA					
Q1 (Lowest)	4928	34.97	11.00	6.43%	1.44%	Q1 (Lowest)	4928	17.57	4.83	8.23%	1.04%
Q2	4928	51.55	11.85	9.80%	0.77%	Q2	4928	23.02	5.09	10.15%	0.38%
Q3	4928	63.02	13.93	12.39%	0.77%	Q3	4928	26.31	5.69	11.35%	0.33%
Q4	4928	75.63	17.06	15.36%	1.00%	Q4	4928	29.90	6.54	12.58%	0.40%
Q5 (Highest)	4927	97.37	26.01	21.47%	3.91%	Q5 (Highest)	4927	35.42	8.48	14.83%	1.43%
Total starch						PUFA					
Q1 (Lowest)	4928	88.12	27.25	17.21%	2.99%	Q1 (Lowest)	4928	8.44	2.37	3.78%	0.50%
Q2	4928	116.86	25.99	22.19%	0.90%	Q2	4928	10.95	2.44	4.79%	0.21%
Q3	4928	129.94	28.31	24.95%	0.75%	Q3	4928	12.74	2.85	5.49%	0.21%
Q4	4928	141.94	30.63	27.68%	0.87%	Q4	4928	14.51	3.30	6.28%	0.27%
Q5 (Highest)	4927	161.92	38.14	32.53%	2.94%	Q5 (Highest)	4927	18.24	4.80	7.93%	1.10%
Starch from						n-3 fatty acids					
Q1 (Lowest)	4928	2.35	3.51	0.42%	0.60%	Q1 (Lowest)	4928	1.21	0.34	0.54%	0.07%
Q2	4928	14.60	4.81	2.78%	0.61%	Q2	4928	1.62	0.37	0.69%	0.04%
Q3	4928	24.47	6.31	4.76%	0.55%	Q3	4928	1.91	0.43	0.82%	0.04%
Q4	4928	34.60	8.63	6.95%	0.75%	Q4	4928	2.28	0.52	0.98%	0.06%
Q5 (Highest)	4927	55.02	18.03	11.56%	2.92%	Q5 (Highest)	4927	2.96	0.82	1.37%	0.28%
Starch from refined						n-6 fatty acids					
Q1 (Lowest)	4928	9.74	7.99	1.83%	1.39%	Q1 (Lowest)	4928	6.90	1.96	3.11%	0.43%
Q2	4928	28.61	8.10	5.46%	0.82%	Q2	4928	9.09	2.04	3.99%	0.19%
Q3	4928	43.00	10.68	8.22%	0.80%	Q3	4928	10.75	2.38	4.61%	0.18%
Q4	4928	58.01	14.23	11.26%	1.00%	Q4	4928	12.34	2.82	5.32%	0.25%
Q5 (Highest)	4927	84.65	25.20	17.18%	3.74%	Q5 (Highest)	4927	15.82	4.24	6.83%	1.00%
Fiber						Total protein					
Q1 (Lowest)	4928	10.88	1.94			Q1 (Lowest)	4928	67.00	16.24	12.14%	1.10%
Q2	4928	14.75	0.81			Q2	4928	76.69	15.99	14.26%	0.44%
Q3	4928	17.44	0.74			Q3	4928	81.47	16.60	15.66%	0.40%
Q4	4928	20.34	0.98			Q4	4928	85.15	17.57	17.18%	0.50%
Q5 (Highest)	4927	26.27	4.02			Q5 (Highest)	4927	89.64	19.94	20.28%	2.12%
Total fat						Protein from plant					
Q1 (Lowest)	4928	50.43	13.54	23.67%	2.74%	Q1 (Lowest)	4928	21.06	5.53	3.95%	0.48%
Q2	4928	64.11	13.96	28.60%	0.93%	Q2	4928	25.48	5.64	4.84%	0.18%
Q3	4928	73.23	15.98	31.55%	0.81%	Q3	4928	27.70	5.92	5.42%	0.17%
Q4	4928	81.65	17.76	34.49%	0.94%	Q4	4928	30.35	6.77	6.07%	0.22%
Q5 (Highest)	4927	94.93	21.79	39.41%	2.92%	Q5 (Highest)	4927	36.63	9.94	7.61%	1.14%
Fat from plant sources						Protein from animal					
Q1 (Lowest)	4928	18.52	5.75	8.42%	1.57%	Q1 (Lowest)	4928	32.33	11.39	6.03%	1.51%
Q2	4928	26.12	6.03	11.48%	0.63%	Q2	4928	47.13	10.18	8.69%	0.48%
Q3	4928	31.35	6.95	13.58%	0.59%	Q3	4928	53.42	11.31	10.19%	0.41%
Q4	4928	37.03	8.31	15.89%	0.78%	Q4	4928	58.65	12.39	11.77%	0.52%
Q5 (Highest)	4927	48.50	13.27	20.61%	3.13%	Q5 (Highest)	4927	67.22	16.44	14.97%	2.27%
Fat from animal						Protein from dairy					
Q1 (Lowest)	4928	22.91	7.48	10.47%	2.25%	Q1 (Lowest)	4928	5.39	2.79	1.02%	0.45%
Q2	4928	33.36	7.68	14.64%	0.84%	Q2	4928	10.27	2.55	1.96%	0.20%
Q3	4928	40.07	8.99	17.30%	0.73%	Q3	4928	13.34	3.05	2.60%	0.18%
Q4	4928	47.20	10.68	20.11%	0.93%	Q4	4928	16.18	3.66	3.27%	0.22%

Q5 (Highest)	4927	59.31	14.96	25.22%	3.08%
Fat from dairy					
Q1 (Lowest)	4928	3.06	1.79	1.37%	0.70%
Q2	4928	7.30	2.05	3.23%	0.46%
Q3	4928	10.99	2.80	4.83%	0.48%
Q4	4928	15.51	3.87	6.77%	0.68%
Q5 (Highest)	4927	25.36	8.55	10.97%	2.64%
Fat from non-dairy animal					
Q1 (Lowest)	4928	14.03	5.27	6.29%	1.76%
Q2	4928	22.38	5.25	9.70%	0.67%
Q3	4928	27.60	6.36	11.85%	0.61%
Q4	4928	33.43	7.68	14.16%	0.75%
Q5 (Highest)	4927	43.20	11.53	18.58%	2.86%

Q5 (Highest)	4927	21.21	5.71	4.59%	0.88%
Protein from non-dairy					
Q1 (Lowest)	4928	18.13	8.72	3.36%	1.35%
Q2	4928	32.72	7.56	6.05%	0.49%
Q3	4928	39.64	8.53	7.54%	0.41%
Q4	4928	45.67	10.13	9.06%	0.51%
Q5 (Highest)	4927	56.19	15.27	12.21%	2.25%

*Amounts displayed are in grams unless otherwise specified

† Expressed in quintiles as a percentage of total daily energy intake (kJ) minus alcohol from Q1 (Lowest) to Q5(Highest)
Abbreviations: g/d = grams per day; MUFA = monounsaturated fatty acids; n-3 = omega-3; n-6 = omega-6; PUFA = polyunsaturated fatty acids; Q = quintile; SD = standard deviations; SFA = saturated fatty acids.

Supplemental Table II. Geometric mean concentrations of total cholesterol (mmol/L) by percentage intake of macronutrients.

Nutrient	N	Geometric mean in mmol/L (95%	Relative geometric mean	p-trend
Total carbohydrates				
Q1 (Lowest)	4928	5.89 (5.86 - 5.93)	1.00 (0.99 - 1.01)	<0.000
Q2	4928	5.80 (5.78 - 5.83)	0.98 (0.98 - 0.99)	
Q3	4928	5.82 (5.79 - 5.85)	0.99 (0.98 - 0.99)	
Q4	4928	5.76 (5.73 - 5.78)	0.98 (0.97 - 0.98)	
Q5 (Highest)	4927	5.69 (5.66 - 5.72)	0.97 (0.96 - 0.97)	
Total sugars				
Q1 (Lowest)	4928	5.82 (5.79 - 5.84)	1.00 (1.00 - 1.00)	0.0139
Q2	4928	5.81 (5.78 - 5.83)	1.00 (0.99 - 1.00)	
Q3	4928	5.80 (5.77 - 5.82)	1.00 (0.99 - 1.00)	
Q4	4928	5.78 (5.75 - 5.80)	0.99 (0.99 - 1.00)	
Q5 (Highest)	4927	5.77 (5.74 - 5.80)	0.99 (0.99 - 1.00)	
Free sugars				
Q1 (Lowest)	4928	5.78 (5.75 - 5.80)	1.00 (1.00 - 1.00)	0.0981
Q2	4928	5.78 (5.76 - 5.81)	1.00 (1.00 - 1.01)	
Q3	4928	5.80 (5.78 - 5.83)	1.00 (1.00 - 1.01)	
Q4	4928	5.79 (5.77 - 5.82)	1.00 (1.00 - 1.01)	
Q5 (Highest)	4927	5.81 (5.78 - 5.84)	1.01 (1.00 - 1.01)	
Non-free sugars				
Q1 (Lowest)	4928	5.86 (5.83 - 5.89)	1.00 (1.00 - 1.00)	<0.000
Q2	4928	5.79 (5.76 - 5.82)	0.99 (0.98 - 0.99)	
Q3	4928	5.78 (5.76 - 5.81)	0.99 (0.98 - 0.99)	
Q4	4928	5.79 (5.76 - 5.81)	0.99 (0.98 - 0.99)	
Q5 (Highest)	4927	5.74 (5.71 - 5.77)	0.98 (0.97 - 0.98)	
Total starch				
Q1 (Lowest)	4928	5.86 (5.83 - 5.89)	1.00 (1.00 - 1.00)	<0.000
Q2	4928	5.83 (5.80 - 5.86)	0.99 (0.99 - 1.00)	
Q3	4928	5.79 (5.76 - 5.82)	0.99 (0.98 - 0.99)	
Q4	4928	5.78 (5.75 - 5.81)	0.99 (0.98 - 0.99)	
Q5 (Highest)	4927	5.71 (5.68 - 5.74)	0.97 (0.97 - 0.98)	
Starch from wholegrains				
Q1 (Lowest)	4928	5.84 (5.81 - 5.87)	1.00 (1.00 - 1.00)	<0.000
Q2	4928	5.82 (5.79 - 5.84)	1.00 (0.99 - 1.00)	
Q3	4928	5.79 (5.76 - 5.82)	0.99 (0.99 - 1.00)	
Q4	4928	5.78 (5.75 - 5.81)	0.99 (0.98 - 0.99)	
Q5 (Highest)	4927	5.74 (5.71 - 5.77)	0.98 (0.98 - 0.99)	
Starch from refined				
Q1 (Lowest)	4928	5.81 (5.79 - 5.84)	1.00 (1.00 - 1.00)	0.0035
Q2	4928	5.82 (5.80 - 5.85)	1.00 (1.00 - 1.01)	
Q3	4928	5.79 (5.76 - 5.81)	1.00 (0.99 - 1.00)	
Q4	4928	5.77 (5.75 - 5.80)	0.99 (0.99 - 1.00)	
Q5 (Highest)	4927	5.77 (5.74 - 5.80)	0.99 (0.99 - 1.00)	
Fiber*				
Q1 (Lowest)	4928	5.87 (5.84 - 5.90)	1.00 (0.99 - 1.01)	<0.000
Q2	4928	5.82 (5.79 - 5.85)	0.99 (0.99 - 1.00)	
Q3	4928	5.79 (5.76 - 5.82)	0.99 (0.98 - 0.99)	
Q4	4928	5.78 (5.75 - 5.80)	0.98 (0.98 - 0.99)	
Q5 (Highest)	4927	5.71 (5.68 - 5.74)	0.97 (0.97 - 0.98)	
Total fat				
Q1 (Lowest)	4928	5.70 (5.67 - 5.73)	1.00 (1.00 - 1.00)	<0.000
Q2	4928	5.77 (5.74 - 5.79)	1.01 (1.01 - 1.02)	
Q3	4928	5.80 (5.77 - 5.83)	1.02 (1.01 - 1.02)	
Q4	4928	5.83 (5.80 - 5.86)	1.02 (1.02 - 1.03)	
Q5 (Highest)	4927	5.87 (5.84 - 5.90)	1.03 (1.02 - 1.03)	
Fat from plant sources				
Q1 (Lowest)	4928	5.81 (5.78 - 5.83)	1.00 (1.00 - 1.00)	0.0097
Q2	4928	5.81 (5.79 - 5.84)	1.00 (1.00 - 1.01)	
Q3	4928	5.80 (5.77 - 5.82)	1.00 (0.99 - 1.00)	
Q4	4928	5.80 (5.77 - 5.82)	1.00 (0.99 - 1.00)	
Q5 (Highest)	4927	5.76 (5.73 - 5.78)	0.99 (0.99 - 1.00)	
Fat from animal sources				
Q1 (Lowest)	4928	5.70 (5.68 - 5.73)	1.00 (1.00 - 1.00)	<0.000
Q2	4928	5.74 (5.71 - 5.76)	1.01 (1.00 - 1.01)	
Q3	4928	5.79 (5.76 - 5.82)	1.01 (1.01 - 1.02)	
Q4	4928	5.84 (5.81 - 5.86)	1.02 (1.02 - 1.03)	
Q5 (Highest)	4927	5.90 (5.87 - 5.93)	1.03 (1.03 - 1.04)	
Fat from dairy				

Nutrient	N	Geometric mean in mmol/L (95%	Relative geometric mean	p-trend
Dietary cholesterol (mg)				
Q1 (Lowest)	4928	5.72 (5.69 - 5.75)	1.00 (1.00 - 1.00)	<0.00
Q2	4928	5.82 (5.79 - 5.85)	1.02 (1.01 - 1.02)	
Q3	4928	5.80 (5.78 - 5.83)	1.01 (1.01 - 1.02)	
Q4	4928	5.80 (5.77 - 5.83)	1.01 (1.01 - 1.02)	
Q5 (Highest)	4927	5.82 (5.80 - 5.85)	1.02 (1.01 - 1.02)	
Trans fatty acids				
Q1 (Lowest)	4928	5.74 (5.71 - 5.77)	1.00 (0.99 - 1.01)	<0.00
Q2	4928	5.74 (5.71 - 5.77)	1.00 (1.00 - 1.01)	
Q3	4928	5.78 (5.75 - 5.81)	1.01 (1.00 - 1.01)	
Q4	4928	5.83 (5.80 - 5.87)	1.02 (1.01 - 1.02)	
Q5 (Highest)	4927	5.86 (5.83 - 5.89)	1.02 (1.02 - 1.03)	
SFA				
Q1 (Lowest)	4928	5.69 (5.66 - 5.71)	1.00 (1.00 - 1.00)	<0.00
Q2	4928	5.76 (5.73 - 5.78)	1.01 (1.01 - 1.02)	
Q3	4928	5.79 (5.77 - 5.82)	1.02 (1.01 - 1.02)	
Q4	4928	5.82 (5.79 - 5.85)	1.02 (1.02 - 1.03)	
Q5 (Highest)	4927	5.91 (5.88 - 5.94)	1.04 (1.03 - 1.04)	
MUFA				
Q1 (Lowest)	4928	5.70 (5.67 - 5.73)	1.00 (1.00 - 1.00)	<0.00
Q2	4928	5.81 (5.78 - 5.84)	1.02 (1.01 - 1.02)	
Q3	4928	5.79 (5.76 - 5.82)	1.02 (1.01 - 1.02)	
Q4	4928	5.83 (5.80 - 5.86)	1.02 (1.02 - 1.03)	
Q5 (Highest)	4927	5.84 (5.81 - 5.86)	1.02 (1.02 - 1.03)	
PUFA				
Q1 (Lowest)	4928	5.79 (5.76 - 5.82)	1.00 (1.00 - 1.00)	0.281
Q2	4928	5.79 (5.76 - 5.82)	1.00 (1.00 - 1.00)	
Q3	4928	5.82 (5.80 - 5.85)	1.01 (1.00 - 1.01)	
Q4	4928	5.81 (5.78 - 5.83)	1.00 (1.00 - 1.01)	
Q5 (Highest)	4927	5.76 (5.73 - 5.78)	0.99 (0.99 - 1.00)	
n-3 fatty acids				
Q1 (Lowest)	4928	5.77 (5.75 - 5.80)	1.00 (1.00 - 1.00)	0.046
Q2	4928	5.80 (5.77 - 5.83)	1.00 (1.00 - 1.01)	
Q3	4928	5.78 (5.76 - 5.81)	1.00 (1.00 - 1.01)	
Q4	4928	5.79 (5.76 - 5.81)	1.00 (1.00 - 1.01)	
Q5 (Highest)	4927	5.82 (5.80 - 5.85)	1.01 (1.00 - 1.01)	
n-6 fatty acids				
Q1 (Lowest)	4928	5.80 (5.77 - 5.83)	1.00 (1.00 - 1.00)	0.030
Q2	4928	5.80 (5.77 - 5.82)	1.00 (0.99 - 1.00)	
Q3	4928	5.82 (5.79 - 5.84)	1.00 (1.00 - 1.01)	
Q4	4928	5.81 (5.78 - 5.83)	1.00 (1.00 - 1.01)	
Q5 (Highest)	4927	5.75 (5.72 - 5.78)	0.99 (0.99 - 1.00)	
Total protein				
Q1 (Lowest)	4928	5.77 (5.74 - 5.80)	1.00 (1.00 - 1.00)	0.171
Q2	4928	5.79 (5.76 - 5.82)	1.00 (1.00 - 1.01)	
Q3	4928	5.81 (5.78 - 5.84)	1.01 (1.00 - 1.01)	
Q4	4928	5.81 (5.78 - 5.83)	1.01 (1.00 - 1.01)	
Q5 (Highest)	4927	5.79 (5.76 - 5.82)	1.00 (1.00 - 1.01)	
Protein from plant				
Q1 (Lowest)	4928	5.86 (5.83 - 5.89)	1.00 (1.00 - 1.00)	<0.00
Q2	4928	5.84 (5.81 - 5.86)	1.00 (0.99 - 1.00)	
Q3	4928	5.78 (5.76 - 5.81)	0.99 (0.98 - 0.99)	
Q4	4928	5.79 (5.76 - 5.82)	0.99 (0.98 - 0.99)	
Q5 (Highest)	4927	5.70 (5.67 - 5.73)	0.97 (0.97 - 0.98)	
Protein from animal sources				
Q1 (Lowest)	4928	5.73 (5.70 - 5.76)	1.00 (1.00 - 1.00)	<0.00
Q2	4928	5.78 (5.75 - 5.80)	1.01 (1.00 - 1.01)	
Q3	4928	5.82 (5.80 - 5.85)	1.02 (1.01 - 1.02)	
Q4	4928	5.83 (5.80 - 5.85)	1.02 (1.01 - 1.02)	
Q5 (Highest)	4927	5.81 (5.78 - 5.84)	1.01 (1.01 - 1.02)	
Protein from dairy				
Q1 (Lowest)	4928	5.81 (5.78 - 5.84)	1.00 (1.00 - 1.00)	0.180
Q2	4928	5.79 (5.77 - 5.82)	1.00 (0.99 - 1.00)	
Q3	4928	5.79 (5.76 - 5.81)	1.00 (0.99 - 1.00)	
Q4	4928	5.80 (5.77 - 5.83)	1.00 (0.99 - 1.00)	
Q5 (Highest)	4927	5.77 (5.75 - 5.80)	0.99 (0.99 - 1.00)	
Protein from non-dairy animal sources				

Q1 (Lowest)	4928	5.76 (5.73 - 5.78)	1.00 (1.00 - 1.00)	<0.000	Q1 (Lowest)	4928	5.72 (5.69 - 5.75)	1.00 (1.00 - 1.00)	<0.00
Q2	4928	5.76 (5.73 - 5.79)	1.00 (1.00 - 1.01)		Q2	4928	5.79 (5.76 - 5.82)	1.01 (1.01 - 1.02)	
Q3	4928	5.79 (5.77 - 5.82)	1.01 (1.00 - 1.01)		Q3	4928	5.82 (5.79 - 5.84)	1.02 (1.01 - 1.02)	
Q4	4928	5.81 (5.79 - 5.84)	1.01 (1.00 - 1.01)		Q4	4928	5.83 (5.81 - 5.86)	1.02 (1.02 - 1.03)	
Q5 (Highest)	4927	5.84 (5.82 - 5.87)	1.02 (1.01 - 1.02)		Q5 (Highest)	4927	5.80 (5.78 - 5.83)	1.02 (1.01 - 1.02)	
Fat from non-dairy animal sources									
Q1 (Lowest)	4928	5.71 (5.68 - 5.74)	1.00 (1.00 - 1.00)	<0.000					
Q2	4928	5.78 (5.76 - 5.81)	1.01 (1.01 - 1.02)						
Q3	4928	5.78 (5.75 - 5.81)	1.01 (1.01 - 1.02)						
Q4	4928	5.82 (5.80 - 5.85)	1.02 (1.01 - 1.02)						
Q5 (Highest)	4927	5.87 (5.84 - 5.90)	1.03 (1.02 - 1.03)						

Models adjusted for age at recruitment (<45, 45-49, 50-54, 55-59, 60-64, >65), sex, ethnicity (white, mixed race, Asian or Asian British, black or black British, other, unknown), region (London, North-West England, North-Eastern England, Yorkshire & the Humber, West Midlands, East Midlands, South-West England, Wales), Townsend deprivation index (quintiles from least to most affluent, unknown), smoking status (never, previous, <15 cigarettes/day, 15-29 cigarettes/day, >30 cigarettes/day, unknown), physical activity (low, medium or high according to MET hours per week, unknown), alcohol (<1, 1-9, 10-19, 20-29, >30 g/day, unknown), body mass index (<20, 20-22.4, 22.5-24.9, 25-27.4, 27.5-29.9, 30-32.4, 32.5-34.9, 35-37.4, 37.5-39.9 or >40 in kg/m^2), height (sex-specific groups in 5cm increments, unknown), diabetes diagnosed by a doctor [illegible]

Abbreviations: CI = confidence interval; g/day = grams per day; MUFA = monounsaturated fatty acids; n=3 = omega-3; n-6 = omega-6; PUFA = polyunsaturated fatty acids; Q = quintile; SFA = saturated fatty acids.

*For fiber Q1 and Q5 represent quintiles of total fiber intake (g/day).

p-trend was calculated by modelling quintiles of macronutrient intake as continuous variables in the regression model.

Supplemental Table III. Geometric mean concentrations of LDL-C (mmol/L) by percentage intake of macronutrients.

Nutrient	N	Geometric mean in mmol/L (95%	Relative geometric mean	p-trend
Total carbohydrates				
Q1 (Lowest)	4928	3.65 (3.62 - 3.67)	1.00 (0.99 - 1.01)	<0.000
Q2	4928	3.60 (3.58 - 3.62)	0.99 (0.98 - 0.99)	
Q3	4928	3.63 (3.61 - 3.65)	1.00 (0.99 - 1.00)	
Q4	4928	3.59 (3.57 - 3.61)	0.98 (0.98 - 0.99)	
Q5 (Highest)	4927	3.54 (3.52 - 3.57)	0.97 (0.97 - 0.98)	
Total sugars				
Q1 (Lowest)	4928	3.60 (3.58 - 3.62)	1.00 (0.99 - 1.01)	0.8907
Q2	4928	3.60 (3.58 - 3.62)	1.00 (0.99 - 1.01)	
Q3	4928	3.60 (3.58 - 3.62)	1.00 (0.99 - 1.01)	
Q4	4928	3.60 (3.58 - 3.62)	1.00 (0.99 - 1.01)	
Q5 (Highest)	4927	3.60 (3.58 - 3.63)	1.00 (1.00 - 1.01)	
Free sugars				
Q1 (Lowest)	4928	3.57 (3.55 - 3.59)	1.00 (0.99 - 1.01)	<0.000
Q2	4928	3.59 (3.57 - 3.61)	1.00 (1.00 - 1.01)	
Q3	4928	3.61 (3.59 - 3.63)	1.01 (1.00 - 1.02)	
Q4	4928	3.61 (3.59 - 3.63)	1.01 (1.01 - 1.02)	
Q5 (Highest)	4927	3.63 (3.61 - 3.65)	1.02 (1.01 - 1.02)	
Non-free sugars				
Q1 (Lowest)	4928	3.65 (3.62 - 3.67)	1.00 (0.99 - 1.01)	<0.000
Q2	4928	3.60 (3.58 - 3.62)	0.99 (0.98 - 0.99)	
Q3	4928	3.60 (3.57 - 3.62)	0.99 (0.98 - 0.99)	
Q4	4928	3.60 (3.58 - 3.62)	0.99 (0.98 - 0.99)	
Q5 (Highest)	4927	3.57 (3.55 - 3.59)	0.98 (0.97 - 0.98)	
Total starch				
Q1 (Lowest)	4928	3.64 (3.62 - 3.66)	1.00 (0.99 - 1.01)	<0.000
Q2	4928	3.62 (3.60 - 3.64)	1.00 (0.99 - 1.00)	
Q3	4928	3.60 (3.58 - 3.62)	0.99 (0.98 - 1.00)	
Q4	4928	3.60 (3.58 - 3.62)	0.99 (0.98 - 0.99)	
Q5 (Highest)	4927	3.55 (3.52 - 3.57)	0.97 (0.97 - 0.98)	
Starch from wholegrains				
Q1 (Lowest)	4928	3.64 (3.62 - 3.66)	1.00 (0.99 - 1.01)	<0.000
Q2	4928	3.62 (3.60 - 3.64)	0.99 (0.99 - 1.00)	
Q3	4928	3.60 (3.58 - 3.62)	0.99 (0.98 - 0.99)	
Q4	4928	3.59 (3.57 - 3.61)	0.99 (0.98 - 0.99)	
Q5 (Highest)	4927	3.56 (3.54 - 3.58)	0.98 (0.97 - 0.98)	
Starch from refined				
Q1 (Lowest)	4928	3.60 (3.58 - 3.63)	1.00 (0.99 - 1.01)	0.0547
Q2	4928	3.62 (3.60 - 3.64)	1.01 (1.00 - 1.01)	
Q3	4928	3.60 (3.58 - 3.62)	1.00 (0.99 - 1.01)	
Q4	4928	3.60 (3.57 - 3.62)	1.00 (0.99 - 1.00)	
Q5 (Highest)	4927	3.58 (3.56 - 3.61)	0.99 (0.99 - 1.00)	
Fiber				
Q1 (Lowest)	4928	3.66 (3.63 - 3.68)	1.00 (0.99 - 1.01)	<0.000
Q2	4928	3.62 (3.60 - 3.64)	0.99 (0.98 - 1.00)	
Q3	4928	3.60 (3.58 - 3.62)	0.98 (0.98 - 0.99)	
Q4	4928	3.59 (3.57 - 3.61)	0.98 (0.97 - 0.99)	
Q5 (Highest)	4927	3.54 (3.52 - 3.57)	0.97 (0.96 - 0.97)	
Total fat				
Q1 (Lowest)	4928	3.54 (3.52 - 3.56)	1.00 (0.99 - 1.01)	<0.000
Q2	4928	3.58 (3.56 - 3.61)	1.01 (1.01 - 1.02)	
Q3	4928	3.61 (3.59 - 3.63)	1.02 (1.01 - 1.02)	
Q4	4928	3.63 (3.61 - 3.65)	1.03 (1.02 - 1.03)	
Q5 (Highest)	4927	3.64 (3.62 - 3.67)	1.03 (1.02 - 1.04)	
Fat from plant sources				
Q1 (Lowest)	4928	3.61 (3.59 - 3.63)	1.00 (0.99 - 1.01)	0.0037
Q2	4928	3.62 (3.60 - 3.64)	1.00 (1.00 - 1.01)	
Q3	4928	3.61 (3.59 - 3.63)	1.00 (0.99 - 1.00)	
Q4	4928	3.60 (3.58 - 3.62)	1.00 (0.99 - 1.00)	
Q5 (Highest)	4927	3.57 (3.55 - 3.59)	0.99 (0.98 - 0.99)	
Fat from animal sources				
Q1 (Lowest)	4928	3.54 (3.52 - 3.56)	1.00 (0.99 - 1.01)	<0.000
Q2	4928	3.57 (3.55 - 3.59)	1.01 (1.00 - 1.01)	
Q3	4928	3.59 (3.57 - 3.62)	1.02 (1.01 - 1.02)	
Q4	4928	3.64 (3.62 - 3.66)	1.03 (1.02 - 1.03)	
Q5 (Highest)	4927	3.67 (3.65 - 3.69)	1.04 (1.03 - 1.04)	
Fat from dairy				

Nutrient	N	Geometric mean in mmol/L (95%	Relative geometric mean	p-trend
Dietary cholesterol (mg)				
Q1 (Lowest)	4928	3.55 (3.53 - 3.57)	1.00 (0.99 - 1.01)	0.004
Q2	4928	3.63 (3.61 - 3.65)	1.02 (1.02 - 1.03)	
Q3	4928	3.61 (3.59 - 3.63)	1.02 (1.01 - 1.02)	
Q4	4928	3.60 (3.58 - 3.63)	1.01 (1.01 - 1.02)	
Q5 (Highest)	4927	3.61 (3.59 - 3.63)	1.02 (1.01 - 1.02)	
Trans fatty acids				
Q1 (Lowest)	4928	3.57 (3.55 - 3.59)	1.00 (0.99 - 1.01)	<0.00
Q2	4928	3.57 (3.54 - 3.59)	1.00 (0.99 - 1.01)	
Q3	4928	3.59 (3.57 - 3.62)	1.01 (1.00 - 1.01)	
Q4	4928	3.63 (3.61 - 3.66)	1.02 (1.01 - 1.02)	
Q5 (Highest)	4927	3.65 (3.63 - 3.67)	1.02 (1.02 - 1.03)	
SFA				
Q1 (Lowest)	4928	3.52 (3.50 - 3.54)	1.00 (0.99 - 1.01)	<0.00
Q2	4928	3.58 (3.56 - 3.60)	1.02 (1.01 - 1.02)	
Q3	4928	3.61 (3.59 - 3.63)	1.02 (1.02 - 1.03)	
Q4	4928	3.62 (3.60 - 3.64)	1.03 (1.02 - 1.04)	
Q5 (Highest)	4927	3.69 (3.67 - 3.71)	1.05 (1.04 - 1.05)	
MUFA				
Q1 (Lowest)	4928	3.54 (3.52 - 3.57)	1.00 (0.99 - 1.01)	<0.00
Q2	4928	3.62 (3.60 - 3.64)	1.02 (1.01 - 1.03)	
Q3	4928	3.60 (3.58 - 3.62)	1.01 (1.01 - 1.02)	
Q4	4928	3.63 (3.61 - 3.65)	1.02 (1.02 - 1.03)	
Q5 (Highest)	4927	3.62 (3.60 - 3.64)	1.02 (1.02 - 1.03)	
PUFA				
Q1 (Lowest)	4928	3.61 (3.59 - 3.63)	1.00 (0.99 - 1.01)	0.006
Q2	4928	3.60 (3.58 - 3.62)	1.00 (0.99 - 1.00)	
Q3	4928	3.62 (3.60 - 3.65)	1.00 (1.00 - 1.01)	
Q4	4928	3.60 (3.58 - 3.63)	1.00 (0.99 - 1.00)	
Q5 (Highest)	4927	3.56 (3.54 - 3.58)	0.99 (0.98 - 0.99)	
n-3 fatty acids				
Q1 (Lowest)	4928	3.60 (3.58 - 3.62)	1.00 (0.99 - 1.01)	0.834
Q2	4928	3.61 (3.59 - 3.63)	1.00 (1.00 - 1.01)	
Q3	4928	3.59 (3.57 - 3.62)	1.00 (0.99 - 1.00)	
Q4	4928	3.60 (3.58 - 3.62)	1.00 (0.99 - 1.01)	
Q5 (Highest)	4927	3.61 (3.59 - 3.63)	1.00 (1.00 - 1.01)	
n-6 fatty acids				
Q1 (Lowest)	4928	3.62 (3.60 - 3.64)	1.00 (0.99 - 1.01)	0.000
Q2	4928	3.61 (3.59 - 3.63)	1.00 (0.99 - 1.00)	
Q3	4928	3.62 (3.60 - 3.64)	1.00 (0.99 - 1.01)	
Q4	4928	3.60 (3.58 - 3.63)	1.00 (0.99 - 1.00)	
Q5 (Highest)	4927	3.56 (3.54 - 3.58)	0.98 (0.98 - 0.99)	
Total protein				
Q1 (Lowest)	4928	3.60 (3.57 - 3.62)	1.00 (0.99 - 1.01)	0.814
Q2	4928	3.59 (3.57 - 3.62)	1.00 (0.99 - 1.01)	
Q3	4928	3.61 (3.59 - 3.64)	1.01 (1.00 - 1.01)	
Q4	4928	3.61 (3.59 - 3.63)	1.00 (1.00 - 1.01)	
Q5 (Highest)	4927	3.59 (3.57 - 3.61)	1.00 (0.99 - 1.00)	
Protein from plant				
Q1 (Lowest)	4928	3.65 (3.63 - 3.67)	1.00 (0.99 - 1.01)	<0.00
Q2	4928	3.63 (3.61 - 3.65)	1.00 (0.99 - 1.00)	
Q3	4928	3.60 (3.58 - 3.62)	0.99 (0.98 - 0.99)	
Q4	4928	3.60 (3.58 - 3.62)	0.99 (0.98 - 0.99)	
Q5 (Highest)	4927	3.53 (3.51 - 3.55)	0.97 (0.96 - 0.97)	
Protein from animal sources				
Q1 (Lowest)	4928	3.56 (3.54 - 3.58)	1.00 (0.99 - 1.01)	0.000
Q2	4928	3.59 (3.57 - 3.61)	1.01 (1.00 - 1.01)	
Q3	4928	3.62 (3.60 - 3.64)	1.02 (1.01 - 1.02)	
Q4	4928	3.62 (3.60 - 3.64)	1.02 (1.01 - 1.02)	
Q5 (Highest)	4927	3.61 (3.59 - 3.63)	1.01 (1.01 - 1.02)	
Protein from dairy				
Q1 (Lowest)	4928	3.61 (3.59 - 3.64)	1.00 (0.99 - 1.01)	0.315
Q2	4928	3.59 (3.57 - 3.62)	0.99 (0.99 - 1.00)	
Q3	4928	3.60 (3.58 - 3.62)	1.00 (0.99 - 1.00)	
Q4	4928	3.61 (3.59 - 3.63)	1.00 (0.99 - 1.01)	
Q5 (Highest)	4927	3.59 (3.57 - 3.61)	0.99 (0.99 - 1.00)	
Protein from non-dairy animal sources				

Q1 (Lowest)	4928	3.57 (3.55 - 3.60)	1.00 (0.99 - 1.01)	<0.000	Q1 (Lowest)	4928	3.56 (3.54 - 3.58)	1.00 (0.99 - 1.01)	0.000
Q2	4928	3.57 (3.55 - 3.60)	1.00 (0.99 - 1.01)		Q2	4928	3.60 (3.58 - 3.62)	1.01 (1.01 - 1.02)	
Q3	4928	3.60 (3.58 - 3.63)	1.01 (1.00 - 1.01)		Q3	4928	3.62 (3.60 - 3.64)	1.02 (1.01 - 1.02)	
Q4	4928	3.62 (3.59 - 3.64)	1.01 (1.01 - 1.02)		Q4	4928	3.63 (3.61 - 3.65)	1.02 (1.02 - 1.03)	
Q5 (Highest)	4927	3.64 (3.62 - 3.66)	1.02 (1.01 - 1.02)		Q5 (Highest)	4927	3.60 (3.58 - 3.62)	1.01 (1.01 - 1.02)	
Fat from non-dairy animal sources									
Q1 (Lowest)	4928	3.54 (3.52 - 3.57)	1.00 (0.99 - 1.01)	<0.000					
Q2	4928	3.60 (3.58 - 3.62)	1.02 (1.01 - 1.02)						
Q3	4928	3.59 (3.57 - 3.61)	1.01 (1.01 - 1.02)						
Q4	4928	3.63 (3.60 - 3.65)	1.02 (1.02 - 1.03)						
Q5 (Highest)	4927	3.65 (3.63 - 3.67)	1.03 (1.02 - 1.04)						

Models adjusted for age at recruitment (<45, 45-49, 50-54, 55-59, 60-64, $\geq$65), sex, ethnicity (white, mixed race, Asian or Asian British, black or black British, other, unknown), region (London, North-West England, North-Eastern England, Yorkshire & the Humber, West Midlands, East Midlands, South-West England, Wales), Townsend deprivation index (quintiles from least to most affluent, unknown), smoking status (never, previous, <15 cigarettes/day, 15-29 cigarettes/day, $\geq$30 cigarettes/day, unknown), physical activity (low, medium or high according to MET hours per week, unknown), alcohol (<1, 1-9, 10-19, 20-29, $\geq$30 g/day, unknown), body mass index (<20, 20-22.4, 22.5-24.9, 25-27.4, 27.5-29.9, 30-32.4, 32.5-34.9, 35-37.4, 37.5-39.9 or $\geq$40 in kg/m^2), height (sex-specific groups in 5cm increments, unknown), diabetes diagnosed by a doctor

Abbreviations: CI = confidence interval; g/day = grams per day; LDL-C = low-density lipoprotein cholesterol; MUFA = monounsaturated fatty acids; n=3 = omega-3; n-6 = omega-6; PUFA = polyunsaturated fatty acids; Q = quintile; SFA = saturated fatty acids.

*For fiber Q1 and Q5 represent quintiles of total fiber intake (g/day).

p-trend was calculated by modelling quintiles of macronutrient intake as continuous variables in the regression model.

Supplemental Table IV. Geometric mean concentrations of HDL-C (mmol/L) by percentage intake of macronutrients.

Nutrient	N	Geometric mean in mmol/L (95%	Relative geometric mean	p-trend	Nutrient	N	Geometric mean in mmol/L (95%	Relative geometric mean	p-trend
Total carbohydrates					Dietary cholesterol (mg)				
Q1 (Lowest)	4928	1.54 (1.53 - 1.55)	1.00 (0.99 - 1.01)	<0.000	Q1 (Lowest)	4928	1.46 (1.45 - 1.46)	1.00 (0.99 - 1.01)	<0.00
Q2	4928	1.49 (1.48 - 1.50)	0.97 (0.96 - 0.97)		Q2	4928	1.47 (1.46 - 1.48)	1.01 (1.01 - 1.02)	
Q3	4928	1.47 (1.47 - 1.48)	0.96 (0.95 - 0.96)		Q3	4928	1.48 (1.47 - 1.49)	1.02 (1.01 - 1.02)	
Q4	4928	1.45 (1.44 - 1.46)	0.94 (0.94 - 0.95)		Q4	4928	1.48 (1.47 - 1.49)	1.02 (1.01 - 1.02)	
Q5 (Highest)	4927	1.43 (1.42 - 1.44)	0.93 (0.92 - 0.93)		Q5 (Highest)	4927	1.50 (1.49 - 1.51)	1.03 (1.02 - 1.03)	
Total sugars					Trans fatty acids				
Q1 (Lowest)	4928	1.51 (1.50 - 1.51)	1.00 (0.99 - 1.01)	<0.000	Q1 (Lowest)	4928	1.46 (1.45 - 1.47)	1.00 (0.99 - 1.01)	<0.00
Q2	4928	1.49 (1.48 - 1.50)	0.99 (0.99 - 1.00)		Q2	4928	1.46 (1.45 - 1.47)	1.00 (1.00 - 1.01)	
Q3	4928	1.48 (1.47 - 1.48)	0.98 (0.98 - 0.99)		Q3	4928	1.47 (1.46 - 1.48)	1.01 (1.00 - 1.01)	
Q4	4928	1.47 (1.46 - 1.47)	0.97 (0.97 - 0.98)		Q4	4928	1.48 (1.47 - 1.49)	1.01 (1.01 - 1.02)	
Q5 (Highest)	4927	1.45 (1.44 - 1.45)	0.96 (0.96 - 0.97)		Q5 (Highest)	4927	1.50 (1.49 - 1.51)	1.03 (1.02 - 1.04)	
Free sugars					SFA				
Q1 (Lowest)	4928	1.51 (1.50 - 1.51)	1.00 (0.99 - 1.01)	<0.000	Q1 (Lowest)	4928	1.46 (1.45 - 1.47)	1.00 (0.99 - 1.01)	<0.00
Q2	4928	1.49 (1.48 - 1.50)	0.99 (0.98 - 1.00)		Q2	4928	1.46 (1.45 - 1.47)	1.00 (1.00 - 1.01)	
Q3	4928	1.49 (1.48 - 1.49)	0.99 (0.98 - 0.99)		Q3	4928	1.48 (1.47 - 1.48)	1.01 (1.01 - 1.02)	
Q4	4928	1.46 (1.45 - 1.47)	0.97 (0.97 - 0.98)		Q4	4928	1.48 (1.47 - 1.49)	1.02 (1.01 - 1.02)	
Q5 (Highest)	4927	1.44 (1.43 - 1.45)	0.96 (0.95 - 0.96)		Q5 (Highest)	4927	1.51 (1.50 - 1.52)	1.03 (1.03 - 1.04)	
Non-free sugars					MUFA				
Q1 (Lowest)	4928	1.48 (1.47 - 1.49)	1.00 (0.99 - 1.01)	0.3221	Q1 (Lowest)	4928	1.45 (1.44 - 1.46)	1.00 (0.99 - 1.01)	<0.00
Q2	4928	1.48 (1.47 - 1.48)	0.99 (0.99 - 1.00)		Q2	4928	1.47 (1.46 - 1.48)	1.02 (1.01 - 1.02)	
Q3	4928	1.48 (1.47 - 1.48)	0.99 (0.99 - 1.00)		Q3	4928	1.48 (1.47 - 1.49)	1.02 (1.02 - 1.03)	
Q4	4928	1.48 (1.47 - 1.49)	1.00 (0.99 - 1.00)		Q4	4928	1.49 (1.48 - 1.49)	1.03 (1.02 - 1.03)	
Q5 (Highest)	4927	1.47 (1.47 - 1.48)	0.99 (0.99 - 1.00)		Q5 (Highest)	4927	1.50 (1.50 - 1.51)	1.04 (1.03 - 1.04)	
Total starch					PUFA				
Q1 (Lowest)	4928	1.51 (1.50 - 1.52)	1.00 (0.99 - 1.01)	<0.000	Q1 (Lowest)	4928	1.45 (1.45 - 1.46)	1.00 (0.99 - 1.01)	<0.00
Q2	4928	1.49 (1.48 - 1.50)	0.99 (0.98 - 0.99)		Q2	4928	1.47 (1.46 - 1.48)	1.01 (1.00 - 1.02)	
Q3	4928	1.47 (1.47 - 1.48)	0.98 (0.97 - 0.98)		Q3	4928	1.49 (1.48 - 1.49)	1.02 (1.02 - 1.03)	
Q4	4928	1.46 (1.45 - 1.47)	0.97 (0.96 - 0.97)		Q4	4928	1.49 (1.48 - 1.50)	1.02 (1.02 - 1.03)	
Q5 (Highest)	4927	1.46 (1.45 - 1.46)	0.96 (0.96 - 0.97)		Q5 (Highest)	4927	1.49 (1.48 - 1.50)	1.02 (1.02 - 1.03)	
Starch from wholegrains					n-3 fatty acids				
Q1 (Lowest)	4928	1.48 (1.47 - 1.48)	1.00 (0.99 - 1.01)	0.3495	Q1 (Lowest)	4928	1.45 (1.44 - 1.46)	1.00 (0.99 - 1.01)	<0.00
Q2	4928	1.48 (1.47 - 1.49)	1.00 (1.00 - 1.01)		Q2	4928	1.47 (1.47 - 1.48)	1.02 (1.01 - 1.02)	
Q3	4928	1.48 (1.47 - 1.49)	1.00 (1.00 - 1.01)		Q3	4928	1.47 (1.46 - 1.48)	1.01 (1.01 - 1.02)	
Q4	4928	1.48 (1.47 - 1.48)	1.00 (0.99 - 1.00)		Q4	4928	1.48 (1.47 - 1.49)	1.02 (1.01 - 1.02)	
Q5 (Highest)	4927	1.47 (1.47 - 1.48)	1.00 (0.99 - 1.00)		Q5 (Highest)	4927	1.51 (1.50 - 1.52)	1.04 (1.04 - 1.05)	
Starch from refined					n-6 fatty acids				
Q1 (Lowest)	4928	1.50 (1.49 - 1.50)	1.00 (0.99 - 1.01)	<0.000	Q1 (Lowest)	4928	1.46 (1.45 - 1.47)	1.00 (0.99 - 1.01)	<0.00
Q2	4928	1.49 (1.48 - 1.50)	0.99 (0.99 - 1.00)		Q2	4928	1.47 (1.46 - 1.48)	1.01 (1.00 - 1.01)	
Q3	4928	1.47 (1.47 - 1.48)	0.98 (0.98 - 0.99)		Q3	4928	1.49 (1.48 - 1.49)	1.02 (1.01 - 1.02)	
Q4	4928	1.47 (1.46 - 1.47)	0.98 (0.97 - 0.99)		Q4	4928	1.48 (1.48 - 1.49)	1.02 (1.01 - 1.02)	
Q5 (Highest)	4927	1.46 (1.46 - 1.47)	0.98 (0.97 - 0.98)		Q5 (Highest)	4927	1.48 (1.48 - 1.49)	1.02 (1.01 - 1.02)	
Fiber					Total protein				
Q1 (Lowest)	4928	1.48 (1.48 - 1.49)	1.00 (0.99 - 1.01)	0.0118	Q1 (Lowest)	4928	1.46 (1.45 - 1.47)	1.00 (0.99 - 1.01)	<0.00
Q2	4928	1.48 (1.47 - 1.49)	1.00 (0.99 - 1.00)		Q2	4928	1.48 (1.47 - 1.48)	1.01 (1.00 - 1.02)	
Q3	4928	1.48 (1.47 - 1.49)	1.00 (0.99 - 1.00)		Q3	4928	1.48 (1.47 - 1.49)	1.01 (1.01 - 1.02)	
Q4	4928	1.48 (1.47 - 1.49)	0.99 (0.99 - 1.00)		Q4	4928	1.48 (1.47 - 1.49)	1.01 (1.01 - 1.02)	
Q5 (Highest)	4927	1.47 (1.46 - 1.47)	0.99 (0.98 - 0.99)		Q5 (Highest)	4927	1.49 (1.48 - 1.50)	1.02 (1.01 - 1.02)	
Total fat					Protein from plant				
Q1 (Lowest)	4928	1.45 (1.44 - 1.46)	1.00 (0.99 - 1.01)	<0.000	Q1 (Lowest)	4928	1.49 (1.49 - 1.50)	1.00 (0.99 - 1.01)	<0.00
Q2	4928	1.46 (1.45 - 1.47)	1.01 (1.00 - 1.01)		Q2	4928	1.48 (1.48 - 1.49)	0.99 (0.99 - 1.00)	
Q3	4928	1.47 (1.47 - 1.48)	1.02 (1.01 - 1.02)		Q3	4928	1.47 (1.46 - 1.48)	0.98 (0.98 - 0.99)	
Q4	4928	1.49 (1.48 - 1.50)	1.03 (1.02 - 1.03)		Q4	4928	1.48 (1.47 - 1.49)	0.99 (0.98 - 0.99)	
Q5 (Highest)	4927	1.51 (1.51 - 1.52)	1.05 (1.04 - 1.05)		Q5 (Highest)	4927	1.46 (1.45 - 1.47)	0.98 (0.97 - 0.98)	
Fat from plant sources					Protein from animal sources				
Q1 (Lowest)	4928	1.48 (1.47 - 1.49)	1.00 (0.99 - 1.01)	0.6228	Q1 (Lowest)	4928	1.46 (1.45 - 1.46)	1.00 (0.99 - 1.01)	<0.00
Q2	4928	1.47 (1.47 - 1.48)	1.00 (0.99 - 1.00)		Q2	4928	1.48 (1.47 - 1.49)	1.01 (1.01 - 1.02)	
Q3	4928	1.47 (1.47 - 1.48)	1.00 (0.99 - 1.00)		Q3	4928	1.48 (1.47 - 1.49)	1.02 (1.01 - 1.02)	
Q4	4928	1.48 (1.47 - 1.49)	1.00 (1.00 - 1.01)		Q4	4928	1.48 (1.47 - 1.49)	1.02 (1.01 - 1.02)	
Q5 (Highest)	4927	1.48 (1.47 - 1.49)	1.00 (0.99 - 1.00)		Q5 (Highest)	4927	1.49 (1.49 - 1.50)	1.03 (1.02 - 1.03)	
Fat from animal sources					Protein from dairy				
Q1 (Lowest)	4928	1.45 (1.44 - 1.46)	1.00 (0.99 - 1.01)	<0.000	Q1 (Lowest)	4928	1.47 (1.46 - 1.48)	1.00 (0.99 - 1.01)	0.078
Q2	4928	1.46 (1.45 - 1.47)	1.01 (1.00 - 1.01)		Q2	4928	1.48 (1.47 - 1.49)	1.00 (1.00 - 1.01)	
Q3	4928	1.48 (1.47 - 1.49)	1.02 (1.01 - 1.02)		Q3	4928	1.47 (1.46 - 1.48)	1.00 (0.99 - 1.01)	
Q4	4928	1.48 (1.47 - 1.49)	1.02 (1.01 - 1.03)		Q4	4928	1.48 (1.47 - 1.49)	1.01 (1.00 - 1.01)	
Q5 (Highest)	4927	1.51 (1.51 - 1.52)	1.04 (1.04 - 1.05)		Q5 (Highest)	4927	1.48 (1.47 - 1.49)	1.01 (1.00 - 1.01)	
Fat from dairy					Protein from non-dairy animal sources				

Q1 (Lowest)	4928	1.46 (1.45 - 1.47)	1.00 (0.99 - 1.01)	<0.000	Q1 (Lowest)	4928	1.46 (1.45 - 1.46)	1.00 (0.99 - 1.01)	<0.00
Q2	4928	1.47 (1.46 - 1.48)	1.01 (1.00 - 1.01)		Q2	4928	1.48 (1.47 - 1.49)	1.02 (1.01 - 1.02)	
Q3	4928	1.47 (1.47 - 1.48)	1.01 (1.00 - 1.02)		Q3	4928	1.48 (1.47 - 1.49)	1.02 (1.01 - 1.02)	
Q4	4928	1.48 (1.47 - 1.49)	1.01 (1.01 - 1.02)		Q4	4928	1.48 (1.47 - 1.49)	1.02 (1.01 - 1.02)	
Q5 (Highest)	4927	1.50 (1.49 - 1.51)	1.03 (1.02 - 1.03)		Q5 (Highest)	4927	1.49 (1.48 - 1.50)	1.02 (1.02 - 1.03)	
Fat from non-dairy animal sources									
Q1 (Lowest)	4928	1.46 (1.45 - 1.46)	1.00 (0.99 - 1.01)	<0.000					
Q2	4928	1.47 (1.47 - 1.48)	1.01 (1.01 - 1.02)						
Q3	4928	1.47 (1.46 - 1.48)	1.01 (1.00 - 1.02)						
Q4	4928	1.49 (1.48 - 1.50)	1.02 (1.02 - 1.03)						
Q5 (Highest)	4927	1.50 (1.49 - 1.50)	1.03 (1.02 - 1.03)						

Models adjusted for age at recruitment (<45, 45-49, 50-54, 55-59, 60-64, >65), sex, ethnicity (white, mixed race, Asian or Asian British, black or black British, other, unknown), region (London, North-West England, North-Eastern England, Yorkshire & the Humber, West Midlands, East Midlands, South-West England, Wales), Townsend deprivation index (quintiles from least to most affluent, unknown), smoking status (never, previous, <15 cigarettes/day, 15-29 cigarettes/day, >30 cigarettes/day, unknown), physical activity (low, medium or high according to MET hours per week, unknown), alcohol (<1, 1-9, 10-19, 20-29, >30 g/day, unknown), body mass index (<20, 20-22.4, 22.5-24.9, 25-27.4, 27.5-29.9, 30-32.4, 32.5-34.9, 35-37.4, 37.5-39.9 or >40 in kg/m^2), height (sex-specific groups in 5cm increments, unknown), diabetes diagnosed by a doctor

Abbreviations: CI = confidence interval; g/day = grams per day; HDL-C = high-density lipoprotein cholesterol; MUFA = monounsaturated fatty acids; n=3 = omega-3; n-6 = omega-6; PUFA = polyunsaturated fatty acids; Q = quintile; SFA = saturated fatty acids.

*For fiber Q1 and Q5 represent quintiles of total fiber intake (g/day).

p-trend was calculated by modelling quintiles of macronutrient intake as continuous variables in the regression model.

602

Supplemental Table V. Geometric mean concentrations of triglycerides (mmol/L) by percentage intake of macronutrients.

Nutrient	N	Geometric mean in mmol/L (95%	Relative geometric mean	p-trend
Total carbohydrates				
Q1 (Lowest)	4928	1.33 (1.31 - 1.35)	1.00 (0.99 - 1.01)	<0.000
Q2	4928	1.38 (1.36 - 1.39)	1.04 (1.02 - 1.05)	
Q3	4928	1.41 (1.40 - 1.43)	1.06 (1.05 - 1.08)	
Q4	4928	1.45 (1.43 - 1.47)	1.09 (1.08 - 1.11)	
Q5 (Highest)	4927	1.47 (1.45 - 1.49)	1.11 (1.09 - 1.12)	
Total sugars				
Q1 (Lowest)	4928	1.36 (1.35 - 1.38)	1.00 (0.99 - 1.01)	<0.000
Q2	4928	1.40 (1.38 - 1.41)	1.02 (1.01 - 1.04)	
Q3	4928	1.42 (1.40 - 1.43)	1.04 (1.03 - 1.05)	
Q4	4928	1.42 (1.40 - 1.43)	1.04 (1.03 - 1.05)	
Q5 (Highest)	4927	1.45 (1.43 - 1.46)	1.06 (1.05 - 1.07)	
Free sugars				
Q1 (Lowest)	4928	1.34 (1.32 - 1.36)	1.00 (0.99 - 1.01)	<0.000
Q2	4928	1.37 (1.36 - 1.39)	1.02 (1.01 - 1.04)	
Q3	4928	1.39 (1.37 - 1.41)	1.03 (1.02 - 1.05)	
Q4	4928	1.44 (1.42 - 1.46)	1.07 (1.06 - 1.09)	
Q5 (Highest)	4927	1.50 (1.48 - 1.51)	1.11 (1.10 - 1.13)	
Non-free sugars				
Q1 (Lowest)	4928	1.44 (1.42 - 1.46)	1.00 (0.99 - 1.01)	<0.000
Q2	4928	1.42 (1.40 - 1.44)	0.99 (0.97 - 1.00)	
Q3	4928	1.40 (1.39 - 1.42)	0.97 (0.96 - 0.99)	
Q4	4928	1.41 (1.39 - 1.42)	0.98 (0.96 - 0.99)	
Q5 (Highest)	4927	1.37 (1.35 - 1.38)	0.95 (0.94 - 0.96)	
Total starch				
Q1 (Lowest)	4928	1.36 (1.34 - 1.38)	1.00 (0.99 - 1.01)	<0.000
Q2	4928	1.39 (1.38 - 1.41)	1.03 (1.01 - 1.04)	
Q3	4928	1.41 (1.39 - 1.43)	1.04 (1.03 - 1.05)	
Q4	4928	1.44 (1.42 - 1.46)	1.06 (1.05 - 1.07)	
Q5 (Highest)	4927	1.43 (1.41 - 1.45)	1.05 (1.04 - 1.07)	
Starch from wholegrains				
Q1 (Lowest)	4928	1.42 (1.40 - 1.44)	1.00 (0.99 - 1.01)	0.5057
Q2	4928	1.40 (1.38 - 1.42)	0.99 (0.98 - 1.00)	
Q3	4928	1.40 (1.38 - 1.42)	0.99 (0.97 - 1.00)	
Q4	4928	1.41 (1.39 - 1.43)	0.99 (0.98 - 1.00)	
Q5 (Highest)	4927	1.41 (1.39 - 1.42)	0.99 (0.98 - 1.00)	
Starch from refined				
Q1 (Lowest)	4928	1.38 (1.37 - 1.40)	1.00 (0.99 - 1.01)	<0.000
Q2	4928	1.39 (1.37 - 1.41)	1.01 (0.99 - 1.02)	
Q3	4928	1.41 (1.39 - 1.42)	1.02 (1.00 - 1.03)	
Q4	4928	1.42 (1.40 - 1.44)	1.02 (1.01 - 1.04)	
Q5 (Highest)	4927	1.44 (1.42 - 1.45)	1.04 (1.03 - 1.05)	
Fiber				
Q1 (Lowest)	4928	1.42 (1.40 - 1.44)	1.00 (0.99 - 1.01)	0.0063
Q2	4928	1.42 (1.40 - 1.44)	1.00 (0.99 - 1.01)	
Q3	4928	1.41 (1.39 - 1.43)	0.99 (0.98 - 1.01)	
Q4	4928	1.40 (1.38 - 1.41)	0.98 (0.97 - 1.00)	
Q5 (Highest)	4927	1.39 (1.37 - 1.40)	0.98 (0.96 - 0.99)	
Total fat				
Q1 (Lowest)	4928	1.43 (1.41 - 1.45)	1.00 (0.99 - 1.01)	<0.000
Q2	4928	1.44 (1.42 - 1.46)	1.00 (0.99 - 1.02)	
Q3	4928	1.42 (1.41 - 1.44)	0.99 (0.98 - 1.01)	
Q4	4928	1.39 (1.37 - 1.40)	0.97 (0.96 - 0.98)	
Q5 (Highest)	4927	1.36 (1.34 - 1.37)	0.95 (0.93 - 0.96)	
Fat from plant sources				
Q1 (Lowest)	4928	1.40 (1.39 - 1.42)	1.00 (0.99 - 1.01)	0.1492
Q2	4928	1.42 (1.40 - 1.44)	1.01 (1.00 - 1.02)	
Q3	4928	1.42 (1.40 - 1.44)	1.01 (1.00 - 1.02)	
Q4	4928	1.41 (1.39 - 1.43)	1.00 (0.99 - 1.02)	
Q5 (Highest)	4927	1.39 (1.37 - 1.40)	0.99 (0.98 - 1.00)	
Fat from animal sources				
Q1 (Lowest)	4928	1.43 (1.41 - 1.45)	1.00 (0.99 - 1.01)	<0.000
Q2	4928	1.42 (1.40 - 1.44)	1.00 (0.98 - 1.01)	
Q3	4928	1.42 (1.40 - 1.43)	0.99 (0.98 - 1.00)	
Q4	4928	1.41 (1.39 - 1.42)	0.98 (0.97 - 1.00)	
Q5 (Highest)	4927	1.36 (1.35 - 1.38)	0.95 (0.94 - 0.97)	
Fat from dairy				

Nutrient	N	Geometric mean in mmol/L (95%	Relative geometric mean	p-trend
Dietary cholesterol (mg)				
Q1 (Lowest)	4928	1.42 (1.40 - 1.44)	1.00 (0.99 - 1.01)	<0.00
Q2	4928	1.43 (1.41 - 1.44)	1.00 (0.99 - 1.02)	
Q3	4928	1.42 (1.40 - 1.43)	1.00 (0.99 - 1.01)	
Q4	4928	1.40 (1.38 - 1.42)	0.99 (0.97 - 1.00)	
Q5 (Highest)	4927	1.38 (1.36 - 1.39)	0.97 (0.96 - 0.98)	
Trans fatty acids				
Q1 (Lowest)	4928	1.41 (1.39 - 1.43)	1.00 (0.99 - 1.01)	0.593
Q2	4928	1.43 (1.41 - 1.45)	1.01 (0.99 - 1.02)	
Q3	4928	1.42 (1.40 - 1.44)	1.00 (0.99 - 1.02)	
Q4	4928	1.43 (1.41 - 1.45)	1.01 (1.00 - 1.03)	
Q5 (Highest)	4927	1.40 (1.38 - 1.42)	0.99 (0.98 - 1.01)	
SFA				
Q1 (Lowest)	4928	1.41 (1.39 - 1.43)	1.00 (0.99 - 1.01)	0.003
Q2	4928	1.43 (1.41 - 1.44)	1.01 (1.00 - 1.02)	
Q3	4928	1.41 (1.39 - 1.43)	1.00 (0.99 - 1.01)	
Q4	4928	1.42 (1.40 - 1.44)	1.01 (1.00 - 1.02)	
Q5 (Highest)	4927	1.37 (1.35 - 1.39)	0.97 (0.96 - 0.98)	
MUFA				
Q1 (Lowest)	4928	1.43 (1.41 - 1.45)	1.00 (0.99 - 1.01)	<0.00
Q2	4928	1.43 (1.41 - 1.45)	1.00 (0.99 - 1.01)	
Q3	4928	1.41 (1.40 - 1.43)	0.99 (0.98 - 1.00)	
Q4	4928	1.40 (1.38 - 1.41)	0.98 (0.97 - 0.99)	
Q5 (Highest)	4927	1.37 (1.35 - 1.38)	0.96 (0.94 - 0.97)	
PUFA				
Q1 (Lowest)	4928	1.46 (1.44 - 1.48)	1.00 (0.99 - 1.01)	<0.00
Q2	4928	1.41 (1.39 - 1.43)	0.97 (0.95 - 0.98)	
Q3	4928	1.41 (1.39 - 1.43)	0.97 (0.95 - 0.98)	
Q4	4928	1.40 (1.38 - 1.42)	0.96 (0.95 - 0.97)	
Q5 (Highest)	4927	1.36 (1.34 - 1.38)	0.93 (0.92 - 0.95)	
n-3 fatty acids				
Q1 (Lowest)	4928	1.47 (1.45 - 1.48)	1.00 (0.99 - 1.01)	<0.00
Q2	4928	1.43 (1.41 - 1.45)	0.98 (0.96 - 0.99)	
Q3	4928	1.43 (1.42 - 1.45)	0.98 (0.97 - 0.99)	
Q4	4928	1.39 (1.37 - 1.40)	0.95 (0.93 - 0.96)	
Q5 (Highest)	4927	1.32 (1.31 - 1.34)	0.90 (0.89 - 0.91)	
n-6 fatty acids				
Q1 (Lowest)	4928	1.44 (1.42 - 1.46)	1.00 (0.99 - 1.01)	<0.00
Q2	4928	1.41 (1.39 - 1.43)	0.98 (0.97 - 0.99)	
Q3	4928	1.40 (1.38 - 1.42)	0.97 (0.96 - 0.99)	
Q4	4928	1.42 (1.40 - 1.43)	0.99 (0.97 - 1.00)	
Q5 (Highest)	4927	1.37 (1.35 - 1.39)	0.95 (0.94 - 0.97)	
Total protein				
Q1 (Lowest)	4928	1.43 (1.41 - 1.45)	1.00 (0.99 - 1.01)	<0.00
Q2	4928	1.42 (1.40 - 1.44)	0.99 (0.98 - 1.00)	
Q3	4928	1.41 (1.39 - 1.43)	0.98 (0.97 - 1.00)	
Q4	4928	1.41 (1.39 - 1.43)	0.98 (0.97 - 1.00)	
Q5 (Highest)	4927	1.36 (1.34 - 1.38)	0.95 (0.94 - 0.96)	
Protein from plant				
Q1 (Lowest)	4928	1.39 (1.37 - 1.41)	1.00 (0.99 - 1.01)	0.286
Q2	4928	1.41 (1.39 - 1.43)	1.01 (1.00 - 1.03)	
Q3	4928	1.42 (1.40 - 1.43)	1.02 (1.01 - 1.03)	
Q4	4928	1.41 (1.39 - 1.43)	1.01 (1.00 - 1.03)	
Q5 (Highest)	4927	1.41 (1.39 - 1.43)	1.01 (1.00 - 1.02)	
Protein from animal sources				
Q1 (Lowest)	4928	1.43 (1.42 - 1.45)	1.00 (0.99 - 1.01)	<0.00
Q2	4928	1.41 (1.39 - 1.43)	0.98 (0.97 - 0.99)	
Q3	4928	1.41 (1.40 - 1.43)	0.99 (0.97 - 1.00)	
Q4	4928	1.41 (1.40 - 1.43)	0.99 (0.97 - 1.00)	
Q5 (Highest)	4927	1.37 (1.35 - 1.38)	0.95 (0.94 - 0.96)	
Protein from dairy				
Q1 (Lowest)	4928	1.42 (1.40 - 1.44)	1.00 (0.99 - 1.01)	0.000
Q2	4928	1.43 (1.41 - 1.45)	1.01 (0.99 - 1.02)	
Q3	4928	1.41 (1.40 - 1.43)	1.00 (0.98 - 1.01)	
Q4	4928	1.39 (1.37 - 1.41)	0.98 (0.97 - 0.99)	
Q5 (Highest)	4927	1.39 (1.37 - 1.40)	0.98 (0.97 - 0.99)	
Protein from non-dairy animal sources				

Q1 (Lowest)	4928	1.42 (1.40 - 1.44)	1.00 (0.99 - 1.01)	0.0001
Q2	4928	1.43 (1.41 - 1.44)	1.00 (0.99 - 1.02)	
Q3	4928	1.40 (1.39 - 1.42)	0.99 (0.98 - 1.00)	
Q4	4928	1.42 (1.40 - 1.44)	1.00 (0.99 - 1.01)	
Q5 (Highest)	4927	1.37 (1.35 - 1.39)	0.96 (0.95 - 0.98)	
Fat from non-dairy animal sources				
Q1 (Lowest)	4928	1.43 (1.41 - 1.44)	1.00 (0.99 - 1.01)	0.0005
Q2	4928	1.42 (1.40 - 1.43)	0.99 (0.98 - 1.01)	
Q3	4928	1.41 (1.40 - 1.43)	0.99 (0.98 - 1.01)	
Q4	4928	1.39 (1.37 - 1.41)	0.98 (0.96 - 0.99)	
Q5 (Highest)	4927	1.39 (1.37 - 1.41)	0.97 (0.96 - 0.99)	

Q1 (Lowest)	4928	1.43 (1.41 - 1.45)	1.00 (0.99 - 1.01)	0.001
Q2	4928	1.40 (1.38 - 1.42)	0.98 (0.97 - 0.99)	
Q3	4928	1.41 (1.39 - 1.43)	0.99 (0.97 - 1.00)	
Q4	4928	1.41 (1.39 - 1.43)	0.98 (0.97 - 1.00)	
Q5 (Highest)	4927	1.38 (1.36 - 1.40)	0.96 (0.95 - 0.98)	

Models adjusted for age at recruitment (<45, 45-49, 50-54, 55-59, 60-64, >65), sex, ethnicity (white, mixed race, Asian or Asian British, black or black British, other, unknown), region (London, North-West England, North-Eastern England, Yorkshire & the Humber, West Midlands, East Midlands, South-West England, Wales), Townsend deprivation index (quintiles from least to most affluent, unknown), smoking status (never, previous, <15 cigarettes/day, 15-29 cigarettes/day, >30 cigarettes/day, unknown), physical activity (low, medium or high according to MET hours per week, unknown), alcohol (<1, 1-9, 10-19, 20-29, >30 g/day, unknown), body mass index (<20, 20-22.4, 22.5-24.9, 25-27.4, 27.5-29.9, 30-32.4, 32.5-34.9, 35-37.4, 37.5-39.9 or >40 in kg/m^2), height (sex-specific groups in 5cm increments, unknown), diabetes diagnosed by a doctor

Abbreviations: CI = confidence interval; g/day = grams per day; MUFA = monounsaturated fatty acids; n=3 = omega-3; n-6 = omega-6; PUFA = polyunsaturated fatty acids; Q = quintile; SFA = saturated fatty acids.

*For fiber Q1 and Q5 represent quintiles of total fiber intake (g/day).

p-trend was calculated by modelling quintiles of macronutrient intake as continuous variables in the regression model.

Supplemental Table VI. Geometric mean concentrations of TC-to-HDL-C ratio by percentage intake of macronutrients.

Nutrient	N	Geometric mean in mmol/L (95%	Relative geometric mean	p-trend
Total carbohydrates				
Q1 (Lowest)	4928	3.82 (3.80 - 3.85)	1.00 (0.99 - 1.01)	<0.000
Q2	4928	3.89 (3.87 - 3.91)	1.02 (1.01 - 1.02)	
Q3	4928	3.95 (3.92 - 3.97)	1.03 (1.03 - 1.04)	
Q4	4928	3.97 (3.95 - 4.00)	1.04 (1.03 - 1.05)	
Q5 (Highest)	4927	3.98 (3.95 - 4.01)	1.04 (1.03 - 1.05)	
Total sugars				
Q1 (Lowest)	4928	3.86 (3.84 - 3.89)	1.00 (0.99 - 1.01)	<0.000
Q2	4928	3.89 (3.87 - 3.92)	1.01 (1.00 - 1.01)	
Q3	4928	3.93 (3.90 - 3.95)	1.02 (1.01 - 1.02)	
Q4	4928	3.94 (3.92 - 3.96)	1.02 (1.01 - 1.03)	
Q5 (Highest)	4927	3.99 (3.97 - 4.01)	1.03 (1.03 - 1.04)	
Free sugars				
Q1 (Lowest)	4928	3.84 (3.81 - 3.86)	1.00 (0.99 - 1.01)	<0.000
Q2	4928	3.88 (3.86 - 3.90)	1.01 (1.01 - 1.02)	
Q3	4928	3.90 (3.88 - 3.93)	1.02 (1.01 - 1.02)	
Q4	4928	3.96 (3.94 - 3.99)	1.03 (1.03 - 1.04)	
Q5 (Highest)	4927	4.03 (4.00 - 4.05)	1.05 (1.04 - 1.06)	
Non-free sugars				
Q1 (Lowest)	4928	3.95 (3.93 - 3.98)	1.00 (0.99 - 1.01)	0.0028
Q2	4928	3.93 (3.90 - 3.95)	0.99 (0.99 - 1.00)	
Q3	4928	3.92 (3.90 - 3.94)	0.99 (0.99 - 1.00)	
Q4	4928	3.91 (3.89 - 3.94)	0.99 (0.98 - 1.00)	
Q5 (Highest)	4927	3.89 (3.87 - 3.92)	0.99 (0.98 - 0.99)	
Total starch				
Q1 (Lowest)	4928	3.88 (3.86 - 3.91)	1.00 (0.99 - 1.01)	0.0022
Q2	4928	3.92 (3.90 - 3.94)	1.01 (1.00 - 1.02)	
Q3	4928	3.93 (3.90 - 3.95)	1.01 (1.01 - 1.02)	
Q4	4928	3.96 (3.93 - 3.98)	1.02 (1.01 - 1.03)	
Q5 (Highest)	4927	3.92 (3.90 - 3.95)	1.01 (1.00 - 1.02)	
Starch from wholegrains				
Q1 (Lowest)	4928	3.96 (3.93 - 3.98)	1.00 (0.99 - 1.01)	0.0015
Q2	4928	3.92 (3.90 - 3.95)	0.99 (0.99 - 1.00)	
Q3	4928	3.91 (3.89 - 3.94)	0.99 (0.98 - 1.00)	
Q4	4928	3.92 (3.89 - 3.94)	0.99 (0.98 - 1.00)	
Q5 (Highest)	4927	3.90 (3.87 - 3.92)	0.99 (0.98 - 0.99)	
Starch from refined				
Q1 (Lowest)	4928	3.88 (3.86 - 3.91)	1.00 (0.99 - 1.01)	0.0003
Q2	4928	3.92 (3.89 - 3.94)	1.01 (1.00 - 1.01)	
Q3	4928	3.93 (3.90 - 3.95)	1.01 (1.00 - 1.02)	
Q4	4928	3.94 (3.91 - 3.96)	1.01 (1.01 - 1.02)	
Q5 (Highest)	4927	3.94 (3.92 - 3.97)	1.02 (1.01 - 1.02)	
Fiber				
Q1 (Lowest)	4928	3.95 (3.93 - 3.98)	1.00 (0.99 - 1.01)	0.0016
Q2	4928	3.94 (3.91 - 3.96)	1.00 (0.99 - 1.00)	
Q3	4928	3.92 (3.89 - 3.94)	0.99 (0.98 - 1.00)	
Q4	4928	3.91 (3.89 - 3.93)	0.99 (0.98 - 1.00)	
Q5 (Highest)	4927	3.89 (3.87 - 3.92)	0.98 (0.98 - 0.99)	
Total fat				
Q1 (Lowest)	4928	3.94 (3.91 - 3.96)	1.00 (0.99 - 1.01)	0.0002
Q2	4928	3.94 (3.92 - 3.97)	1.00 (1.00 - 1.01)	
Q3	4928	3.94 (3.91 - 3.96)	1.00 (0.99 - 1.01)	
Q4	4928	3.91 (3.89 - 3.94)	0.99 (0.99 - 1.00)	
Q5 (Highest)	4927	3.88 (3.85 - 3.90)	0.98 (0.98 - 0.99)	
Fat from plant sources				
Q1 (Lowest)	4928	3.93 (3.90 - 3.95)	1.00 (0.99 - 1.01)	0.0139
Q2	4928	3.94 (3.92 - 3.97)	1.00 (1.00 - 1.01)	
Q3	4928	3.93 (3.91 - 3.96)	1.00 (1.00 - 1.01)	
Q4	4928	3.91 (3.89 - 3.93)	1.00 (0.99 - 1.00)	
Q5 (Highest)	4927	3.89 (3.87 - 3.92)	0.99 (0.99 - 1.00)	
Fat from animal sources				
Q1 (Lowest)	4928	3.93 (3.90 - 3.95)	1.00 (0.99 - 1.01)	0.2306
Q2	4928	3.93 (3.90 - 3.95)	1.00 (0.99 - 1.01)	
Q3	4928	3.91 (3.89 - 3.94)	1.00 (0.99 - 1.00)	
Q4	4928	3.94 (3.92 - 3.97)	1.00 (1.00 - 1.01)	
Q5 (Highest)	4927	3.90 (3.87 - 3.92)	0.99 (0.99 - 1.00)	
Fat from dairy				

Nutrient	N	Geometric mean in mmol/L (95%	Relative geometric mean	p-trend
Dietary cholesterol (mg)				
Q1 (Lowest)	4928	3.93 (3.90 - 3.95)	1.00 (0.99 - 1.01)	0.002
Q2	4928	3.95 (3.93 - 3.98)	1.01 (1.00 - 1.01)	
Q3	4928	3.92 (3.90 - 3.95)	1.00 (0.99 - 1.01)	
Q4	4928	3.92 (3.89 - 3.94)	1.00 (0.99 - 1.00)	
Q5 (Highest)	4927	3.89 (3.86 - 3.91)	0.99 (0.98 - 1.00)	
Trans fatty acids				
Q1 (Lowest)	4928	3.94 (3.92 - 3.97)	1.00 (0.99 - 1.01)	0.372
Q2	4928	3.93 (3.90 - 3.96)	1.00 (0.99 - 1.00)	
Q3	4928	3.94 (3.91 - 3.97)	1.00 (0.99 - 1.01)	
Q4	4928	3.95 (3.92 - 3.98)	1.00 (0.99 - 1.01)	
Q5 (Highest)	4927	3.91 (3.89 - 3.94)	0.99 (0.99 - 1.00)	
SFA				
Q1 (Lowest)	4928	3.90 (3.87 - 3.92)	1.00 (0.99 - 1.01)	0.508
Q2	4928	3.94 (3.91 - 3.96)	1.01 (1.00 - 1.02)	
Q3	4928	3.93 (3.90 - 3.95)	1.01 (1.00 - 1.01)	
Q4	4928	3.93 (3.91 - 3.95)	1.01 (1.00 - 1.01)	
Q5 (Highest)	4927	3.92 (3.89 - 3.94)	1.00 (1.00 - 1.01)	
MUFA				
Q1 (Lowest)	4928	3.94 (3.91 - 3.96)	1.00 (0.99 - 1.01)	0.000
Q2	4928	3.95 (3.93 - 3.97)	1.00 (1.00 - 1.01)	
Q3	4928	3.91 (3.89 - 3.94)	0.99 (0.99 - 1.00)	
Q4	4928	3.92 (3.90 - 3.95)	1.00 (0.99 - 1.00)	
Q5 (Highest)	4927	3.88 (3.86 - 3.91)	0.99 (0.98 - 0.99)	
PUFA				
Q1 (Lowest)	4928	3.98 (3.96 - 4.00)	1.00 (0.99 - 1.01)	<0.00
Q2	4928	3.94 (3.91 - 3.96)	0.99 (0.98 - 1.00)	
Q3	4928	3.92 (3.90 - 3.94)	0.98 (0.98 - 0.99)	
Q4	4928	3.90 (3.88 - 3.93)	0.98 (0.97 - 0.99)	
Q5 (Highest)	4927	3.87 (3.84 - 3.89)	0.97 (0.97 - 0.98)	
n-3 fatty acids				
Q1 (Lowest)	4928	3.98 (3.96 - 4.00)	1.00 (0.99 - 1.01)	<0.00
Q2	4928	3.93 (3.91 - 3.95)	0.99 (0.98 - 0.99)	
Q3	4928	3.93 (3.91 - 3.96)	0.99 (0.98 - 0.99)	
Q4	4928	3.91 (3.89 - 3.94)	0.98 (0.98 - 0.99)	
Q5 (Highest)	4927	3.85 (3.83 - 3.87)	0.97 (0.96 - 0.97)	
n-6 fatty acids				
Q1 (Lowest)	4928	3.97 (3.95 - 4.00)	1.00 (0.99 - 1.01)	<0.00
Q2	4928	3.94 (3.91 - 3.96)	0.99 (0.98 - 1.00)	
Q3	4928	3.92 (3.89 - 3.94)	0.99 (0.98 - 0.99)	
Q4	4928	3.91 (3.89 - 3.93)	0.98 (0.98 - 0.99)	
Q5 (Highest)	4927	3.87 (3.85 - 3.90)	0.97 (0.97 - 0.98)	
Total protein				
Q1 (Lowest)	4928	3.95 (3.92 - 3.97)	1.00 (0.99 - 1.01)	0.006
Q2	4928	3.92 (3.90 - 3.94)	0.99 (0.99 - 1.00)	
Q3	4928	3.93 (3.91 - 3.95)	1.00 (0.99 - 1.00)	
Q4	4928	3.92 (3.90 - 3.94)	0.99 (0.99 - 1.00)	
Q5 (Highest)	4927	3.89 (3.87 - 3.91)	0.99 (0.98 - 0.99)	
Protein from plant				
Q1 (Lowest)	4928	3.92 (3.90 - 3.95)	1.00 (0.99 - 1.01)	0.129
Q2	4928	3.93 (3.91 - 3.96)	1.00 (1.00 - 1.01)	
Q3	4928	3.93 (3.91 - 3.96)	1.00 (1.00 - 1.01)	
Q4	4928	3.92 (3.90 - 3.94)	1.00 (0.99 - 1.00)	
Q5 (Highest)	4927	3.90 (3.88 - 3.92)	0.99 (0.99 - 1.00)	
Protein from animal sources				
Q1 (Lowest)	4928	3.93 (3.91 - 3.96)	1.00 (0.99 - 1.01)	0.104
Q2	4928	3.91 (3.89 - 3.94)	0.99 (0.99 - 1.00)	
Q3	4928	3.94 (3.91 - 3.96)	1.00 (0.99 - 1.01)	
Q4	4928	3.93 (3.91 - 3.96)	1.00 (0.99 - 1.01)	
Q5 (Highest)	4927	3.89 (3.87 - 3.91)	0.99 (0.98 - 0.99)	
Protein from dairy				
Q1 (Lowest)	4928	3.95 (3.92 - 3.97)	1.00 (0.99 - 1.01)	0.009
Q2	4928	3.92 (3.90 - 3.94)	0.99 (0.99 - 1.00)	
Q3	4928	3.93 (3.91 - 3.95)	1.00 (0.99 - 1.00)	
Q4	4928	3.92 (3.89 - 3.94)	0.99 (0.99 - 1.00)	
Q5 (Highest)	4927	3.90 (3.87 - 3.92)	0.99 (0.98 - 0.99)	
Protein from non-dairy animal sources				

Q1 (Lowest)	4928	3.94 (3.92 - 3.97)	1.00 (0.99 - 1.01)	0.0088	Q1 (Lowest)	4928	3.93 (3.91 - 3.95)	1.00 (0.99 - 1.01)	0.494
Q2	4928	3.92 (3.90 - 3.95)	1.00 (0.99 - 1.00)		Q2	4928	3.91 (3.89 - 3.94)	1.00 (0.99 - 1.00)	
Q3	4928	3.93 (3.90 - 3.95)	1.00 (0.99 - 1.00)		Q3	4928	3.93 (3.90 - 3.95)	1.00 (0.99 - 1.01)	
Q4	4928	3.92 (3.90 - 3.95)	0.99 (0.99 - 1.00)		Q4	4928	3.94 (3.91 - 3.96)	1.00 (1.00 - 1.01)	
Q5 (Highest)	4927	3.89 (3.87 - 3.92)	0.99 (0.98 - 0.99)		Q5 (Highest)	4927	3.90 (3.88 - 3.93)	0.99 (0.99 - 1.00)	
Fat from non-dairy animal sources									
Q1 (Lowest)	4928	3.92 (3.90 - 3.94)	1.00 (0.99 - 1.01)	0.9417					
Q2	4928	3.92 (3.90 - 3.95)	1.00 (0.99 - 1.01)						
Q3	4928	3.93 (3.90 - 3.95)	1.00 (1.00 - 1.01)						
Q4	4928	3.91 (3.89 - 3.94)	1.00 (0.99 - 1.00)						
Q5 (Highest)	4927	3.92 (3.90 - 3.95)	1.00 (0.99 - 1.01)						

Models adjusted for age at recruitment (<45, 45-49, 50-54, 55-59, 60-64, >65), sex, ethnicity (white, mixed race, Asian or Asian British, black or black British, other, unknown), region (London, North-West England, North-Eastern England, Yorkshire & the Humber, West Midlands, East Midlands, South-West England, Wales), Townsend deprivation index (quintiles from least to most affluent, unknown), smoking status (never, previous, <15 cigarettes/day, 15-29 cigarettes/day, >30 cigarettes/day, unknown), physical activity (low, medium or high according to MET hours per week, unknown), alcohol (<1, 1-9, 10-19, 20-29, >30 g/day, unknown), body mass index (<20, 20-22.4, 22.5-24.9, 25-27.4, 27.5-29.9, 30-32.4, 32.5-34.9, 35-37.4, 37.5-39.9 or >40 in kg/m^2), height (sex-specific groups in 5cm increments, unknown), diabetes diagnosed by a doctor [illegible]

Abbreviations: CI = confidence interval; g/day = grams per day; HDL-C = high-density lipoprotein cholesterol; MUFA = monounsaturated fatty acids; n=3 = omega-3; n-6 = omega-6; PUFA = polyunsaturated fatty acids; Q = quintile; SFA = saturated fatty acids; TC = total cholesterol.

*For fiber Q1 and Q5 represent quintiles of total fiber intake (g/day).

p-trend was calculated by modelling quintiles of macronutrient intake as continuous variables in the regression model.

Supplemental Table VII. Geometric mean concentrations of TG-to-HDL-C ratio by percentage intake of macronutrients.

Nutrient	N	Geometric mean in mmol/L (95%	Relative geometric mean	p-trend
Total carbohydrates				
Q1 (Lowest)	4928	0.86 (0.85 - 0.88)	1.00 (0.98 - 1.02)	<0.000
Q2	4928	0.92 (0.91 - 0.94)	1.07 (1.05 - 1.09)	
Q3	4928	0.96 (0.94 - 0.97)	1.11 (1.10 - 1.13)	
Q4	4928	1.00 (0.99 - 1.02)	1.16 (1.15 - 1.18)	
Q5 (Highest)	4927	1.03 (1.01 - 1.05)	1.19 (1.17 - 1.21)	
Total sugars				
Q1 (Lowest)	4928	0.91 (0.89 - 0.92)	1.00 (0.98 - 1.02)	<0.000
Q2	4928	0.94 (0.92 - 0.95)	1.03 (1.02 - 1.05)	
Q3	4928	0.96 (0.94 - 0.97)	1.06 (1.04 - 1.08)	
Q4	4928	0.97 (0.95 - 0.98)	1.07 (1.05 - 1.08)	
Q5 (Highest)	4927	1.00 (0.98 - 1.01)	1.10 (1.09 - 1.12)	
Free sugars				
Q1 (Lowest)	4928	0.89 (0.88 - 0.90)	1.00 (0.98 - 1.02)	<0.000
Q2	4928	0.92 (0.91 - 0.94)	1.03 (1.02 - 1.05)	
Q3	4928	0.93 (0.92 - 0.95)	1.05 (1.03 - 1.06)	
Q4	4928	0.99 (0.97 - 1.00)	1.11 (1.09 - 1.12)	
Q5 (Highest)	4927	1.04 (1.02 - 1.05)	1.16 (1.15 - 1.18)	
Non-free sugars				
Q1 (Lowest)	4928	0.97 (0.96 - 0.99)	1.00 (0.98 - 1.02)	0.0002
Q2	4928	0.96 (0.95 - 0.98)	0.99 (0.98 - 1.01)	
Q3	4928	0.95 (0.94 - 0.97)	0.98 (0.97 - 1.00)	
Q4	4928	0.95 (0.94 - 0.97)	0.98 (0.97 - 1.00)	
Q5 (Highest)	4927	0.93 (0.91 - 0.94)	0.95 (0.94 - 0.97)	
Total starch				
Q1 (Lowest)	4928	0.90 (0.89 - 0.91)	1.00 (0.98 - 1.02)	<0.000
Q2	4928	0.94 (0.92 - 0.95)	1.04 (1.03 - 1.06)	
Q3	4928	0.96 (0.94 - 0.97)	1.06 (1.05 - 1.08)	
Q4	4928	0.99 (0.97 - 1.00)	1.10 (1.08 - 1.11)	
Q5 (Highest)	4927	0.98 (0.97 - 1.00)	1.09 (1.07 - 1.11)	
Starch from wholegrains				
Q1 (Lowest)	4928	0.96 (0.95 - 0.98)	1.00 (0.98 - 1.02)	0.8402
Q2	4928	0.95 (0.93 - 0.96)	0.98 (0.97 - 1.00)	
Q3	4928	0.95 (0.93 - 0.96)	0.98 (0.97 - 1.00)	
Q4	4928	0.95 (0.94 - 0.97)	0.99 (0.98 - 1.01)	
Q5 (Highest)	4927	0.95 (0.94 - 0.97)	0.99 (0.98 - 1.01)	
Starch from refined				
Q1 (Lowest)	4928	0.92 (0.91 - 0.94)	1.00 (0.98 - 1.02)	<0.000
Q2	4928	0.94 (0.92 - 0.95)	1.01 (1.00 - 1.03)	
Q3	4928	0.95 (0.94 - 0.97)	1.03 (1.02 - 1.05)	
Q4	4928	0.97 (0.95 - 0.98)	1.05 (1.03 - 1.06)	
Q5 (Highest)	4927	0.98 (0.97 - 1.00)	1.06 (1.05 - 1.08)	
Fiber				
Q1 (Lowest)	4928	0.96 (0.94 - 0.97)	1.00 (0.98 - 1.02)	0.1944
Q2	4928	0.96 (0.95 - 0.98)	1.01 (0.99 - 1.02)	
Q3	4928	0.95 (0.94 - 0.97)	1.00 (0.98 - 1.01)	
Q4	4928	0.95 (0.93 - 0.96)	0.99 (0.97 - 1.00)	
Q5 (Highest)	4927	0.95 (0.93 - 0.96)	0.99 (0.97 - 1.01)	
Total fat				
Q1 (Lowest)	4928	0.99 (0.97 - 1.01)	1.00 (0.98 - 1.02)	<0.000
Q2	4928	0.98 (0.97 - 1.00)	0.99 (0.98 - 1.01)	
Q3	4928	0.97 (0.95 - 0.98)	0.98 (0.96 - 0.99)	
Q4	4928	0.93 (0.92 - 0.95)	0.94 (0.93 - 0.96)	
Q5 (Highest)	4927	0.90 (0.88 - 0.91)	0.90 (0.89 - 0.92)	
Fat from plant sources				
Q1 (Lowest)	4928	0.95 (0.93 - 0.96)	1.00 (0.98 - 1.02)	0.1815
Q2	4928	0.96 (0.95 - 0.98)	1.01 (1.00 - 1.03)	
Q3	4928	0.96 (0.95 - 0.98)	1.01 (1.00 - 1.03)	
Q4	4928	0.95 (0.94 - 0.96)	1.00 (0.99 - 1.02)	
Q5 (Highest)	4927	0.94 (0.92 - 0.95)	0.99 (0.97 - 1.00)	
Fat from animal sources				
Q1 (Lowest)	4928	0.98 (0.97 - 1.00)	1.00 (0.98 - 1.02)	<0.000
Q2	4928	0.97 (0.96 - 0.99)	0.99 (0.97 - 1.00)	
Q3	4928	0.96 (0.94 - 0.97)	0.97 (0.96 - 0.99)	
Q4	4928	0.95 (0.93 - 0.96)	0.96 (0.95 - 0.98)	
Q5 (Highest)	4927	0.90 (0.89 - 0.91)	0.91 (0.90 - 0.93)	
Fat from dairy				

Nutrient	N	Geometric mean in mmol/L (95%	Relative geometric mean	p-trend
Dietary cholesterol (mg)				
Q1 (Lowest)	4928	0.97 (0.96 - 0.99)	1.00 (0.98 - 1.02)	<0.00
Q2	4928	0.97 (0.95 - 0.98)	0.99 (0.98 - 1.01)	
Q3	4928	0.96 (0.94 - 0.97)	0.98 (0.97 - 1.00)	
Q4	4928	0.94 (0.93 - 0.96)	0.97 (0.95 - 0.98)	
Q5 (Highest)	4927	0.92 (0.90 - 0.93)	0.94 (0.93 - 0.96)	
Trans fatty acids				
Q1 (Lowest)	4928	0.97 (0.95 - 0.99)	1.00 (0.98 - 1.02)	0.006
Q2	4928	0.98 (0.96 - 0.99)	1.00 (0.99 - 1.02)	
Q3	4928	0.97 (0.95 - 0.98)	1.00 (0.98 - 1.01)	
Q4	4928	0.97 (0.95 - 0.99)	1.00 (0.98 - 1.02)	
Q5 (Highest)	4927	0.94 (0.92 - 0.95)	0.96 (0.95 - 0.98)	
SFA				
Q1 (Lowest)	4928	0.97 (0.95 - 0.98)	1.00 (0.98 - 1.02)	<0.00
Q2	4928	0.98 (0.96 - 0.99)	1.01 (0.99 - 1.03)	
Q3	4928	0.96 (0.94 - 0.97)	0.99 (0.97 - 1.01)	
Q4	4928	0.96 (0.94 - 0.97)	0.99 (0.98 - 1.01)	
Q5 (Highest)	4927	0.91 (0.89 - 0.92)	0.94 (0.92 - 0.95)	
MUFA				
Q1 (Lowest)	4928	0.99 (0.97 - 1.00)	1.00 (0.98 - 1.02)	<0.00
Q2	4928	0.97 (0.96 - 0.99)	0.99 (0.97 - 1.00)	
Q3	4928	0.95 (0.94 - 0.97)	0.97 (0.95 - 0.98)	
Q4	4928	0.94 (0.93 - 0.95)	0.95 (0.94 - 0.97)	
Q5 (Highest)	4927	0.91 (0.89 - 0.92)	0.92 (0.91 - 0.93)	
PUFA				
Q1 (Lowest)	4928	1.00 (0.99 - 1.02)	1.00 (0.98 - 1.02)	<0.00
Q2	4928	0.96 (0.94 - 0.97)	0.96 (0.94 - 0.97)	
Q3	4928	0.95 (0.93 - 0.96)	0.95 (0.93 - 0.96)	
Q4	4928	0.94 (0.93 - 0.96)	0.94 (0.93 - 0.96)	
Q5 (Highest)	4927	0.91 (0.90 - 0.93)	0.91 (0.90 - 0.93)	
n-3 fatty acids				
Q1 (Lowest)	4928	1.01 (0.99 - 1.03)	1.00 (0.98 - 1.02)	<0.00
Q2	4928	0.97 (0.95 - 0.98)	0.96 (0.95 - 0.97)	
Q3	4928	0.98 (0.96 - 0.99)	0.97 (0.95 - 0.98)	
Q4	4928	0.94 (0.92 - 0.95)	0.93 (0.91 - 0.94)	
Q5 (Highest)	4927	0.88 (0.86 - 0.89)	0.87 (0.85 - 0.88)	
n-6 fatty acids				
Q1 (Lowest)	4928	0.98 (0.97 - 1.00)	1.00 (0.98 - 1.02)	<0.00
Q2	4928	0.96 (0.94 - 0.97)	0.97 (0.96 - 0.99)	
Q3	4928	0.94 (0.93 - 0.96)	0.96 (0.94 - 0.97)	
Q4	4928	0.95 (0.94 - 0.97)	0.97 (0.95 - 0.98)	
Q5 (Highest)	4927	0.92 (0.91 - 0.94)	0.94 (0.92 - 0.95)	
Total protein				
Q1 (Lowest)	4928	0.98 (0.96 - 1.00)	1.00 (0.98 - 1.02)	<0.00
Q2	4928	0.96 (0.95 - 0.98)	0.98 (0.97 - 1.00)	
Q3	4928	0.95 (0.94 - 0.97)	0.97 (0.96 - 0.99)	
Q4	4928	0.95 (0.94 - 0.97)	0.97 (0.96 - 0.99)	
Q5 (Highest)	4927	0.92 (0.90 - 0.93)	0.93 (0.92 - 0.95)	
Protein from plant				
Q1 (Lowest)	4928	0.93 (0.92 - 0.95)	1.00 (0.98 - 1.02)	0.007
Q2	4928	0.95 (0.94 - 0.97)	1.02 (1.01 - 1.04)	
Q3	4928	0.96 (0.95 - 0.98)	1.04 (1.02 - 1.05)	
Q4	4928	0.95 (0.94 - 0.97)	1.02 (1.01 - 1.04)	
Q5 (Highest)	4927	0.96 (0.95 - 0.98)	1.03 (1.02 - 1.05)	
Protein from animal sources				
Q1 (Lowest)	4928	0.98 (0.97 - 1.00)	1.00 (0.98 - 1.02)	<0.00
Q2	4928	0.95 (0.94 - 0.97)	0.97 (0.95 - 0.98)	
Q3	4928	0.96 (0.94 - 0.97)	0.97 (0.96 - 0.99)	
Q4	4928	0.96 (0.94 - 0.97)	0.97 (0.96 - 0.99)	
Q5 (Highest)	4927	0.91 (0.90 - 0.93)	0.93 (0.91 - 0.94)	
Protein from dairy				
Q1 (Lowest)	4928	0.96 (0.95 - 0.98)	1.00 (0.98 - 1.02)	0.000
Q2	4928	0.97 (0.95 - 0.98)	1.00 (0.99 - 1.02)	
Q3	4928	0.96 (0.94 - 0.97)	1.00 (0.98 - 1.01)	
Q4	4928	0.94 (0.92 - 0.95)	0.98 (0.96 - 0.99)	
Q5 (Highest)	4927	0.93 (0.92 - 0.95)	0.97 (0.96 - 0.99)	
Protein from non-dairy animal sources				

Q1 (Lowest)	4928	0.97 (0.96 - 0.99)	1.00 (0.98 - 1.02)	<0.000	Q1 (Lowest)	4928	0.98 (0.97 - 1.00)	1.00 (0.98 - 1.02)	<0.00
Q2	4928	0.97 (0.96 - 0.99)	1.00 (0.98 - 1.01)		Q2	4928	0.95 (0.93 - 0.96)	0.96 (0.95 - 0.98)	
Q3	4928	0.95 (0.94 - 0.97)	0.98 (0.96 - 0.99)		Q3	4928	0.95 (0.94 - 0.97)	0.97 (0.95 - 0.98)	
Q4	4928	0.96 (0.94 - 0.97)	0.98 (0.97 - 1.00)		Q4	4928	0.95 (0.94 - 0.96)	0.97 (0.95 - 0.98)	
Q5 (Highest)	4927	0.91 (0.90 - 0.93)	0.94 (0.92 - 0.95)		Q5 (Highest)	4927	0.93 (0.91 - 0.94)	0.94 (0.93 - 0.96)	
Fat from non-dairy animal sources									
Q1 (Lowest)	4928	0.98 (0.96 - 0.99)	1.00 (0.98 - 1.02)	<0.000					
Q2	4928	0.96 (0.95 - 0.98)	0.98 (0.97 - 1.00)						
Q3	4928	0.96 (0.95 - 0.98)	0.98 (0.97 - 1.00)						
Q4	4928	0.93 (0.92 - 0.95)	0.96 (0.94 - 0.97)						
Q5 (Highest)	4927	0.93 (0.91 - 0.94)	0.95 (0.93 - 0.96)						

Models adjusted for age at recruitment (<45, 45-49, 50-54, 55-59, 60-64, $\geq$65), sex, ethnicity (white, mixed race, Asian or Asian British, black or black British, other, unknown), region (London, North-West England, North-Eastern England, Yorkshire & the Humber, West Midlands, East Midlands, South-West England, Wales), Townsend deprivation index (quintiles from least to most affluent, unknown), smoking status (never, previous, <15 cigarettes/day, 15-29 cigarettes/day, $\geq$30 cigarettes/day, unknown), physical activity (low, medium or high according to MET hours per week, unknown), alcohol (<1, 1-9, 10-19, 20-29, $\geq$30 g/day, unknown), body mass index (<20, 20-22.4, 22.5-24.9, 25-27.4, 27.5-29.9, 30-32.4, 32.5-34.9, 35-37.4, 37.5-39.9 or $\geq$40 in kg/m^2), height (sex-specific groups in 5cm increments, unknown), diabetes diagnosed by a doctor

Abbreviations: CI = confidence interval; g/day = grams per day; HDL-C = high-density lipoprotein cholesterol; MUFA = monounsaturated fatty acids; n=3 = omega-3; n-6 = omega-6; PUFA = polyunsaturated fatty acids; Q = quintile; SFA = saturated fatty acids; TG = triglyceride.

*For fiber Q1 and Q5 represent quintiles of total fiber intake (g/day).

p-trend was calculated by modelling quintiles of macronutrient intake as continuous variables in the regression model.

Supplemental Table VIII. Geometric mean concentrations of ApoB (mmol/L) by percentage intake of macronutrients.

Nutrient	N	Geometric mean in mmol/L (95%	Relative geometric mean	p-trend
Total carbohydrates				
Q1 (Lowest)	4928	1.04 (1.04 - 1.05)	1.00 (0.99 - 1.01)	<0.000
Q2	4928	1.03 (1.02 - 1.04)	0.99 (0.98 - 0.99)	
Q3	4928	1.04 (1.03 - 1.04)	0.99 (0.99 - 1.00)	
Q4	4928	1.03 (1.02 - 1.03)	0.98 (0.98 - 0.99)	
Q5 (Highest)	4927	1.02 (1.01 - 1.03)	0.98 (0.97 - 0.98)	
Total sugars				
Q1 (Lowest)	4928	1.03 (1.03 - 1.04)	1.00 (0.99 - 1.01)	0.2692
Q2	4928	1.03 (1.02 - 1.04)	1.00 (0.99 - 1.00)	
Q3	4928	1.03 (1.02 - 1.04)	1.00 (0.99 - 1.01)	
Q4	4928	1.03 (1.03 - 1.04)	1.00 (0.99 - 1.01)	
Q5 (Highest)	4927	1.04 (1.03 - 1.04)	1.00 (1.00 - 1.01)	
Free sugars				
Q1 (Lowest)	4928	1.02 (1.02 - 1.03)	1.00 (0.99 - 1.01)	<0.000
Q2	4928	1.03 (1.02 - 1.03)	1.00 (1.00 - 1.01)	
Q3	4928	1.03 (1.03 - 1.04)	1.01 (1.00 - 1.01)	
Q4	4928	1.03 (1.03 - 1.04)	1.01 (1.00 - 1.02)	
Q5 (Highest)	4927	1.04 (1.04 - 1.05)	1.02 (1.01 - 1.02)	
Non-free sugars				
Q1 (Lowest)	4928	1.04 (1.04 - 1.05)	1.00 (0.99 - 1.01)	0.0008
Q2	4928	1.03 (1.02 - 1.04)	0.99 (0.98 - 0.99)	
Q3	4928	1.03 (1.02 - 1.04)	0.99 (0.98 - 0.99)	
Q4	4928	1.03 (1.02 - 1.04)	0.99 (0.98 - 0.99)	
Q5 (Highest)	4927	1.03 (1.02 - 1.03)	0.98 (0.98 - 0.99)	
Total starch				
Q1 (Lowest)	4928	1.04 (1.04 - 1.05)	1.00 (0.99 - 1.01)	<0.000
Q2	4928	1.04 (1.03 - 1.04)	0.99 (0.99 - 1.00)	
Q3	4928	1.03 (1.02 - 1.04)	0.99 (0.98 - 0.99)	
Q4	4928	1.03 (1.02 - 1.04)	0.99 (0.98 - 0.99)	
Q5 (Highest)	4927	1.02 (1.01 - 1.02)	0.97 (0.97 - 0.98)	
Starch from wholegrains				
Q1 (Lowest)	4928	1.04 (1.04 - 1.05)	1.00 (0.99 - 1.01)	<0.000
Q2	4928	1.03 (1.03 - 1.04)	0.99 (0.99 - 1.00)	
Q3	4928	1.03 (1.02 - 1.04)	0.99 (0.98 - 0.99)	
Q4	4928	1.03 (1.02 - 1.04)	0.99 (0.98 - 0.99)	
Q5 (Highest)	4927	1.02 (1.02 - 1.03)	0.98 (0.97 - 0.99)	
Starch from refined				
Q1 (Lowest)	4928	1.03 (1.03 - 1.04)	1.00 (0.99 - 1.01)	0.0714
Q2	4928	1.04 (1.03 - 1.04)	1.01 (1.00 - 1.01)	
Q3	4928	1.03 (1.02 - 1.04)	1.00 (0.99 - 1.00)	
Q4	4928	1.03 (1.02 - 1.04)	1.00 (0.99 - 1.00)	
Q5 (Highest)	4927	1.03 (1.02 - 1.03)	0.99 (0.99 - 1.00)	
Fiber				
Q1 (Lowest)	4928	1.05 (1.04 - 1.05)	1.00 (0.99 - 1.01)	<0.000
Q2	4928	1.03 (1.03 - 1.04)	0.99 (0.98 - 0.99)	
Q3	4928	1.03 (1.02 - 1.04)	0.98 (0.98 - 0.99)	
Q4	4928	1.03 (1.02 - 1.03)	0.98 (0.98 - 0.99)	
Q5 (Highest)	4927	1.02 (1.01 - 1.03)	0.98 (0.97 - 0.98)	
Total fat				
Q1 (Lowest)	4928	1.02 (1.01 - 1.03)	1.00 (0.99 - 1.01)	<0.000
Q2	4928	1.03 (1.02 - 1.03)	1.01 (1.00 - 1.01)	
Q3	4928	1.03 (1.03 - 1.04)	1.01 (1.01 - 1.02)	
Q4	4928	1.04 (1.03 - 1.04)	1.02 (1.01 - 1.02)	
Q5 (Highest)	4927	1.04 (1.03 - 1.05)	1.02 (1.01 - 1.02)	
Fat from plant sources				
Q1 (Lowest)	4928	1.03 (1.03 - 1.04)	1.00 (0.99 - 1.01)	0.1019
Q2	4928	1.03 (1.03 - 1.04)	1.00 (1.00 - 1.01)	
Q3	4928	1.03 (1.03 - 1.04)	1.00 (0.99 - 1.01)	
Q4	4928	1.03 (1.02 - 1.04)	1.00 (0.99 - 1.00)	
Q5 (Highest)	4927	1.03 (1.02 - 1.03)	0.99 (0.99 - 1.00)	
Fat from animal sources				
Q1 (Lowest)	4928	1.02 (1.01 - 1.03)	1.00 (0.99 - 1.01)	<0.000
Q2	4928	1.03 (1.02 - 1.03)	1.00 (1.00 - 1.01)	
Q3	4928	1.03 (1.02 - 1.04)	1.01 (1.00 - 1.01)	
Q4	4928	1.04 (1.03 - 1.05)	1.02 (1.01 - 1.02)	
Q5 (Highest)	4927	1.04 (1.04 - 1.05)	1.02 (1.02 - 1.03)	
Fat from dairy				
Dietary cholesterol (mg)				
Q1 (Lowest)	4928	1.02 (1.01 - 1.03)	1.00 (0.99 - 1.01)	0.006
Q2	4928	1.04 (1.03 - 1.05)	1.02 (1.01 - 1.03)	
Q3	4928	1.03 (1.03 - 1.04)	1.01 (1.01 - 1.02)	
Q4	4928	1.03 (1.03 - 1.04)	1.01 (1.01 - 1.02)	
Q5 (Highest)	4927	1.04 (1.03 - 1.04)	1.02 (1.01 - 1.02)	
Trans fatty acids				
Q1 (Lowest)	4928	1.03 (1.02 - 1.04)	1.00 (0.99 - 1.01)	0.017
Q2	4928	1.02 (1.02 - 1.03)	0.99 (0.99 - 1.00)	
Q3	4928	1.03 (1.02 - 1.04)	1.00 (0.99 - 1.01)	
Q4	4928	1.04 (1.03 - 1.04)	1.01 (1.00 - 1.01)	
Q5 (Highest)	4927	1.04 (1.03 - 1.04)	1.01 (1.00 - 1.01)	
SFA				
Q1 (Lowest)	4928	1.02 (1.01 - 1.02)	1.00 (0.99 - 1.01)	<0.00
Q2	4928	1.03 (1.02 - 1.03)	1.01 (1.00 - 1.02)	
Q3	4928	1.03 (1.03 - 1.04)	1.01 (1.01 - 1.02)	
Q4	4928	1.03 (1.03 - 1.04)	1.02 (1.01 - 1.02)	
Q5 (Highest)	4927	1.04 (1.04 - 1.05)	1.03 (1.02 - 1.03)	
MUFA				
Q1 (Lowest)	4928	1.02 (1.01 - 1.03)	1.00 (0.99 - 1.01)	0.000
Q2	4928	1.04 (1.03 - 1.04)	1.02 (1.01 - 1.02)	
Q3	4928	1.03 (1.02 - 1.03)	1.01 (1.00 - 1.02)	
Q4	4928	1.04 (1.03 - 1.04)	1.02 (1.01 - 1.02)	
Q5 (Highest)	4927	1.04 (1.03 - 1.04)	1.02 (1.01 - 1.02)	
PUFA				
Q1 (Lowest)	4928	1.03 (1.03 - 1.04)	1.00 (0.99 - 1.01)	0.322
Q2	4928	1.03 (1.02 - 1.04)	1.00 (0.99 - 1.00)	
Q3	4928	1.04 (1.03 - 1.04)	1.00 (1.00 - 1.01)	
Q4	4928	1.03 (1.03 - 1.04)	1.00 (1.00 - 1.01)	
Q5 (Highest)	4927	1.03 (1.02 - 1.03)	0.99 (0.99 - 1.00)	
n-3 fatty acids				
Q1 (Lowest)	4928	1.03 (1.02 - 1.04)	1.00 (0.99 - 1.01)	0.016
Q2	4928	1.03 (1.02 - 1.04)	1.00 (1.00 - 1.01)	
Q3	4928	1.03 (1.02 - 1.03)	1.00 (0.99 - 1.00)	
Q4	4928	1.03 (1.02 - 1.04)	1.00 (1.00 - 1.01)	
Q5 (Highest)	4927	1.04 (1.03 - 1.05)	1.01 (1.01 - 1.02)	
n-6 fatty acids				
Q1 (Lowest)	4928	1.04 (1.03 - 1.04)	1.00 (0.99 - 1.01)	0.021
Q2	4928	1.03 (1.03 - 1.04)	1.00 (0.99 - 1.00)	
Q3	4928	1.03 (1.03 - 1.04)	1.00 (0.99 - 1.01)	
Q4	4928	1.03 (1.03 - 1.04)	1.00 (0.99 - 1.00)	
Q5 (Highest)	4927	1.02 (1.02 - 1.03)	0.99 (0.98 - 0.99)	
Total protein				
Q1 (Lowest)	4928	1.03 (1.02 - 1.03)	1.00 (0.99 - 1.01)	0.124
Q2	4928	1.03 (1.02 - 1.03)	1.00 (0.99 - 1.01)	
Q3	4928	1.03 (1.03 - 1.04)	1.00 (1.00 - 1.01)	
Q4	4928	1.03 (1.03 - 1.04)	1.01 (1.00 - 1.01)	
Q5 (Highest)	4927	1.03 (1.03 - 1.04)	1.00 (1.00 - 1.01)	
Protein from plant				
Q1 (Lowest)	4928	1.04 (1.04 - 1.05)	1.00 (0.99 - 1.01)	<0.00
Q2	4928	1.04 (1.03 - 1.04)	1.00 (0.99 - 1.00)	
Q3	4928	1.03 (1.03 - 1.04)	0.99 (0.98 - 1.00)	
Q4	4928	1.03 (1.03 - 1.04)	0.99 (0.98 - 1.00)	
Q5 (Highest)	4927	1.02 (1.01 - 1.02)	0.98 (0.97 - 0.98)	
Protein from animal sources				
Q1 (Lowest)	4928	1.02 (1.02 - 1.03)	1.00 (0.99 - 1.01)	0.000
Q2	4928	1.03 (1.02 - 1.03)	1.00 (1.00 - 1.01)	
Q3	4928	1.04 (1.03 - 1.04)	1.01 (1.01 - 1.02)	
Q4	4928	1.04 (1.03 - 1.04)	1.01 (1.01 - 1.02)	
Q5 (Highest)	4927	1.04 (1.03 - 1.04)	1.01 (1.01 - 1.02)	
Protein from dairy				
Q1 (Lowest)	4928	1.04 (1.04 - 1.05)	1.00 (0.99 - 1.01)	0.000
Q2	4928	1.03 (1.02 - 1.04)	0.99 (0.98 - 0.99)	
Q3	4928	1.03 (1.02 - 1.04)	0.99 (0.98 - 0.99)	
Q4	4928	1.03 (1.03 - 1.04)	0.99 (0.98 - 1.00)	
Q5 (Highest)	4927	1.02 (1.02 - 1.03)	0.98 (0.98 - 0.99)	
Protein from non-dairy animal sources				

Q1 (Lowest)	4928	1.03 (1.03 - 1.04)	1.00 (0.99 - 1.01)	0.4918
Q2	4928	1.03 (1.02 - 1.03)	0.99 (0.99 - 1.00)	
Q3	4928	1.03 (1.03 - 1.04)	1.00 (0.99 - 1.00)	
Q4	4928	1.03 (1.03 - 1.04)	1.00 (0.99 - 1.01)	
Q5 (Highest)	4927	1.03 (1.03 - 1.04)	1.00 (0.99 - 1.00)	
Fat from non-dairy animal sources				
Q1 (Lowest)	4928	1.02 (1.01 - 1.02)	1.00 (0.99 - 1.01)	<0.000
Q2	4928	1.03 (1.02 - 1.04)	1.01 (1.01 - 1.02)	
Q3	4928	1.03 (1.02 - 1.03)	1.01 (1.00 - 1.02)	
Q4	4928	1.04 (1.03 - 1.04)	1.02 (1.01 - 1.02)	
Q5 (Highest)	4927	1.04 (1.04 - 1.05)	1.03 (1.02 - 1.03)	

Q1 (Lowest)	4928	1.02 (1.01 - 1.02)	1.00 (0.99 - 1.01)	<0.00
Q2	4928	1.03 (1.02 - 1.03)	1.01 (1.00 - 1.02)	
Q3	4928	1.03 (1.03 - 1.04)	1.02 (1.01 - 1.02)	
Q4	4928	1.04 (1.03 - 1.05)	1.02 (1.02 - 1.03)	
Q5 (Highest)	4927	1.04 (1.03 - 1.04)	1.02 (1.01 - 1.02)	

Models adjusted for age at recruitment (<45, 45-49, 50-54, 55-59, 60-64, ≥65), sex, ethnicity (white, mixed race, Asian or Asian British, black or black British, other, unknown), region (London, North-West England, North-Eastern England, Yorkshire & the Humber, West Midlands, East Midlands, South-West England, Wales), Townsend deprivation index (quintiles from least to most affluent, unknown), smoking status (never, previous, <15 cigarettes/day, 15-29 cigarettes/day, ≥30 cigarettes/day, unknown), physical activity (low, medium or high according to MET hours per week, unknown), alcohol (<1, 1-9, 10-19, 20-29, ≥30 g/day, unknown), body mass index (<20, 20-22.4, 22.5-24.9, 25-27.4, 27.5-29.9, 30-32.4, 32.5-34.9, 35-37.4, 37.5-39.9 or ≥40 in kg/m^2), height (sex-specific groups in 5cm increments, unknown), diabetes diagnosed by a doctor

Abbreviations: Apo = apolipoprotein; CI = confidence interval; g/day = grams per day; MUFA = monounsaturated fatty acids; n=3 = omega-3; n-6 = omega-6; PUFA = polyunsaturated fatty acids; Q = quintile; SFA = saturated fatty acids.

*For fiber Q1 and Q5 represent quintiles of total fiber intake (g/day).

p-trend was calculated by modelling quintiles of macronutrient intake as continuous variables in the regression model.

Supplemental Table IX. Geometric mean concentrations of ApoA1 (mmol/L) by percentage intake of macronutrients.

Nutrient	N	Geometric mean in mmol/L (95%	Relative geometric mean	p-trend
Total carbohydrates				
Q1 (Lowest)	4928	1.61 (1.61 - 1.62)	1.00 (1.00 - 1.00)	<0.000
Q2	4928	1.57 (1.57 - 1.58)	0.98 (0.97 - 0.98)	
Q3	4928	1.56 (1.55 - 1.56)	0.97 (0.96 - 0.97)	
Q4	4928	1.54 (1.53 - 1.54)	0.95 (0.95 - 0.96)	
Q5 (Highest)	4927	1.52 (1.51 - 1.53)	0.94 (0.94 - 0.95)	
Total sugars				
Q1 (Lowest)	4928	1.59 (1.58 - 1.59)	1.00 (1.00 - 1.00)	<0.000
Q2	4928	1.57 (1.57 - 1.58)	0.99 (0.99 - 0.99)	
Q3	4928	1.56 (1.55 - 1.57)	0.98 (0.98 - 0.99)	
Q4	4928	1.55 (1.54 - 1.56)	0.98 (0.97 - 0.98)	
Q5 (Highest)	4927	1.53 (1.53 - 1.54)	0.97 (0.96 - 0.97)	
Free sugars				
Q1 (Lowest)	4928	1.58 (1.57 - 1.58)	1.00 (1.00 - 1.00)	<0.000
Q2	4928	1.57 (1.56 - 1.57)	0.99 (0.99 - 1.00)	
Q3	4928	1.57 (1.56 - 1.57)	0.99 (0.99 - 1.00)	
Q4	4928	1.55 (1.55 - 1.56)	0.99 (0.98 - 0.99)	
Q5 (Highest)	4927	1.54 (1.53 - 1.55)	0.98 (0.97 - 0.98)	
Non-free sugars				
Q1 (Lowest)	4928	1.58 (1.57 - 1.58)	1.00 (1.00 - 1.00)	<0.000
Q2	4928	1.56 (1.56 - 1.57)	0.99 (0.99 - 1.00)	
Q3	4928	1.56 (1.55 - 1.56)	0.99 (0.98 - 0.99)	
Q4	4928	1.56 (1.55 - 1.57)	0.99 (0.98 - 0.99)	
Q5 (Highest)	4927	1.54 (1.54 - 1.55)	0.98 (0.97 - 0.98)	
Total starch				
Q1 (Lowest)	4928	1.58 (1.58 - 1.59)	1.00 (1.00 - 1.00)	<0.000
Q2	4928	1.57 (1.56 - 1.57)	0.99 (0.99 - 0.99)	
Q3	4928	1.56 (1.55 - 1.56)	0.98 (0.98 - 0.99)	
Q4	4928	1.55 (1.54 - 1.56)	0.98 (0.98 - 0.98)	
Q5 (Highest)	4927	1.54 (1.54 - 1.55)	0.98 (0.97 - 0.98)	
Starch from wholegrains				
Q1 (Lowest)	4928	1.57 (1.56 - 1.57)	1.00 (1.00 - 1.00)	<0.000
Q2	4928	1.57 (1.56 - 1.57)	1.00 (0.99 - 1.00)	
Q3	4928	1.56 (1.55 - 1.57)	0.99 (0.99 - 1.00)	
Q4	4928	1.56 (1.55 - 1.56)	0.99 (0.99 - 1.00)	
Q5 (Highest)	4927	1.55 (1.55 - 1.56)	0.99 (0.99 - 0.99)	
Starch from refined				
Q1 (Lowest)	4928	1.57 (1.56 - 1.58)	1.00 (1.00 - 1.00)	<0.000
Q2	4928	1.56 (1.56 - 1.57)	1.00 (0.99 - 1.00)	
Q3	4928	1.56 (1.55 - 1.57)	0.99 (0.99 - 1.00)	
Q4	4928	1.55 (1.54 - 1.56)	0.99 (0.98 - 0.99)	
Q5 (Highest)	4927	1.56 (1.55 - 1.56)	0.99 (0.99 - 0.99)	
Fiber				
Q1 (Lowest)	4928	1.58 (1.57 - 1.58)	1.00 (1.00 - 1.00)	<0.000
Q2	4928	1.57 (1.56 - 1.57)	0.99 (0.99 - 1.00)	
Q3	4928	1.56 (1.55 - 1.57)	0.99 (0.99 - 0.99)	
Q4	4928	1.56 (1.55 - 1.56)	0.99 (0.98 - 0.99)	
Q5 (Highest)	4927	1.54 (1.54 - 1.55)	0.98 (0.97 - 0.98)	
Total fat				
Q1 (Lowest)	4928	1.53 (1.52 - 1.54)	1.00 (1.00 - 1.00)	<0.000
Q2	4928	1.55 (1.54 - 1.55)	1.01 (1.01 - 1.02)	
Q3	4928	1.56 (1.55 - 1.56)	1.02 (1.02 - 1.02)	
Q4	4928	1.57 (1.56 - 1.58)	1.03 (1.02 - 1.03)	
Q5 (Highest)	4927	1.60 (1.59 - 1.60)	1.04 (1.04 - 1.05)	
Fat from plant sources				
Q1 (Lowest)	4928	1.56 (1.55 - 1.56)	1.00 (1.00 - 1.00)	0.0117
Q2	4928	1.55 (1.55 - 1.56)	1.00 (0.99 - 1.00)	
Q3	4928	1.56 (1.55 - 1.56)	1.00 (1.00 - 1.01)	
Q4	4928	1.57 (1.56 - 1.57)	1.01 (1.00 - 1.01)	
Q5 (Highest)	4927	1.56 (1.56 - 1.57)	1.00 (1.00 - 1.01)	
Fat from animal sources				
Q1 (Lowest)	4928	1.54 (1.53 - 1.54)	1.00 (1.00 - 1.00)	<0.000
Q2	4928	1.55 (1.54 - 1.55)	1.01 (1.00 - 1.01)	
Q3	4928	1.56 (1.56 - 1.57)	1.02 (1.01 - 1.02)	
Q4	4928	1.56 (1.56 - 1.57)	1.02 (1.01 - 1.02)	
Q5 (Highest)	4927	1.59 (1.58 - 1.60)	1.04 (1.03 - 1.04)	
Fat from dairy				

Nutrient	N	Geometric mean in mmol/L (95%	Relative geometric mean	p-trend
Dietary cholesterol (mg)				
Q1 (Lowest)	4928	1.54 (1.54 - 1.55)	1.00 (1.00 - 1.00)	<0.00
Q2	4928	1.56 (1.55 - 1.56)	1.01 (1.00 - 1.01)	
Q3	4928	1.56 (1.55 - 1.57)	1.01 (1.01 - 1.01)	
Q4	4928	1.57 (1.56 - 1.57)	1.01 (1.01 - 1.02)	
Q5 (Highest)	4927	1.58 (1.57 - 1.58)	1.02 (1.02 - 1.02)	
Trans fatty acids				
Q1 (Lowest)	4928	1.54 (1.53 - 1.55)	1.00 (1.00 - 1.00)	<0.00
Q2	4928	1.55 (1.54 - 1.56)	1.01 (1.00 - 1.01)	
Q3	4928	1.55 (1.55 - 1.56)	1.01 (1.01 - 1.01)	
Q4	4928	1.56 (1.56 - 1.57)	1.02 (1.01 - 1.02)	
Q5 (Highest)	4927	1.58 (1.57 - 1.59)	1.03 (1.02 - 1.03)	
SFA				
Q1 (Lowest)	4928	1.54 (1.53 - 1.55)	1.00 (1.00 - 1.00)	<0.00
Q2	4928	1.55 (1.54 - 1.55)	1.01 (1.00 - 1.01)	
Q3	4928	1.56 (1.55 - 1.56)	1.01 (1.01 - 1.02)	
Q4	4928	1.57 (1.56 - 1.57)	1.02 (1.01 - 1.02)	
Q5 (Highest)	4927	1.59 (1.58 - 1.60)	1.03 (1.03 - 1.04)	
MUFA				
Q1 (Lowest)	4928	1.53 (1.52 - 1.54)	1.00 (1.00 - 1.00)	<0.00
Q2	4928	1.55 (1.55 - 1.56)	1.02 (1.01 - 1.02)	
Q3	4928	1.56 (1.56 - 1.57)	1.02 (1.02 - 1.02)	
Q4	4928	1.57 (1.56 - 1.57)	1.03 (1.02 - 1.03)	
Q5 (Highest)	4927	1.59 (1.58 - 1.59)	1.04 (1.03 - 1.04)	
PUFA				
Q1 (Lowest)	4928	1.55 (1.54 - 1.55)	1.00 (1.00 - 1.00)	<0.00
Q2	4928	1.55 (1.55 - 1.56)	1.00 (1.00 - 1.01)	
Q3	4928	1.57 (1.56 - 1.57)	1.01 (1.01 - 1.02)	
Q4	4928	1.57 (1.56 - 1.57)	1.01 (1.01 - 1.02)	
Q5 (Highest)	4927	1.57 (1.56 - 1.58)	1.02 (1.01 - 1.02)	
n-3 fatty acids				
Q1 (Lowest)	4928	1.55 (1.54 - 1.55)	1.00 (1.00 - 1.00)	<0.00
Q2	4928	1.56 (1.56 - 1.57)	1.01 (1.01 - 1.01)	
Q3	4928	1.56 (1.55 - 1.56)	1.01 (1.00 - 1.01)	
Q4	4928	1.56 (1.55 - 1.57)	1.01 (1.00 - 1.01)	
Q5 (Highest)	4927	1.58 (1.57 - 1.58)	1.02 (1.01 - 1.02)	
n-6 fatty acids				
Q1 (Lowest)	4928	1.55 (1.54 - 1.55)	1.00 (1.00 - 1.00)	<0.00
Q2	4928	1.55 (1.55 - 1.56)	1.01 (1.00 - 1.01)	
Q3	4928	1.56 (1.56 - 1.57)	1.01 (1.01 - 1.02)	
Q4	4928	1.57 (1.56 - 1.57)	1.01 (1.01 - 1.02)	
Q5 (Highest)	4927	1.57 (1.56 - 1.58)	1.01 (1.01 - 1.02)	
Total protein				
Q1 (Lowest)	4928	1.55 (1.55 - 1.56)	1.00 (1.00 - 1.00)	0.026
Q2	4928	1.56 (1.56 - 1.57)	1.01 (1.00 - 1.01)	
Q3	4928	1.56 (1.55 - 1.57)	1.01 (1.00 - 1.01)	
Q4	4928	1.56 (1.56 - 1.57)	1.01 (1.00 - 1.01)	
Q5 (Highest)	4927	1.56 (1.56 - 1.57)	1.01 (1.00 - 1.01)	
Protein from plant				
Q1 (Lowest)	4928	1.58 (1.57 - 1.58)	1.00 (1.00 - 1.00)	<0.00
Q2	4928	1.57 (1.56 - 1.57)	1.00 (0.99 - 1.00)	
Q3	4928	1.56 (1.55 - 1.56)	0.99 (0.98 - 0.99)	
Q4	4928	1.56 (1.55 - 1.56)	0.99 (0.98 - 0.99)	
Q5 (Highest)	4927	1.54 (1.54 - 1.55)	0.98 (0.98 - 0.98)	
Protein from animal sources				
Q1 (Lowest)	4928	1.55 (1.54 - 1.55)	1.00 (1.00 - 1.00)	<0.00
Q2	4928	1.56 (1.55 - 1.56)	1.01 (1.00 - 1.01)	
Q3	4928	1.56 (1.56 - 1.57)	1.01 (1.01 - 1.02)	
Q4	4928	1.56 (1.56 - 1.57)	1.01 (1.01 - 1.01)	
Q5 (Highest)	4927	1.57 (1.56 - 1.58)	1.02 (1.01 - 1.02)	
Protein from dairy				
Q1 (Lowest)	4928	1.56 (1.55 - 1.56)	1.00 (1.00 - 1.00)	0.965
Q2	4928	1.56 (1.56 - 1.57)	1.00 (1.00 - 1.01)	
Q3	4928	1.56 (1.55 - 1.57)	1.00 (1.00 - 1.00)	
Q4	4928	1.56 (1.55 - 1.57)	1.00 (1.00 - 1.00)	
Q5 (Highest)	4927	1.56 (1.55 - 1.57)	1.00 (1.00 - 1.00)	
Protein from non-dairy animal sources				

Q1 (Lowest)	4928	1.54 (1.54 - 1.55)	1.00 (1.00 - 1.00)	<0.000	Q1 (Lowest)	4928	1.54 (1.54 - 1.55)	1.00 (1.00 - 1.00)	<0.00
Q2	4928	1.55 (1.55 - 1.56)	1.01 (1.00 - 1.01)		Q2	4928	1.56 (1.56 - 1.57)	1.01 (1.01 - 1.01)	
Q3	4928	1.56 (1.55 - 1.56)	1.01 (1.00 - 1.01)		Q3	4928	1.56 (1.56 - 1.57)	1.01 (1.01 - 1.02)	
Q4	4928	1.57 (1.56 - 1.57)	1.01 (1.01 - 1.02)		Q4	4928	1.56 (1.56 - 1.57)	1.01 (1.01 - 1.02)	
Q5 (Highest)	4927	1.58 (1.58 - 1.59)	1.02 (1.02 - 1.03)		Q5 (Highest)	4927	1.57 (1.56 - 1.57)	1.01 (1.01 - 1.02)	
Fat from non-dairy animal sources									
Q1 (Lowest)	4928	1.54 (1.54 - 1.55)	1.00 (1.00 - 1.00)	<0.000					
Q2	4928	1.56 (1.55 - 1.56)	1.01 (1.00 - 1.01)						
Q3	4928	1.56 (1.55 - 1.56)	1.01 (1.00 - 1.01)						
Q4	4928	1.57 (1.56 - 1.57)	1.01 (1.01 - 1.02)						
Q5 (Highest)	4927	1.58 (1.57 - 1.58)	1.02 (1.02 - 1.03)						

Models adjusted for age at recruitment (<45, 45-49, 50-54, 55-59, 60-64, ≥65), sex, ethnicity (white, mixed race, Asian or Asian British, black or black British, other, unknown), region (London, North-West England, North-Eastern England, Yorkshire & the Humber, West Midlands, East Midlands, South-West England, Wales), Townsend deprivation index (quintiles from least to most affluent, unknown), smoking status (never, previous, <15 cigarettes/day, 15-29 cigarettes/day, ≥30 cigarettes/day, unknown), physical activity (low, medium or high according to MET hours per week, unknown), alcohol (<1, 1-9, 10-19, 20-29, ≥30 g/day, unknown), body mass index (<20, 20-22.4, 22.5-24.9, 25-27.4, 27.5-29.9, 30-32.4, 32.5-34.9, 35-37.4, 37.5-39.9 or ≥40 in kg/m^2), height (sex-specific groups in 5cm increments, unknown), diabetes diagnosed by a doctor

Abbreviations: Apo = apolipoprotein; CI = confidence interval; g/day = grams per day; MUFA = monounsaturated fatty acids; n=3 = omega-3; n-6 = omega-6; PUFA = polyunsaturated fatty acids; Q = quintile; SFA = saturated fatty acids.

*For fiber Q1 and Q5 represent quintiles of total fiber intake (g/day).

p-trend was calculated by modelling quintiles of macronutrient intake as continuous variables in the regression model.

Supplemental Table X. Geometric mean concentrations of ApoB-to-ApoA1 ratio by percentage intake of macronutrients.

Nutrient	N	Geometric mean in mmol/L (95%	Relative geometric mean	p-trend
Total carbohydrates				
Q1 (Lowest)	4928	0.65 (0.64 - 0.65)	1.00 (0.99 - 1.01)	<0.000
Q2	4928	0.65 (0.65 - 0.66)	1.01 (1.00 - 1.02)	
Q3	4928	0.67 (0.66 - 0.67)	1.03 (1.02 - 1.04)	
Q4	4928	0.67 (0.66 - 0.67)	1.03 (1.02 - 1.04)	
Q5 (Highest)	4927	0.67 (0.67 - 0.68)	1.04 (1.03 - 1.04)	
Total sugars				
Q1 (Lowest)	4928	0.65 (0.65 - 0.65)	1.00 (0.99 - 1.01)	<0.000
Q2	4928	0.66 (0.65 - 0.66)	1.01 (1.00 - 1.01)	
Q3	4928	0.66 (0.66 - 0.66)	1.02 (1.01 - 1.02)	
Q4	4928	0.67 (0.66 - 0.67)	1.02 (1.02 - 1.03)	
Q5 (Highest)	4927	0.68 (0.67 - 0.68)	1.04 (1.03 - 1.05)	
Free sugars				
Q1 (Lowest)	4928	0.65 (0.64 - 0.65)	1.00 (0.99 - 1.01)	<0.000
Q2	4928	0.66 (0.65 - 0.66)	1.01 (1.00 - 1.02)	
Q3	4928	0.66 (0.65 - 0.66)	1.01 (1.01 - 1.02)	
Q4	4928	0.67 (0.66 - 0.67)	1.02 (1.02 - 1.03)	
Q5 (Highest)	4927	0.68 (0.67 - 0.68)	1.04 (1.03 - 1.05)	
Non-free sugars				
Q1 (Lowest)	4928	0.66 (0.66 - 0.67)	1.00 (0.99 - 1.01)	0.3136
Q2	4928	0.66 (0.65 - 0.66)	1.00 (0.99 - 1.00)	
Q3	4928	0.66 (0.66 - 0.67)	1.00 (0.99 - 1.01)	
Q4	4928	0.66 (0.66 - 0.67)	1.00 (0.99 - 1.01)	
Q5 (Highest)	4927	0.66 (0.66 - 0.67)	1.01 (1.00 - 1.01)	
Total starch				
Q1 (Lowest)	4928	0.66 (0.66 - 0.67)	1.00 (0.99 - 1.01)	0.9921
Q2	4928	0.66 (0.66 - 0.67)	1.00 (0.99 - 1.01)	
Q3	4928	0.66 (0.66 - 0.67)	1.00 (1.00 - 1.01)	
Q4	4928	0.66 (0.66 - 0.67)	1.01 (1.00 - 1.01)	
Q5 (Highest)	4927	0.66 (0.65 - 0.66)	1.00 (0.99 - 1.00)	
Starch from wholegrains				
Q1 (Lowest)	4928	0.67 (0.66 - 0.67)	1.00 (0.99 - 1.01)	0.1157
Q2	4928	0.66 (0.66 - 0.67)	0.99 (0.99 - 1.00)	
Q3	4928	0.66 (0.66 - 0.66)	0.99 (0.99 - 1.00)	
Q4	4928	0.66 (0.66 - 0.67)	0.99 (0.99 - 1.00)	
Q5 (Highest)	4927	0.66 (0.65 - 0.66)	0.99 (0.98 - 1.00)	
Starch from refined				
Q1 (Lowest)	4928	0.66 (0.65 - 0.66)	1.00 (0.99 - 1.01)	0.3715
Q2	4928	0.66 (0.66 - 0.67)	1.01 (1.00 - 1.02)	
Q3	4928	0.66 (0.66 - 0.67)	1.01 (1.00 - 1.01)	
Q4	4928	0.66 (0.66 - 0.67)	1.01 (1.00 - 1.02)	
Q5 (Highest)	4927	0.66 (0.66 - 0.67)	1.00 (1.00 - 1.01)	
Fiber				
Q1 (Lowest)	4928	0.66 (0.66 - 0.67)	1.00 (0.99 - 1.01)	0.7867
Q2	4928	0.66 (0.66 - 0.66)	0.99 (0.99 - 1.00)	
Q3	4928	0.66 (0.66 - 0.66)	0.99 (0.99 - 1.00)	
Q4	4928	0.66 (0.66 - 0.67)	1.00 (0.99 - 1.00)	
Q5 (Highest)	4927	0.66 (0.66 - 0.67)	1.00 (0.99 - 1.01)	
Total fat				
Q1 (Lowest)	4928	0.67 (0.66 - 0.67)	1.00 (0.99 - 1.01)	<0.000
Q2	4928	0.66 (0.66 - 0.67)	1.00 (0.99 - 1.00)	
Q3	4928	0.66 (0.66 - 0.67)	0.99 (0.99 - 1.00)	
Q4	4928	0.66 (0.66 - 0.67)	0.99 (0.98 - 1.00)	
Q5 (Highest)	4927	0.65 (0.65 - 0.66)	0.98 (0.97 - 0.98)	
Fat from plant sources				
Q1 (Lowest)	4928	0.66 (0.66 - 0.67)	1.00 (0.99 - 1.01)	0.0063
Q2	4928	0.67 (0.66 - 0.67)	1.00 (0.99 - 1.01)	
Q3	4928	0.66 (0.66 - 0.67)	1.00 (0.99 - 1.01)	
Q4	4928	0.66 (0.65 - 0.66)	0.99 (0.98 - 1.00)	
Q5 (Highest)	4927	0.66 (0.65 - 0.66)	0.99 (0.98 - 1.00)	
Fat from animal sources				
Q1 (Lowest)	4928	0.66 (0.66 - 0.67)	1.00 (0.99 - 1.01)	0.1113
Q2	4928	0.66 (0.66 - 0.67)	1.00 (0.99 - 1.01)	
Q3	4928	0.66 (0.65 - 0.66)	0.99 (0.98 - 1.00)	
Q4	4928	0.66 (0.66 - 0.67)	1.00 (0.99 - 1.01)	
Q5 (Highest)	4927	0.66 (0.65 - 0.66)	0.99 (0.98 - 1.00)	
Fat from dairy				

Nutrient	N	Geometric mean in mmol/L (95%	Relative geometric mean	p-trend
Dietary cholesterol (mg)				
Q1 (Lowest)	4928	0.66 (0.66 - 0.67)	1.00 (0.99 - 1.01)	0.070
Q2	4928	0.67 (0.66 - 0.67)	1.01 (1.00 - 1.02)	
Q3	4928	0.66 (0.66 - 0.67)	1.00 (0.99 - 1.01)	
Q4	4928	0.66 (0.65 - 0.66)	1.00 (0.99 - 1.00)	
Q5 (Highest)	4927	0.66 (0.65 - 0.66)	1.00 (0.99 - 1.00)	
Trans fatty acids				
Q1 (Lowest)	4928	0.67 (0.66 - 0.68)	1.00 (0.99 - 1.01)	0.003
Q2	4928	0.66 (0.66 - 0.67)	0.99 (0.98 - 0.99)	
Q3	4928	0.66 (0.66 - 0.67)	0.99 (0.98 - 1.00)	
Q4	4928	0.66 (0.66 - 0.67)	0.99 (0.98 - 1.00)	
Q5 (Highest)	4927	0.66 (0.65 - 0.66)	0.98 (0.97 - 0.99)	
SFA				
Q1 (Lowest)	4928	0.66 (0.66 - 0.67)	1.00 (0.99 - 1.01)	0.074
Q2	4928	0.66 (0.66 - 0.67)	1.00 (1.00 - 1.01)	
Q3	4928	0.66 (0.66 - 0.67)	1.00 (0.99 - 1.01)	
Q4	4928	0.66 (0.66 - 0.66)	1.00 (0.99 - 1.00)	
Q5 (Highest)	4927	0.66 (0.65 - 0.66)	0.99 (0.99 - 1.00)	
MUFA				
Q1 (Lowest)	4928	0.67 (0.66 - 0.67)	1.00 (0.99 - 1.01)	0.000
Q2	4928	0.67 (0.66 - 0.67)	1.00 (0.99 - 1.01)	
Q3	4928	0.66 (0.65 - 0.66)	0.99 (0.98 - 1.00)	
Q4	4928	0.66 (0.66 - 0.67)	0.99 (0.99 - 1.00)	
Q5 (Highest)	4927	0.65 (0.65 - 0.66)	0.98 (0.97 - 0.99)	
PUFA				
Q1 (Lowest)	4928	0.67 (0.66 - 0.67)	1.00 (0.99 - 1.01)	<0.00
Q2	4928	0.66 (0.66 - 0.67)	0.99 (0.99 - 1.00)	
Q3	4928	0.66 (0.66 - 0.67)	0.99 (0.98 - 1.00)	
Q4	4928	0.66 (0.65 - 0.66)	0.99 (0.98 - 0.99)	
Q5 (Highest)	4927	0.65 (0.65 - 0.66)	0.98 (0.97 - 0.99)	
n-3 fatty acids				
Q1 (Lowest)	4928	0.67 (0.66 - 0.67)	1.00 (0.99 - 1.01)	0.264
Q2	4928	0.66 (0.66 - 0.66)	0.99 (0.99 - 1.00)	
Q3	4928	0.66 (0.66 - 0.67)	0.99 (0.99 - 1.00)	
Q4	4928	0.66 (0.66 - 0.67)	0.99 (0.99 - 1.00)	
Q5 (Highest)	4927	0.66 (0.66 - 0.66)	0.99 (0.99 - 1.00)	
n-6 fatty acids				
Q1 (Lowest)	4928	0.67 (0.66 - 0.67)	1.00 (0.99 - 1.01)	<0.00
Q2	4928	0.66 (0.66 - 0.67)	0.99 (0.98 - 1.00)	
Q3	4928	0.66 (0.66 - 0.67)	0.99 (0.98 - 1.00)	
Q4	4928	0.66 (0.65 - 0.66)	0.98 (0.98 - 0.99)	
Q5 (Highest)	4927	0.65 (0.65 - 0.66)	0.97 (0.97 - 0.98)	
Total protein				
Q1 (Lowest)	4928	0.66 (0.66 - 0.67)	1.00 (0.99 - 1.01)	0.988
Q2	4928	0.66 (0.65 - 0.66)	0.99 (0.99 - 1.00)	
Q3	4928	0.66 (0.66 - 0.67)	1.00 (0.99 - 1.01)	
Q4	4928	0.66 (0.66 - 0.67)	1.00 (0.99 - 1.01)	
Q5 (Highest)	4927	0.66 (0.66 - 0.67)	1.00 (0.99 - 1.00)	
Protein from plant				
Q1 (Lowest)	4928	0.66 (0.66 - 0.67)	1.00 (0.99 - 1.01)	0.773
Q2	4928	0.66 (0.66 - 0.67)	1.00 (0.99 - 1.01)	
Q3	4928	0.66 (0.66 - 0.67)	1.00 (1.00 - 1.01)	
Q4	4928	0.66 (0.66 - 0.67)	1.00 (0.99 - 1.01)	
Q5 (Highest)	4927	0.66 (0.65 - 0.66)	1.00 (0.99 - 1.00)	
Protein from animal sources				
Q1 (Lowest)	4928	0.66 (0.66 - 0.67)	1.00 (0.99 - 1.01)	0.736
Q2	4928	0.66 (0.65 - 0.66)	1.00 (0.99 - 1.00)	
Q3	4928	0.66 (0.66 - 0.67)	1.00 (1.00 - 1.01)	
Q4	4928	0.66 (0.66 - 0.67)	1.00 (1.00 - 1.01)	
Q5 (Highest)	4927	0.66 (0.66 - 0.66)	1.00 (0.99 - 1.01)	
Protein from dairy				
Q1 (Lowest)	4928	0.67 (0.66 - 0.67)	1.00 (0.99 - 1.01)	0.006
Q2	4928	0.66 (0.65 - 0.66)	0.99 (0.98 - 0.99)	
Q3	4928	0.66 (0.66 - 0.67)	0.99 (0.98 - 1.00)	
Q4	4928	0.66 (0.66 - 0.67)	0.99 (0.98 - 1.00)	
Q5 (Highest)	4927	0.66 (0.65 - 0.66)	0.98 (0.98 - 0.99)	
Protein from non-dairy animal sources				

Q1 (Lowest)	4928	0.67 (0.66 - 0.67)	1.00 (0.99 - 1.01)	<0.000
Q2	4928	0.66 (0.66 - 0.67)	0.99 (0.98 - 0.99)	
Q3	4928	0.66 (0.66 - 0.67)	0.99 (0.98 - 1.00)	
Q4	4928	0.66 (0.66 - 0.67)	0.99 (0.98 - 0.99)	
Q5 (Highest)	4927	0.65 (0.65 - 0.66)	0.98 (0.97 - 0.98)	
Fat from non-dairy animal sources				
Q1 (Lowest)	4928	0.66 (0.66 - 0.66)	1.00 (0.99 - 1.01)	0.5330
Q2	4928	0.66 (0.66 - 0.67)	1.00 (1.00 - 1.01)	
Q3	4928	0.66 (0.66 - 0.67)	1.00 (0.99 - 1.01)	
Q4	4928	0.66 (0.66 - 0.67)	1.00 (1.00 - 1.01)	
Q5 (Highest)	4927	0.66 (0.66 - 0.67)	1.00 (1.00 - 1.01)	
Q1 (Lowest)	4928	0.66 (0.65 - 0.66)	1.00 (0.99 - 1.01)	0.129
Q2	4928	0.66 (0.65 - 0.66)	1.00 (0.99 - 1.01)	
Q3	4928	0.66 (0.66 - 0.67)	1.00 (1.00 - 1.01)	
Q4	4928	0.67 (0.66 - 0.67)	1.01 (1.00 - 1.02)	
Q5 (Highest)	4927	0.66 (0.66 - 0.67)	1.00 (1.00 - 1.01)	

Models adjusted for age at recruitment (<45, 45-49, 50-54, 55-59, 60-64, >65), sex, ethnicity (white, mixed race, Asian or Asian British, black or black British, other, unknown), region (London, North-West England, North-Eastern England, Yorkshire & the Humber, West Midlands, East Midlands, South-West England, Wales), Townsend deprivation index (quintiles from least to most affluent, unknown), smoking status (never, previous, <15 cigarettes/day, 15-29 cigarettes/day, >30 cigarettes/day, unknown), physical activity (low, medium or high according to MET hours per week, unknown), alcohol (<1, 1-9, 10-19, 20-29, >30 g/day, unknown), body mass index (<20, 20-22.4, 22.5-24.9, 25-27.4, 27.5-29.9, 30-32.4, 32.5-34.9, 35-37.4, 37.5-39.9 or >40 in kg/m^2), height (sex-specific groups in 5cm increments, unknown), diabetes diagnosed by a doctor

Abbreviations: Apo = apolipoprotein; CI = confidence interval; g/day = grams per day; MUFA = monounsaturated fatty acids; n=3 = omega-3; n-6 = omega-6; PUFA = polyunsaturated fatty acids; Q = quintile; SFA = saturated fatty acids.

*For fiber Q1 and Q5 represent quintiles of total fiber intake (g/day).

p-trend was calculated by modelling quintiles of macronutrient intake as continuous variables in the regression model.

Supplemental Table XI. Percentage change in serum lipids for every 5% of energy intake increase in macronutrients (main model) and percentage change in serum lipids for a 5% of energy intake isoenergetic replacement of saturated fatty acids with other macronutrients (isoenergetic substitution model).

	Total cholesterol (mmol/L)	LDL-C (mmol/L)	HDL-C (mmol/L)	Triglycerides (mmol/L)	TC-to-HDL-C ratio	TG-to-HDL-C ratio	ApoB (mmol/L)	ApoA1 (mmol/L)	ApoB-to-ApoA1 ratio
<u>**Main model ***</u>									
**Change in lipid marker for every 5% increase in:**									
Carbohydrate from free sugars									
Absolute Δ in geometric mean (95% CI)	0.01 (0.00, 0.02)	0.02 (0.01, 0.03)	-0.02 (-0.03, -0.02)	0.06 (0.05, 0.07)	0.08 (0.07, 0.09)	0.07 (0.06, 0.08)	0.01 (0.00, 0.01)	-0.01 (-0.02, -0.01)	0.01 (0.01, 0.01)
% Δ in geometric mean Δ (95% CI)	0.17% (-0.05%, 0.40%)	0.59% (0.31%, 0.86%)	-1.70% (-1.96%, -1.44%)	4.04% (3.45%, 4.63%)	1.88% (1.59%, 2.16%)	5.74% (5.01%, 6.48%)	0.64% (0.36%, 0.92%)	-0.91% (-1.10%, -0.72%)	1.55% (1.21%, 1.89%)
Carbohydrate from non-free sugars									
Absolute Δ in geometric mean (95% CI)	-0.04 (-0.05, -0.02)	-0.03 (-0.04, -0.02)	0.00 (0.00, 0.00)	-0.03 (-0.04, -0.02)	-0.02 (-0.04, -0.01)	-0.02 (-0.03, -0.01)	-0.01 (-0.01, 0.00)	-0.01 (-0.01, -0.01)	0.00 (0.00, 0.00)
% Δ in geometric mean Δ (95% CI)	-0.62% (-0.83%, -0.40%)	-0.70% (-0.97%, -0.43%)	0.00% (-0.26%, 0.25%)	-1.68% (-2.25%, -1.11%)	-0.61% (-0.89%, -0.34%)	-1.67% (-2.38%, -0.96%)	-0.57% (-0.83%, -0.30%)	-0.58% (-0.76%, -0.40%)	0.01% (-0.31%, 0.34%)
Carbohydrate from whole grains									
Absolute Δ in geometric mean (95% CI)	-0.04 (-0.06, -0.03)	-0.03 (-0.05, -0.02)	0.00 (-0.01, 0.00)	0.00 (-0.01, 0.01)	-0.03 (-0.04, -0.01)	-0.01 (-0.02, 0.01)	-0.01 (-0.01, 0.00)	-0.01 (-0.01, 0.00)	0.00 (-0.01, 0.00)
% Δ in geometric mean Δ (95% CI)	-0.72% (-0.99%, -0.46%)	-0.89% (-1.22%, -0.56%)	-0.13% (-0.44%, 0.18%)	-0.14% (-0.84%, 0.57%)	-0.59% (-0.93%, -0.25%)	-0.01% (-0.88%, 0.87%)	-0.77% (-1.10%, -0.44%)	-0.44% (-0.66%, -0.21%)	-0.33% (-0.74%, 0.07%)
Carbohydrate from refined grains									
Absolute Δ in geometric mean (95% CI)	-0.02 (-0.03, -0.01)	-0.01 (-0.02, 0.00)	-0.01 (-0.01, -0.01)	0.02 (0.01, 0.03)	0.01 (0.00, 0.03)	0.02 (0.01, 0.03)	0.00 (-0.01, 0.00)	0.00 (-0.01, 0.00)	0.00 (0.00, 0.00)
% Δ in geometric mean (95% CI)	-0.33% (-0.52%, -0.14%)	-0.35% (-0.59%, -0.11%)	-0.66% (-0.88%, -0.43%)	1.14% (0.63%, 1.65%)	0.32% (0.08%, 0.57%)	1.80% (1.16%, 2.43%)	-0.32% (-0.56%, -0.09%)	-0.27% (-0.43%, -0.11%)	-0.06% (-0.35%, 0.24%)
SFA									
Absolute Δ in geometric mean (95% CI)	0.14 (0.12, 0.17)	0.10 (0.08, 0.12)	0.04 (0.04, 0.05)	-0.05 (-0.07, -0.03)	-0.02 (-0.04, 0.00)	-0.07 (-0.09, -0.05)	0.01 (0.01, 0.02)	0.04 (0.04, 0.05)	-0.01 (-0.01, 0.00)
% Δ in geometric mean (95% CI)	2.49% (2.10%, 2.88%)	2.82% (2.34%, 3.31%)	2.87% (2.41%, 3.33%)	-2.47% (-3.51%, -1.44%)	-0.38% (-0.88%, 0.12%)	-5.35% (-6.63%, -4.06%)	1.50% (1.02%, 1.98%)	2.56% (2.23%, 2.89%)	-1.06% (-1.65%, -0.46%)
MUFA									
Absolute Δ in geometric mean (95% CI)	0.11 (0.08, 0.13)	0.06 (0.04, 0.08)	0.05 (0.04, 0.06)	-0.06 (-0.09, -0.04)	-0.06 (-0.09, -0.04)	-0.09 (-0.11, -0.06)	0.01 (0.01, 0.02)	0.05 (0.04, 0.06)	-0.01 (-0.02, -0.01)
% Δ in geometric mean (95% CI)	1.83% (1.37%, 2.30%)	1.54% (0.96%, 2.12%)	3.44% (2.89%, 3.99%)	-4.28% (-5.52%, -3.04%)	-1.61% (-2.21%, -1.00%)	-7.72% (-9.25%, -6.18%)	1.38% (0.80%, 1.96%)	3.14% (2.75%, 3.54%)	-1.76% (-2.47%, -1.05%)
PUFA									
Absolute Δ in geometric mean (95% CI)	-0.04 (-0.08, 0.00)	-0.06 (-0.09, -0.03)	0.05 (0.03, 0.06)	-0.12 (-0.16, -0.09)	-0.16 (-0.20, -0.12)	-0.14 (-0.17, -0.10)	-0.01 (-0.02, 0.00)	0.04 (0.03, 0.05)	-0.02 (-0.03, -0.01)
% Δ in geometric mean (95% CI)	-0.75% (-1.47%, -0.03%)	-1.87% (-2.76%, -0.98%)	3.25% (2.41%, 4.09%)	-7.84% (-9.74%, -5.94%)	-4.00% (-4.92%, -3.08%)	-11.09% (-13.44%, -8.73%)	-0.85% (-1.74%, 0.04%)	2.33% (1.73%, 2.93%)	-3.18% (-4.27%, -2.09%)
Protein									
Absolute Δ in geometric mean (95% CI)	0.02 (0.00, 0.04)	0.00 (-0.02, 0.02)	0.03 (0.02, 0.03)	-0.05 (-0.07, -0.03)	-0.05 (-0.07, -0.03)	-0.05 (-0.07, -0.03)	0.00 (0.00, 0.01)	0.01 (0.01, 0.02)	0.00 (-0.01, 0.00)
% Δ in geometric mean (95% CI)	0.33% (-0.05%, 0.71%)	-0.05% (-0.52%, 0.42%)	1.68% (1.23%, 2.12%)	-3.71% (-4.72%, -2.71%)	-1.35% (-1.83%, -0.86%)	-5.39% (-6.64%, -4.14%)	0.38% (-0.09%, 0.85%)	0.78% (0.46%, 1.10%)	-0.39% (-0.97%, 0.19%)
<u>**Isoenergetic substitution model †**</u>									
**For each substitution of 5% SFA by:**									
Carbohydrate from free sugars									
Absolute Δ in geometric mean (95% CI)	-0.06 (-0.11, -0.02)	-0.04 (-0.07, 0.00)	-0.06 (-0.07, -0.04)	0.13 (0.10, 0.17)	0.13 (0.09, 0.17)	0.16 (0.12, 0.19)	0.01 (0.00, 0.02)	-0.04 (-0.05, -0.03)	0.03 (0.02, 0.04)
% Δ in geometric mean Δ (95% CI)	-1.03% (-1.78%, -0.28%)	-0.98% (-1.91%, -0.05%)	-3.84% (-4.71%, -2.96%)	6.64% (4.66%, 8.62%)	2.81% (1.85%, 3.77%)	10.48% (8.03%, 12.93%)	1.25% (0.33%, 2.18%)	-2.60% (-3.23%, -1.97%)	3.85% (2.71%, 4.99%)
Carbohydrate from non-free sugars									

Absolute Δ in geometric mean (95% CI)	-0.08 (-0.12, -0.04)	-0.06 (-0.09, -0.03)	-0.03 (-0.04, -0.02)	0.06 (0.02, 0.09)	0.05 (0.01, 0.08)	0.08 (0.04, 0.11)	0.00 (-0.01, 0.01)	-0.03 (-0.04, -0.02)	0.02 (0.01, 0.02)
% Δ in geometric mean Δ (95% CI)	-1.34% (-2.03%, -0.65%)	-1.58% (-2.44%, -0.72%)	-2.20% (-3.00%, -1.39%)	1.92% (0.09%, 3.74%)	0.86% (-0.02%, 1.75%)	4.11% (1.85%, 6.37%)	0.35% (-0.51%, 1.20%)	-2.00% (-2.58%, -1.42%)	2.35% (1.30%, 3.40%)
Carbohydrate from whole grains									
Absolute Δ in geometric mean (95% CI)	-0.10 (-0.14, -0.06)	-0.08 (-0.11, -0.05)	-0.04 (-0.06, -0.03)	0.10 (0.06, 0.13)	0.05 (0.01, 0.09)	0.11 (0.08, 0.14)	0.00 (-0.01, 0.01)	- -0.03 (-0.04, -0.02)	0.01 (0.01, 0.02)
% Δ in geometric mean (95% CI)	-1.75% (-2.45%, -1.05%)	-2.10% (-2.97%, -1.23%)	-2.86% (-3.68%, -2.05%)	4.61% (2.77%, 6.46%)	1.11% (0.21%, 2.01%)	7.48% (5.19%, 9.77%)	-0.20% (-1.06%, 0.67%)	-2.14% (-2.72%, -1.55%)	1.94% (0.88%, 3.01%)
Carbohydrate from refined grains									
Absolute Δ in geometric mean (95% CI)	-0.10 (-0.14, -0.06)	-0.07 (-0.10, -0.04)	-0.05 (-0.06, -0.03)	0.10 (0.07, 0.14)	0.08 (0.04, 0.11)	0.12 (0.09, 0.16)	0.00 (-0.01, 0.01)	- -0.03 (-0.04, -0.02)	0.02 (0.01, 0.02)
% Δ in geometric mean (95% CI)	-1.63% (-2.33%, -0.93%)	-1.92% (-2.78%, -1.05%)	-3.20% (-4.01%, -2.38%)	4.78% (2.93%, 6.63%)	1.56% (0.67%, 2.46%)	7.98% (5.69%, 10.26%)	0.07% (-0.80%, 0.93%)	-2.17% (-2.76%, -1.59%)	2.24% (1.18%, 3.31%)
MUFA									
Absolute Δ in geometric mean (95% CI)	0.00 (-0.07, 0.07)	-0.01 (-0.06, 0.04)	-0.03 (-0.05, -0.01)	0.13 (0.07, 0.19)	0.10 (0.04, 0.17)	0.14 (0.08, 0.20)	0.02 (0.01, 0.04)	-0.01 (-0.02, 0.01)	0.02 (0.01, 0.03)
% Δ in geometric mean (95% CI)	0.16% (-1.03%, 1.35%)	-0.09% (-1.57%, 1.38%)	-1.93% (-3.31%, -0.54%)	5.28% (2.14%, 8.43%)	2.09% (0.56%, 3.62%)	7.21% (3.32%, 11.10%)	2.21% (0.73%, 3.68%)	-0.54% (-1.54%, 0.46%)	2.74% (0.93%, 4.56%)
PUFA									
Absolute Δ in geometric mean (95% CI)	-0.19 (-0.24, -0.13)	-0.15 (-0.19, -0.11)	-0.01 (-0.03, 0.00)	-0.06 (-0.10, -0.01)	-0.10 (-0.15, -0.05)	-0.04 (-0.09, 0.01)	-0.02 (-0.03, -0.01)	-0.02 (-0.03, -0.01)	0.00 (-0.01, 0.01)
% Δ in geometric mean (95% CI)	-3.39% (-4.36%, -2.42%)	-4.48% (-5.68%, -3.28%)	-0.80% (-1.93%, 0.32%)	-3.83% (-6.38%, -1.27%)	-2.59% (-3.83%, -1.34%)	-3.02% (-6.19%, 0.15%)	-2.22% (-3.42%, -1.02%)	-1.30% (-2.11%, -0.48%)	-0.92% (-2.40%, 0.55%)
Protein									
Absolute Δ in geometric mean (95% CI)	-0.05 (-0.09, -0.01)	-0.04 (-0.08, -0.01)	-0.03 (-0.04, -0.01)	0.07 (0.04, 0.11)	0.06 (0.02, 0.10)	0.09 (0.05, 0.12)	0.01 (0.00, 0.02)	-0.02 (-0.03, -0.01)	0.02 (0.01, 0.03)
% Δ in geometric mean (95% CI)	-0.87% (-1.61%, -0.13%)	-1.29% (-2.21%, -0.38%)	-1.89% (-2.75%, -1.03%)	2.32% (0.37%, 4.27%)	1.02% (0.08%, 1.97%)	4.21% (1.80%, 6.63%)	1.08% (0.16%, 1.99%)	-1.61% (-2.23%, -0.99%)	2.68% (1.56%, 3.81%)

*Main model included the % of total energy intake for the nutrient of interest (expressed in 5% increments) and daily total energy intake (kJ) and was adjusted for covariates (see below). Concentrations are expressed as geometric means.

† Isoenergetic substitution model included % of total energy intake from carbohydrates, % of total energy intake from MUFA, % of total energy intake from PUFA, % of total energy intake from protein, daily total energy intake (kJ), and was adjusted for covariates (see below).

Models adjusted for age at recruitment (<45, 45-49, 50-54, 55-59, 60-64, >65), sex, ethnicity (white, mixed race, Asian or Asian British, black or black British, other, unknown), region (London, North-West England, North-Eastern England, Yorkshire & the Humber, West Midlands, East Midlands, South-West England, Wales), Townsend deprivation index (quintiles from least to most affluent, unknown), smoking status (never, previous, <15 cigarettes/day, 15-29 cigarettes/day, >30 cigarettes/day, unknown), physical activity (low, medium or high according to MET hours per week, unknown), alcohol (<1, 1-9, 10-19, 20-29, >30 g/day, unknown), body mass index (<20, 20-22.4, 22.5-24.9, 25-27.4, 27.5-29.9, 30-32.4, 32.5-34.9, 35-37.4, 37.5-39.9 or >40 in kg/m^2), height (sex-specific groups in 5cm increments, unknown) and diabetes diagnosed by a doctor (yes, no, unknown).

Abbreviations: Apo = apolipoprotein; CI = confidence interval; g/day = grams per day; HDL-C = high-density lipoprotein cholesterol; kJ = kilojoules; LDL-C = low-density lipoprotein cholesterol; MUFA = monounsaturated fatty acids; PUFA = polyunsaturated fatty acids; Q = quintile; SFA = saturated fatty acids; TC = total cholesterol; TEI = total energy intake; TG = triglycerides.

Supplemental Table XII. Sample aliquot number for lipid marker measurements at baseline in 24,639 UK Biobank participants.

Lipid Marker	Manual	Aliquot 1	Aliquot 2	Missing
Total cholesterol	543 (2.20%)	23,183 (94.10%)	881 (3.58%)	32 (0.13%)
LDL-C	543 (2.20%)	23,183 (94.10%)	881 (3.58%)	32 (0.13%)
HDL-C	543 (2.20%)	23,183 (94.10%)	881 (3.58%)	32 (0.13%)
Triglycerides	543 (2.20%)	23,183 (94.10%)	881 (3.58%)	32 (0.13%)
ApoB	543 (2.20%)	23,183 (94.10%)	881 (3.58%)	32 (0.13%)
ApoA1	543 (2.20%)	23,183 (94.10%)	881 (3.58%)	32 (0.13%)

Abbreviations: Apo = apolipoprotein; HDL-C = high-density lipoprotein cholesterol; LDL-C = low-density lipoprotein cholesterol.

Supplemental Table XIII. List of lipid-modifying drug names and codes obtained from baseline touchscreen questionnaire used to exclude participants taking lipid modifying medication (n=84,008).

Coding	Description
1140861848	colestid 5g/sachet granules
1140861856	gemfibrozil
1140861858	lopid 300 capsule
1140861866	nicofuranose
1140861868	nicotinic acid product
1140861876	probucol
1140861878	lurselle 250mg tablet
1140861922	lipid lowering drug
1140861924	bezafibrate
1140861926	bezalip 200mg tablet
1140861928	bezalip-mono 400mg m/r tablet
1140861936	questran 4g/sachet powder
1140861942	cholestyramine+aspartame 4g/sachet powder
1140861944	clofibrate
1140861946	atromid-s 500mg capsule
1140861954	fenofibrate
1140861958	simvastatin
1140862026	ciprofibrate
1140862028	modalim 100mg tablet
1140864592	lescol 20mg capsule
1140865576	cholestyramine
1140881748	zocor 10mg tablet
1140888590	colestipol
1140888594	fluvastatin
1140888648	pravastatin
1140910632	eptastatin
1140910654	velastatin
1140910670	niacin
1141146138	lipitor 10mg tablet
1141146234	atorvastatin
1141157260	bezafibrate product
1141157262	gemfibrozil product
1141157416	cholestyramine product
1141188546	niaspan 500mg m/r tablet
1141192410	rosuvastatin
1141192414	crestor 10mg tablet
1141192736	ezetimibe
1141200040	zocor heart-pro 10mg tablet

MAJOR RESOURCES TABLE

In order to allow validation and replication of experiments, all essential research materials listed in the Methods should be included in the Major Resources Table below. Authors are encouraged to use public repositories for protocols, data, code, and other materials and provide persistent identifiers and/or links to repositories when available. Authors may add or delete rows as needed.

Data & Code Availability

Description	Source / Repository	Persistent ID / URL
Bona fide researchers can apply to use the UK Biobank dataset by registering and applying at	UK Biobank	http://ukbiobank.ac.uk/register-apply/.

REFERENCES

1. Liu B, Young H, Crowe FL, Benson VS, Spencer EA, Key TJ, Appleby PN, Beral V. Development and evaluation of the Oxford WebQ, a low-cost, web-based method for assessment of previous 24 h dietary intakes in large-scale prospective studies. *Public Health Nutr.* 2011;14:1998-2005.

2. Greenwood DC, Hardie LJ, Frost GS, et al. Validation of the Oxford WebQ online 24-hour dietary questionnaire using biomarkers. *Am J Epidemiol.* 2019;188:1858-1867.

3. Carter JL, Lewington S, Piernas C, Bradbury K, Key TJ, Jebb SA, Arnold M, Bennett D, Clarke R. Reproducibility of dietary intakes of macronutrients, specific food groups, and dietary patterns in 211 050 adults in the UK Biobank study. *J Nutr Sci*. 2019;8:e34.

4. Galante J, Adamska L, Young A, Young H, Littlejohns TJ, Gallacher J, Allen N. The acceptability of repeat Internet-based hybrid diet assessment of previous 24-h dietary intake: administration of the Oxford WebQ in UK Biobank. *Br J Nutr*. 2016;115:681-686.

5. UK Biobank. UK Biobank: blood sample collection, processing and transport version 1.0. https://biobank.ctsu.ox.ac.uk/crystal/crystal/docs/Bloodsample.pdf. Accessed September 10, 2020.

6. Elliott P, Peakman TC. The UK Biobank sample handling and storage protocol for the collection, processing and archiving of human blood and urine. *Int J Epidemiol.* 2008;37:234-244.

7. Fry S, Almond R, Moffat S, Gordon M, Singh P. UK Biobank biomarker project: companion document to accompany serum biomarker data. https://biobank.ndph.ox.ac.uk/showcase/showcase/docs/serum_biochemistry.pdf. Accessed September 10, 2020.

8. Mora S, Martin SS, Virani SS. Cholesterol insights and controversies from the UK Biobank study. *Circulation*. 2019;140:553-555.

9. Bradbury KE, Guo W, Cairns BJ, Armstrong ME, Key TJ. Association between physical activity and body fat percentage, with adjustment for BMI: a large cross-sectional analysis of UK Biobank. *BMJ Open.* 2017;7:e011843.

10. National Institute for Health and Care Excellence. Lipid-modifying drugs. https://www.nice.org.uk/advice/ktt3/resources/lipidmodifying-drugs-pdf-1632168482245. Accessed September 10, 2020.

11. National Health Service. Medicines A to Z. https://www.nhs.uk/medicines/. Accessed September 10, 2020.
