# Supplementary figures and images for: Associations Between Macronutrients From Different Dietary Sources and Serum Lipids in 24 639 UK Biobank Study Participants
Source: Arterioscler Thromb Vasc Biol. 2021 May 27;41(7):2190–200. doi: 10.1161/ATVBAHA.120.315628 (PMC8216602; doi:10.1161/ATVBAHA.120.315628)

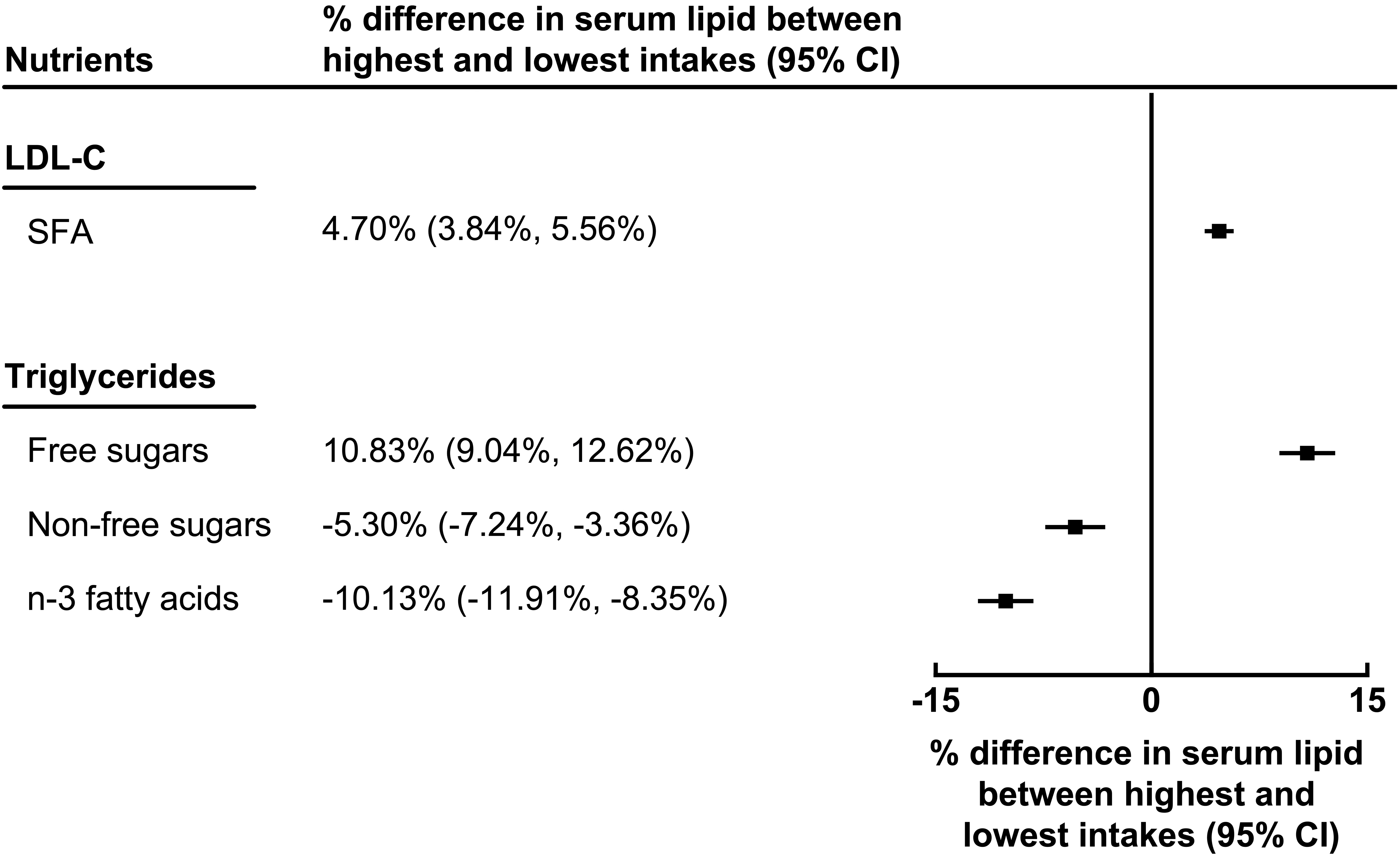

Supplement: Supplementary file 2 [file atv-41-2190-s002.jpg]
